# Stereoselective Chemoenzymatic Cascades for the Synthesis of Densely Functionalized Iminosugars

**DOI:** 10.1021/jacs.4c16732

**Published:** 2025-02-10

**Authors:** Christopher R. B. Swanson, Léa Gourbeyre, Grayson J. Ford, Pere Clapés, Sabine L. Flitsch

**Affiliations:** † Manchester Institute of Biotechnology, School of Chemistry, The University of Manchester, 131 Princess Street, M1 7DN Manchester, United Kingdom; ‡ Biological Chemistry Department, Institute for Advanced Chemistry of Catalonia, IQAC−CSIC, Barcelona 08034, Spain

## Abstract

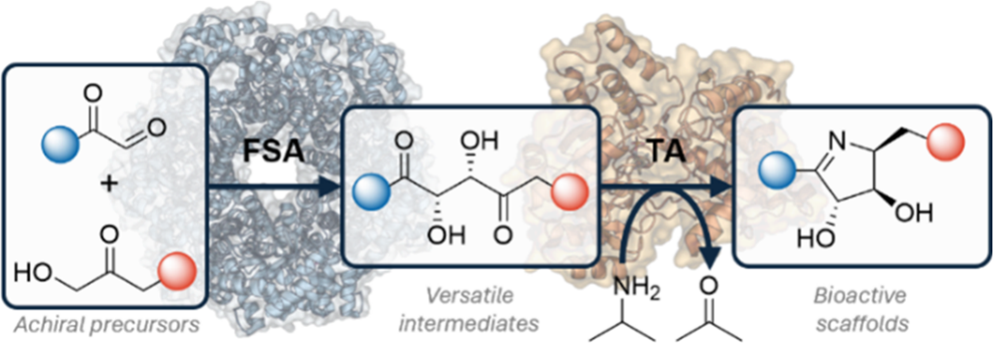

1,4-Dicarbonyls are versatile synthons for the construction of diverse pharmacophores and natural products. However, the stereoselective synthesis of densely functionalized 1,4-dicarbonyls is challenging. Here, we report a versatile biocatalytic route to access chiral 2,3-dihydroxy-1,4-diketones in high yields and up to gram scale using d-fructose-6-phosphate aldolase (*Ec*FSA). The utility of these compounds as synthons is exemplified in enzyme cascades with subsequent regio- and stereoselective enzymatic transamination to form densely functionalized homochiral 1-pyrrolines followed by chemical or enzymatic reduction to tetrasubstituted pyrrolidines.

## Introduction

**Figure 1:**
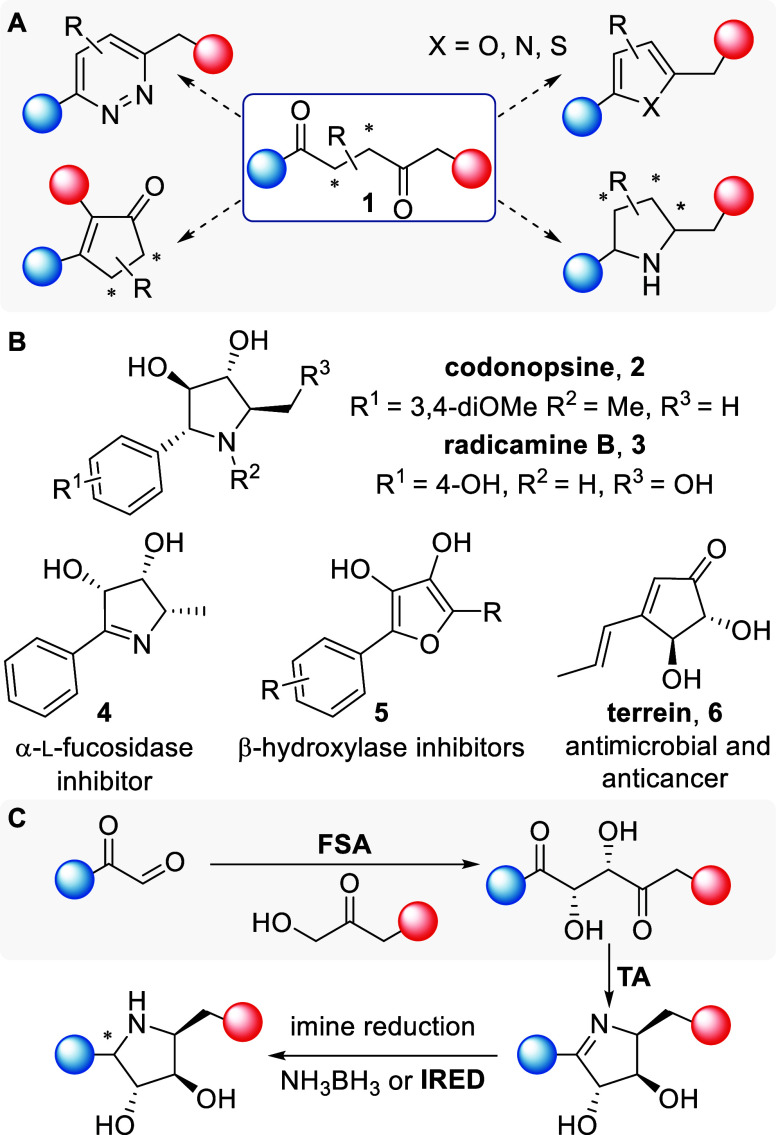
(A) Functionalized 1,4-diketone synthons as versatile intermediates. (B) Examples of polyhydroxylated targets (C) A proposed (chemo)-enzymatic cascade for the modular synthesis and diversification of chiral 2,3-dihydroxylated-1,4-dicarbonyls (FSA: aldolase, TA: transaminase, IRED: imine reductase).

1,4-Dicarbonyl compounds are versatile synthetic intermediates for the synthesis of heterocycles and bioactive fragments.^1^ 2,3-Disubstituted-1,4-diketones (**1**) are particularly valuable for generation of highly functionalized heterocycles (Figure 1A,B) including pyrroles,^2,3^ thiophenes,^4^ pyridazines,^5^ pyrrolidines (**2** and **3**), pyrrolines (**4**),^6−8^ and furans (**5**).^9,10^ The 1,4-diketone moiety is also present in natural products and can be converted into pharmacophores such as sugar polyols, diketone sugars or cyclopentenones (**6**, Figure 1).^11−14^

In comparison to 1,3- and 1,5-dicarbonyls, the synthesis of 1,4-diketones is a challenging process due to the inherent mismatch in carbonyl polarity. Consequently, these compounds are often synthesized by umpolung type chemistries that leverage inverse carbonyl reactivity.^1^ A classic example of this approach is the Stetter reaction; a 1,4 conjugate addition of an aldehyde onto an α,β-unsaturated carbonyl which proceeds via an *N*-heterocyclic carbene intermediate. Recent developments allow for enantioselective variants of this reaction and have proven to be a versatile tool for 1,4-dicarbonyl synthesis.^1,15−18^ Other chemical approaches to dicarbonyls include radical additions,^15,19,20^ enolate couplings^21,22^ and alkene carbonylation.^23^ Organocatalytic aldol reactions have also recently been reported to allow access to a panel of chiral 2,3-disubstituted-1,4-diketones.^24,25^ Despite the plethora of synthetic methodologies for preparation of 1,4-dicarbonyls, the stereoselective synthesis of 2,3-disubstituted nonsymmetrical 1,4-diketones with high enantiopurity remains a significant challenge.^1,15,17,26^ Furthermore, the requirement for protection strategies hampers the synthesis and diversification of polyhydroxylated targets such as iminosugars **2**, **3** and **4** (Figure 1B).

Biocatalysis is an attractive technology for the enantioselective synthesis of complex fragments without the need for protecting groups.^27,28^ Enzymatic catalysis of 1,4-dicarbonyl forming Stetter reactions was discovered using thiamine diphosphate (ThDP)-dependent carboligases such as the biosynthetic enzymes PigD and MenD.^29,30^ In these enzymes, the umpolung reactive intermediate is generated by decarboxylation of an α-keto acid (e.g., pyruvate or α-ketoglutarate).^29−33^ Similar reactivity has also been described within benzaldehyde lyase (BAL), another ThDP-dependent lyase that typically catalyzes 1,2 additions similar to benzoin reaction.^34^ Chen et al. showed that benzaldehyde lyase was capable of performing intramolecular Stetter additions to furnish chroman-4-one scaffolds in good to excellent yields and enantiopurity. More recently, MacAulay et al. demonstrated that conjugation of a thiamine-inspired *N*-heterocyclic carbene to a steroid carrier protein creates an artificial “Stetterase” from an otherwise inert protein scaffold.^35^ Enzymatic, regiospecific oxidation of sugars can also yield 1,4-diketones and ketoaldehydes. In this way, a number of synthetic building blocks and potential artificial sweeteners have been synthesized.^13,14,36^

The synthetic approach developed in this work exploits d-fructose-6-phosphate aldolase (FSA) to generate a library of 2,3-dihydroxylated-1,4-diketones from simple, achiral precursors (Figure 1C). FSA has a broad scope for both nucleophile and electrophile substrates including highly electrophilic methylglyoxal, hydroxypyruvaldehyde phosphate (HPP), and 2,3-dihydroxy-3-phenylpropanal as electrophiles.^37−43^ In these cases the diketone or 5-hydroxyketone products spontaneously cyclize to produce flavor and fragrance compounds, diulose sugars or C6-aryl carbohydrates.^43−46^ Thus, we sought to expand the electrophile scope of FSA to arylated glyoxals and produce chiral 2,3-dihydroxy-1,4-diketones flanked by aryl and alkyl substituents (Figure 1C).

As part of our ongoing research interest in the synthesis of natural and abiotic aminopolyols and iminosugars^47,48^ it was envisaged that 2,3-dihydroxylated-1,4-diketones would serve as ideal intermediates toward 2-aryl iminosugars. As illustrated by the plant alkaloid natural products codonopsine and radicamine B (**2** and **3**, Figure 1B),^8,49−51^ these uncommon arylated iminosugars (and their precursor imines, e.g., **4**) are glycosidase inhibitors and antibiotics (MRSA).^52,53^ Some analogous compounds also inhibit nucleoside hydrolases, acting as *C*-nucleoside mimics.^54,55^ Based on precedents for biocatalytic synthesis of chiral aminopolyols and iminosugars;^47,56,57^ the diversification of the FSA derived compounds with transaminase (TA) and imine reductase (IRED) biocatalysts was explored (Figure 1C). However, the transformation of complex targets with multiple reactive centers and hydroxyl substituents is not trivial and strict regio- and stereochemical control would be key to success of this strategy.^47,48,58^ The goals of this approach were: (i) to enable synthetic diversity through exploration of enzyme substrate scope, (ii) to evaluate the synthetic versatility of intermediates, and (iii) to identify novel enzyme activity on challenging polyhydroxylated substrates.

## Results [Tc16]and Discussion

### Screening of *Ec*FSA for Activity toward Aryl Glyoxals

**Figure 2:**
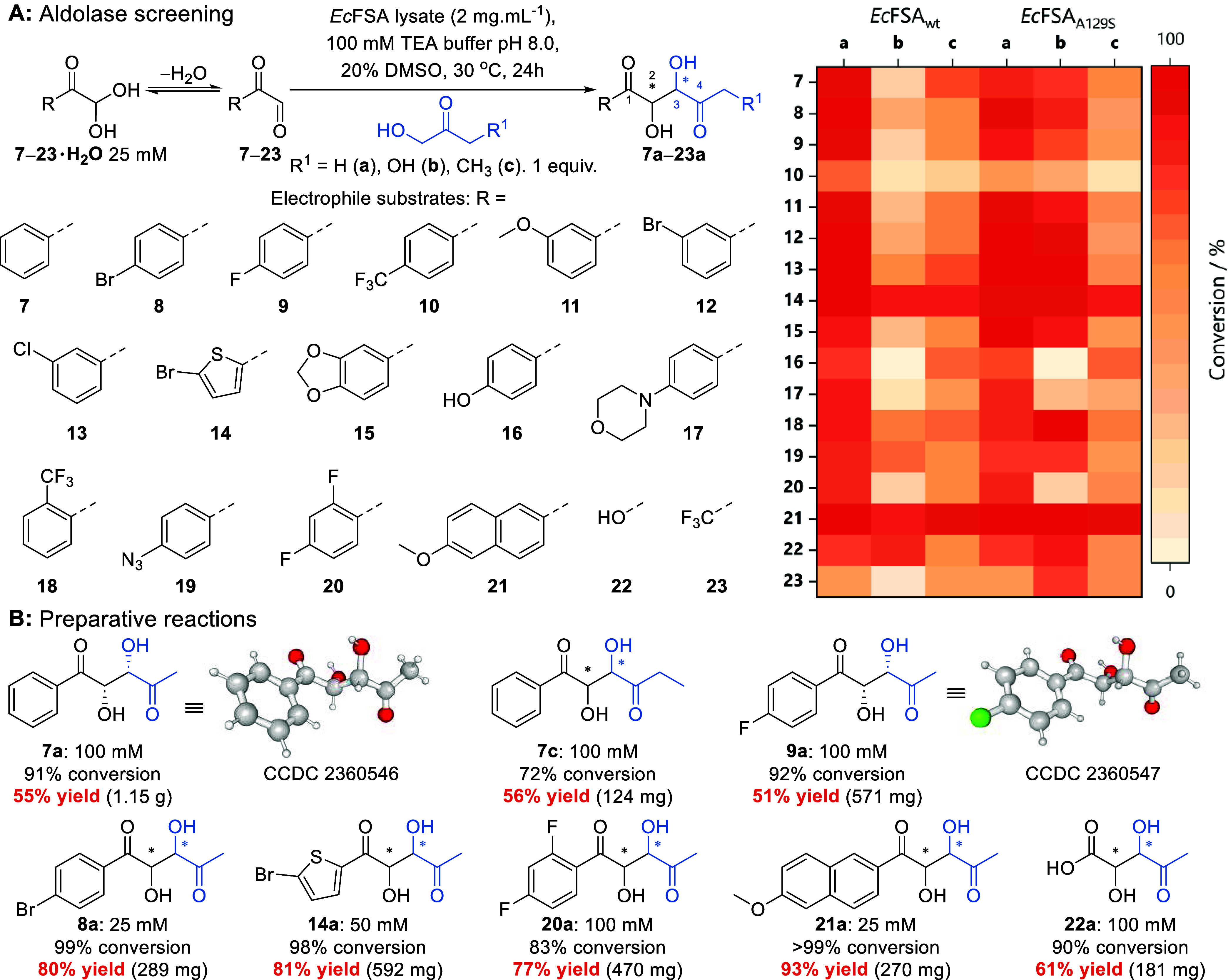
(A) *Ec*FSA-catalyzed aldol addition of hydroxyketones into glyoxals and a heatmap of screening data for *Ec*FSA (wild-type or A129S mutant) with electrophiles **7**–**23** and hydroxyacetone **a**, dihydroxyacetone **b** or 1-hydroxybutan-2-one **c**. Conversions are calculated from substrate depletion by UPLC-QDa for **7**–**21** or NMR for **22** and **23**. (B) Preparative reactions catalyzed by *Ec*FSAwt. Reaction conditions: *Ec*FSA lysate (2 mg·mL^–1^), glyoxal substrate **7**–**23** (25 mM), hydroxyacetone (1 equiv. Twenty-five mM) in triethanolamine buffer (TEA, 100 mM, pH 8.0) containing 20 v% DMSO. Reactions were incubated at 30 °C for 24 h. Preparative reactions were performed with increased substrate loading as specified.

The wild-type *Ec*FSA was first evaluated for its activity in the aldol addition of hydroxyacetone (HA, **a**) to phenylglyoxal **7**. Good conversion (91%) was observed and the panel of electrophile substrates was expanded(**7**–**23**, Figure 2A). Additionally, dihydroxyacetone (DHA, **b**) and 1-hydroxybutan-2-one (HB, **c**) were screened as nucleophile substrates. Based on previous protein engineering work, *Ec*FSA variant A129S was also screened, as it has shown improved conversion in reactions with DHA (**b**).^59^

UPLC-QDa analysis of biotransformations showed good to high conversions (49–99%) for both *Ec*FSA wt and A129S across the panel of substrates with HA (**a**, Figure 2A) and moderate conversions with DHA and HB (**b** 0–96% and **c** 22–90%, Figure 2A). Prior work with these aldolases have shown that the structural and electronic influence of aryl moieties can affect the acceptance of electrophile substrates.^60^ Indeed, the only previous examples of FSA acting upon related 2-oxoaldehydes utilize highly electrophilic 2-oxoalkanals as the electrophilic substrate. The electrophile substrate scope of *Ec*FSA tested herein was broad, and a range of functionality and substitution patterns were accepted (Figure 2A). Chemically useful handles such as brominated and chlorinated compounds **8**, **12** and **13** showed higher conversions than the unsubstituted phenylglyoxal **7** and an azidated derivative (**19**) was also well accepted. Free and methylated hydroxyl groups were accepted (**11**, **15** and **16**), as well as fluorinated derivatives (**9**, **10**, **18**, and **20**). Surprisingly, the bulky 6-methoxynaphthylglyoxal **21** was the best substrate, highlighting the potential of *Ec*FSA to generate diverse product libraries. The nonarylated substrates, glyoxylic acid **22** and trifluoropyruvaldehyde **23**, were also accepted, demonstrating a broader utility for the *Ec*FSA-catalyzed synthesis of 1,4-keto acids and nonsymmetric-1,4-diketones bearing two different alkyl substituents (Figure 2). No detrimental effects of the hydrate-aldehyde equilibrium of the glyoxal substrates were observed suggesting that the aldolase reaction has a sufficient driving force to outcompete this equilibrium.

### Preparative-Scale Aldolase Reactions and Chiral Analysis

Aldolase reactions were scaled up at increased substrate loading when feasible (100 mM with 10–20 v% DMSO). However, due to solubility limitations the scale up was performed at 25 mM for **8** and **21** and 50 mM for **14**. Products were purified by extraction and column chromatography where necessary. Accordingly, aldol products **7a**, **7c**, **8a**, **9a**, **14a**, **20a**, **21a** and **22a** were isolated in 51% to 93% yield (124 mg–1.15 g, Figure 2B). The chirality of aldol adducts **7a** and **9a** were confirmed to be the (2*S,3S*) stereoisomer by X-ray crystallography (CCDC 2360546 and 2360547). This is consistent with the FSA literature as C2 has inverted CIP priorities due to the adjacent ketone functionality. Chiral HPLC and NMR analysis of crude and purified products also indicates formation of a single major stereoisomer (Figures S32–S36). The electrophile scope and stereoselectivity of the reactions catalyzed by *Ec*FSA are complementary to a reported organocatalytic method, while enabling the use of equimolar substrate concentrations and additional nucleophilic substrates.^25^

With a library of aldolase derived diketones in hand, the enzymatic diversification of these compounds was investigated. Previously, simple 1,4-diketones have been utilized as substrates in the enzymatic cascade synthesis of pyrrolidine alkaloids using transaminase (TA), imine reductase (IRED) and monoamine oxidase biocatalysts.^6,7,61−64^ However, the synthesis of tetrasubstituted pyrrolidines in this manner remains challenging due to the unavailability of the corresponding 1,4-diketone precursors.^6,7,61,63^ By expanding upon these existing methodologies, the aldolase derived 2,3-dihydroxy-1,4-dicarbonyls provide an ideal test-bed to demonstrate the chemoselectivity of biocatalysis on substrates with multiple reactive sites. The decision to screen transaminase and reductase biocatalysts was bolstered by successful implementation of these enzymes in concert in the literature, where chiral aminopolyols, γ-hydroxy-α-amino acids and valuable heterocycles were synthesized.^37,47,56,65−70^ However, the question remained as to how applicable these strategies would be on complex targets with multiple reactive centers and hydroxyl substituents.

### Screening of TA for Activity toward FSA Aldol Products

A panel of transaminase biocatalysts was evaluated for the transamination of the aldol adduct **7a**.^71−73^ A screen of biotransformations was performed using 5 mM substrate **7a** and 5 equiv of l-alanine as a common amine donor. Three enzymes, the commercial ATA 113 from Codexis, RhTA^74^ from *Rhodobacter sphaeroides* and pQR2191 TA^75^ from a household drain metagenome were found to generate a new signal in the ^1^H NMR corresponding to the product **7ai** in 11, 15, and 37% conversion, respectively (**SI**Figures S39 and S40). The metagenomic transaminase pQR2191, which is known to be active against pharmaceutically relevant cyclic ketones and ketose sugars, was taken forward for reaction optimization and implementation into the FSA-TA cascade.^75,76^

### Optimization of the Transamination Step and FSA-TA Cascade Implementation

Following the promising results from the preliminary screening, the transamination conditions for substrate **7a** were subsequently optimized with purified pQR2191 TA. Throughout these experiments, the buffer and reaction temperature were kept constant to ensure compatibility of conditions in the planned FSA-TA cascade. According to the literature, the pQR2191 TA displays good activity with isopropylamine (IPA) as the amine donor. The use of this substrate results in a higher atom economy and a shift of the reaction equilibrium toward the amine product, due to the formation of volatile acetone as a byproduct.^77−79^ A reaction conducted with low concentration of the catalyst and the amine donor IPA leads to a promising initial yield of 26% (**SI**Table S5). Increasing the concentration of either the enzyme or IPA has a strong effect on the conversion, with reactions completed with 2 mg·mL^–1^ of purified pQR2191 TA or 10 equiv of IPA. The reactions were monitored by ^1^H NMR, where a characteristic shift of the methyl signal of the substrate (2.34 ppm, 3H, s) to a 3H doublet at 1.31 ppm indicates that the less hindered methyl ketone was aminated as expected.

**Figure 3:**
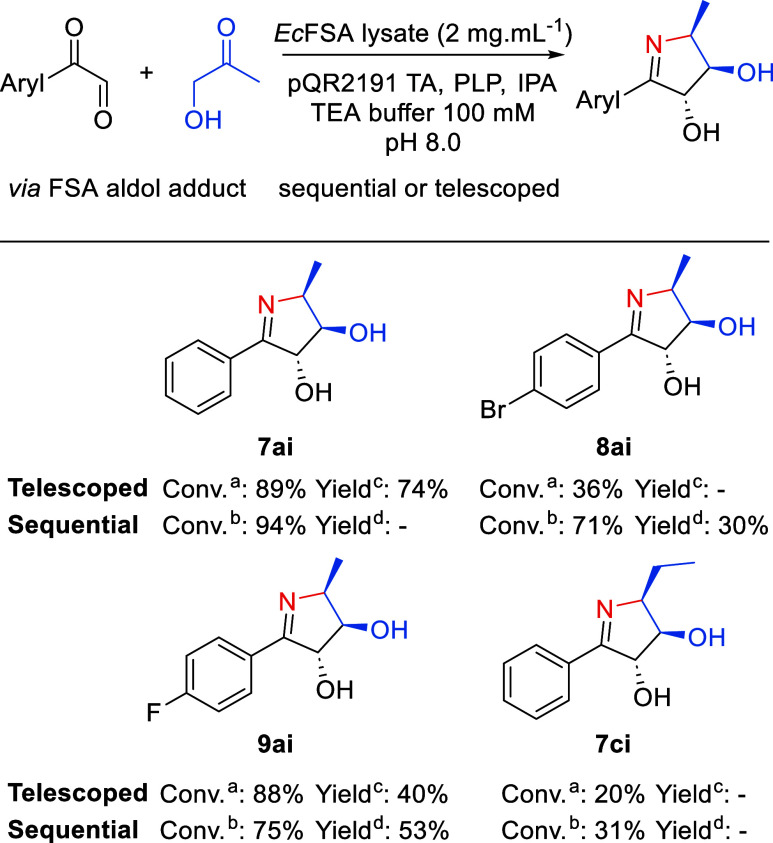
FSA-TA cascade implementation. (a) Conversion data for the telescoped cascade: 25 mM aldol adducts, 250 mM IPA, 1 mM PLP, *Ec*FSA_wt_ lysate 2 mg·mL^–1^, purified TA pQR2191 1 mg·mL^–1^ in 100 mM TEA buffer pH 8.0, 625 μL reaction volume, 200 rpm, 30 °C, 24 h. **(**b) Conversion data for the sequential cascade: (1) 25 mM aldol adducts, *Ec*FSA_wt_ lysate 2 mg·mL^–1^ in 100 mM TEA buffer pH 8.0, 500 μL reaction volume, 200 rpm, 30 °C, 24 h. (2) 250 mM IPA, 1 mM PLP, purified TA pQR2191 1 mg·mL^–1^ in 100 mM TEA buffer pH 8.0, 625 μL reaction volume, 200 rpm, 30 °C, 24 h. (c) Isolated yield from the telescoped cascade (25 mL scale, 0.5 mmol). (d) Isolated yield from the sequential cascade (25 mL scale, 0.5 mmol).

The FSA-TA cascade was subsequently investigated as either a telescoped or sequential one-pot process (Figure 3 and **SI**Table S6). In the telescoped cascade all reaction components are added simultaneously, whereas in the sequential mode the aldolase reaction is allowed to proceed for 24 h before addition of the transaminase reaction components. The conditions of the FSA-TA cascade were optimized by varying enzyme concentrations with a constant amount of IPA (**SI**Table S6). Very good conversions were achieved with the substrates phenylglyoxal **7** and hydroxyacetone **a** in both telescoped and sequential cascades. A telescoped reaction on 0.5 mmol scale gave an isolated yield of 74% of **7ai** (Figure 3). HRMS analysis of transaminase product **7ai** gave *m*/*z* = 192.1019, corresponding to the [M + H]^+^ of the cyclized 3,4-dihydro-2H-pyrrole (1-pyrroline) species **7ai**. ^13^C NMR revealed the disappearance of one carbonyl environment, as expected with conversion a ketone to an amine and a shift of the other signal in the carbonyl region, further supporting the formation of the cyclic imine product **7ai**. The presence of a single new doublet peak for the CH_3_ suggests that a single new diastereomer has been formed from the less hindered ketone in the substrate. The ^3^*J*_*H–H*_coupling constants along the C4–C5 and C3–C4 bonds and comparison with known compounds suggest that the protons on C4–C5 are *cis* to each other,^80^ and the protons on C3–C4 are *trans*, as expected from the aldolase derived stereochemistry for C3 & C4. Taken together, these data suggest the formation of the *S* stereochemistry at the new amine center in accordance with previous work with this transaminase.^75,76^

The reported synthetic route to this analogous 2-aryl-1-pyrroline iminosugar involves six synthetic steps and protection of all hydroxyl groups of the starting material, d-ribose. The key aryl moiety is introduced via an elegant tandem addition-cyclization of an aryl-Grignard reagent and the d-ribose derived methanesulfonylglycononitrile.^80^ Cyclic sugar imines have also been accessed previously via chemoenzymatic synthesis using DHAP-dependent aldolases and azidoaldehyde precursors as masked amine equivalents.^81^ In contrast to the two methods described above, the FSA-TA cascade described herein can furnish sterechemically complementary pyrrolines from diverse aryl glyoxals in a telescoped or sequential one-pot process without need of any protecting groups.

The scope of the cascade was extended to encompass 1-pyrroline products **8ai**, **9ai**, and **7ci** (Figure 3 and **SI**Table S6). Interestingly, the sequential cascade yielded better results for product **8ai** in comparison to a telescoped sequence. This difference might be explained by a substrate inhibition of the transaminase, a high activity toward one of the substrates or a mismatch in relative rates of the aldolase and transaminase catalysts in the telescoped sequence. Nevertheless, **8ai** was isolated in a 30% yield from the sequential cascade. Sequential and telescoped cascades gave good conversions and isolated yields for **6ai**. Unfortunately, low conversions were observed for the product **7ci** on analytical scale. In this case, an ethyl group has to be accommodated in the small pocket of the binding site of the transaminase, which is commonly reported to be a challenge for wild-type enzymes of this class.^82−87^ The screening or engineering of transaminases with expanded substrate scope toward these bulky substrates would further expand the synthetic potential of this approach.

### Reduction of Biocatalytically Synthesized Pyrrolines and Screening of Imine Reductases

**Figure 4:**
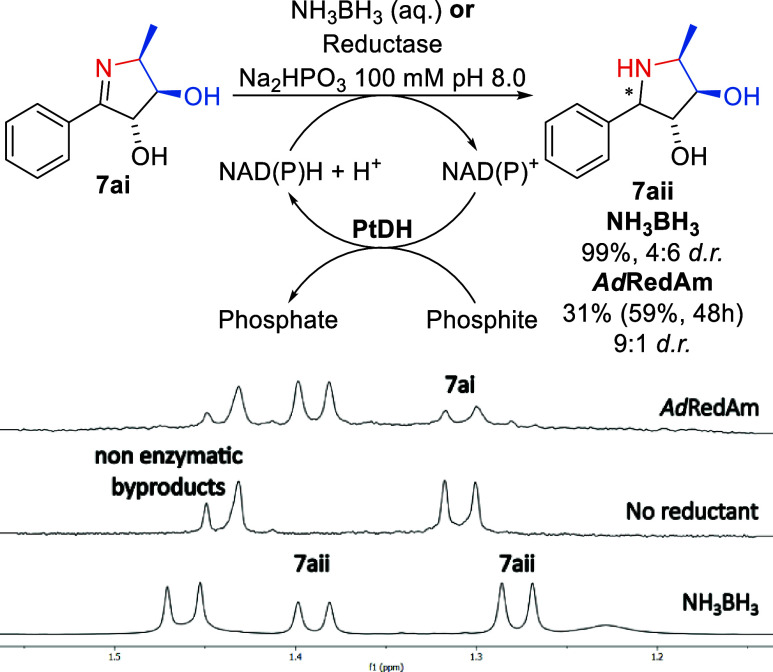
Chemical and enzymatic reductions of biocatalytically synthesized pyrroline **7ai** to form pyrrolidine **7aii** and characteristic CH_3_ signals in enzymatic, chemical and control reactions. Reaction conditions: 10 mM substrate **7ai** and 20 mM NH_3_BH_3_ or 1 mg·mL^–1^ purified *Ad*RedAm, 0.5 mM NADP^+^, 0.25 mg·mL^–1^ PtDH^90^ lysate in 100 mM NaPt buffer pH 8.0. Reactions were incubated at 30 °C, 200 rpm and a second portion of enzyme was added at 24 h. NMR yield is calculated as the percentage of both amine diastereomers relative to remaining substrate.

Polyhydroxlated substrates are known to be challenging for typical imine reductases due to the polarity of the enzyme active site architecture or potential interference of promiscuous alcohol dehydrogenase activity giving rise to false positive hits or undesirable byproducts.^47,48,58^ In the course of investigations toward diversification of the FSA derived diketones, a panel of 384 metagenomic IREDs was screened against diketone **9a** for reductive amination with cyclopropylamine. This screen returned only a handful of enzymes exhibiting trace product formation (<5% by UPLC-QDa, **SI**Figures S143 and S144), supporting the hypothesis that the densely functionalized structures explored in this work are challenging substrates for IREDs. As such, a targeted panel of previously characterized IREDs and RedAms were rationally selected based on related literature substrate scope and screened for activity against the 3,4-dihydro-2H-pyrrole (1-pyrroline) product **7ai** (Figure 4 and **SI**Figure S60).^47,48,58,63,88,89^

The chemical reduction of pyrroline **7ai** was achieved by treatment with NH_3_BH_3_ after an initial, unsuccessful attempt with NaCNBH_3_ left **7ai** unreacted. Accordingly, NH_3_BH_3_ mediated imine reduction provided the pyrrolidine product **7aii** in 99% yield (as measured by ^1^H NMR) as a mixture of two diastereomers (6:4 *d.r.*, Figure 4). The identified reductases were first screened against **7ai** using QDa mass spectrometry, whereby three enzymes, AniN6, *Nh*IRED and *Ad*RedAm, appeared to give rise to a new species with *m*/*z* 194, corresponding to the pyrrolidine product **7aii** (Figure S61).

Reductase biotransformations were subsequently monitored by ^1^H NMR which showed that *Ad*RedAm provided 31% NMR yield after 24 h, and 59% after 48 h and a second enzyme addition, and *Nh*IREDafforded 30% of amine product after 48 h and two enzyme additions. Interestingly, the major diastereomer formed with both enzymes corresponds to the minor diastereomer formed upon chemical reduction. No conversion was observed in biotransformations with AniN6 by ^1^H NMR. Attempts to increase product formation by increasing enzyme loading and reaction time to 2.5 mg·mL^–1^ and 48–72 h were unsuccessful. The reaction with *Ad*RedAm conducted at a larger scale (5 mL) was significantly slower than at analytical (0.5 mL) scale, as seen in previous work with this enzyme on challenging substrates.^63^ Similarly, attempts to incorporate *Ad*RedAm into the FSA-TA cascade established above were not fruitful, with only 9–12% conversion to pyrrolidine seen by ^1^H NMR (Table S9).

Previous synthetic methods toward comparable compounds in this class of 2-aryl pyrrolidine iminosugars generally involve long synthetic sequences, multiple protection strategies and reliance upon chiral pool reagents.^8,52,91−93^ For example, El-Nezhawy et al. developed an eight step synthesis of diverse codonopsine analogs from d-tartaric acid,^8^ the group of Behr have demonstrated the synthesis of 2-aryl pyrroline and pyrrolidine iminosugars from d-ribose in seven to 11 steps using Grignard reagents to introduce the key aryl moiety.^52,80^ Similarly, Toyao et al. used a key Grignard addition to furnish (−)-codonopsinine from l-lyxose via a sugar derived nitrone in 11 steps.^91^ A more concise synthesis of (−)-codonopsinine was described by Reddy et al.,^93^ where six synthetic steps from d-alanine furnish the pyrrolidine scaffold via asymmetric Sharpless dihydroxylation and acid catalyzed amidocyclisation.

In comparison, at present our methodology provides one-pot access to 2-aryl pyrroline iminosugars without need of any protecting groups. Preliminary findings indicate that reductive aminases or a chemical reduction are capable of furnishing the 2-aryl pyrroline scaffold in a further synthetic step albeit with imperfect stereocontrol. Despite the inability to perform preparative synthesis of the pyrrolidine iminosugars using these wild-type reductases, the stereoselectivity (*d.r.* 9:1) observed in biotransformations with *Ad*RedAm is promising. It is anticipated that protein engineering or directed evolution of *Ad*RedAm could provide a more synthetically utile biocatalyst capable of reducing these challenging substrates but such efforts are outside the scope of this work.

## Conclusions

The enzymatic synthesis of chiral 2,3-dihydroxy-1,4-dicarbonyls, which are versatile synthons for the synthesis of highly functionalized (hetero)cyclic scaffolds from simple, achiral precursors is described. d-Fructose-6-phosphate aldolase from *Escherichia coli* (*Ec*FSA) is shown to catalyze the synthesis of a diverse library of arylated-1,4-diketones and a dihydroxy-1,4-keto acid with remarkable efficiency. Preparative reactions provide good product yields and XRD analysis identified the 2*S*,3*S* enantiomer, which is consistent with FSA stereochemical outcome reported in the literature. Furthermore, the downstream synthetic potential of these compounds is exemplified. The identification and implementation of an (*S*)-selective transaminase enables the cascade synthesis of chiral 2-aryl-1-pyrroline iminosugars with three new stereocenters in a regio- and stereoselective manner. Finally, preliminary findings suggest that the fungal reductive aminase *Ad*RedAm is a promising candidate for protein engineering or directed evolution of a stereoselective iminosugar imine reductase. By identifying and engineering stereocomplementary enzymes, we anticipate that this procedure will provide a platform for exploring the structure–activity relationships of highly functionalized hydroxylated heterocycles and developing new pharmaceutically relevant fragments.

## Supporting Information Available

Experimental details including: materials and methods, biotransformation procedures, protein sequences, expression and purification procedures, full biotransformation UPLC and NMR analysis and optimization data, UPLC-QDa methods and traces, synthetic procedures and NMR characterization data (PDF)
