# Supplementary material for: Stereoselective Chemoenzymatic Cascades for the Synthesis of Densely Functionalized Iminosugars
Source: J Am Chem Soc. 2025 Feb 10;147(7):6067–75. doi: 10.1021/jacs.4c16732 (PMC11848910; doi:10.1021/jacs.4c16732)
Supplement: Supplementary file 1 — ja4c16732_si_001.pdf [file ja4c16732_si_001.pdf]

Supporting information for

# Stereoselective chemo-enzymatic cascades for the synthesis of densely functionalized iminosugars.

Christopher R. B. Swanson¶a, Léa Gourbeyre¶a, Grayson J. Ford[a], Pere Clapés [b] and Sabine L. Flitsch [a]*

¶ These authors contributed equally

[a] Manchester Institute of Biotechnology, School of Chemistry, The University of Manchester, 131 Princess Street, M1 7DN, Manchester, United Kingdom. [b] Biological Chemistry Department, Institute for Advanced Chemistry of Catalonia, IQAC−CSIC, 08034 Barcelona, Spain.

*Email: Sabine.flitsch@manchester.ac.uk

## Contents

Materials and methods ..... 2
Analytical methods ..... 3
Biocatalyst Production ..... 4
  Protein production ..... 4
  Protein purification ..... 5
  SDS-PAGE analysis ..... 5
  Protein sequences ..... 5
Biotransformation Methods ..... 9
  Analytical scale aldol additions catalyzed by FSA variants. ..... 9
Substrate scope of the FSA catalyzed aldol addition ..... 10
  Time course experiments ..... 13
  Preparative scale aldol additions catalyzed by FSA variants ..... 16
Chiral analysis of aldolase products ..... 31
  Comparison with chemically synthesized aldol products by chiral HPLC ..... 31
  Crystallisation and XRD analysis ..... 35
Transaminase biotransformations ..... 37
  Screening of TA for activity towards FSA aldol products ..... 37
  Optimization of the transamination step ..... 38
  Implementation of the telescoped FSA – TA cascade ..... 39
  Implementation of the sequential FSA – TA cascade ..... 40
  Scale up and structural characterisation of transaminase products ..... 46
Reduction of transaminase derived pyrrolines ..... 55
  Chemical reduction of Ph,Me substituted pyrroline **7ai** ..... 55
  Screening of reductases for IRED activity against transaminase derived pyrroline **7ai** ..... 58
  Comparison of selectivity in enzyme catalyzed and chemical imine reduction ..... 62
  Attempts to scale-up the enzymatic reduction ..... 63

Attempts to assemble the FSA-TA-IRED cascade ........ 64
Glycosidase activity assays ........ 69
UPLC-QDa chromatograms of FSA biotransformations ........ 71
Calibration curves ........ 71
Zero-hour biotransformations ........ 75
Example traces of biotransformations at 24h ........ 81
NMR spectra used to determine conversion in FSA biotransformations of **22** and **23** ........ 96
Glyoxylic acid **22** ........ 96
Trifluoropyruvaldehyde **23** ........ 99
Investigations into alternate enzymatic transformations of aldol adducts ........ 102
Screening of metagenomic imine reductases ........ 102
References ........ 105

## *Materials and methods*

All commercial reagents and solvents used in this work were purchased from Sigma-Aldrich (Poole, Dorset,UK), Alfa-Aesar (Heysham, Lancashire, UK), Acros Organics (Loughborough, UK), or Fluorochem (Hadfield, Derbyshire, UK) and used without further purification. Isopropyl β-D1-thiogalactopyranoside (IPTG), kanamycin, Terrific broth (TB) and LB (Luria-Bertani) Agar Miller was purchased from Formedium (Hunstanton, England).

Unless stated otherwise, a Bruker Avance 400 MHz spectrometer was used to record NMR spectra with chemical shifts reported in ppm relative to tetramethylsilane (TMS) or the residual protic solvent signal. 800 MHz NMR spectra were recorded on a Bruker AVIII 800 MHz AVIII spectrophotometer. $D_2O$ or $CD_3OD$ were used as solvents for all NMR experiments. Coupling constants (J) are reported in Hz.

Reverse phase UPLC-QDa analysis was carried out on a Waters Acquity H-class system with sample manager (model J15SD1368G), column heater (model H15CHA), FLR detector (Model G15UPF045G), TUV detector (model E16TUV487A), QDa detector (model KAB1525). A HSS C18 1.8 µm, 2.1 x 100 mm column was used as stationary phase. Thompson UPLC grade nano filter vials with PVDF membrane, 0.45 µm pore size were used for all UPLC samples and standards.

Chiral HPLC was performed on an Agilent system (Santa Clara, CA, USA) equipped with a G1379A degasser, G1312A binary pump, a G1367A well plate autosampler unit, a G1316A temperature-controlled column compartment and a G1315C diode array detector. Daicel (Osaka, Japan) CHIRALPAK®IA, CHIRALPAK®IC and CHIRALPAK®IE analytical (all 250 mm length, 4.6 mm diameter, 5 µm particle size) columns were used. The typical injection volume was 10 µl and chromatograms were monitored at 254 nm, unless stated otherwise.

*Analytical methods*

All UV data were collected at 254 nm.

Table S1: RP-UPLC-QDa method 1 used in this work. Solvent A = $dH_2O$ + 0.1% DFA, solvent B = Acetonitrile + 0.1% DFA. This method was applied with a HSS C18 1.8 µm, 2.1 x 100 mm column as stationary phase.

| Time / min | Flow rate / mL $min^{-1}$ | % solvent A | % solvent B |
|---|---|---|---|
| 0.00 | 0.5 | 95 | 5 |
| 8.0 | 0.5 | 50 | 50 |
| 8.1 | 0.5 | 5 | 95 |
| 8.9 | 0.5 | 5 | 95 |
| 9.0 | 0.5 | 95 | 5 |
| 10.0 | 0.5 | 95 | 5 |

Table S2: RP-UPLC-QDa method 2 used in this work. Solvent A = $dH_2O$ + 0.1% DFA, solvent B = Acetonitrile + 0.1% DFA. This method was applied with a HSS C18 1.8 µm, 2.1 x 100 mm column as stationary phase.

| Time / min | Flow rate / mL $min^{-1}$ | % solvent A | % solvent B |
|---|---|---|---|
| 0.00 | 0.5 | 95 | 5 |
| 4.5 | 0.5 | 50 | 50 |
| 4.6 | 0.5 | 5 | 95 |
| 5.0 | 0.5 | 5 | 95 |
| 5.1 | 0.5 | 95 | 5 |
| 6.0 | 0.5 | 95 | 5 |

Table S3: RP-UPLC-QDa method 3 used in this work. Solvent A = $dH_2O$ + 0.1% DFA, solvent B = Acetonitrile + 0.1% DFA. This method was applied with a HSS C18 1.8 µm, 2.1 x 100 mm column as stationary phase.

| Time / min | Flow rate / mL $min^{-1}$ | % solvent A | % solvent B |
|---|---|---|---|
| 0.00 | 0.5 | 95 | 5 |
| 3.00 | 0.5 | 60 | 40 |
| 3.10 | 0.5 | 95 | 5 |
| 4.00 | 0.5 | 95 | 5 |

### *Biocatalyst Production*

Chemically competent E. coli cells were transformed with DNA plasmid vectors that contain genes for the desired protein (biocatalyst) and grown on LB agar plates containing 30 µg $mL^{-1}$ antibiotic (kanamycin) as shown in **Table S4**. Commercial enzymes ATA 113 and 256 from Codexis and IRED09, 23, 39 and 83 from Prozomix Ltd were also used.

Table S4**:** Biocatalysts and cell strains used in this work. BL21(DE3) cells were purchased from New England Biolabs.

| Protein | Organism | GenBank/ UniProt | Plasmid | Cell strain |
|---|---|---|---|---|
| *Ec*FSAwt | *E. coli* | P78055 | pET28a | BL21(DE3) |
| *Ec*FSA A129S | *E. coli* | - | pET28a | BL21(DE3) |
| pQR2191 TA | *Novosphingobium aromaticivorans* | MK121627 | pET28a | BL21(DE3) |
| *Cv*TA | *Chromobacterium violaceum* | Q7NWG4 | pET28a | BL21(DE3) |
| *He*WT | *Halomonas elongata* | E1V913 | pET28a | BL21(DE3) |
| *Pp*TA | *Pseudomonas putida* | Q88CJ8 | pET28a | BL21(DE3) |
| *Rh*TA | *Rhodobacter sphaeroides* | A4WWF7 | pET28a | BL21(DE3) |
| AniN1 | *Streptomyces hygrospinosus* | A0A1V0QSW6 | pET28a | BL21(DE3) |
| AniN5 | *Chromobacterium violaceum* | Q7NVT4 | pET28a | BL21(DE3) |
| AniN6 | *Pseudomonas protegens* | A0A2C9EKC9 | pET28a | BL21(DE3) |
| pRed-14 | *Paenibacillus sp.* | A0A089IG04 | pET28a | BL21(DE3) |
| pRed-15 | *Bacillus subtilis* | A0A0K9HAW1 | pET28a | BL21(DE3) |
| *Nh*IRED | Nocardiopsis halophila | A0A0J9X1X6 | pET28a | BL21(DE3) |
| *Ad*RedAm | *Ajellomyces dermatitidis* | A0A179UH34 | pET28a | BL21(DE3) |
| PtDH[1] | *Pseudomonas stutzeri* | - | pET28a | BL21(DE3) |

### *Protein production*

A single colony harbouring the desired gene was added to 20 mL LB medium supplemented with 20 µL kanamycin (30 mg.$mL^{-1}$) and incubated overnight at 250 rpm and 30 °C. The full volume of this preculture was used to inoculate 400 mL of TB media, supplemented with 400 µL of kanamycin (30 mg.$mL^{-1}$), in a 2 L baffled flask and incubated at 37 °C, 250 rpm until optical cell density (OD600) of 0.6 was reached. At this point, protein expression was induced by inoculating the flask with 400 µL of isopropyl-β-D-1-thiogalactopyranoside (IPTG, 0.1 M). The cultures were then incubated at 22 °C at 200 rpm for 20 h. The cells were harvested by centrifugation at 4000 rpm for 30 min at 4 °C. The cell pellets were washed with NaPi buffer (100 mM, pH 7.4) and centrifuged again using the same conditions and the supernatant discarded. The cell pellets were stored at -20 °C in 50 mL falcon tubes until further use. Cell free extracts were prepared as follows; the thawed cell pellets were resuspended in 4–5 mL (per g of cell pellet) in NaPi or TEA buffer (100 mM, pH 7.4–8.0) and lysed by ultra-sonication (60 sec ON, 120 sec OFF, 40 AMP, 4 cycles) while samples were submerged in an ice bath. The lysed cells were clarified by ultra-centrifugation at 20,000 rpm at 4 °C for 45 min. The clarified supernatant was filtered through a

cellulose membrane (0.45 μm), frozen at -80 ºC then lyophilized on a Buchi Lyovapor-200. Lyophilized cell free extract was stored at -20 ºC until further use.

### *Protein purification*

Thawed cell pellets were resuspended in 5 mL (per g of cell pellet) in 10 % Buffer B (100 mM NaPi buffer, 300 mM NaCl, 30 mM imidazole, pH 7.5) and lysed by ultra-sonication (60 sec ON, 120 sec OFF, 40 AMP, 4 cycles) while samples were submerged in an ice bath. The lysed cells were clarified by ultra-centrifugation at 18,000 rpm at 4 ºC for 60 min. The clarified supernatant was filtered through a cellulose membrane (0.45 μm) and loaded onto a His-Trap Crude FF column (GE Healthcare) charged with 0.1 M nickel sulphate equilibrated with 10% buffer B. The column was washed with 15 mL 10% buffer B and then 15 mL 20% buffer B. His-tagged protein was then eluted with 100% buffer B and 1–2 mL fractions were collected. The protein concentration of each fraction was monitored by a thermofisher NanoDrop™ microvolume spectrophotometer. Fractions containing pure protein were combined and then concentrated using a membrane 30,000 MWCO PES vivaspin columns to remove excess imidazole and exchanged into NaPi or TEA buffer until the desired concentration was achieved (typically 10 mg.mL$^{-1}$). The pure protein was divided into 1 mL aliquots, flash frozen and stored at -80 ºC until further use. Expression yields of proteins were up to 150 mg/L of culture broth.

### *SDS-PAGE analysis*

SDS PAGE was carried out on BioRad premade gel in 1X TGS running buffer. Samples were loaded with 50% Laemmli buffer purchased from Sigma Aldrich, Molecular weight marker was purchased from New England Biolabs.

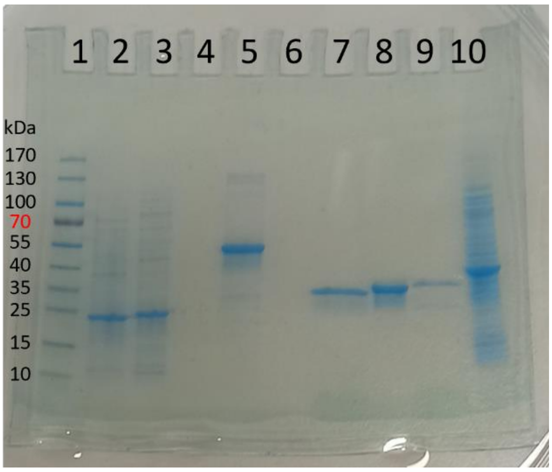


Figure S1: SDS-PAGE gel of key proteins used in this work. Left to right – PageRule ladder (1), *Ec*FSA$_{wt}$ lysate (2), *Ec*FSA$_{A129S}$ lysate (3), purified pQR2191 TA (5), purified AniN6 (7), purified *Nh*IRED (8), purified *Ad*RedAm (9), PtDH lysate (10).

### *Protein sequences*

#### *FSAwt*

sp|P78055|FSAA_ECOLI Fructose-6-phosphate aldolase 1 OS=Escherichia coli (strain K12) OX=83333 GN=fsaA PE=1 SV=2

MELYLDTSDVVAVKALSRIFPLAGVTTNPSIIAAGKKPLDVVLPQLHEAMGGQGRLFAQVMATTAEGM
VNDALKLRSIIADIVVKVPVTAEGLAAIKMLKAEGIPTLGTAVYGAAQGLLSALAGAEYVAPYVNRIDAQ
GGSGIQTVTDLHQLLKMHAPQAKVLAASFKTPRQALDCLLAGCESITLPLDVAQQMISYPAVDAAVAKF
EQDWQGAFGRTSI

*FSA A129S*

MELYLDTSDVVAVKALSRIFPLAGVTTNPSIIAAGKKPLDVVLPQLHEAMGGQGRLFAQVMATTAEGM
VNDALKLRSIIADIVVKVPVTAEGLAAIKMLKAEGIPTLGTAVYGAAQGLLSLAGAEYVSPYVNRIDAQG
GSGIQTVTDLHQLLKMHAPQAKVLAASFKTPRQALDCLLAGCESITLPLDVAQQMISYPAVDAAVAKFE
QDWQGAFGRTSI

*pQR2191 TA*

>MK121627

MSGQRDQELRARAAKVMPSSAFGHVGTALLPANYPQFFERAEGAYVWDADGNRYLDYMCAFGPNLL
GYRDPRVESAASAQAARGDVMTGPSPLAVELAEKFVEIVSHADWAFFCKNGTDATTIARTIARAQTGR
RKILIAEGSYHGAAPWCNPFPAGTVPEDRAHMLTFTFNDIASLEAAVAEAGDDLAGIIATPFKHEAFAN
QEFPTQDYARRCREICDASGAVLVVDDVRAGFRLAVDCSWATVGVKPDLSCWGKCFANGYSISAVMGS
NRVKQGADSIFATGSFWQSAISMAAALATLDIIRDGKVIEKTVRLGQRLRDGLDEVSRRHGFTLNQTGP
VQMPQILFEGDPDFRVGFAWTSAMIDRGFYLHPWHNMFLCDAMTEEDIDQTIEAADSAFATVRAALP
TLQPHERVLALFSARAE

*Chromobacterium violaceum TA (CvTA)*

tr|Q7NWG4|Q7NWG4_CHRVO Putative 8-amino-7-oxononanoate synthase OS=Chromobacterium violaceum (strain ATCC 12472 / DSM 30191 / JCM 1249 / NBRC 12614 / NCIMB 9131 / NCTC 9757) OX=243365 GN=CV_2025 PE=1 SV=1

MQKQRTTSQWRELDAAHHLHPFTDTASLNQAGARVMTRGEGVYLWDSEGNKIIDGMAGLWCVNVG
YGRKDFAEAARRQMEELPFYNTFFKTTHPAVVELSSLLAEVTPAGFDRVFYTNSGSESVDTMIRMVRR
YWDVQGKPEKKTLIGRWNGYHGSTIGGASLGGMKYMHEQGDLPIPGMAHIEQPWWYKHGKDMTPD
EFGVVAARWLEEKILEIGADKVAAFVGEPIQGAGGVIVPPATYWPEIERICRKYDVLLVADEVICGFGRT
GEWFGHQHFGFQPDLFTAAKGLSSGYLPIGAVFVGKRVAEGLIAGGDFNHGFTYSGHPVCAAVAHANV
AALRDEGIVQRVKDDIGPYMQKRWRETFSRFEHVDDVRGVGMVQAFTLVKNKAKRELFPDFGEIGTLC
RDIFFRNNLIMRACGD HIVSAPPLVMTRAEVDEMLAVAERCLEEFEQTLKARGLA

*Halomonas elongata TA (HeWT)*

>tr|E1V913|E1V913_HALED Putrescine aminotransferase OS=Halomonas elongata (strain ATCC 33173 / DSM 2581 / NBRC 15536 / NCIMB 2198 / 1H9) OX=768066 GN=spuC PE=1 SV=1

MQTQDYQALDRAHHLHPFTDFKALGEEGSRVVTHAEGVYIHDSEGNRILDGMAGLWCVNLGYGRREL
VEAATAQLEQLPYYNTFFKTTHPPAVRLAEKLCDLAPAHINRVFFTGSGSEANDTVLRMVRRYWALKG
QPDKQWIIGRENAYHGSTLAGMSLGGMAPMHAQGGPCVPGIAHIRQPYWFGEGRDMSPEAFGQTCAE
ALEEKILELGEEKVAAFIAEPVQGAGGAIMPPESYWPAVKKVLAKYDILLVADEVICGFGRLGEWFGSQH
YGLEPDLMPIAKGLSSGYLPIGGVLVGDRVAETLIEEGGEFFHGFTYSGHPTCAAVALKNLELLEAEGVV
DRVRDDLGPYLAERWASLVDHPIVGEARSLGLMGALELVADKTTGQRFDKSLGAGNLCRDLCFANGLV
MRSVGDTMIISPPLVIRREEIDELVELARRALDETARQLTQVPHTQEEPTA

*Pseudomonas putida TA (ppTA)*

>tr|Q88CJ8|Q88CJ8_PSEPK Polyamine:pyruvate transaminase OS=Pseudomonas putida (strain ATCC 47054 / DSM 6125 / CFBP 8728 / NCIMB 11950 / KT2440) OX=160488 GN=spuC-II PE=3 SV=1

MSVNNPQTREWQTLSGEHHLAPFSDYKQLKEKGPRIITKAQGVHLWDSEGHKILDGMAGLWCVAVGY
GREELVQAAEKQMRELPYYNLFFQTAHPPALELAKAITDVAPKGMTHVFFTGSGSEGNDTVLRMVRH
YWALKGKPHKQTIIGRINGYHGSTFAGACLGGMSGMHEQGGLPIPGIVHIPQPYWFGEGGDMTPDEFG
VWAAEQLEKKILEVGEDNVAAFIAEPIQGAGGVIIPPETYWPKVKEILARYDILFVADEVICGFGRTGEW
FGSDYYDLKPDLMTIAKGLTSGYIPMGGVIVRDTVAKVISEGGDFNHGFTYSGHPVAAAVGLENLRILRD
EKIVEKARTEAAPYLQKRLRELQDHPLVGEVRGLGMLGAIELVKDKATRSRYEGKGVGMICRTFCFENG
LIMRAVGDTMIIAPPLVISHAEIDELVEKARKCLDLTLEAIQ

*Rhodobacter sphaeroides TA (RhTA)*

>tr|A4WWF7|A4WWF7_CERS5 Aminotransferase OS=Cereibacter sphaeroides (strain ATCC 17025 / ATH 2.4.3) OX=349102 GN=Rsph17025_2835 PE=3 SV=1

MALNDAAKAVGAVGAAMRDHVLLPAQEMAKLGKAAQPVLTHAEGIYVYVEDGRRLIDGPAGMWCAQ
VGYGRREIVDAMAHQAMVLPYASPWYMASSPAARLAQKIATLTPGDLNRIFFTTGGSTAVDSALRFSEF
YNNVLGRPQKKRIIVRYDGYHGSTALTAACTGRTGNWPNFDIAQDRISFLSSPNPRHAGNRSQEAFLDD
LVQEFEDRIESLGPDTIAAFLAEPILASGGVIIPPKGYHARFKAICEKHDILYISDEVVTGFGRCGEWFASE
KVFGVVPDIITFAKGVTSGYVPLGGLAISEAVLARISGENARGSWFTNGYTYSNQPVACAAALANIELME
REGLVDQAREMADYFAAALASLRDLPGVAETRSVGLVGCVQCLLDPTRADGTAEDKAFTLKIDERCFEL
GLIVRPLGDLCVISPPLIISRAQIDDMVAIMRQAITEVGAAHGLTAKEPAAV

*AniN1*

>tr|AniN_A0A1V0QSW6|A0A1V0QSW6_9ACTN Short-chain dehydrogenase OS=Streptomyces hygrospinosus OX=516360 GN=aniN PE=4 SV=1

MDFATGFADKVALVVGGGRGIGSAVVEELARRGARVVVADTDTLPSQYNHYQSTQVSGYADAQKLAAR
LTEEGLQVTAAQADATDEDQVSRLYADLAEQAGRLDVVVNAFGVTHVCPVERMELAEFQRVVSGNLD
GVFLSSKHAVPLLRDSGGGAIINFSSVSGRSGFAKVAHYCAGKFGVVGFTAALAQEVARDGIRVNAVCPG
IVRSNMWRYLLSEFVRPGETEDECWERMRSMIPQREFQTPKDLAELVVYLAGATKVTGQAISVDGGMT
AP

*AniN5*

>tr|AniN5_Q7NVT4|Q7NVT4_CHRVO Probable short-chain dehydrogenase OS=Chromobacterium violaceum (strain ATCC 12472 / DSM 30191 / JCM 1249 / NBRC 12614 / NCIMB 9131 / NCTC 9757) OX=243365 GN=CV_2258 PE=4 SV=1

MTQENTKHGKLAGKTMIVVGGARGIGAAIVEALARQEVKVSIFDTDRAPTATNHYQSRDISGYQAACQL
AEKLAAQGLSVQAMAVDATAESQVVEAVDLVAHAAEDFYGLVNAIGSSHVANTVDSSLSEFEAILQTNL
TAPYLTSREAARILVRQGRGGAILNISSIAAKLAFPGISAYCAAKSGLQGFSGALALELAPHNVRVNCVCP
GIVKTNMWKYLENQLMGPGENLEQLWARMEGLIPLGRTQTAANIARFCVAILENEDITAQSLSVDGGM
NLYG

*AniN6*

>tr|AniN6_A0A2C9EKC9|A0A2C9EKC9_PSEPH 3-oxoacyl-[acyl-carrier-protein] reductase FabG OS=Pseudomonas protegens (strain DSM 19095 / LMG 27888 / CHA0) OX=1124983 GN=fabG7 PE=4 SV=1

MTQGNKAQGKLSGKTMIVVGGARGIGSAIVEVLAKEGVHVYIFDTDRTPNAINHYQTQDVSGYQAARTL
AADLVEQGLSVKAIAVDATSEAQVIEAVAGVVHESEHFYGLVNAIGSSHLINTVESSLSEFDAIVQTNLTA
PYLTSREAARALIQRGKGGAILNISSIAAKVAFPGISSYCAAKAGLQGFSGALALELATHNIRVNCVCPGIV
KTNMWKYLENRLIEPNESLDDLWARMEGLIPLGRTQIPENIARFCLAVLENEDITAQSLSVDGGMNLY
G

*pRed-14*

>tr|pRed-14_A0A089IG04m|A0A089IG04_9BACL 6-phosphogluconate dehydrogenase OS=Paenibacillus sp. FSL H7-0737 OX=1536775 GN=H70737_05320 PE=4 SV=1

MKISFIGLGKMGFPMAQNLLKAGNELIVFNRTREKAQPLIDQGAHYAETPLEAAQKSDMVITMLSDDA
ALEEIVEGPNGILNGLSEKGIHISASTISVDLARKLSAAHAERKQYFVSATVLGRPDAAKAAKLRIILAGPE
QARRQLIPVLEQLGQEIFEIGDYSEAGNVVKIGVNFLIASMLEALSETQLMVEKYGIEPARFMDVVNALF
QSPLYQNYGAIMTEQRFEPAGFKMKLGLKDVALAIEAAQSVQAPLPLGQLIHHHLSEGIAHGYGEMDW
TALIRCLEHSS

*pRed-15*

>tr|pRed-15_A0A0K9HAW1|A0A0K9HAW1_9BACI Oxidoreductase OS=Bacillus sp. FJAT-27231 OX=1679168 GN=AC623_05230 PE=4 SV=1

MKIGFIGTGVMGKSMAGHLQKAGHQLFLYTRTKEKAEELLNKGAVWCKTPAEVARQTEVVFTIVGEPH
DVEEVYLGAEGILAGSSEGQMVIDMTTSQPSLARNIYEKAREKGVEALDAPVSGGDTGAKNGTLAIMVG
GSKAAFEKALPLFETIGSNIVYQGEAGAGQHTKMSNQIAIATNMIGVCEALLYAQKAGLDLDNVLKSIST
GAAGSWSLSNLAPRMIQGDFAPGFYVKHFIKDMKIAIEEAEKMGLELPGLSLAKRMYEELAARGEEKSG
TQALFNYWN

*NhIRED*

>tr|A0A0J9X1X6|A0A0J9X1X6_9ACTN Imine reductase OS=Nocardiopsis halophila OX=141692 PE=1 SV=1

MTNTKAKKSPVTLIGLGPMGQAMAGALLEAGYELTVWNRTKAKAEALAERGAAVADSPAEALRAGGP
VLLSLTEHAVMYRVLEGAESDLKGRTILNLGSDTPAASRAAAAWAEGHGARYVTGGVMSPAPGIGSSSV
FSFYSGDRAAFEENKALLEVLTATDFRGEDPGLAQVYYQILLDLFWTTMTGYLHALAVARAEGVPVGTI
TPYLIEGNDMAMFFEGTSAAVAEGRFPGDEDRISMDAASMEHVVQTSRDAGVDTALPEAVLSLFRRGL
DAGFAESSFARLVTLMDAEG

*AdRedAm*

>tr|A0A179UH34|A0A179UH34_BLAGS Oxidoreductase OS=Ajellomyces dermatitidis (strain SLH14081) OX=559298 GN=BDBG_03427 PE=1 SV=1

LFQGPAMANSPVSVFGLGAMGTALATQFLRKGHKTTVWNRTPAKAQPLIAIGASHAPTIDSAAAASSLL
IICQLDKASVMQTLQQAPTAWAAKTIVDLTNGTPAHARETADWALAHGARYIHGGIMAVPFMIGQPD
AMILYSGPAEVFEGVKDTLSVLGTNTYVGEDVGLASLHDLALLSGMYGLFSGFTHAVALVQSANIPAAG
FVATQLIPWLTAMTQHLNLLATQVDEKDYGDGGSSLDMQAKAAPNILEASQAQGVSVELIQPIFKLIER
RVEEGKGSEGLAALVGMIMK

*PtDH*

MLPKLVITHRVHDEILQLLAPHCELMTNQTDSTLTREEILRRCRDAQAMMAFMPDRVDADFLQACPEL
RVIGCALKGFDNFDVDACTARGVWLTFVPDLLTVPTAELAIGLAVGLGRHLRAADAFVRSGKFRGWQP
RFYGTGLDNATVGFLGMGAIGLAMADRLQGWGATLQYHEAKALDTQTEQRLGLRQVACSELFASSDFI
LLALPLNADTLHLVNAELLALVRPGALLVNPCRGSVVDEAAVLAALERGQLGGYAADVFEMEDWARA
DRPQQIDPALLAHPNTLFTPHIGSAVRAVRLEIERCAAQNLIQALAGERPINAVNRLPKAEPAAC

## *Biotransformation Methods*

### *Analytical scale aldol additions catalyzed by FSA variants.*

Reactions were carried out in flat bottomed 96-well plates with a total reaction volume of 250 µL or 2 mL Eppendorf tubes with a total reaction volume of 500 µL. Each reaction contained components diluted from stock solutions, with final concentrations of 25-100 mM ketoaldehyde substrate, 25-100 mM donor substrate (hydroxyacetone, dihydroxyacetone or 1-hydroxybutan-2-one, 1 equiv.) and 2 mg.mL$^{-1}$ lyophilized FSA cell free extract in 100 mM triethanolamine (TEA) buffer pH 8.0 containing 10-20 v% DMSO depending on substrate solubility. Reactions were incubated at 30 $^{o}$C, 200 rpm for up to 24 h and followed by UPLC-QDa analysis. Analytical samples were prepared by diluting an aliquot of biotransformation into 50 mM HCl in MeOH:water (9:1) to a theoretical substrate concentration of 1 mM (i.e. biotransformations at 100 mM substrate concentration were diluted 100x). The samples were centrifuged (10,000 rpm, 10 mins) and decanted into filter vials for UPLC-QDa analysis. Biotransformations with substrates **22** and **23** were monitored by $^{1}$H NMR after diluting an aliquot (250 µL) of the reaction mixture with $D_2O$ (250 µL).

## *Substrate scope of the FSA catalyzed aldol addition*

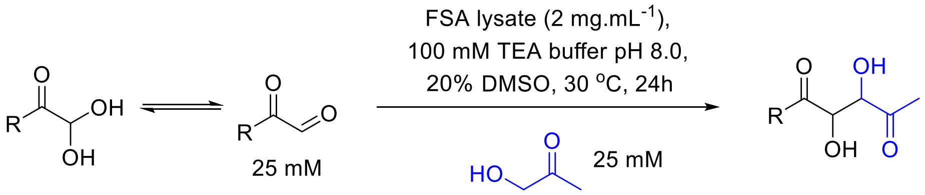


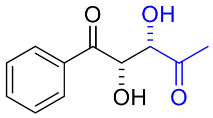

**7a**
WT: 91%
A129S: 84%

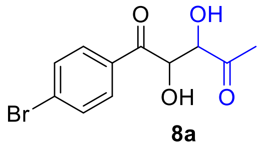

**8a**
WT: 99%
A129S: 94%

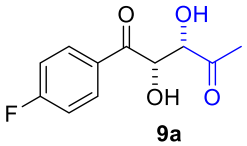

**9a**
WT: 92%
A129S: 86%

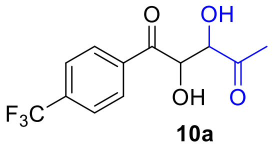

**10a**
WT: 67%
A129S: 49%

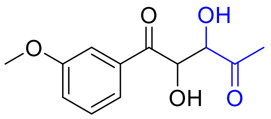

**11a**
WT: 94%
A129S: 92%

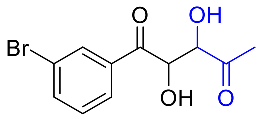

**12a**
WT: 99%
A129S: 97%

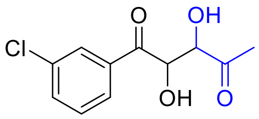

**13a**
WT: 97%
A129S: 98%

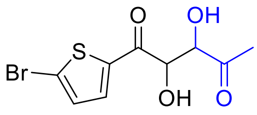

**14a**
WT: 98%
A129S: 90%

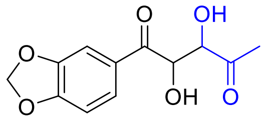

**15a**
WT: 87%
A129S: 95%

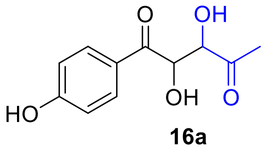

**16a**
WT: 52%
A129S: 74%

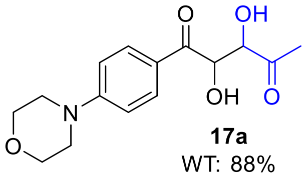

**17a**
WT: 88%
A129S: 84%

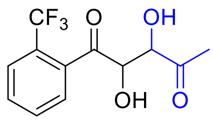

**18a**
WT: 85%
A129S: 82%

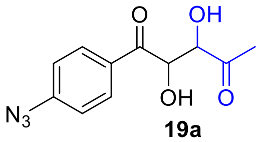

**19a**
WT: 84%
A129S: 77%

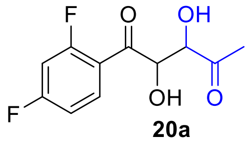

**20a**
WT: 83%
A129S: 84%

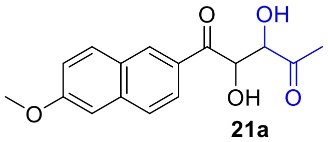

**21a**
WT: >99%
A129S: >99%

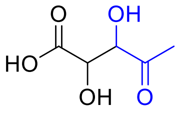

**22a**
WT: 78%
A129S: 75%

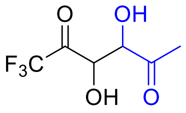

**23a**
WT: 47%
A129S: 47%

Figure S2: Conversion data (substrate depletion) for FSA catalyzed aldol addition of HA **a** into a panel of glyoxals. Conversion determined by UPLC-QDa.

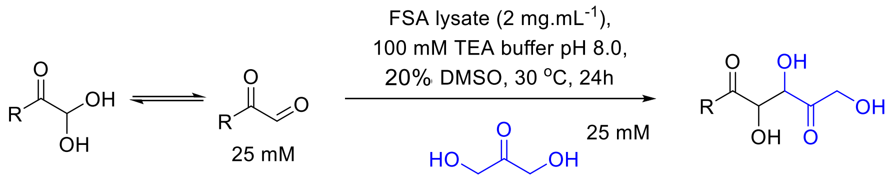


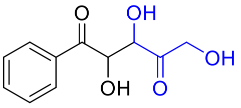

**7b**
WT: 17%
A129S: 75%

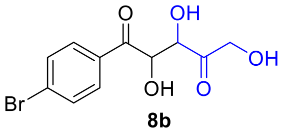

**8b**
WT: 36%
A129S: 83%

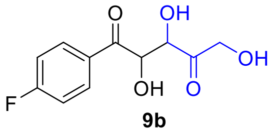

**9b**
WT: 19%
A129S: 73%

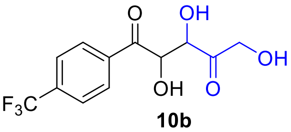

**10b**
WT: 10%
A129S: 39%

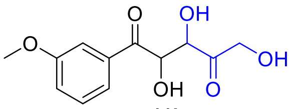

**11b**
WT: 29%
A129S: 85%

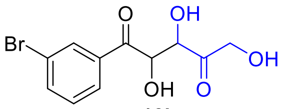

**12b**
WT: 36%
A129S: 94%

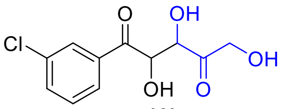

**13b**
WT: 57%
A129S: 96%

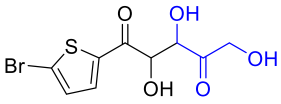

**14b**
WT: 85%
A129S: 90%

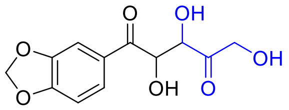

**15b**
WT: 29%
A129S: 87%

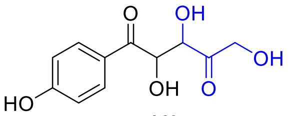

**16b**
WT: n.d.
A129S: n.d.

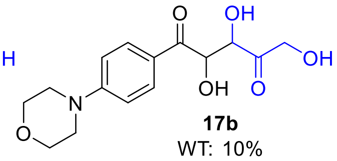

**17b**
WT: 10%
A129S: 27%

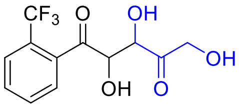

**18b**
WT: 63%
A129S: 95%

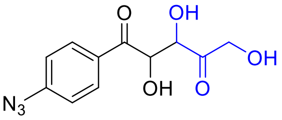

**19b**
WT: 67%
A129S: 76%

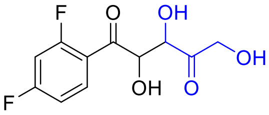

**20b**
WT: 16%
A129S: 15%

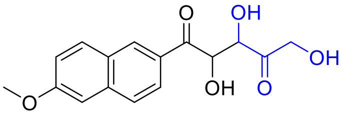

**21b**
WT: 85%
A129S: 95%

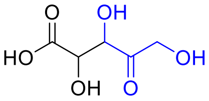

**22b**
WT: 81%
A129S: 85%

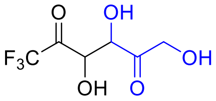

**23b**
WT: 9%
A129S: 76%

Figure S3: Conversion data for FSA catalyzed aldol addition of DHA **b** into a panel of glyoxals. Conversion determined by UPLC-QDa.

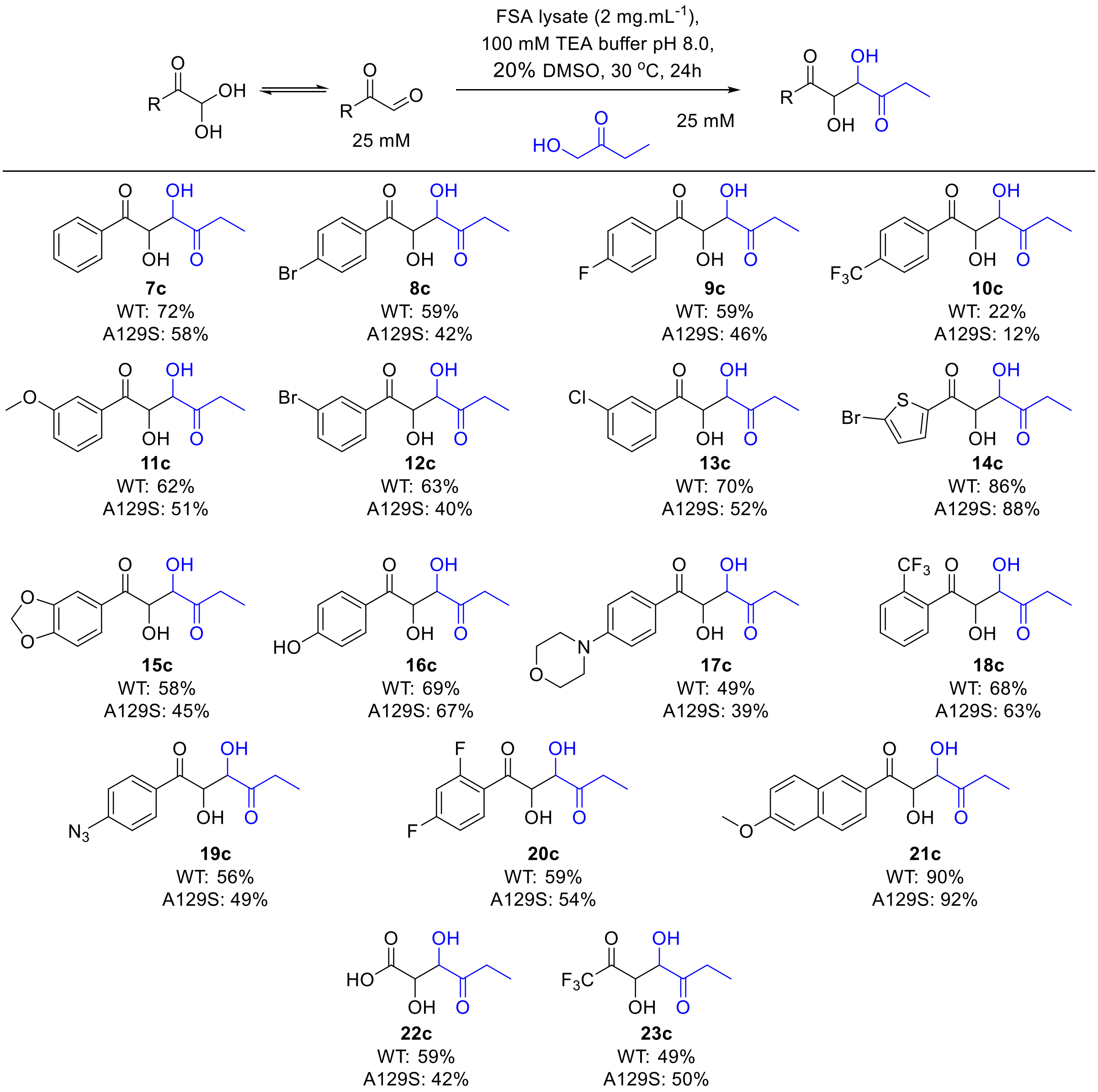


Figure S4: Conversion data for FSA catalyzed aldol addition of HB **c** into a panel of glyoxals. Conversion determined by UPLC-QDa.

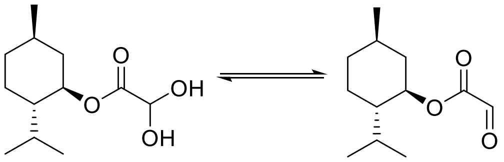

Figure S5: An additional substrate (1*R*)-(−)-menthyl glyoxylate monohydrate which was not accepted by the FSA variants tested in this work. $^{1}$H NMR analysis of biotransformations with this substrate returned only substrate signals after up to 72h.

### *Time course experiments*

Reactions were carried out in 2 mL Eppendorf tubes with a total reaction volume of 500 µL. Each reaction contained components diluted from stock solutions, with final concentrations of 25–100 mM ketoaldehyde substrate, 25–100 mM donor substrate (hydroxyacetone, 1 equiv.) and 2 mg.mL$^{-1}$ lyophilized $FSA_{wt}$ cell free extract in 100 mM triethanolamine (TEA) buffer pH 8.0 containing 10-20 v% DMSO depending on substrate solubility. Reactions were incubated at 30 $^{o}$C, 200 rpm and followed by UPLC-QDa or NMR analysis. Analytical samples (UPLC-QDa) were prepared by diluting an aliquot of biotransformation into 50 mM HCl in MeOH:water (9:1) to a theoretical substrate concentration of 1 mM (i.e. biotransformations at 100 mM substrate concentration were diluted 100x). The samples were centrifuged (10,000 rpm, 10 mins) and decanted into filter vials for UPLC-QDa analysis. Samples for NMR analysis were prepared by adding $D_2O$ (50 µL) to 500 µL of biotransformation mixture followed by centrifugation (10,000 rpm, 10 mins) and decanted into NMR tubes for $^{1}H$ or $^{19}F$ NMR analysis.

#### *4-bromophenyl glyoxal*

25 mM substrate concentration with 20% DMSO. Analyzed by UPLC-QDa.

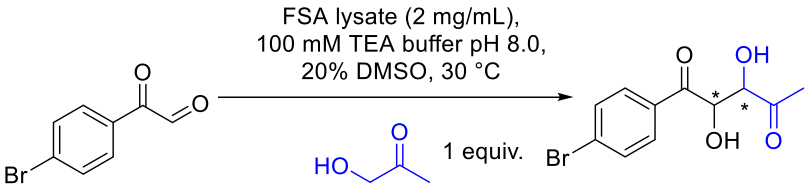


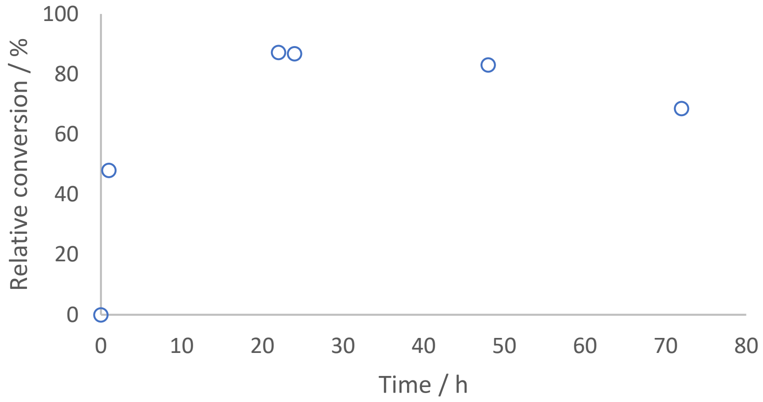


Figure S6: Time course data for reaction of 4-bromophenyl glyoxal **8** with hydroxyacetone (**a**) and $FSA_{wt}$. Conversion reaches peak at 22–24h then diminishes, suggesting product degradation.

*5-bromothienyl glyoxal*

50 mM substrate concentration with 20% DMSO. Analyzed by UPLC-QDa.

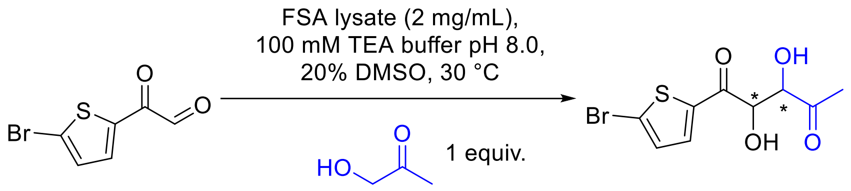


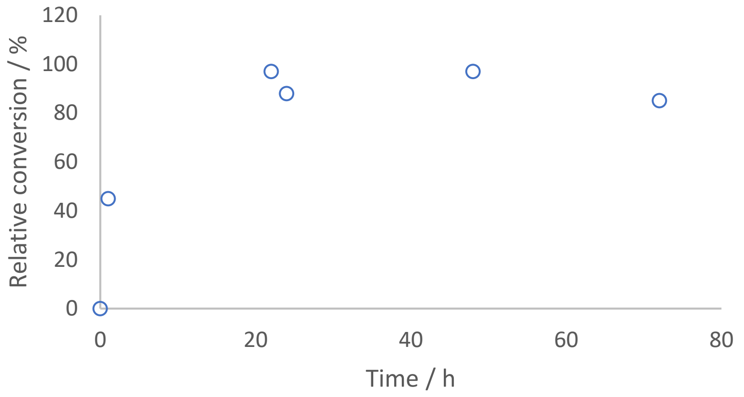


Figure S7: Time course data for reaction of 5-bromothienyl glyoxal **14** with hydroxyacetone **a** and $FSA_{wt}$. Conversion reaches peak at 22h.

*Glyoxylic acid*

100 mM substrate concentration with no DMSO. Analyzed by $^{1}H$ NMR.

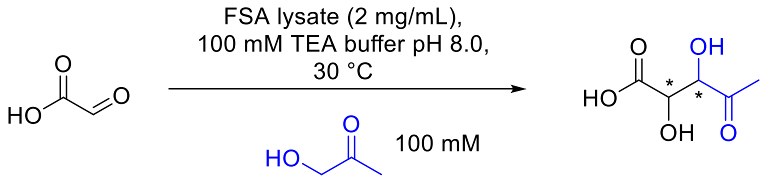


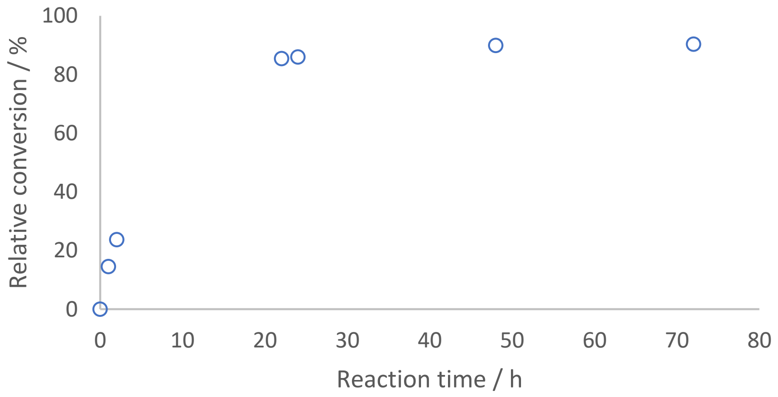


Figure S8: Time course data for reaction of glyoxylic acid **22** with hydroxyacetone **a** and $FSA_{wt}$. Conversion reaches peak at c. 24h.

*Trifluoropyruvaldehyde*

100 mM substrate concentration with 10% DMSO. Analyzed by [19]F NMR.

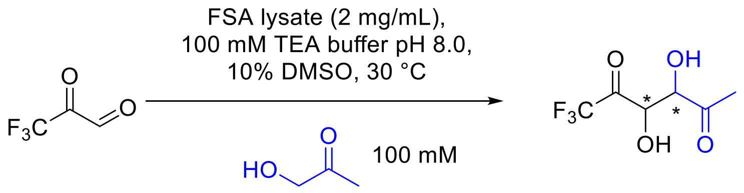


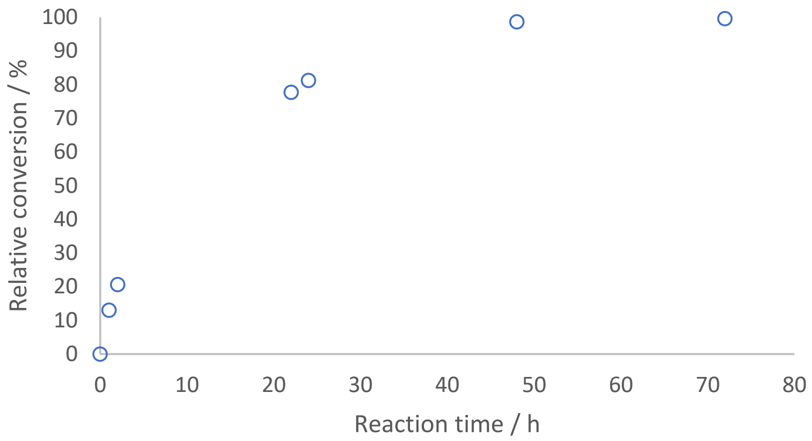


Figure S9: Time course data for reaction of trifluoropyruvaldehyde **23** with hydroxyacetone **a** and $FSA_{wt}$. Conversion reaches peak at 48h.

### *Preparative scale aldol additions catalyzed by FSA variants.*

Reactions were carried out in 50 mL falcon tubes or 250 mL conical flasks with total reaction volumes between 10 and 100 mL. Each reaction contained components diluted from stock solutions, with final concentrations of 25-100 mM ketoaldehyde substrate, 25-100 mM donor substrate (hydroxyacetone, 1 equiv.) and 2 $mg.mL^{-1}$ lyophilized FSA cell free extract in 100 mM triethanolamine (TEA) buffer pH 8.0 containing 10-20 v% DMSO depending on substrate solubility. Reactions were incubated at 30 $^{o}$C, 200 rpm for up to 24 h and followed by UPLC-QDa analysis. Analytical samples were prepared as above. Upon reaction completion the biotransformation was extracted with EtOAc (1 volume equiv. x 3). The organics were dried over $MgSO_4$, filtered, and concentrated in vacuo. If required, the product was further purified by column chromatography (ethyl acetate/ cyclohexane gradient 20–100 %).

#### *(2S,3S)-2,3-dihydroxy-1-phenylpentane-1,4-dione **7a***

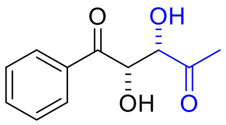


Synthesized using the general procedure above at 100 mM substrate concentration with 20% DMSO. Total reaction volume 100 mL, 10 mmol scale. Isolated yield 1.15 g (55%) of a white solid. Stereochemistry determined as *2S,3S* by XRD. δH (400 MHz, $CD_3OD$) 8.02 (d, *J* = 7.9 Hz, 2H), 7.64 (t, *J* = 7.5 Hz, 1H), 7.54 (dd, *J* = 7.9, 7.5 Hz, 2H), 5.59 (d, *J* = 2.2 Hz, 1H), 4.48 (d, *J* = 2.2 Hz, 1H), 2.34 (s, 3H). δC (101 MHz, $CD_3OD$) 210.49 (**C**O), 199.91 (**C**O), 135.84 (Ar), 134.72 (Ar), 129.93 (Ar), 129.56 (Ar), 79.67 (**C**HOH), 76.06 (**C**HOH), 26.67 (**C**$H_3$). HRMS(ESI) *m/z* calculated for $C_{11}H_{12}O_4$ $[M+Na]^+$ 231.0628, observed 231.0484 $[M+Na]^+$.

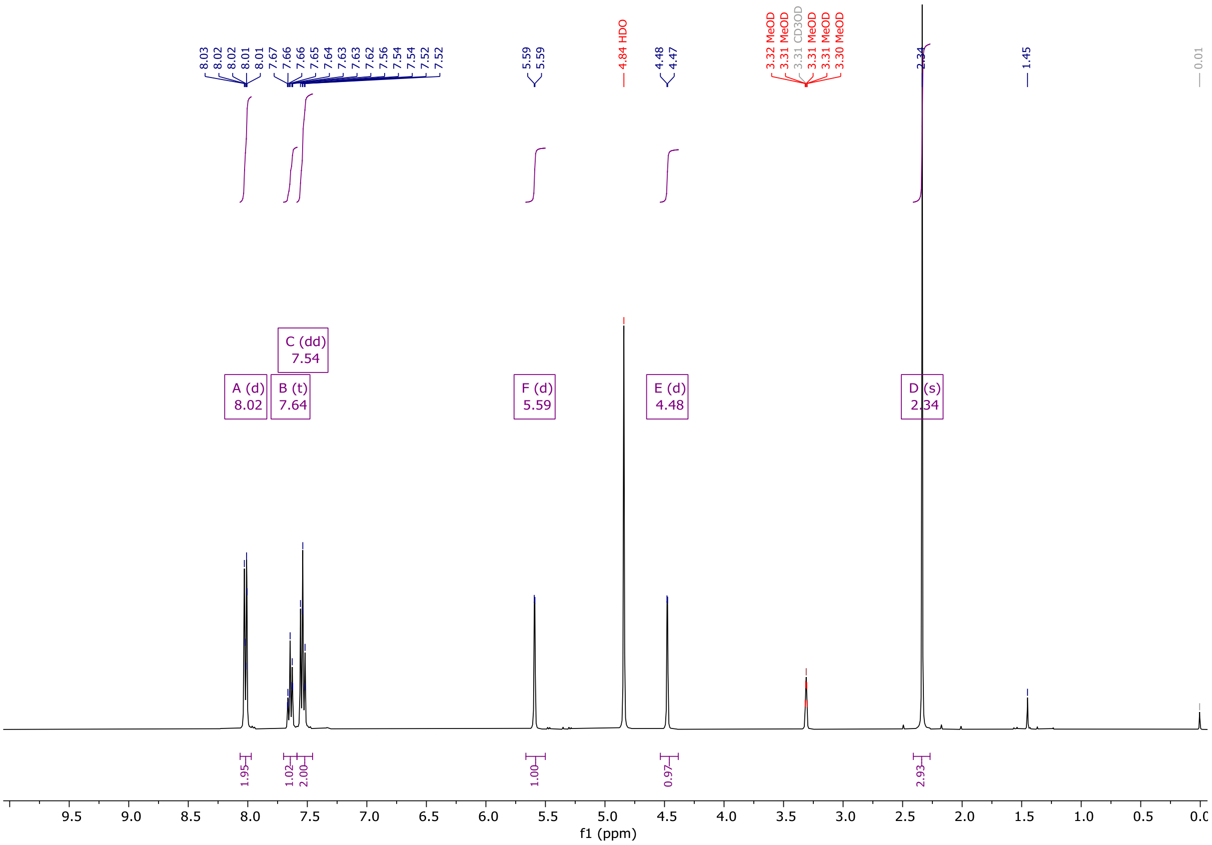


Figure S10: $^{1}$H NMR of biocatalytically synthesized **7a**.

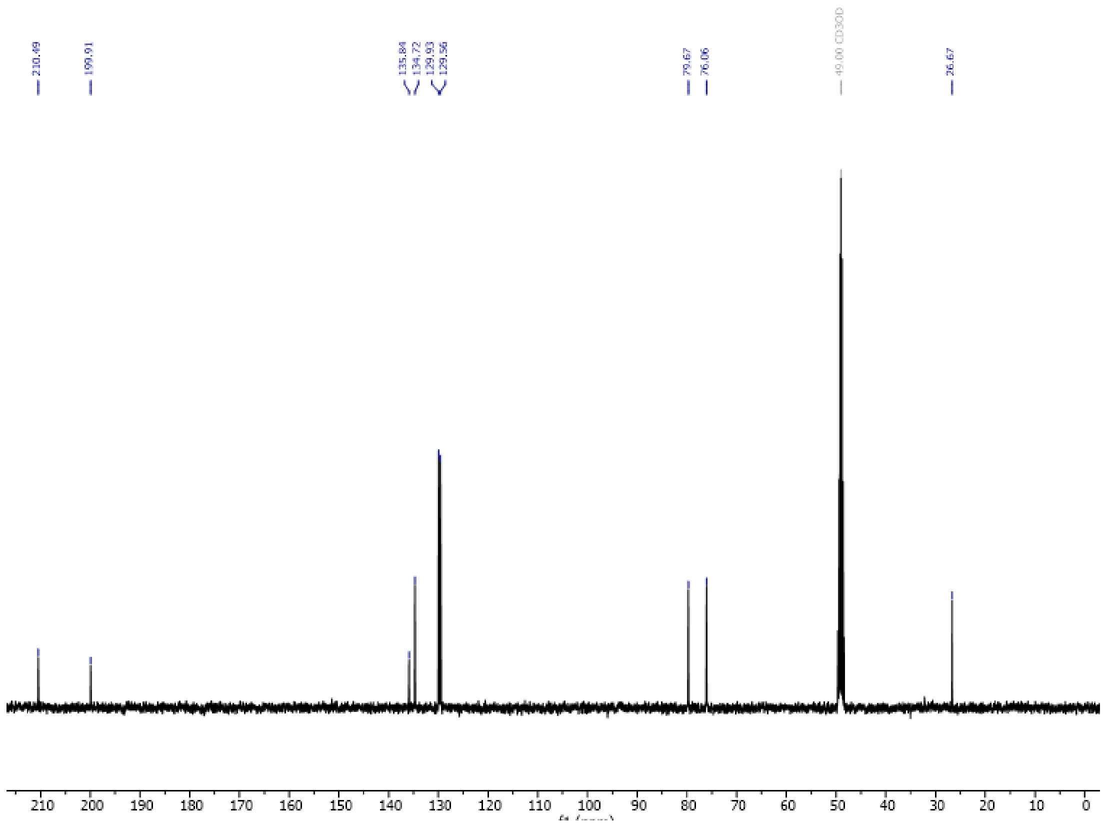


Figure S11: $^{13}C$ NMR of biocatalytically synthesized **7a**.

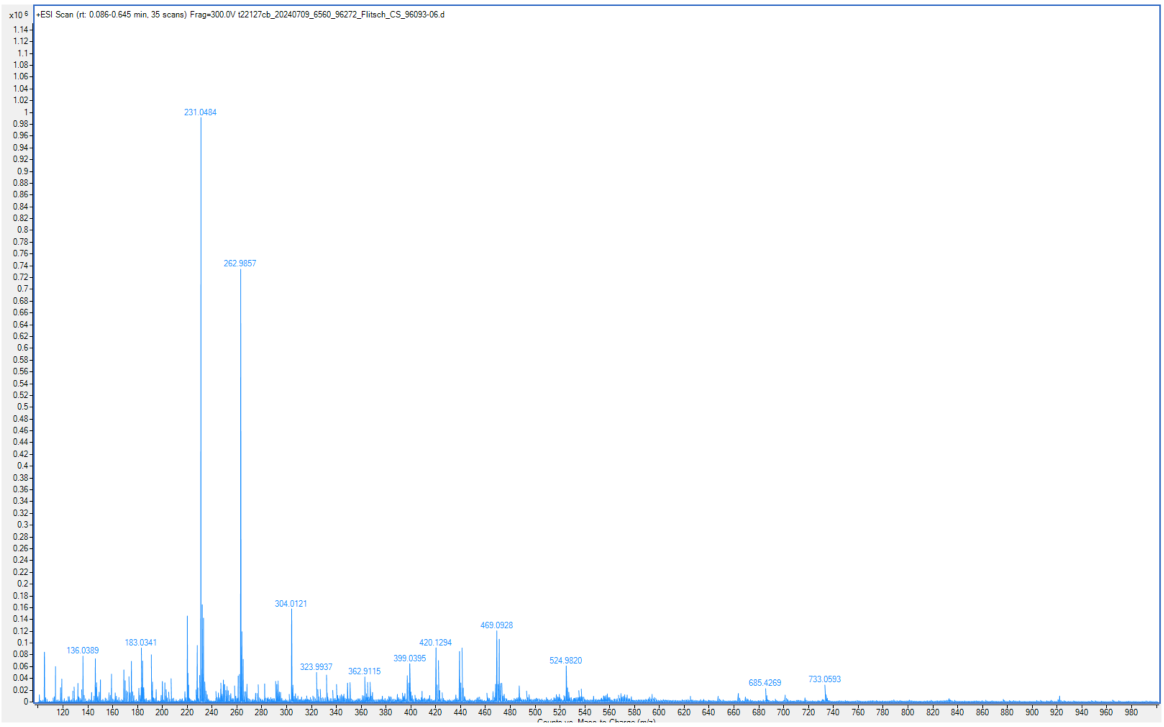


Figure S12: HRMS(ESI) of biocatalytically synthesized **7a**.

*2,3-dihydroxy-1-phenylhexane-1,4-dione* ***7c***

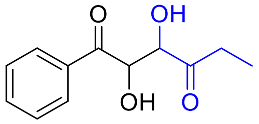


Synthesized using the general procedure above at 100 mM substrate concentration with 20% DMSO. Total reaction volume 10 mL, 1 mmol scale. Isolated yield 124 mg (56%) of an off-white solid. δH (400 MHz, $CD_3OD$) 8.02 (d, *J* = 8.1 Hz, 2H, 2x ArC**H**CCO), 7.65 (t, *J* = 7.4 Hz, 1H, ArC**H**), 7.54 (dd, *J* = 8.1, 7.4 Hz, 2H, 2x ArC**H**CHCCO), 5.58 (d, *J* = 2.2 Hz, 1H, C**H**OH), 4.47 (d, *J* = 2.2 Hz, 1H, C**H**OH), 2.86 – 2.64 (m, 2H, C$\mathbf{H_2}$$CH_3$), 1.08 (t, *J* = 7.2 Hz, 3H, $CH_2$C$\mathbf{H_3}$). δC (101 MHz, $CD_3OD$) 213.03 ($CH_3CH_2$**C**O), 200.04 (Ph**C**O), 135.84 (Ar**C**), 134.72 (Ar**C**H), 129.93 (Ar**C**H), 129.56 (Ar**C**H), 79.34 (**C**HOH), 76.23 (**C**HOH), 32.97 (**C**$H_2CH_3$), 7.48 (**C**$H_3$). HRMS(ESI) *m/z* calculated for $C_{12}H_{14}O_4$ $[M+Na]^+$ 245.0784, observed 245.0702 $[M+Na]^+$.

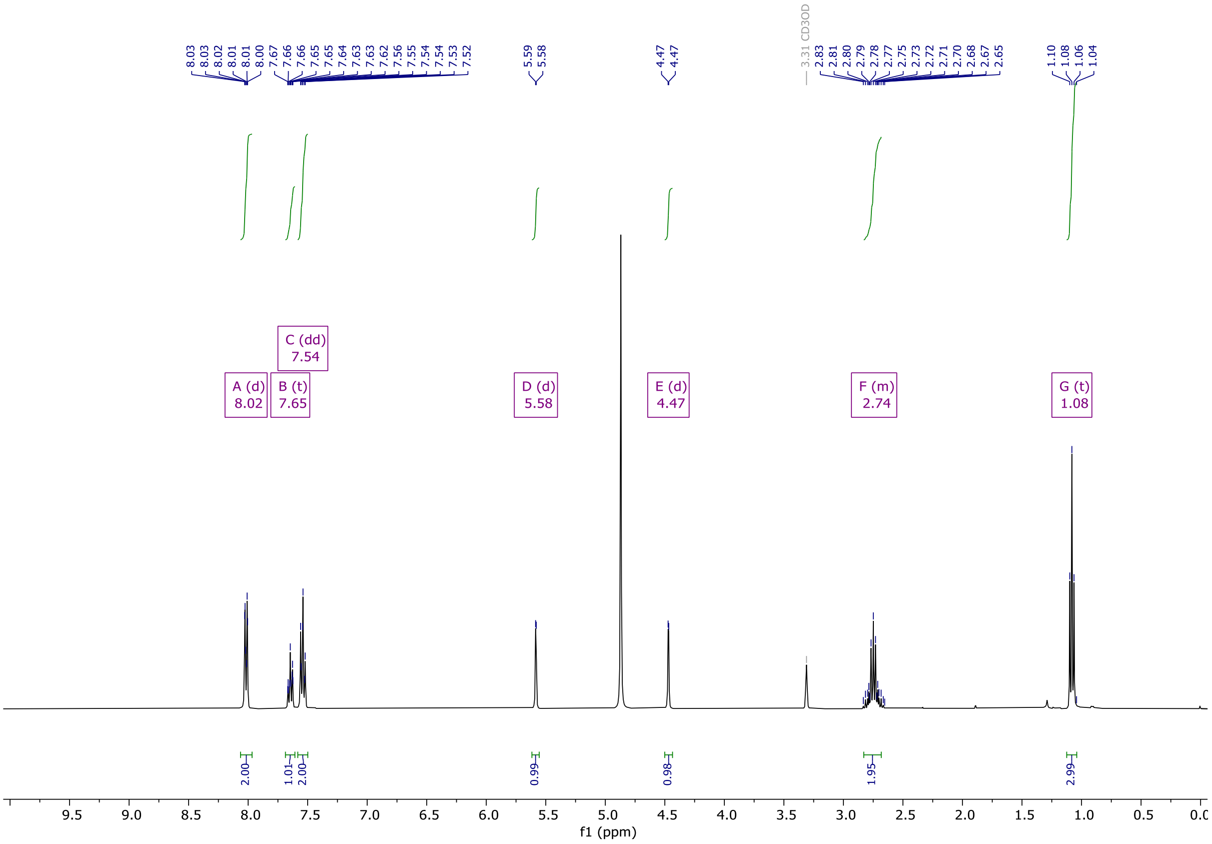


Figure S13: [1]H NMR of biocatalytically synthesized **7c**.

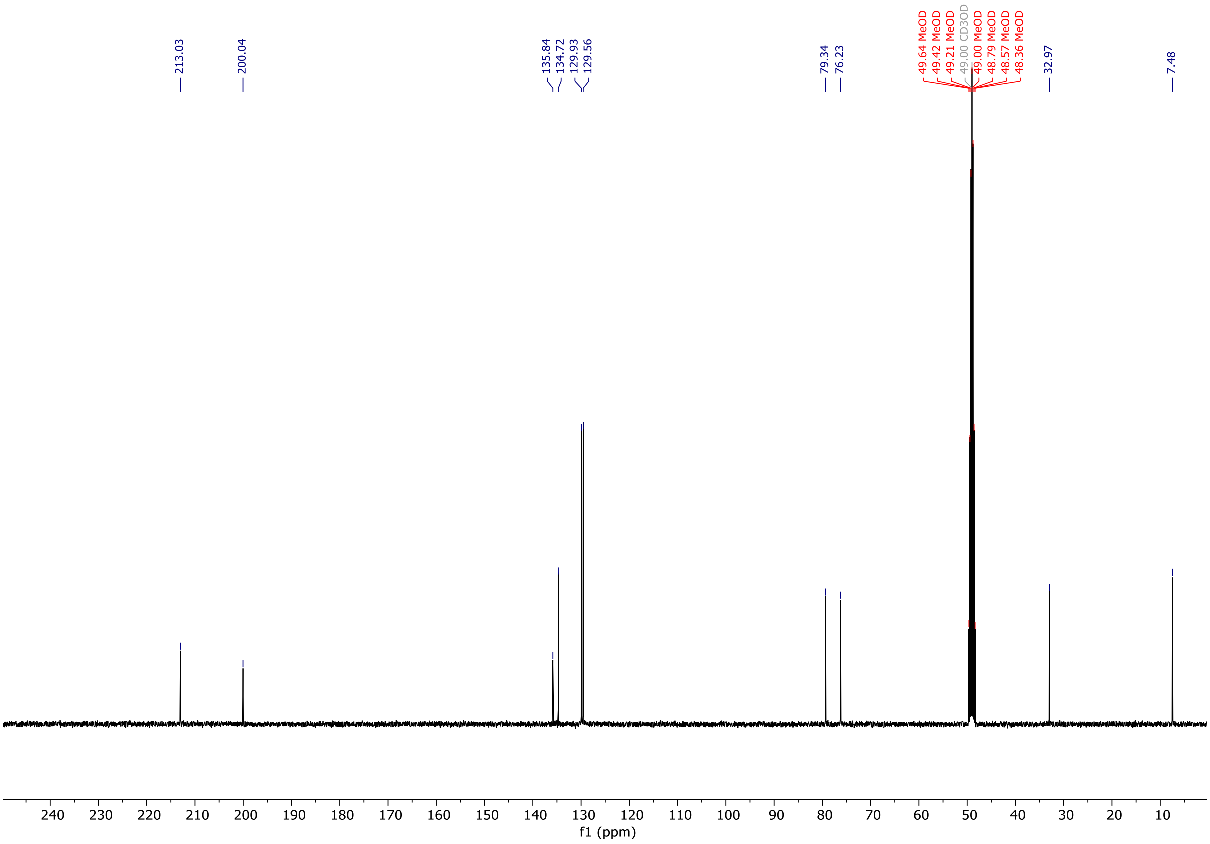

Figure S14: $^{13}$C NMR of biocatalytically synthesized **7c**.

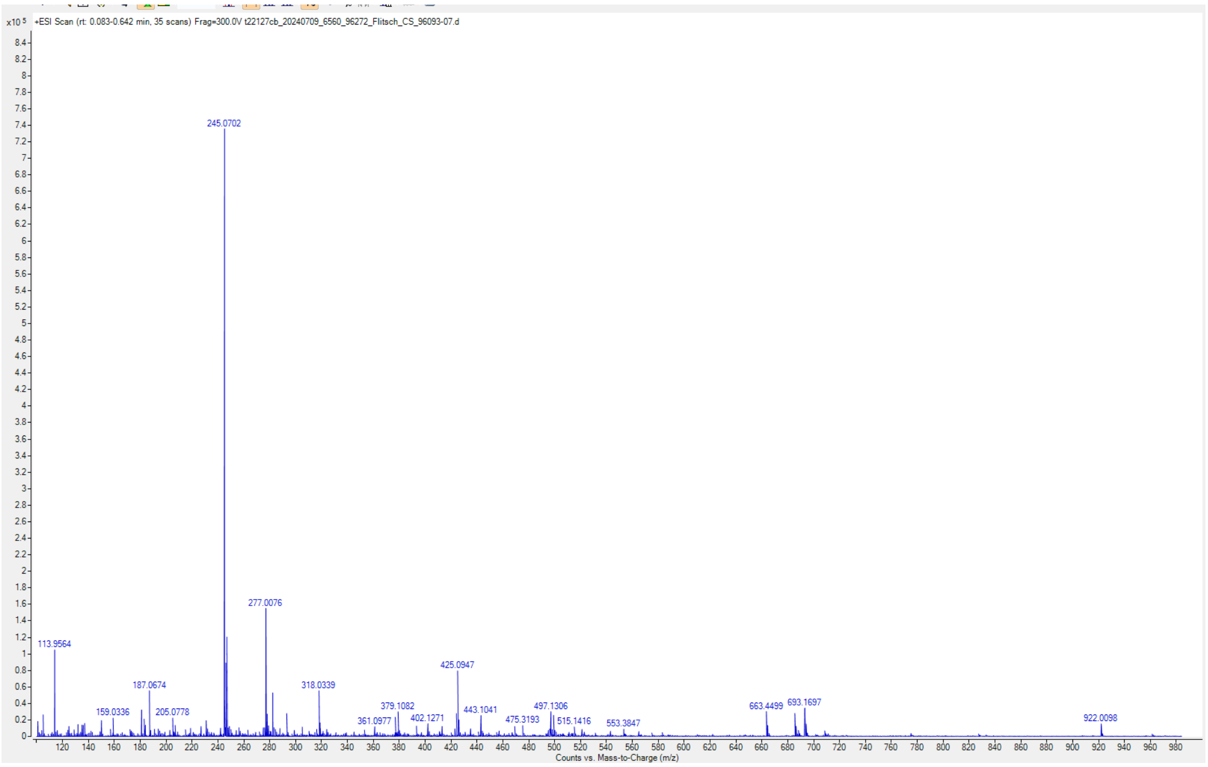

Figure S15: HRMS(ESI) of biocatalytically synthesized **7c**.

*1-(4-bromophenyl)-2,3-dihydroxypentane-1,4-dione **8a***

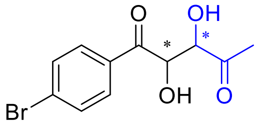


Synthesized using the general procedure above at 25 mM substrate concentration with 20% DMSO. Total reaction volume 50 mL, 1.25 mmol scale. Isolated yield 289 mg (80%) of an off-white solid. δH (400 MHz, $CD_3OD$) 7.97 – 7.90 (m, 2H, ArC**H**), 7.75 – 7.68 (m, 2H, ArC**H**), 5.51 (d, *J* = 2.3 Hz, 1H, C**H**OH), 4.47 (d, *J* = 2.3 Hz, 1H, C**H**OH), 2.65 (s, 3H, C**H₃**). δC (101 MHz, $CD_3OD$) 209.00 (**C**O), 197.86 (**C**O), 133.54 (Ar), 131.80 (Ar), 130.01 (Ar), 128.06 (Ar), 78.17 (**C**HOH), 74.92 (**C**HOH), 39.04 (DMSO), 25.30 (**C**$H_3$). HRMS(ESI) *m/z* calculated for $C_{11}H_{11}BrO_4$ $[M+Na]^+$ 308.9733 and 310.9712, observed 308.9675 and 310.9675 $[M+Na]^+$.

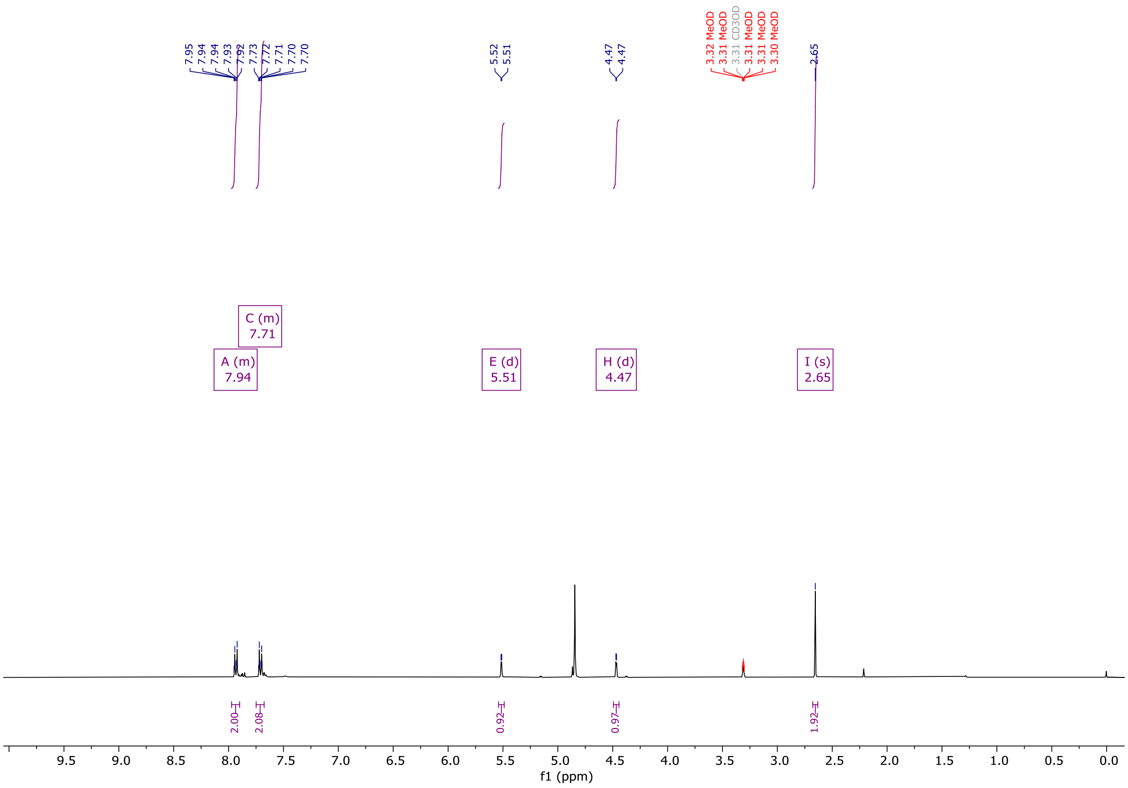


Figure S16: $^1$H NMR of biocatalytically synthesized **8a**.

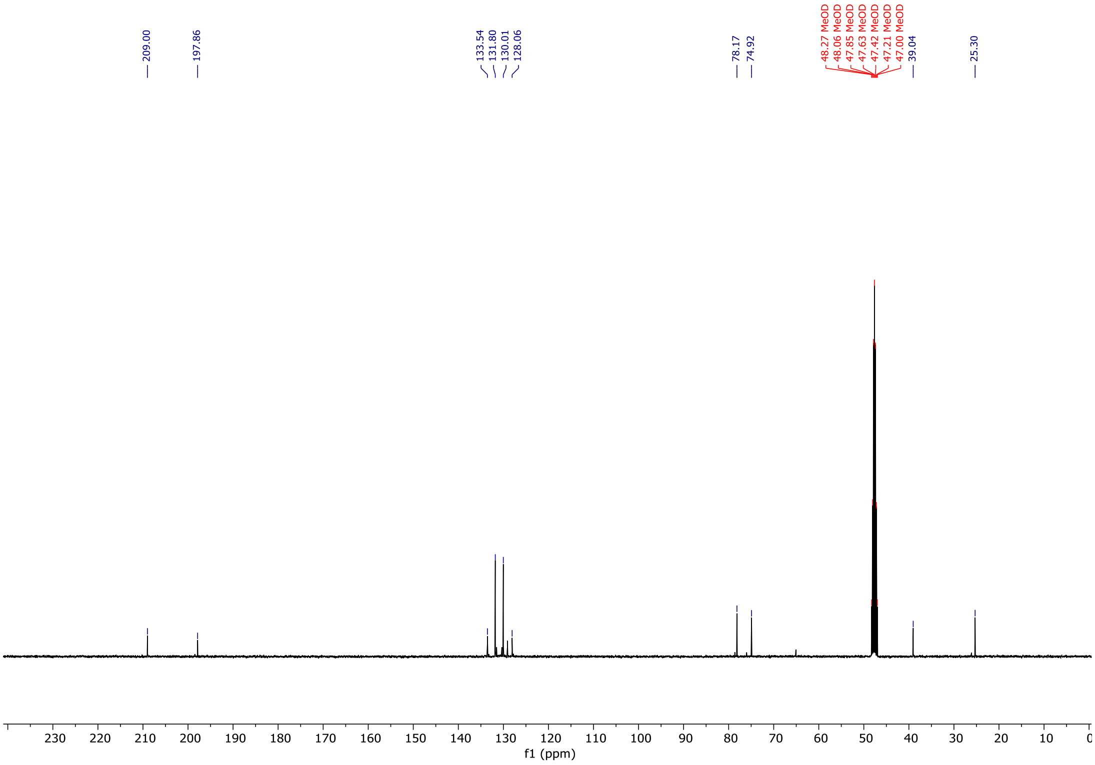


Figure S17: ${}^{13}C$ NMR of biocatalytically synthesized **8a**. Several impurity peaks are present from traces of the glyoxal substrate in the NMR sample.

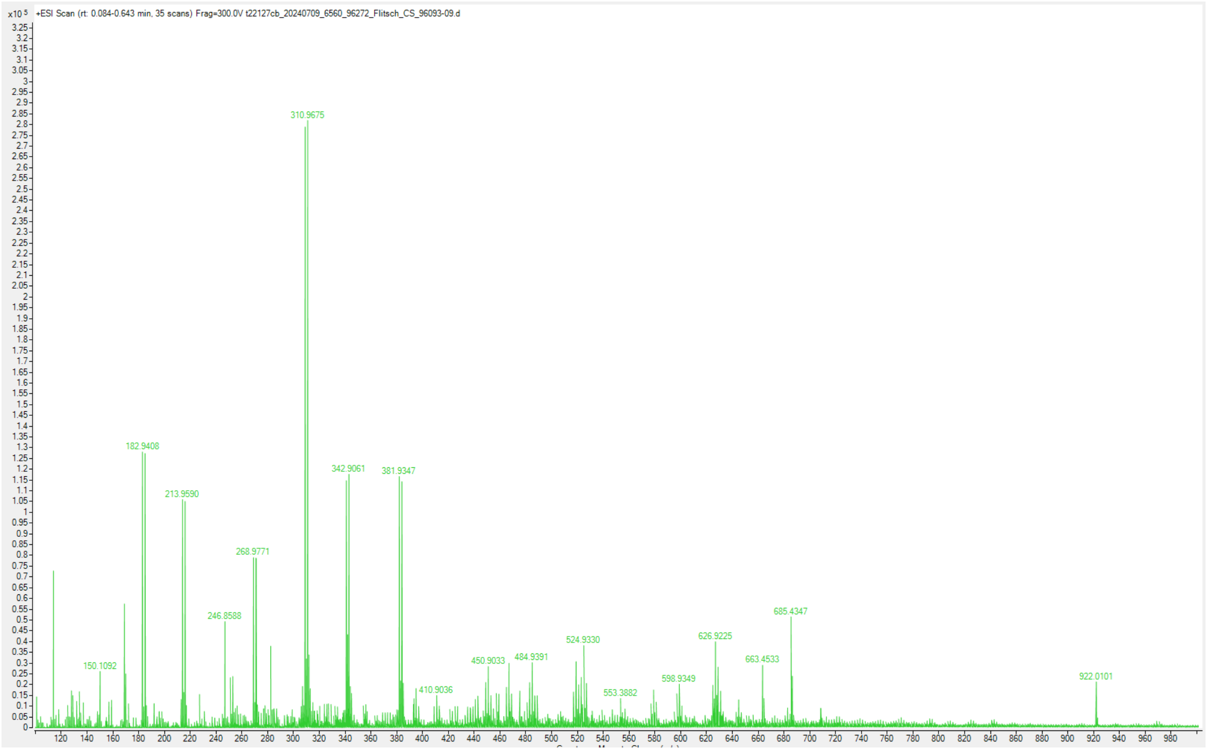


Figure S18: HRMS(ESI) of biocatalytically synthesized **8a**.

*(2S,3S)-1-(4-fluorophenyl)-2,3-dihydroxypentane-1,4-dione* ***9a***

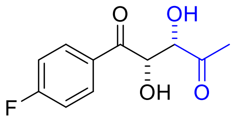


Synthesized using the general procedure above at 100 mM substrate concentration with 10% DMSO. Total reaction volume 50 mL, 5 mmol scale. Isolated yield 571 mg (51%) of a white solid. Stereochemistry determined as *2S,3S* by XRD. δH (400 MHz, $CD_3OD$) 8.13 – 8.05 (m, 2H, Ar), 7.29 – 7.20 (m, 2H, Ar), 5.57 (d, $J$ = 1.9 Hz, 1H, C**H**OH), 4.51 (d, $J$ = 1.8 Hz, 1H, C**H**OH), 2.34 (s, 3H, C**H**$_3$). δF (376 MHz, $CD_3OD$) -106.62. δC (101 MHz, $CD_3OD$) 210.41 (**C**O), 198.43 (**C**O), 167.20 (d, $^1J_{CF}$ = 253.8 Hz, 1C, Ar**C**F), 132.54 (d, $^3J_{CF}$ = 9.2 Hz, 2C, ArCFC**C**H), 116.83 (d, $^2J_{CF}$ = 22.4 Hz, 2C, ArCF**C**H). 79.55 (**C**HOH), 75.99 (**C**HOH), 26.64 (**C**H$_3$). HRMS(ESI) *m/z* calculated for $C_{11}H_{11}FO_4$ $[M+Na]^+$ 249.0534, observed 249.0560 $[M+Na]^+$.

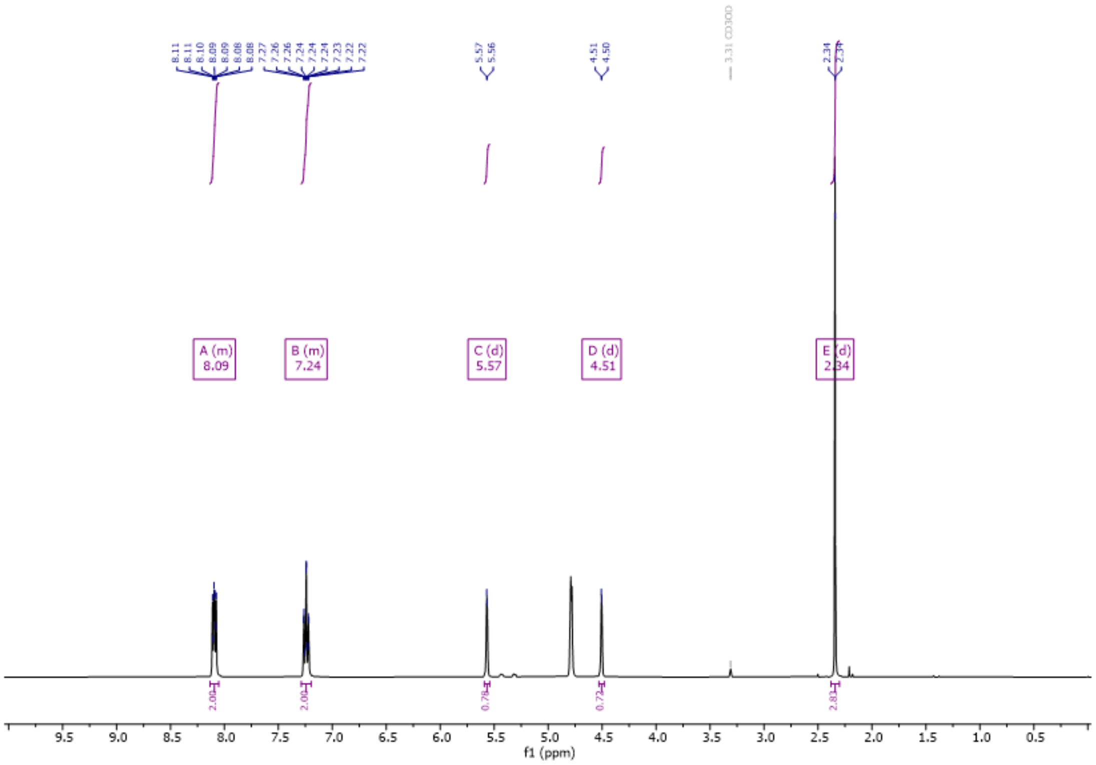


Figure S19: $^1$H NMR of biocatalytically synthesized **9a**.

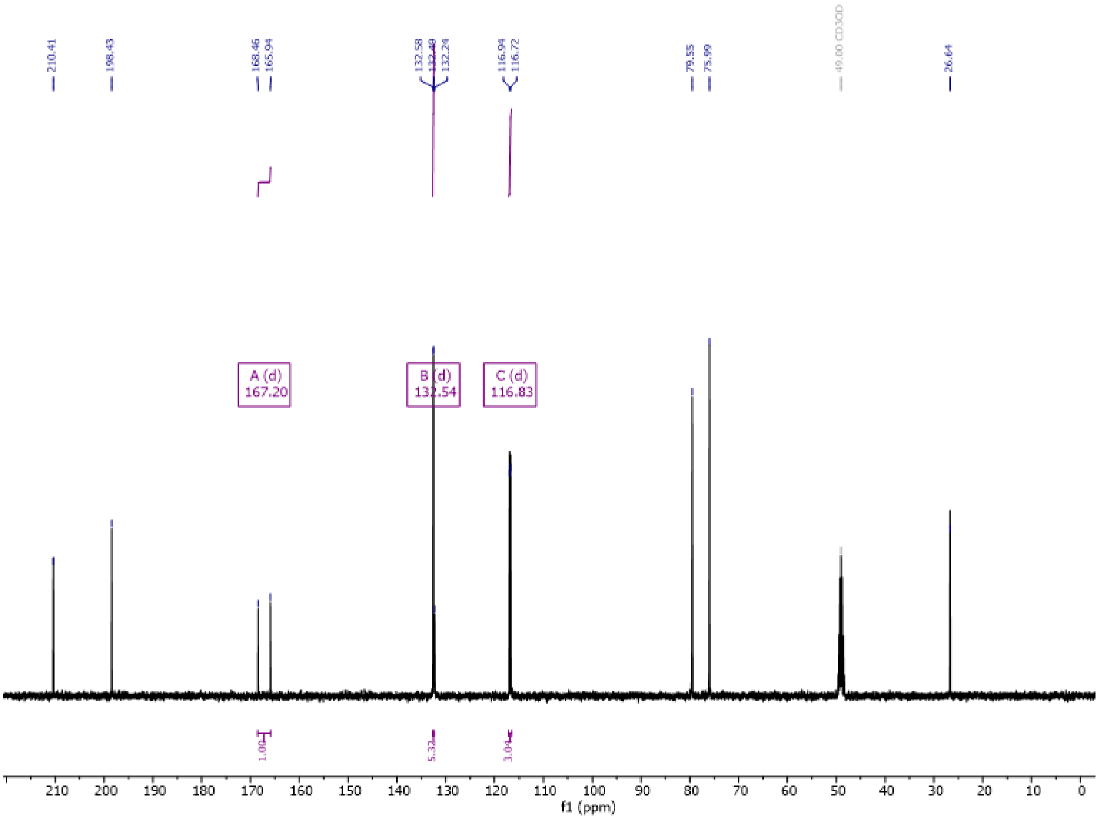


Figure S20: [13]C NMR of biocatalytically synthesized **9a**.

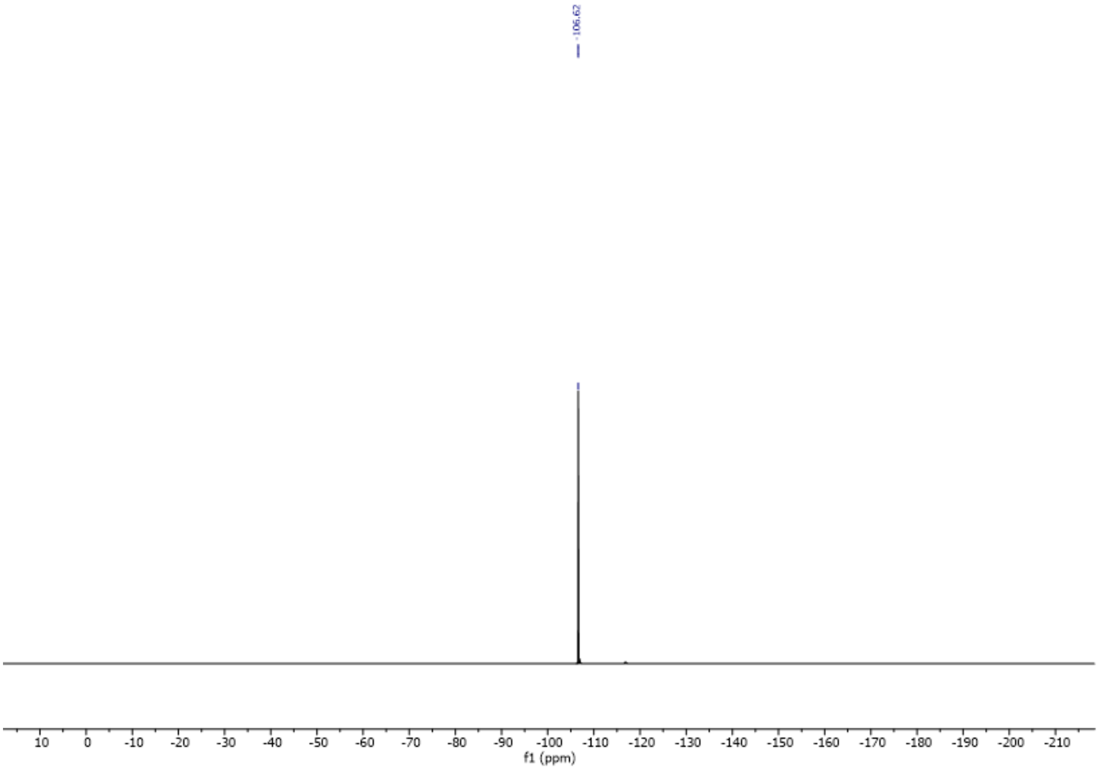


Figure S21: [19]F NMR of biocatalytically synthesized **9a**.

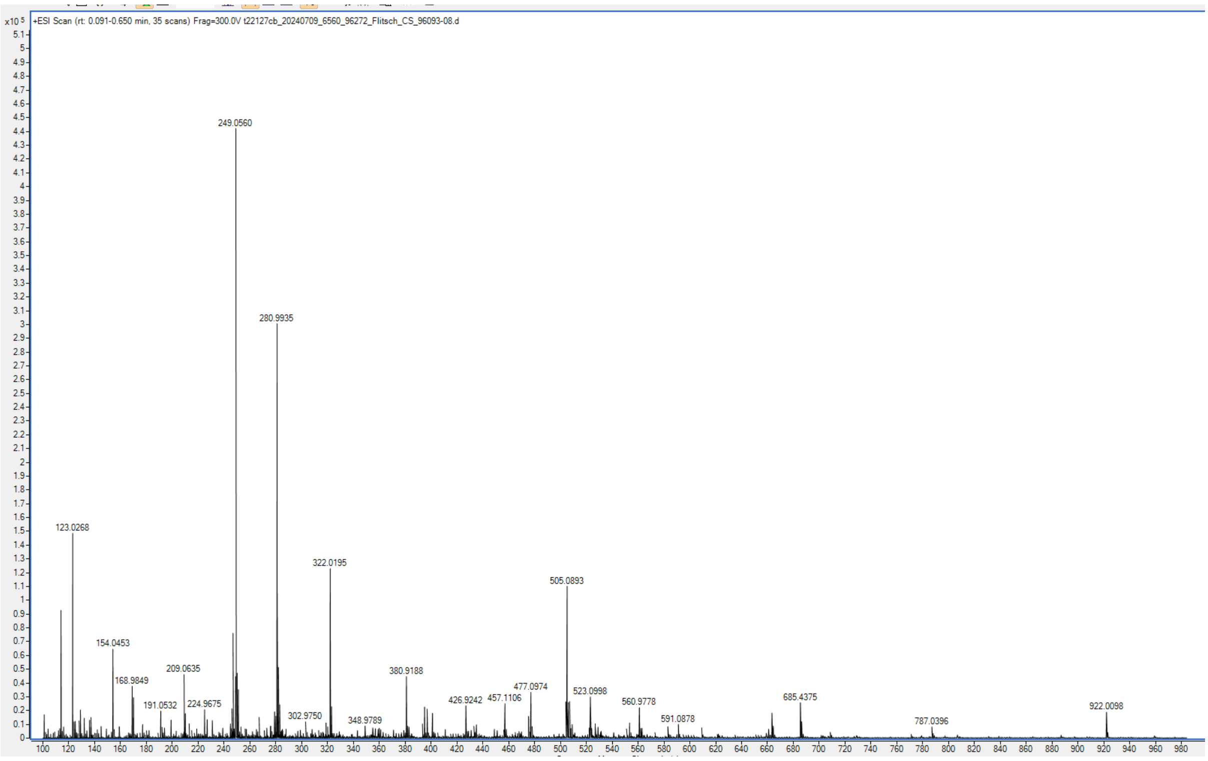

Figure S22: HRMS(ESI) of biocatalytically synthesized **9a**.

*1-(5-bromothiophen-2-yl)-2,3-dihydroxypentane-1,4-dione* ***14a***

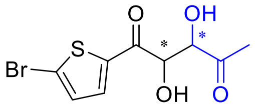

Synthesized using the general procedure above at 50 mM substrate concentration with 20% DMSO. Total reaction volume 50 mL, 2.5 mmol scale. Isolated yield 592 mg (81%) of an off-white solid. δH (400 MHz, $CD_3OD$) 7.85 (d, *J* = 4.1 Hz, 1H, ArC**H**), 7.25 (d, *J* = 4.1 Hz, 1H, ArC**H**), 5.13 (d, *J* = 2.4 Hz, 1H, C**H**OH), 4.55 (d, *J* = 2.4 Hz, 1H, C**H**OH), 2.31 (s, 3H, C**H**$_3$). δC (101 MHz, $CD_3OD$) 209.24 (**C**O), 191.29 (**C**O), 141.99 (Ar**C**), 134.32 (Ar**C**H), 131.29 (Ar**C**H), 123.17 (Ar**C**), 78.88 (**C**HOH), 76.07 (**C**HOH), 39.05 (DMSO), 25.33 (**C**H$_3$).

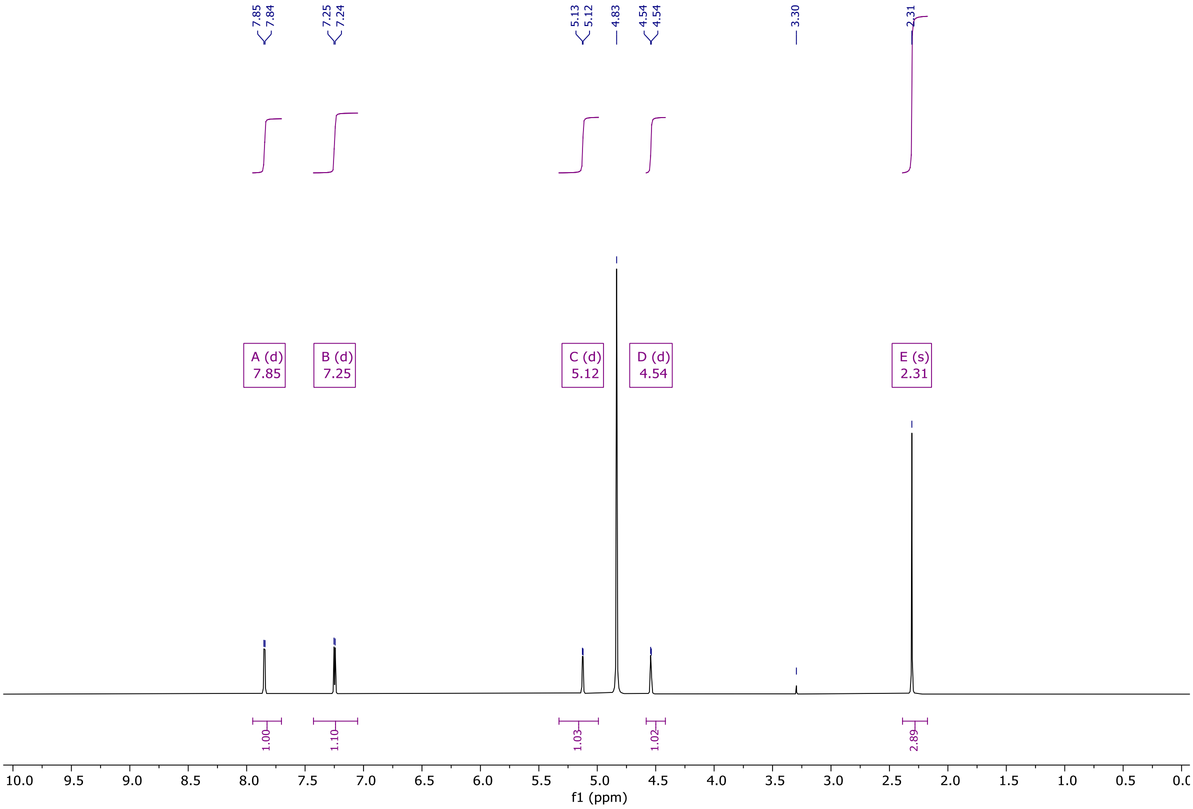


Figure S23: ¹H NMR of biocatalytically synthesized **14a**.

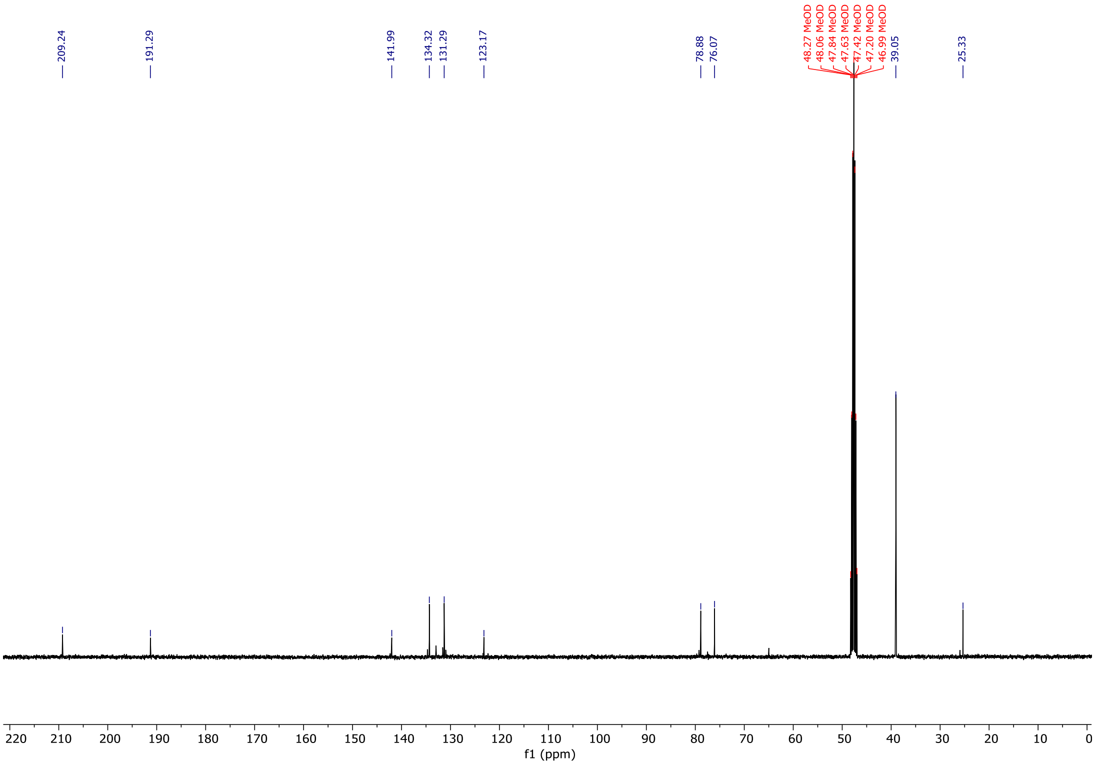


Figure S24: ¹³C NMR of biocatalytically synthesized **14a**. Several impurity peaks are present from traces of the glyoxal substrate in the NMR sample.

### *1-(2,4-difluorophenyl)-2,3-dihydroxypentane-1,4-dione **20a***

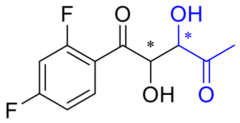

Synthesized using the general procedure above at 100 mM substrate concentration with 10% DMSO. Total reaction volume 25 mL, 2.5 mmol scale. Isolated yield 470 mg (77%) of an off-white solid. δH (400 MHz, $CD_3OD$) 8.03 – 7.91 (m, 1H, ArC**H**), 7.21 – 7.10 (m, 2H, 2x ArC**H**), 5.36 (d, *J* = 2.2 Hz, 1H, C**H**OH), 4.47 (d, *J* = 1.8 Hz, 1H, C**H**OH), 2.30 (s, 3H, C**H**$_3$). δC (101 MHz, $CD_3OD$) 209.68 ($CH_3$**C**O), 196.87 (d, $^3J_{CF}$ = 4.6 Hz, 2,4-diFPh**C**O), 167.50 (dd, *J* = 255.7, 13.0 Hz, Ar**C**F), 163.43 (dd, *J* = 255.2, 13.0 Hz, Ar**C**F), 134.23 (dd, *J* = 10.8, 4.7 Hz, Ar**C**), 121.30 (d, *J* = 17.3 Hz, Ar**C**), 113.68 (dd, *J* = 21.8, 3.3 Hz, Ar**C**), 107.53–104.07 (m, Ar**C**), 78.90 (**C**HOH), 78.69 (**C**HOH), 26.33 (**C**H$_3$).

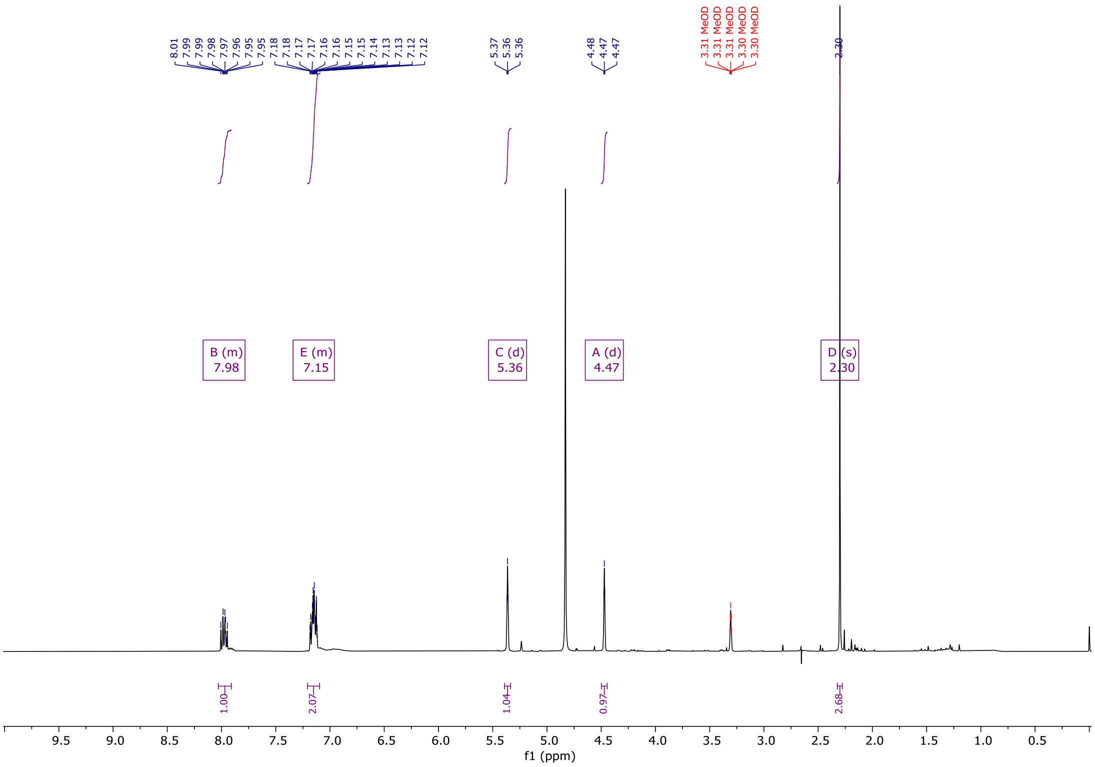


Figure S25: $^1$H NMR of biocatalytically synthesized **20a**.

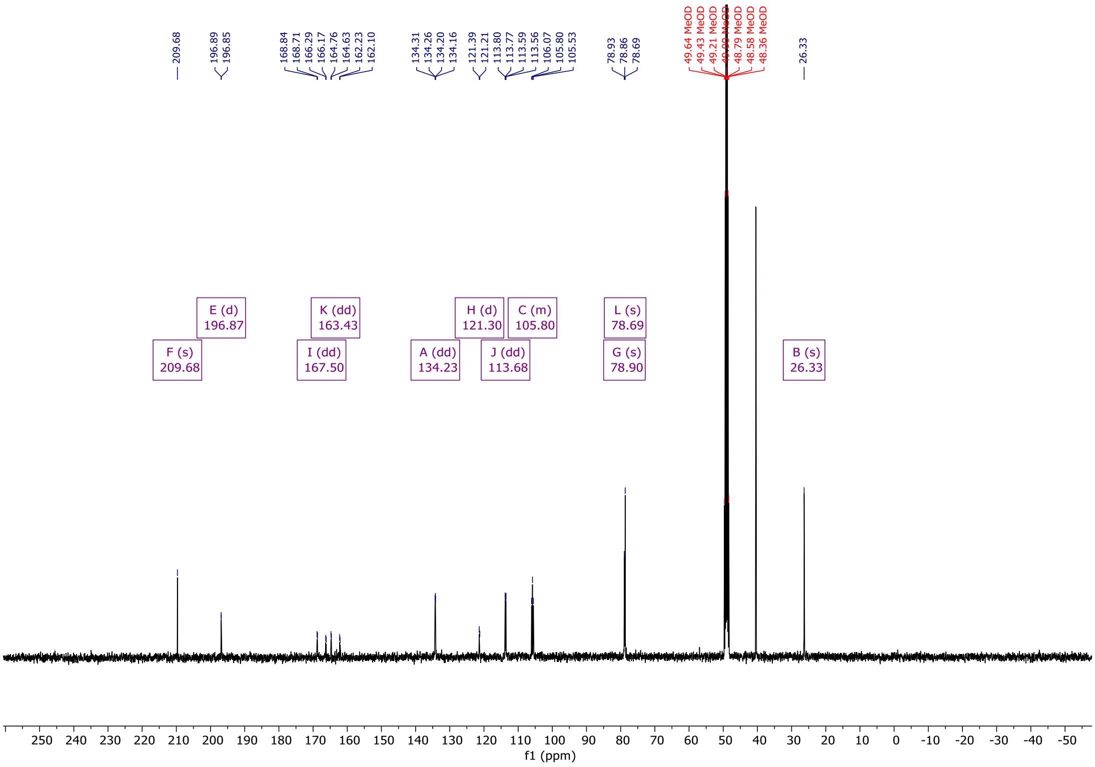


Figure S26: $^{13}$C NMR of biocatalytically synthesized **20a**.

### *2,3-dihydroxy-1-(6-methoxynaphthalen-2-yl)pentane-1,4-dione* ***21a***

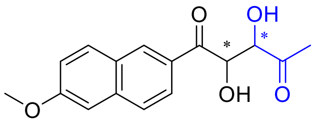


Synthesized using the general procedure above at 25 mM substrate concentration with 20% DMSO. Total reaction volume 40 mL, 1 mmol scale. Isolated yield 270 mg (93%) of a white solid. δH (400 MHz, $CD_3OD$) 8.56 (d, *J* = 1.8 Hz, 1H, ArC**H**), 8.06 – 7.93 (m, 2H, 2x ArC**H**), 7.88 (d, *J* = 8.7 Hz, 1H, ArCHC**H**), 7.32 (d, *J* = 2.5 Hz, 1H, ArC**H**), 7.23 (dd, *J* = 8.9, 2.5 Hz, 1H, ArCHC**H**), 5.71 (d, *J* = 2.3 Hz, 1H, C**H**OH), 4.56 (d, *J* = 2.3 Hz, 1H, C**H**OH), 3.95 (s, 3H, OC**H**$_3$), 2.37 (s, 3H, C**H**$_3$). δC (101 MHz, $CD_3OD$) 209.37 (**C**O), 197.98 (**C**O), 160.28 (Ar**C**), 137.78 (Ar**C**), 130.93 (Ar**C**H), 129.93 (Ar**C**H), 129.46 (Ar**C**), 127.82 (Ar**C**), 127.16 (Ar**C**H), 124.30 (Ar**C**H), 119.59 (Ar**C**H), 105.48 (Ar**C**H), 78.63 (**C**HOH), 74.54 (**C**HOH), 54.59 (O**C**H$_3$), 39.02 (DMSO), 25.38 (**C**H$_3$). HRMS(ESI) *m/z* calculated for $C_{16}H_{16}O_5$ $[M+Na]^+$ 311.0890, observed 311.0904 $[M+Na]^+$.

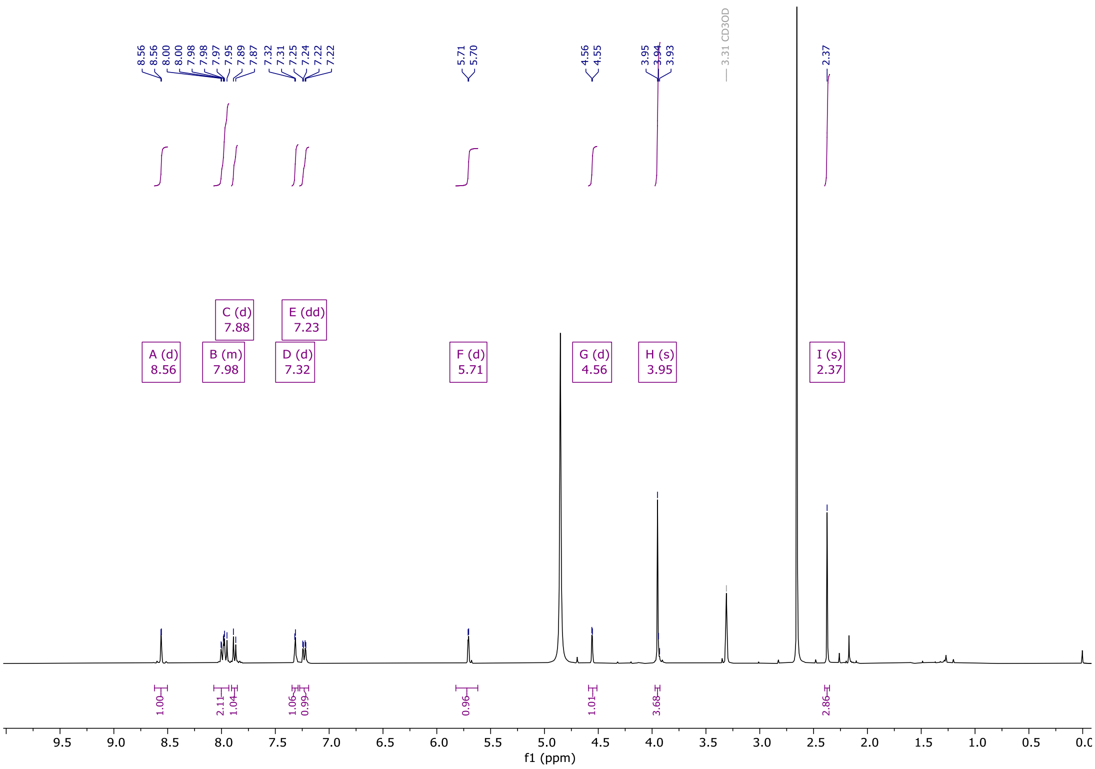


Figure S27: [1]H NMR of biocatalytically synthesized **21a**.

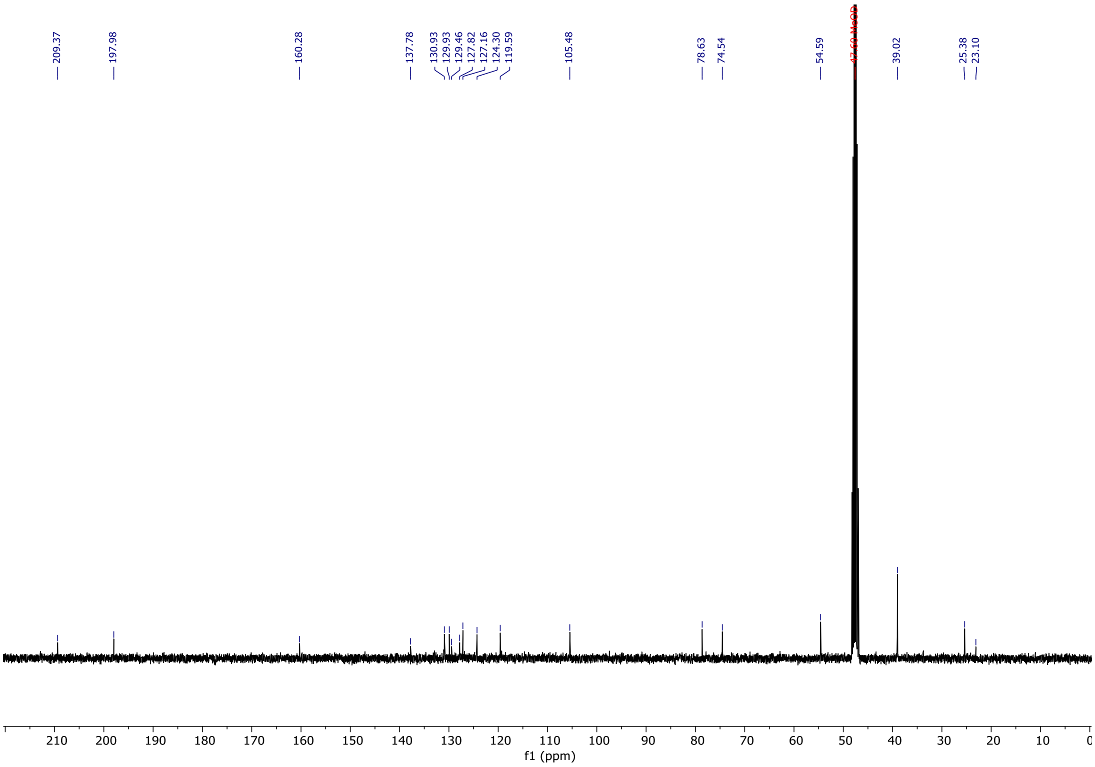


Figure S28: [13]C NMR of biocatalytically synthesized **21a**.

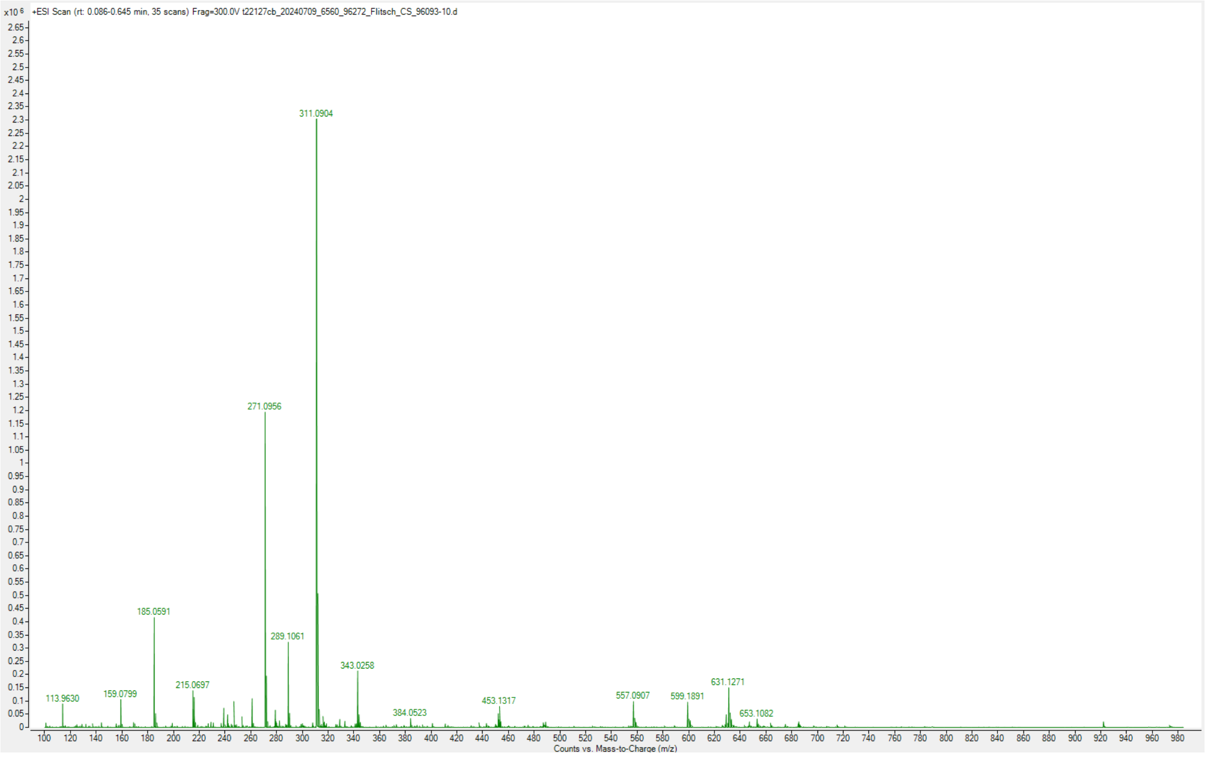


Figure S29: HRMS(ESI) of biocatalytically synthesized **21a**.

### *2,3-dihydroxy-4-oxopentanoic acid **22a***

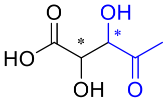


Glyoxylic acid hydrate (230mg, 2.5 mmol, 1 equiv.) and hydroxyacetone (171 µL, 2.5 mmol, 1 equiv.) were dissolved in water and the pH set to 8.0 (the final reaction volume was 25 mL, 100 mM substrate concentration). FSAwt lysate (50 mg, 2 mg/mL) was added, and the reaction incubated at 30 °C, 200 rpm for 24 hours. The reaction was monitored by $^{1}$H NMR (water suppression mode) and samples were prepared by mixing 500 µL of biotransformation mixture with 100 µL $D_2O$ and centrifuging the solution (13,000 rpm, 5 mins). The supernatant was decanted for analysis. Upon reaction completion, the pH was adjusted to 10.0, centrifuged (4,000 rpm, 15 mins) and the supernatant loaded onto a column of anion exchange resin (Dowex 1X8, $HCO_2^-$ form, 5 mL bed volume prewashed with 10 c.v. of water). The loading flow through was passed through the column a further time to ensure full binding before the column was washed with water (10 c.v., 50 mL). The product was eluted using 1M $HCO_2H$ (5 c.v., 25 mL) and the collected fraction was lyophilized. The title compound was isolated as a colourless oil (281 mg, 76% yield). δH (400 MHz, $D_2O$) 4.65 (d, *J* = 2.0 Hz, 1H), 4.50 (d, *J* = 2.0 Hz, 1H), 2.30 (s, 3H, C**H**$_3$). δC (101 MHz, $D_2O$) 212.22 ($CH_3$**C**O), 176.99 (**C**$O_2$H), 78.75 (**C**HOH), 72.44 (**C**HOH), 25.60 (**C**$H_3$).

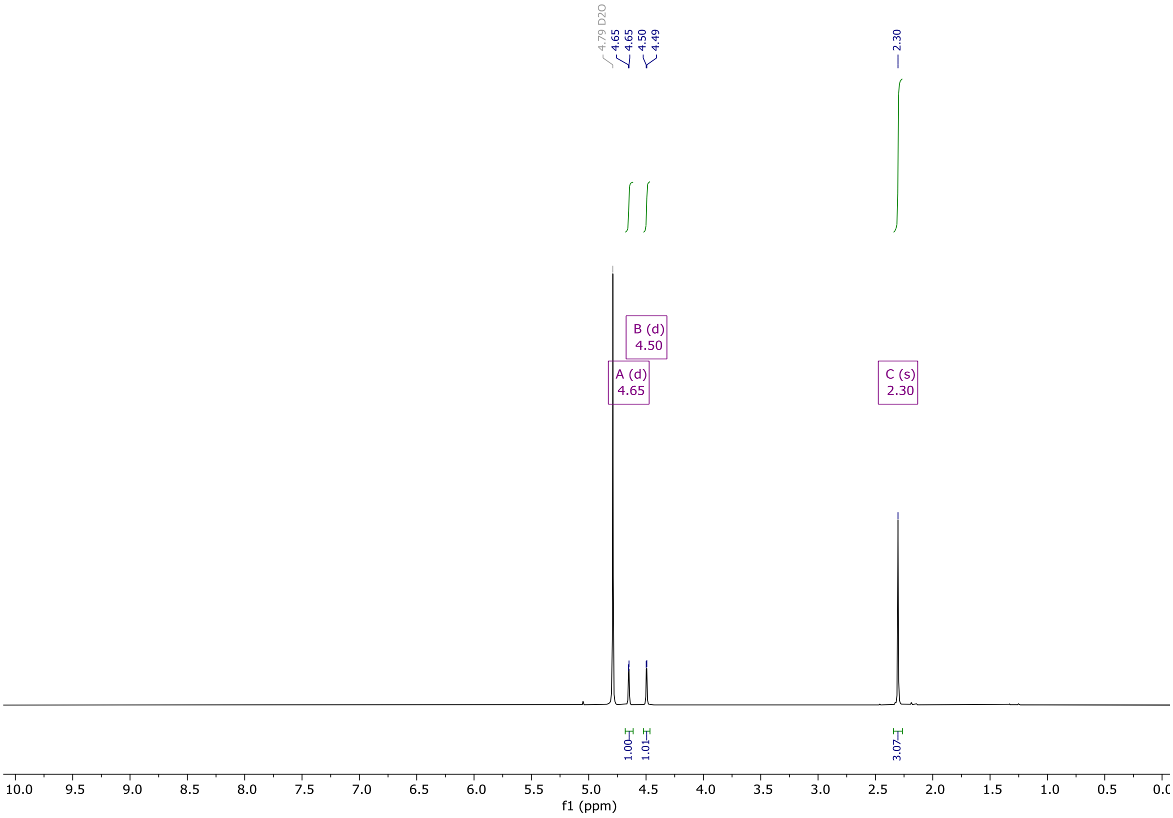


Figure S30: [1]H NMR of biocatalytically synthesized 2,3-dihydroxy-4-oxopentanoic acid **22a**.

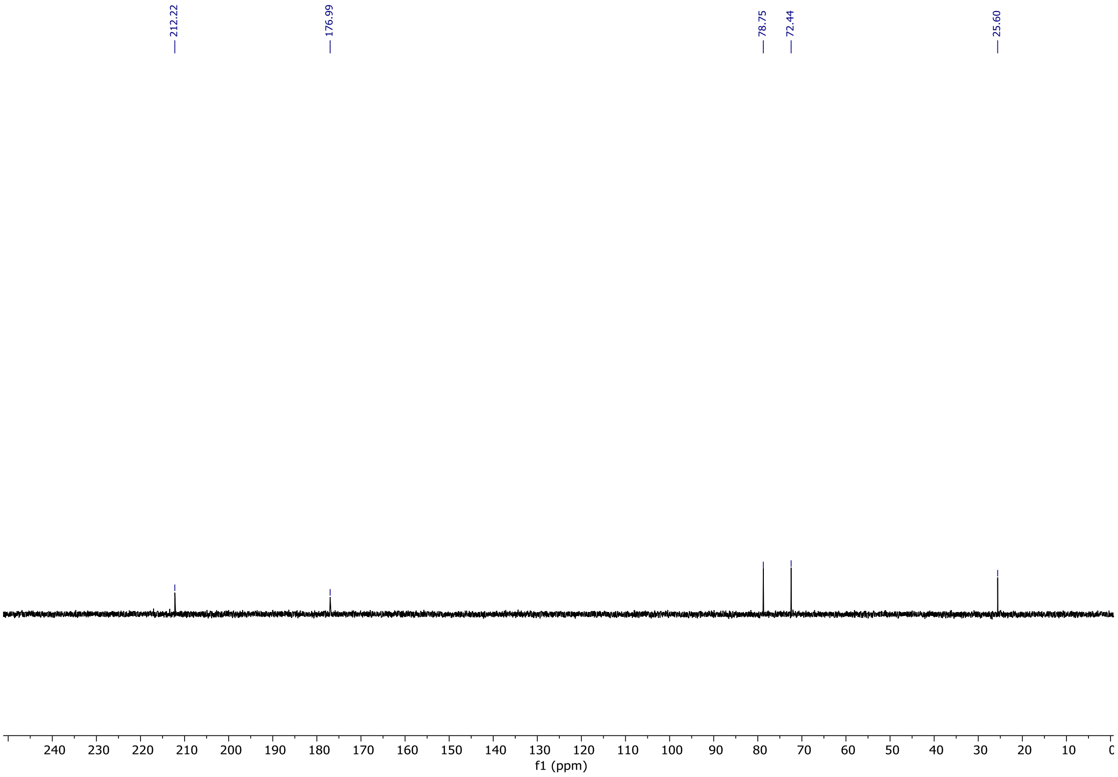


Figure S31: [13]C NMR of biocatalytically synthesized 2,3-dihydroxy-4-oxopentanoic acid **22a**.

### *Chiral analysis of aldolase products*

#### *Comparison with chemically synthesized aldol products by chiral HPLC*

Aldol products were synthesized as mixture of stereoisomers using the organocatalytic procedure below, which was modified from the literature.[2]

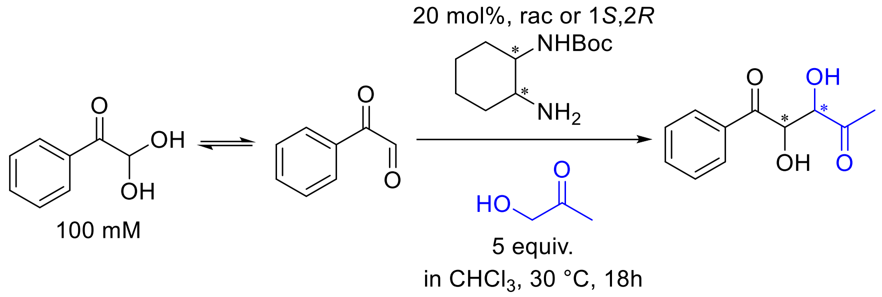


To a glass vial were charged phenylglyoxal hydrate **7** (0.2 mmol, 26.8 mg), hydroxyacetone **a** (1 mmol, 5 equiv., 70 µL) dissolved in chloroform (total reaction volume 2 mL, 100 mM substrate concentration) and *tert*-butyl-2-aminocyclohexyl carbamate (*rac* or (*S*,*R*), 0.04 mmol, 20 mol%, 8.6 mg). The reaction was mixed at 200 rpm, 30 $^{o}$C for 18 hours. Upon full conversion by $^{1}$H NMR the reaction was analyzed by chiral HPLC and NMR. Reactions with both the racemic and 1*S*,2*R* catalysts gave a mixture of stereoisomers 85:15 *d.r.* (racemic catalyst) and 84:16 *d.r.* (*S*,*R* catalyst) by $^{1}$H NMR. δH (400 MHz, $CD_3OD$) 8.02 (d, *J* = 8.1 Hz, 2H, major diastereomer), 7.97 (d, *J* = 7.8 Hz, 2H, minor diastereomer) 7.70 – 7.60 (m, 1H, both diastereomers), 7.59 – 7.45 (m, 2H, both diastereomers), 5.59 (d, *J* = 2.3 Hz, 1H, major diastereomer), 5.23 (d, *J* = 4.4 Hz, 1H, minor diastereomer), 4.47 (d, *J* = 2.2 Hz, 1H, major diastereomer), 4.40 (d, *J* = 4.4 Hz, 1H, minor diastereomer), 2.34 (s, 3H, major diastereomer) , 2.26 (s, 3H, minor diastereomer).

Samples for chiral HPLC were diluted 10-fold (to theoretical substrate concentration of 10 mM) in 80:20 hexane/2-propanol and injected onto a Daicel CHIRALPAK® IC column. Integration of peaks shows 13:5:39:43 *e.r.*, 82:18 *d.r.* for the racemic catalyst and 14:1.4:54:30.6 *e.r.*, 84.6:15.4 *d.r.* for the *S*,*R* catalyst (determined by chiral HPLC).

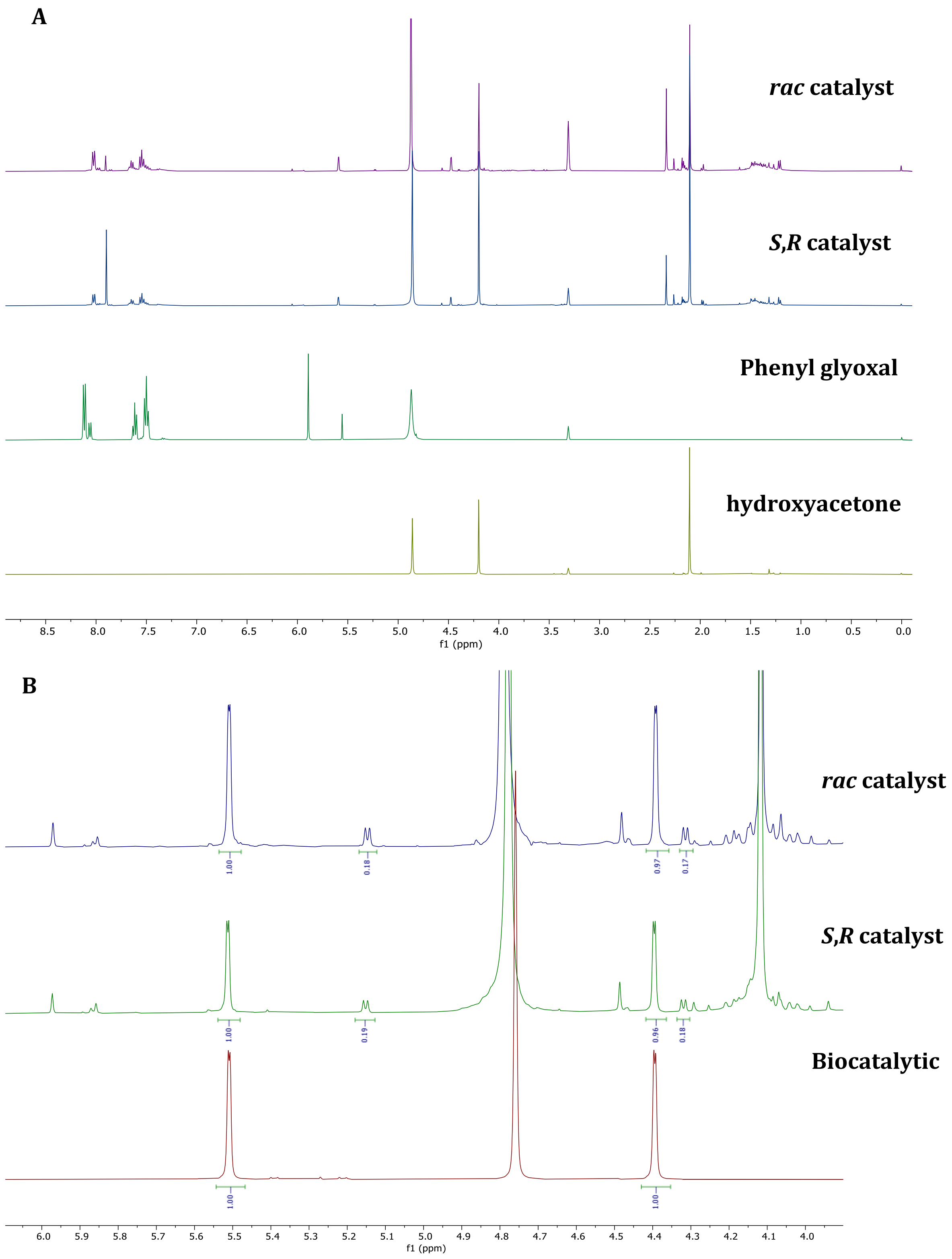


Figure S32**:** Top (**A**) – Stacked $^{1}$H NMR spectra of the reaction with racemic and *S*,*R* catalysts, phenylglyoxal and hydroxyacetone substrates (top to bottom) showing full conversion of phenylglyoxal and formation of new product peaks. Bottom (**B**) – Comparison of diastereomeric C**H**OH peaks in the 4.0 – 6.0 ppm region of the stacked spectra of the reaction with racemic (top), *S*,*R* (middle) catalysts and purified aldolase product **7a** (bottom). Integration shows 85:15 *d.r.* in the reaction with the racemic catalyst; 84:16 *d.r.* with the (1*S*,2*R*) organocatalyst and only one diastereomer in the biocatalytic product **7a**.

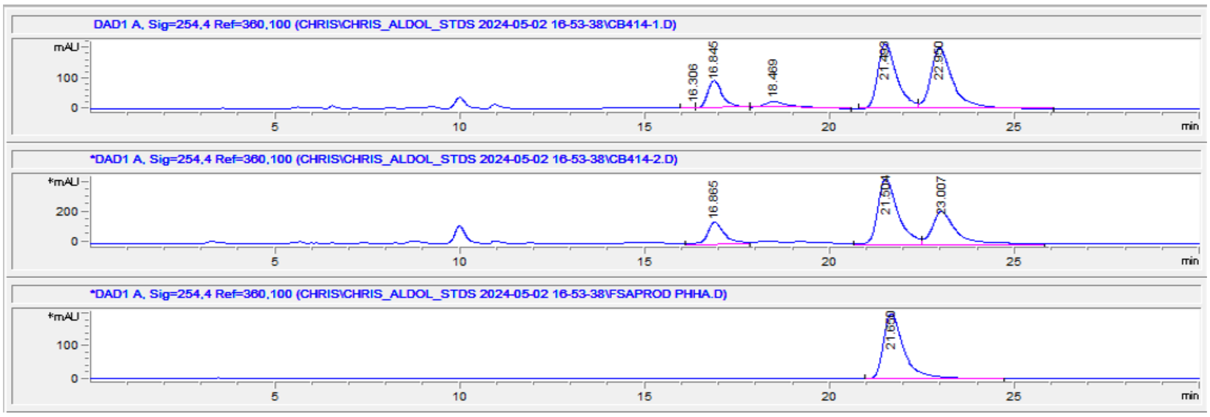


Figure S33: Overlaid chiral HPLC chromatograms of chemically synthesized stereoisomeric mixture of 2,3-dihydroxy-1-phenylpentane-1,4-dione **7a** with racemic catalyst (top), *S*,*R* catalyst (middle) and the biocatalytically synthesized product **7a** on a CHIRALPAK® IC column. Elution was performed with a 90:10 mixture of hexane/isopropanol over 30 minutes.

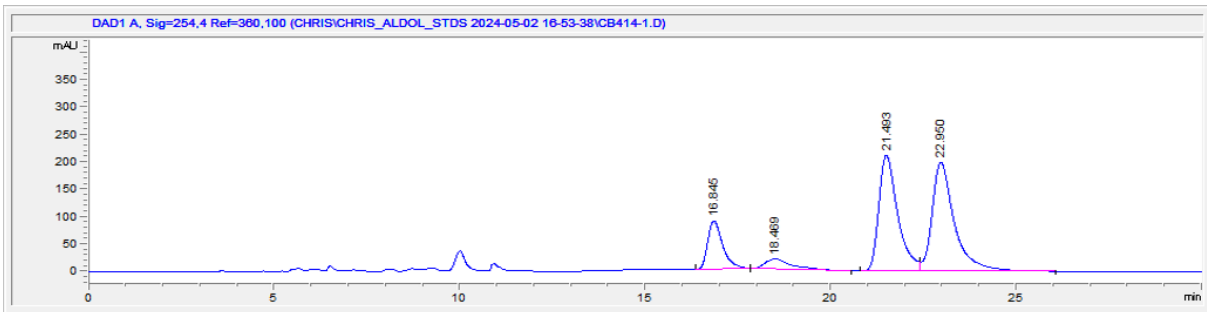


| Retention time / min | Area | Area% |
|---|---|---|
| 16.845 | 2471.7 | 13 |
| 18.469 | 928.9 | 5 |
| 21.493 | 7455.6 | 39 |
| 22.95 | 8250.5 | 43 |

Figure S34: Chiral HPLC chromatogram of chemically synthesized stereoisomeric mixture of 2,3-dihydroxy-1-phenylpentane-1,4-dione **7a** with racemic catalyst on a CHIRALPAK® IC column. Elution was performed with a 90:10 mixture of hexane/isopropanol over 30 minutes.

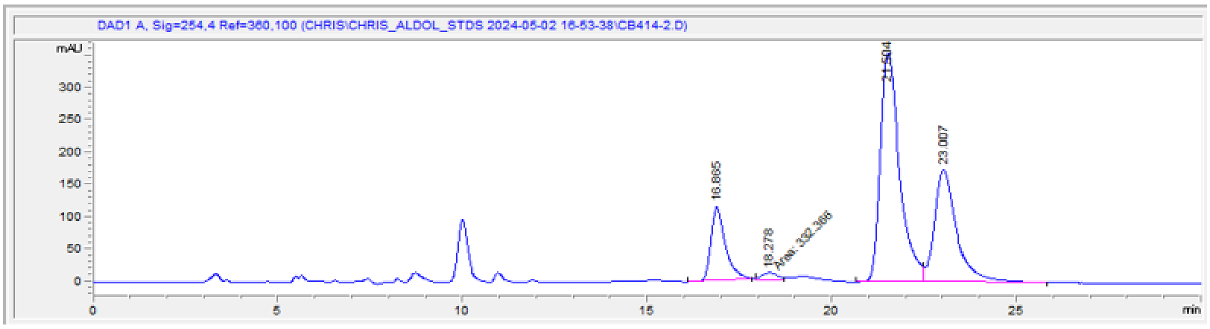


| Retention time / min | Area | Area% |
|---|---|---|
| 16.865 | 3258.4 | 14 |
| 18.278 | 332.4 | 1.4 |
| 21.504 | 12737.6 | 54 |
| 23.007 | 7203.3 | 30.6 |

Figure S35: Chiral HPLC chromatogram of chemically synthesized stereoisomeric mixture of 2,3-dihydroxy-1-phenylpentane-1,4-dione **7a** with *S*,*R* catalyst on a CHIRALPAK® IC column. Elution was performed with a 90:10 mixture of hexane/isopropanol over 30 minutes.

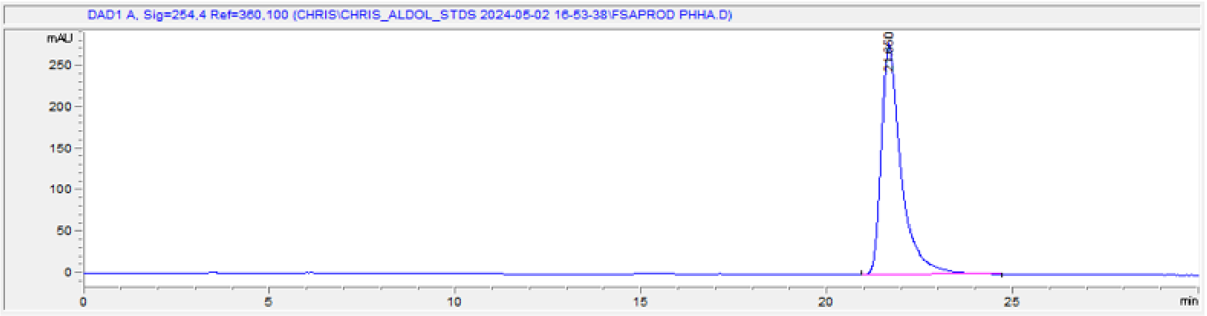


| Retention time / min | Area | Area% |
|---|---|---|
| 21.65 | 10502.3 | 100 |

Figure S36: Chiral HPLC chromatogram of biocatalytically synthesized 2,3-dihydroxy-1-phenylpentane-1,4-dione **7a** on a CHIRALPAK® IC column. Elution was performed with a 90:10 mixture of hexane/isopropanol over 30 minutes.

### *Crystallisation and XRD analysis*

Biocatalytically synthesized aldol adducts were crystalized from methanol by diffusion with *n*-pentane. Samples were dissolved in minimal methanol followed by layering with *n*-pentane or placed inside a larger vessel containing *n*-pentane and sealed.

```
Bond precision:    C-C = 0.0030 A          Wavelength=1.54184

Cell:               a=4.85907(17)       b=8.6605(3)       c=24.1701(7)
                    alpha=90            beta=90           gamma=90
Temperature:        100 K
```

| | Calculated | Reported |
|---|---|---|
| Volume | 1017.13(6) | 1017.12(6) |
| Space group | P 21 21 21 | P 21 21 21 |
| Hall group | P 2ac 2ab | P 2ac 2ab |
| Moiety formula | C11 H12 O4 | C11 H12 O4 |
| Sum formula | C11 H12 O4 | C11 H12 O4 |
| Mr | 208.21 | 208.21 |
| Dx,g cm-3 | 1.360 | 1.360 |
| Z | 4 | 4 |
| Mu (mm-1) | 0.870 | 0.870 |
| F000 | 440.0 | 440.0 |
| F000' | 441.54 | |
| h,k,lmax | 6,10,30 | 5,10,30 |
| Nref | 2129[ 1283] | 2077 |
| Tmin,Tmax | 0.959,0.971 | 0.784,1.000 |
| Tmin' | 0.860 | |

```
Correction method= # Reported T Limits: Tmin=0.784 Tmax=1.000
AbsCorr = MULTI-SCAN

Data completeness= 1.62/0.98        Theta(max)= 76.054

R(reflections)= 0.0311( 1982)                      wR2(reflections)=
                                                   0.0752( 2077)
S = 1.051                  Npar= 184
```

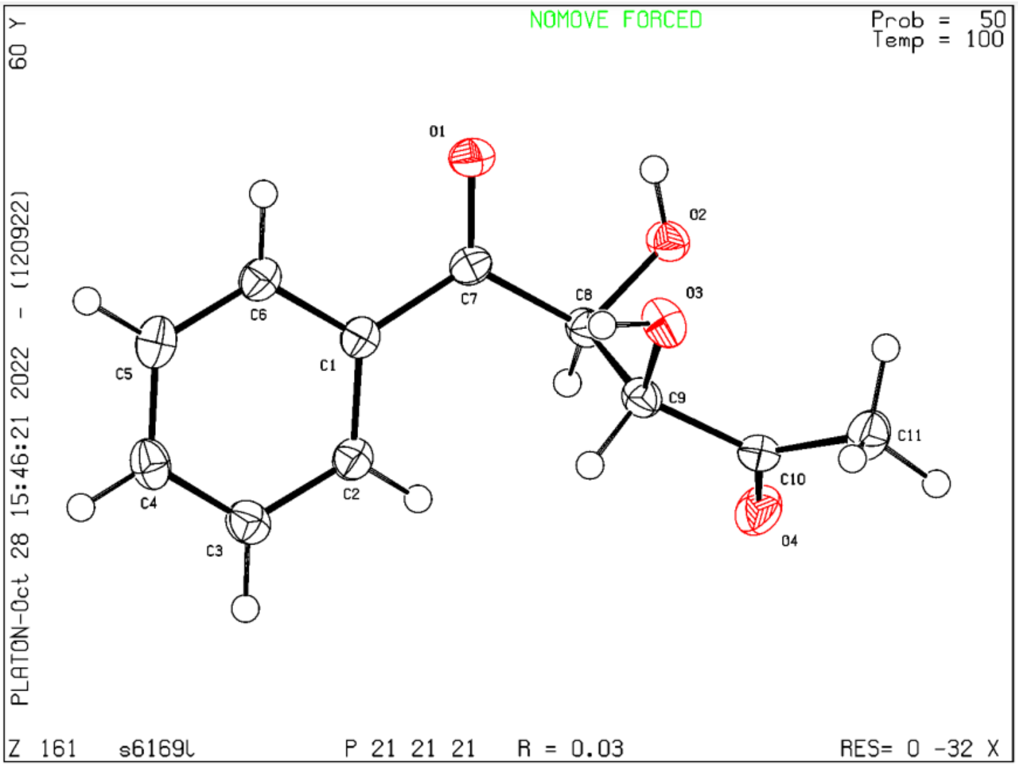


Figure S37: Crystal structure report for biocatalytically synthesized **7a** (CCDC 2360546).

Bond precision: C-C = 0.0040 A Wavelength=1.54184

| | | | |
|---|---|---|---|
| Cell: | a=4.9101(3) | b=10.1545(5) | c=10.2058(5) |
| | alpha=90 | beta=97.106(5) | gamma=90 |
| Temperature: | 100 K | | |

| | Calculated | Reported |
|---|---|---|
| Volume | 504.95(5) | 504.95(5) |
| Space group | P 21 | P 1 21 1 |
| Hall group | P 2yb | P 2yb |
| Moiety formula | C11 H11 F O4 | C11 H11 F O4 |
| Sum formula | C11 H11 F O4 | C11 H11 F O4 |
| Mr | 226.20 | 226.20 |
| Dx,g cm-3 | 1.488 | 1.488 |
| Z | 2 | 2 |
| Mu (mm-1) | 1.073 | 1.073 |
| F000 | 236.0 | 236.0 |
| F000' | 236.91 | |
| h,k,lmax | 6,12,12 | 5,12,12 |
| Nref | 2109[ 1116] | 1960 |
| Tmin,Tmax | 0.928,0.969 | 0.430,1.000 |
| Tmin' | 0.786 | |

Correction method= # Reported T Limits: Tmin=0.430 Tmax=1.000
AbsCorr = MULTI-SCAN

Data completeness= 1.76/0.93 Theta(max)= 76.259

R(reflections)= 0.0400( 1883) wR2(reflections)= 0.1143( 1960)

S = 1.096 Npar= 149

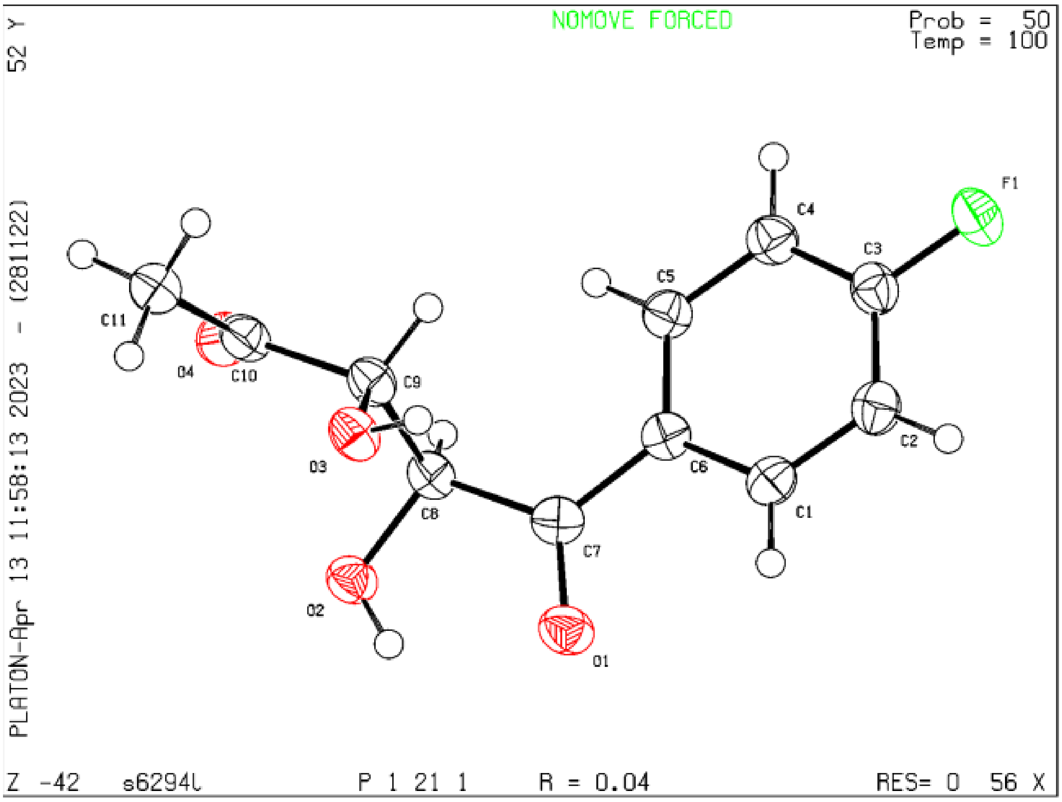


Figure S38: Crystal structure report for biocatalytically synthesized **9a** (CCDC 2360547).

*Transaminase biotransformations*

*Screening of TA for activity towards FSA aldol products*

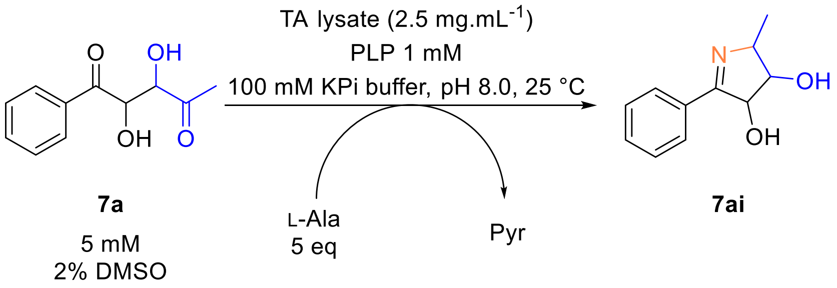


Reactions were carried out in 2 mL Eppendorf tubes with a total reaction volume of 500 µL. Each reaction contained components diluted from stock solutions, with final concentrations of 5 mM diketone substrate **7a**, 25 mM L-alanine (5 eq.), 1 mM PLP and 2.5 mg.mL$^{-1}$ transaminase lysates in 100 mM KPi buffer pH 8.0 containing 2 v% DMSO. Reactions were incubated at 30 °C, 200 rpm for 24h and followed by $^1$H NMR analysis. Analytical samples ($^1$H NMR) were prepared by adding 350 µL of $D_2O$ to 250 µL of biotransformation. The conversions have been calculated from the characteristic signal doublet in aromatic area from the substrates and the product (**Figure S39**). A no enzyme control reaction with all other components shows only substrates signals of **7a**, L-Alanine and DMSO (**Figure S40**).

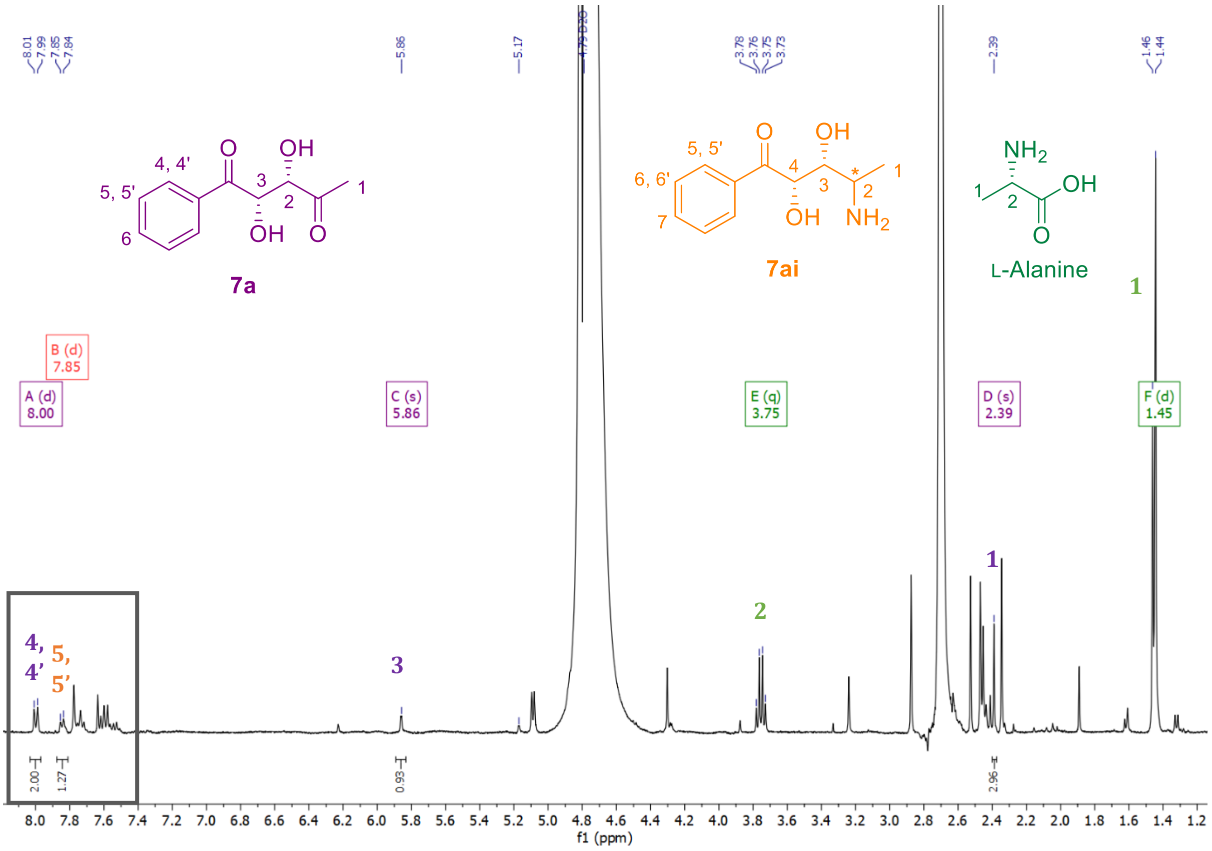


Figure S39: $^1$H NMR of crude biotransformation of aldol **7a** catalyzed by pQR2191 TA.

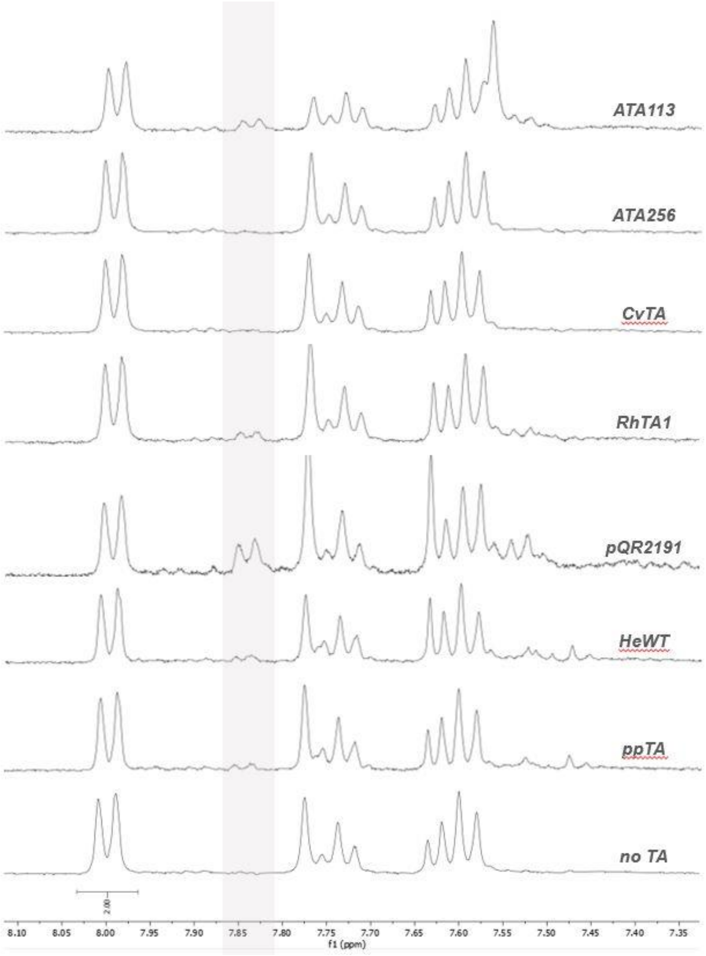


| Transaminase | 7a integration δ8.00 ppm (H) | 7ai integration δ7.86 ppm (H) | Conversions (%) |
|---|---|---|---|
| ATA113 | 2 | 0.25 | 11 |
| ATA256 | 2 | nd. | - |
| CvTA[3] | 2 | nd. | - |
| RhTA1[4] | 2 | 0.34 | 15 |
| pQR2191[5] | 2 | 1.18 | 37 |
| HeWT[6] | 2 | nd. | - |
| ppTA[7] | 2 | nd. | - |

Figure S40: $^{1}$H NMR of crude biotransformation of aldol **7a** catalyzed by various TA and the corresponding conversions.

### *Optimization of the transamination step*

#### *Various pQR2191 TA concentrations*

Reactions were carried out in 2 mL Eppendorf tubes with a total reaction volume of 500 µL. Each reaction contained components diluted from stock solutions, with final concentrations of 25 mM diketone substrate **7a**, 250 mM IPA (10 eq.), 1 mM PLP and 0.5, 1 or 2 mg.mL$^{-1}$ purified transaminase pQR2191 in 100 mM TEA buffer pH 8.0 containing 10 v% DMSO. Reactions were incubated at 30 °C, 200 rpm for 24h and followed by $^{1}$H NMR analysis. Analytical samples ($^{1}$H NMR) were prepared by adding 350 µL of $D_2O$ to 250 µL of biotransformation. The conversions have been calculated from the characteristic signal doublet in aromatic area from the substrate (δ8.00 ppm) and the product (δ7.85 ppm).

### *Various IPA concentrations*

Reactions were carried out in 2 mL Eppendorf tubes with a total reaction volume of 500 µL. Each reaction contained components diluted from stock solutions, with final concentrations of 25 mM diketone substrate **7a**, 50, 125 or 250 mM IPA (2eq., 5 eq. or 10 eq.), 1 mM PLP and 0.5 mg.mL$^{-1}$ purified transaminase pQR2191 in 100 mM TEA buffer pH 8.0 containing 10 v% DMSO. Reactions were incubated at 30 °C, 200 rpm for 24h and followed by $^{1}$H NMR analysis. Analytical samples ($^{1}$H NMR) were prepared by adding 350 µL of $D_2O$ to 250 µL of biotransformation. The conversions have been calculated from the characteristic signal doublet in aromatic area from the substrate (δ8.00 ppm) and the product (δ7.85 ppm).

Table S5**:** Conversions observed with various pQR2191 TA and IPA concentrations using $^{1}$H NMR analysis**.**

| pQR2191 (mg.mL$^{-1}$) | IPA (eq.) | Conversion (%) |
|---|---|---|
| 0.5 | | **26** |
| 1 | 2 | **66** |
| 2 | | **99** |
| | 2 | **26** |
| 0.5 | 5 | **69** |
| | 10 | **99** |

### *Implementation of the telescoped FSA – TA cascade*

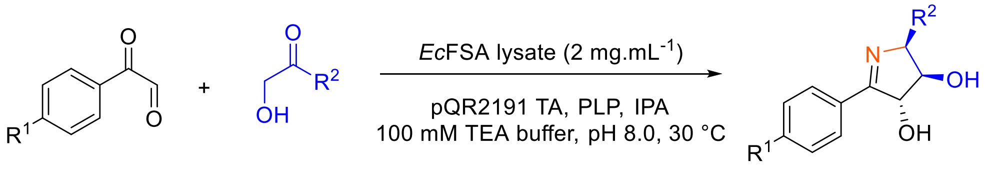


FSA aldol adduct

Reactions were carried out in 2 mL Eppendorf tubes with a total reaction volume of 625 µL. Each reaction contained components diluted from stock solutions, with final concentrations of 20 mM ketoaldehyde substrate **7**, **8** or **9**, 20 mM donor substrate **a** or **b**, 200 mM IPA (10 eq.), 1 mM PLP, 2 mg.mL$^{-1}$ *Ec*FSAwt lysate and 0.5, 1 or 2 mg.mL$^{-1}$ purified transaminase pQR2191 in 100 mM TEA buffer pH 8.0 containing 10 v% DMSO. Reactions were incubated at 30 °C, 200 rpm for 24h and followed by $^{1}$H NMR analysis. Analytical samples ($^{1}$H NMR) were prepared by adding 350 µL of $D_2O$ to 250 µL of biotransformation. The conversions of the aldol reaction have been calculated comparing signals integration of the donor substrate (at δ2.13 ppm for **a**), the aldol product **7a** (at δ8.00 ppm for **7a**), and the product of transamination (at δ7.85 ppm for **7ai**) as it directly formed from the aldol product. The conversions of the transamination step have been calculated comparing signals integration of the aldol product (at δ8.00 ppm for **7a**), and the product of transamination (at δ7.85 ppm for **7ai**). An example applied to the synthesis of **7ai** is detailed below. All the conversions are summarized in **Table S6**.

## *Implementation of the sequential FSA – TA cascade*

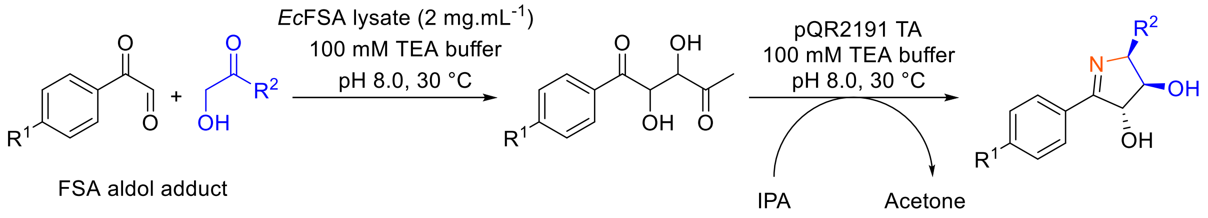


Aldol reactions were carried out in 2 mL Eppendorf tubes with a total reaction volume of 500 µL. Each reaction contained components diluted from stock solutions, with final concentrations of 20 mM ketoaldehyde substrate **7**, **8** or **9**, 20 mM donor substrate **a** or **c** and 2 mg.mL$^{-1}$ *Ec*FSAwt lysate in 100 mM TEA buffer pH 8.0 containing 10 v% DMSO. Reactions were incubated at 30 °C, 200 rpm for 24h and followed by $^{1}$H NMR analysis. The conversions of the aldol reaction have been calculated as described above.

After 24h, transamination reactions were carried out in 2 mL Eppendorf tubes containing the aldol reaction with a final volume of 625 µL. At each aldol reaction are added components diluted from stock solution with final concentration of 200 mM IPA (10 eq.), 1 mM PLP and 0.5, 1 or 2 mg.mL$^{-1}$ purified transaminase pQR2191 in mM TEA buffer pH 8.0. Reactions were incubated at 30 °C, 200 rpm for 24h and followed by $^{1}$H NMR analysis. The conversions of the transamination reaction have been calculated as described above. All the conversions are summarized on the following **Table S6**.

Table S6**:** Conversions obtained with FSA – TA cascades for the synthesis of **7ai**, **8ai**, **9ai** and **7ci**.

| **Product** | **pQR2191 TA (mg.ml$^{-1}$)** | **Telescoped cascade** AL Conv (%) | **Telescoped cascade** TA conversion (%) | **Telescoped cascade** Overall conversion (%) | **Sequential cascade** AL Conv (%) | **Sequential cascade** TA conversion (%) | **Sequential cascade** Overall conversion (%) |
|---|---|---|---|---|---|---|---|
| 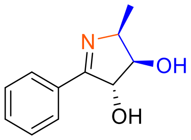 **7ai** | 0.5 | 93 | 65 | 60 | 94 | 85 | 81 |
| | 1 | 96 | 93 | **89*** | 94 | ≥99 | 94 |
| | 2 | ≥99 | 85 | 85 | 94 | ≥99 | 94 |
| 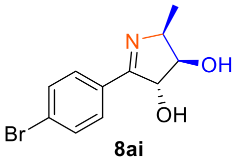 **8ai** | 0.5 | 51 | 0 | - | 66 | ≥99 | 65 |
| | 1 | 36 | ≥99 | 36 | 71 | ≥99 | **71*** |
| | 2 | 35 | ≥99 | 35 | 62 | ≥99 | 62 |
| 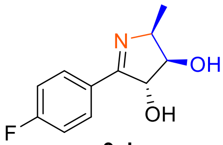 **9ai** | 0.5 | 78 | 53 | 42 | 76 | 99 | 75 |
| | 1 | 88 | ≥99 | **88*** | 76 | 99 | 75 |
| | 2 | ≥99 | 78 | 78 | 76 | 99 | 75 |
| 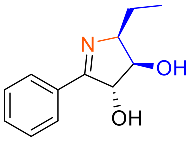 **7ci** | 0.5 | ≥99 | 12 | 11 | 78 | 24 | 19 |
| | 1 | ≥99 | 21 | 20 | 78 | 40 | **31*** |
| | 2 | ≥99 | 38 | 37 | 78 | 57 | 44 |

*The $^{1}$H NMR crude analysis of these conditions is detailed below.

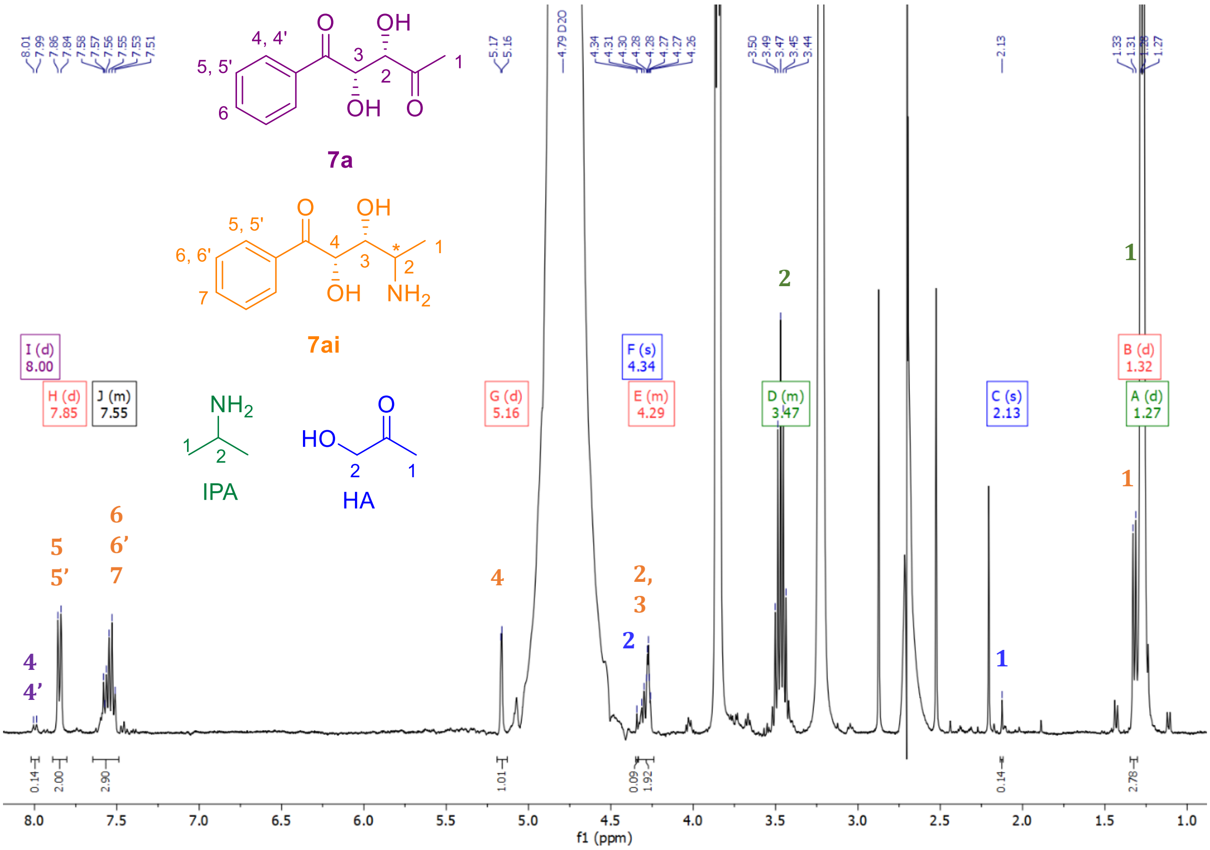

Figure S41: [1]H NMR of crude biotransformation of achiral substrates **7** and **a** to access, by telescoped reaction, to the product **7ai** catalyzed by *Ec*FSAwt lysate and pQR2191 TA.

HA: δ2.13 ppm 3H = 0.14 → 1H = 0.05
Aldol product: δ7.99 ppm 2H = 0.14 → 1H = 0.07
Product of transamination: δ7.84 ppm 2H = 2 → 1H = 1

Conversion AL = (0.07+1)/(0.07+1+0.05)*100 = 96%
Conversion TA = 1/(0.07+1)*100 = 93%

Conversion AL-TA – telescoped cascade = 0.96*0.93= 89%

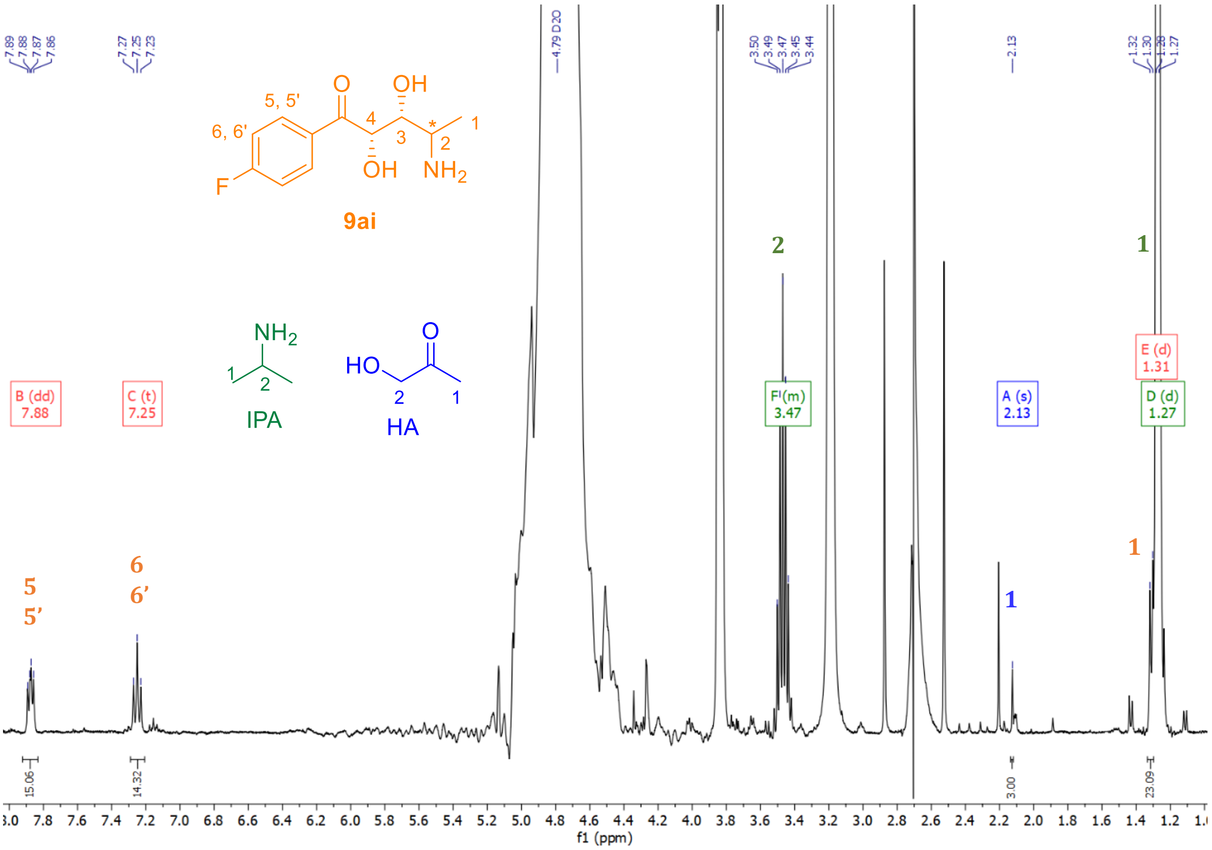


Figure S42: [1]H NMR of crude biotransformation of achiral substrates **9** and **a** to access, by telescoped reaction, to the product **9ai** catalyzed by *Ec*FSAwt lysate and pQR2191 TA.

HA: δ2.13 ppm 3H = 3 → 1H = 1
Aldol product: δ7.99 ppm – not observed – the same integration as the product of transamination **9ai** is used for the aldol conversion - δ7.84 ppm 2H = 14.32 → 1H = 7.16
Product of transamination: δ7.84 ppm 2H = 14.32 → 1H = 7.16

Conversion AL = (7.16)/(7.16+1)*100 = 89%
Conversion TA ≥ 99% as no more aldol product **9a** is observed.

Conversion AL-TA – telescoped cascade = 0.89*0.99= 88%

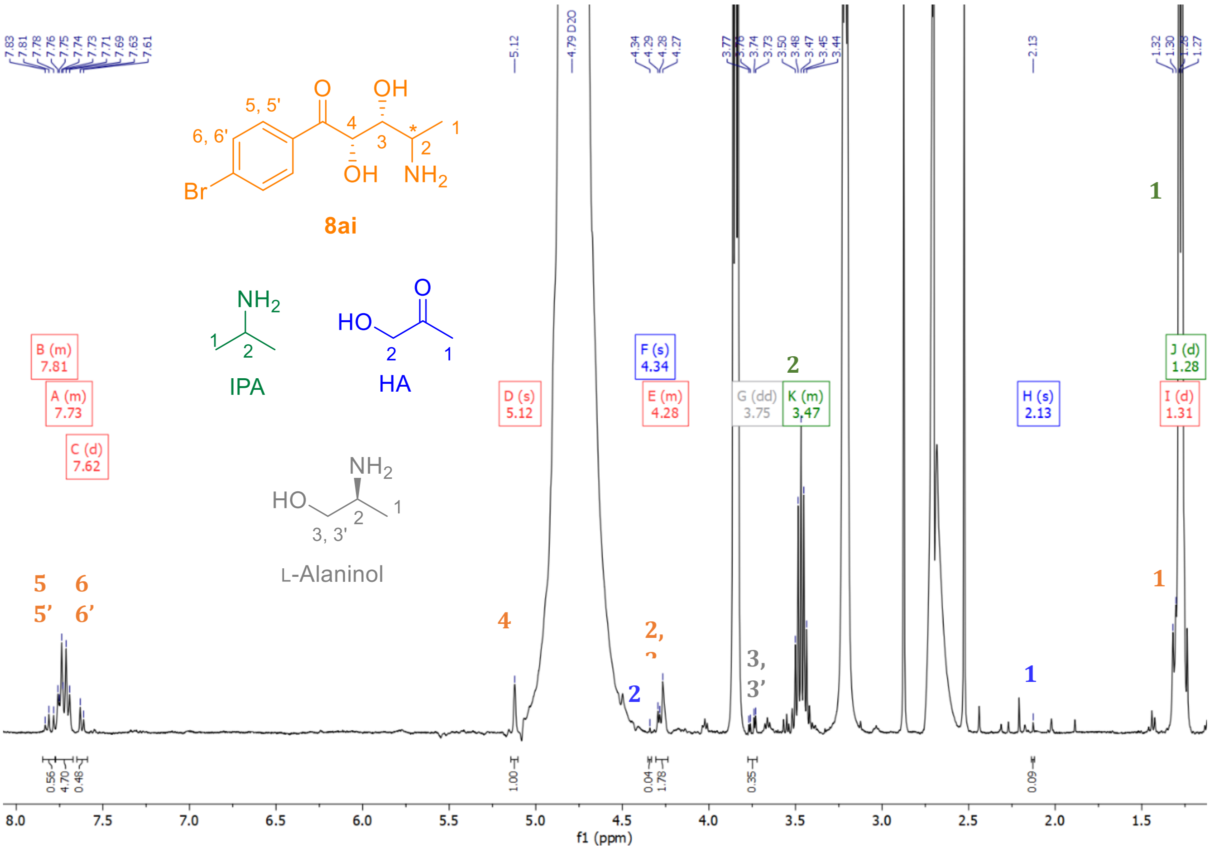


Figure S43: [1]H NMR of crude biotransformation of achiral substrates **8** and **a** to access, by sequential reaction, to the product **8ai** catalyzed by *Ec*FSA lysate wt and TA pQR2191.

HA: δ2.13 ppm 3H = 0.09 → 1H = 0.03
Aldol product: δ7.99 ppm – not observed – same integration as the product **8ai** is used for the aldol conversion – δ5.11 ppm 1H = 1
Product of transamination: δ5.11 ppm 1H = 1
L-Alaninol δ3.75 ppm 1H = 0.35

Conversion AL = 1/(1+0.03+0.35)*100 = 72%
Conversion TA ≥ 99% as no more aldol product **8a** is observed.

Conversion AL-TA – sequential cascade = 0.72*0.99= 71%

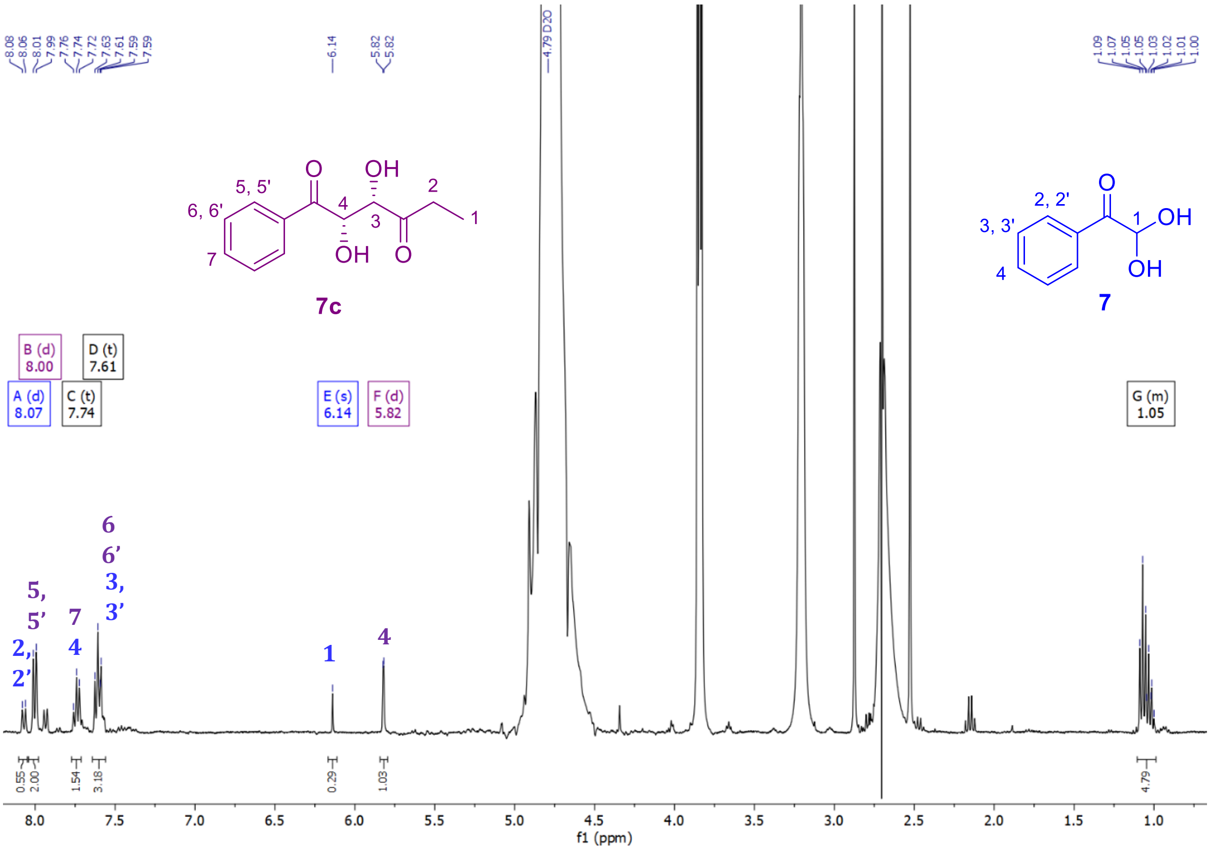


Figure S44: [1]H NMR of crude biotransformation of achiral substrates **7** and **c** to access to the aldol product **7c** catalyzed by *Ec*FSAwt lysate.

Aldehyde substrate **7**: δ8.07 ppm 2H = 0.55 → 1H = 0.275
Aldol product **7c**: δ8.00 ppm 2H = 2 → 1H = 1

Conversion AL = 1/(1+0.275)*100 = 78%

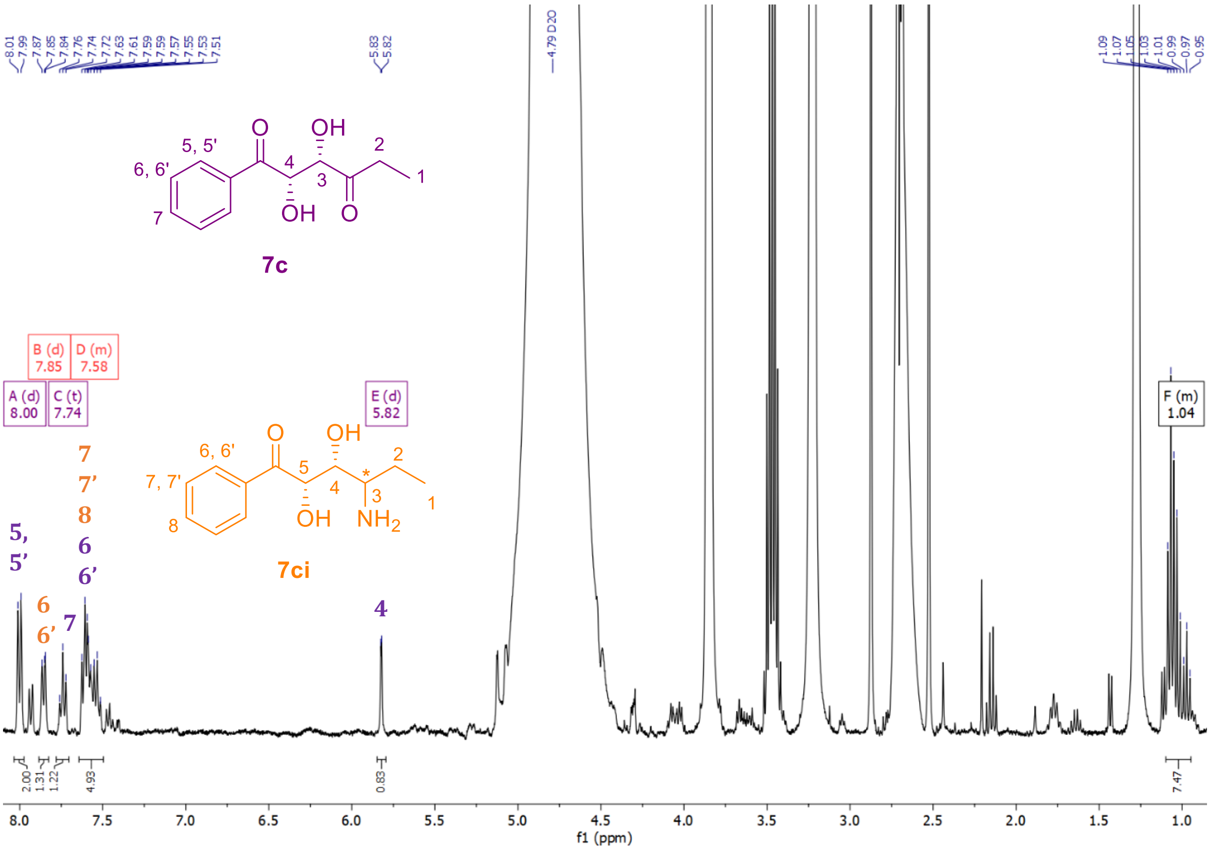


Figure S45: [1]H NMR of crude biotransformation the aldol product **7c** to access to product **7ci** catalyzed by pQR2191 TA.

Aldol product **7c**: δ8.00 ppm 2H = 2 → 1H = 1
Product of transamination **7ci**: δ7.84 ppm 2H = 1.31 → 1H = 0.66

Conversion AL = 78%
Conversion TA = 1/(1+-0.66)*100 = 40%

Conversion AL-TA – sequential cascade = 0.78*0.40 = 31%

## *Scale up and structural characterisation of transaminase products*

### *Telescoped cascade*

Reactions were carried out in 50 mL falcon tubes with a total reaction volume of 25 mL. Each reaction contained components diluted from stock solutions, with final concentrations of 20 mM ketoaldehyde substrate, 20 mM donor substrate (hydroxyacetone **a**, 1 equiv.), 200 mM IPA (10 equiv.), 1 mM PLP, 2 mg.mL$^{-1}$ lyophilized FSA cell free extract and 1 mg.mL$^{-1}$ pQR2191 TA in 100 mM triethanolamine (TEA) buffer pH 8.0 containing 8-16 v% DMSO depending on substrate solubility. Reactions were incubated at 30 $^{o}$C, 200 rpm for up to 24 h and followed by $^{1}$H NMR analysis in $D_2O$.The biotransformations were poured onto Ni-NTA agarose (5 mL) previously washed with distilled water. The column was washed with distilled water before elution of the tagged enzyme with the buffer containing 500 mM imidazole, 300 mM KCl, 50 mM KPi, pH 8 (3 CV). The ninhydrin positive fractions were pooled and then poured on a column of DOWEX®50WX8 ($H^+$ form, 20 mL). The column was washed with $H_2O$ (60 mL) and then eluted with 0.4 M $NH_3$ (100 mL) and 1 M $NH_3$ (60 mL). The ninhydrin positive fractions were pooled and concentrated under $N_2$ overnight. The solid residue was then dissolved in a minimum of n-propanol/$H_2O$:8/2 and the product was further purified by column chromatography (n-propanol/$H_2O$:8/2). The ninhydrin positive fractions were pooled and concentrated under $N_2$ overnight.

### (3R,4S,5S)-5-methyl-2-phenyl-1-pyrroline-3,4-diol **7ai**

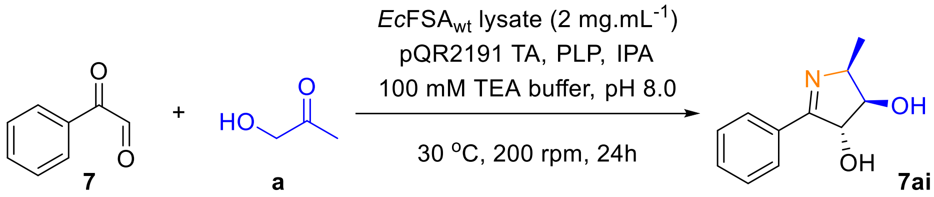


Synthesized using the telescoped procedure above. The title compound was isolated as a white solid (71 mg, 74%). δH (400 MHz, $CD_3OD$) 7.92 (d, *J* = 7.3 Hz, 2H, 2x ArC**H**CCO), 7.54–7.41 (m, 3H, ArC**H**), 5.00 (d, *J* = 2.2 Hz, 1H, C**H**OHC=N), 4.31–4.19 (m, 1H, $CH_3$C**H**N), 4.15 (dd, *J* = 5.2, 2.2 Hz, 1H, $CH_3$CHC**H**OH), 1.37 (d, *J* = 7.2 Hz, 3H, $CH_3$). δH (800 MHz, $CD_3OD$) 7.92 (d, *J* = 7.3 Hz, 2H, 2x ArC**H**CCO), 7.50 – 7.47 (m, 1H, ArC**H**), 7.47–7.43 (m, 2H, ArC**H**CHC), 5.00 (d, *J* = 2.2 Hz, 1H, C**H**OHC=N), 4.25 (qd, *J* = 7.2, 5.2 Hz, 1H, $CH_3$C**H**N), 4.15 (dd, *J* = 5.2, 2.2 Hz, 1H, $CH_3$CHC**H**OH), 1.37 (d, *J* = 7.2 Hz, 3H, $CH_3$). δC (101 MHz, $CD_3OD$) 175.06 (ArCN), 134.14 (Ar**C**CN), 132.06 (Ar**C**HCHCHCCN), 129.61 (Ar**C**HCHCCN), 129.26 (Ar**C**HCCN), 83.60 (**C**HOHCN), 79.47 (**C**HOHCHCH$_3$), 69.08 (**C**HCH$_3$), 14.55 (**C**H$_3$). HRMS(APCI) *m/z* calculated for $C_{11}H_{14}NO_2$ [M+H]$^+$ 192.1019, observed 192.1019 [M+H]$^+$.

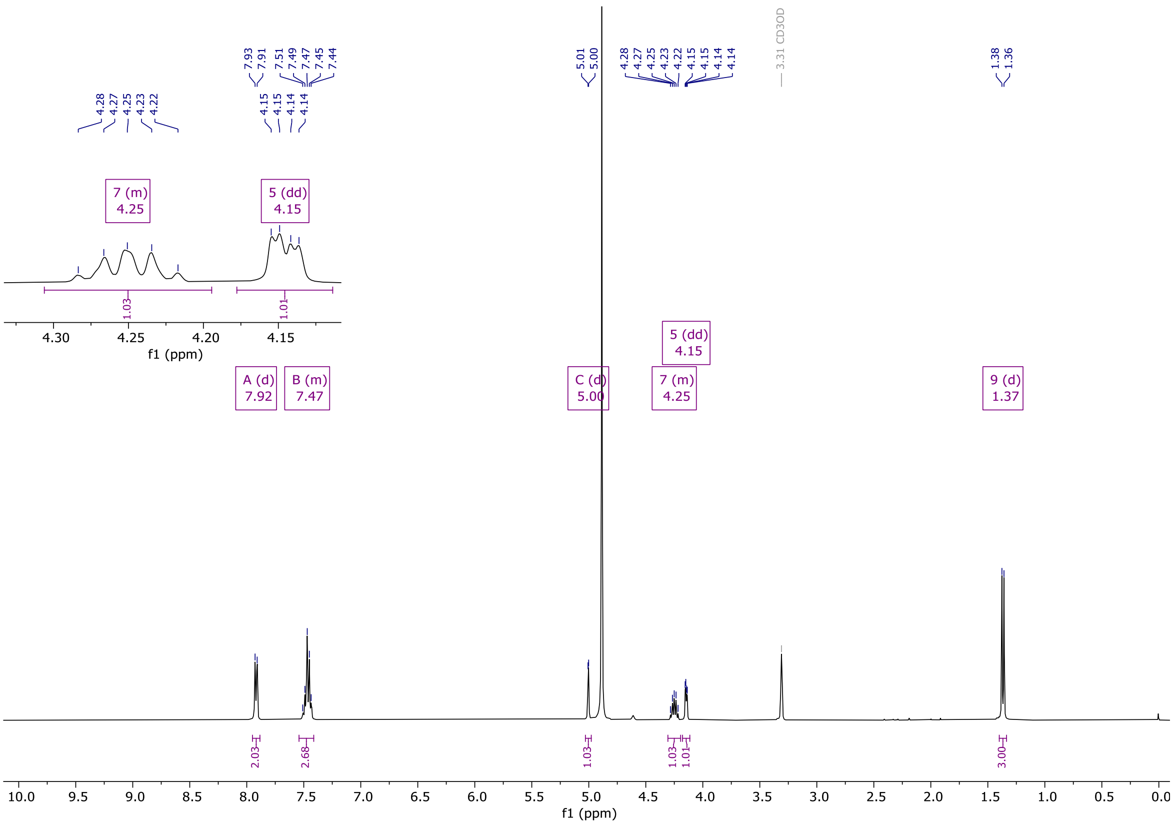

Figure S46: $^{1}$H NMR (400 MHz) of biocatalytically synthesized pyrroline **7ai**. Expansion pane of 4.10–4.35 ppm showing the C**H**$CH_3$ and $CH_3$CHC**H**OH signals.

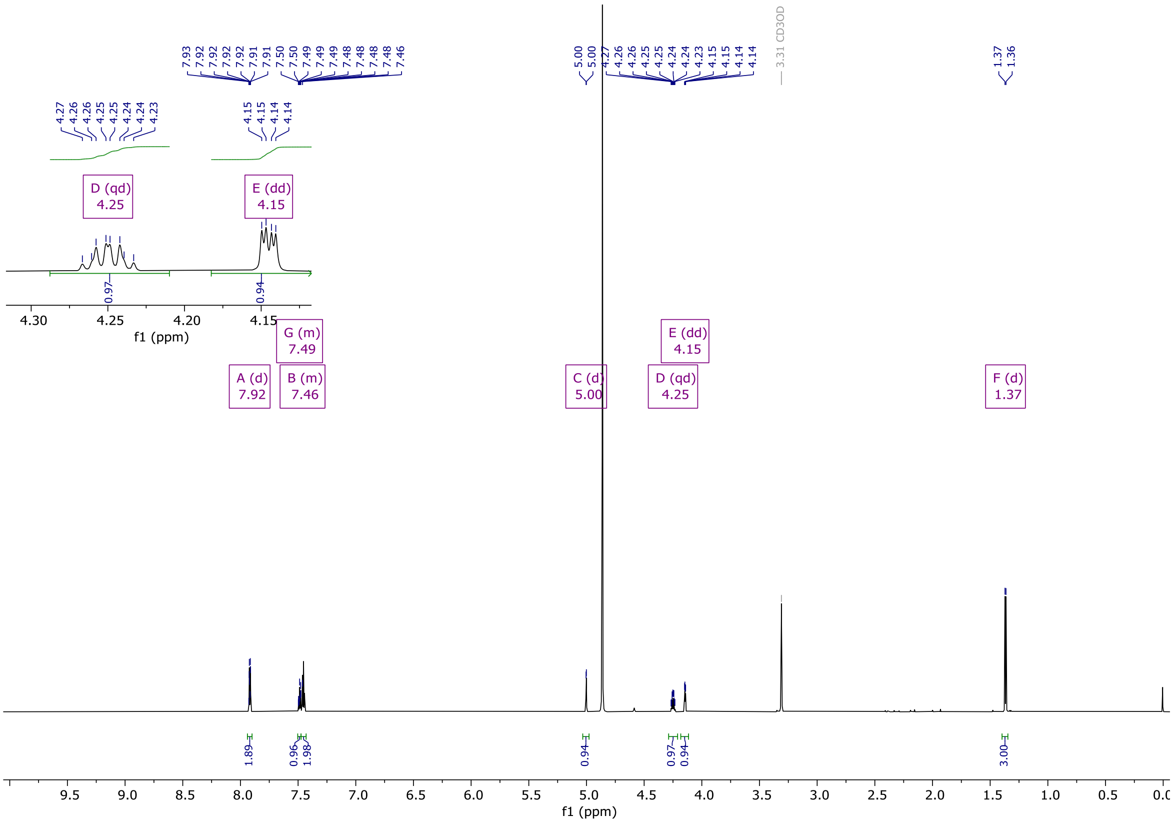

Figure S47: $^{1}$H NMR (800 MHz) of biocatalytically synthesized pyrroline **7ai**. Expansion pane of 4.10–4.35 ppm showing the C**H**$CH_3$ and $CH_3$CHC**H**OH signals.

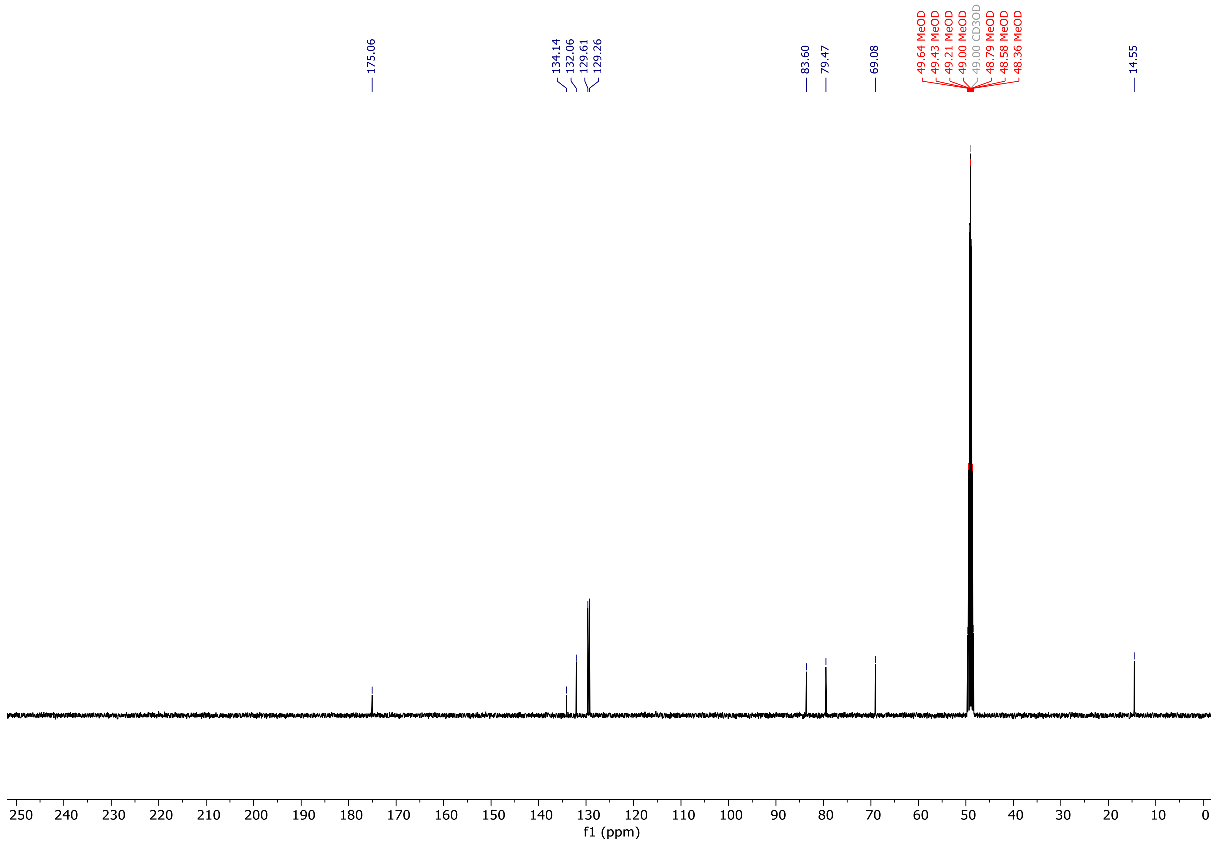


Figure S48: $^{13}C$ NMR of biocatalytically synthesized pyrroline **7ai**.

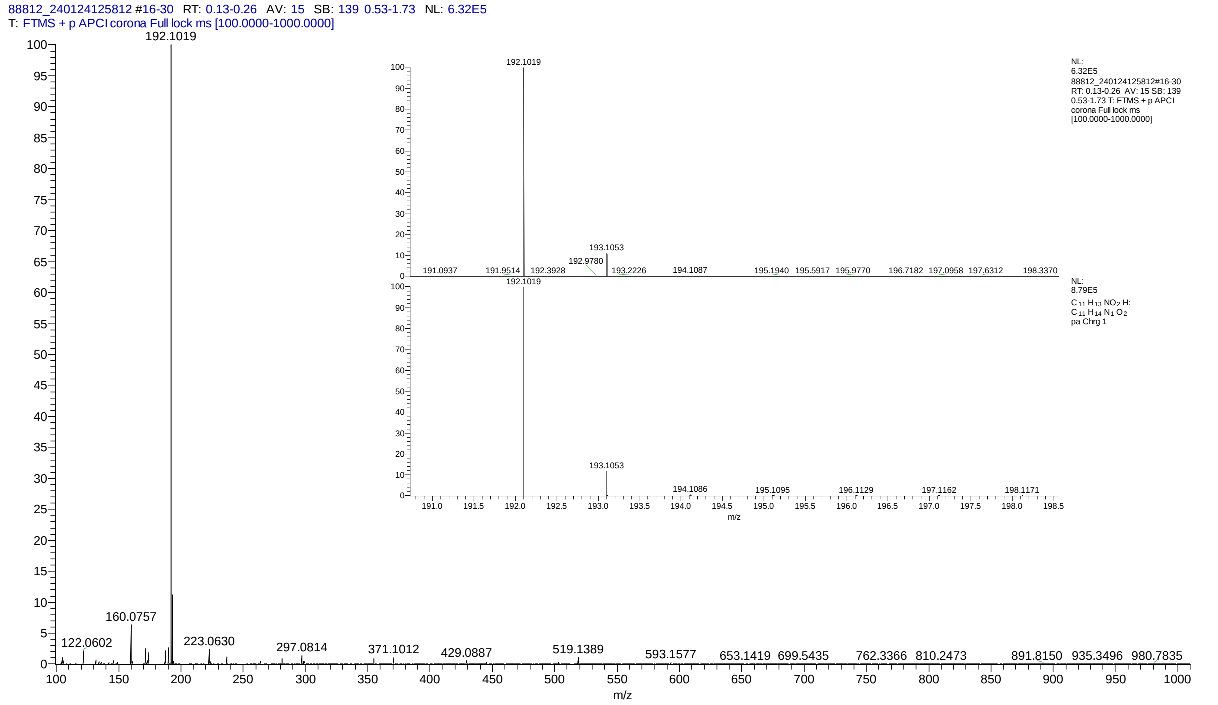


Figure S49: HRMS (APCI) of biocatalytically synthesized pyrroline **7ai**. Expansion pane showing region 190 – 199 *m/z*.

### *Sequential cascade*

Aldol reactions were carried out in 50 mL falcon tubes with a total reaction volume of 20 mL. Each reaction contained components diluted from stock solutions, with final concentrations of 25 mM ketoaldehyde substrate, 25 mM donor substrate (hydroxyacetone, 1 equiv.) and 2 mg.mL$^{-1}$ lyophilized FSA cell free extract in 100 mM triethanolamine (TEA) buffer pH 8.0 containing 10-20 v% DMSO depending on substrate solubility. Reactions were incubated at 30 $^{o}$C, 200 rpm for up to 24 h and followed by $^{1}$H NMR analysis in $D_2O$. Transamination reactions were carried out in 50 mL falcon tubes containing the aldol reaction with a final volume of 25 mL. At each aldol reaction are added components diluted from stock solution with final concentration of 200 mM IPA (10 eq.), 1 mM PLP and 1 mg.mL$^{-1}$ purified pQR2191 TA in 100 mM TEA buffer pH 8.0. Reactions were incubated at 30 °C, 200 rpm for 24h and followed by $^{1}$H NMR analysis in $D_2O$. The biotransformations were poured onto Ni-NTA agarose (5 mL) previously washed with distilled water. The column was washed with distilled water before elution of the tagged enzyme with the buffer containing 500 mM imidazole, 300 mM KCl, 50 mM KPi, pH 8 (3 CV). The ninhydrin positive fractions were pooled and then poured on a column of DOWEX®50WX8 ($H^+$ form, 20 mL). The column was washed with $H_2O$ (60 mL) and then eluted with 0.4 M $NH_3$ (100 mL) and 1 M $NH_3$ (60 mL). The ninhydrin positive fractions were pooled and concentrated under $N_2$ overnight. The solid residue was then dissolved in a minimum of n-propanol/$H_2O$:8/2 and the product was further purified by column chromatography (n-propanol/$H_2O$:8/2). The ninhydrin positive fractions were pooled and concentrated under $N_2$ overnight.

### (3R,4S,5S)-5-methyl-2-(4-fluorophenyl)-1-pyrroline-3,4-diol **9ai**

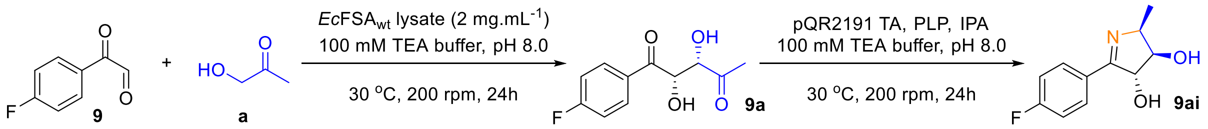


Synthesized using the sequential cascade procedure above. The title compound was isolated as a yellow solid (55 mg, 53% yield). δH (400 MHz, $CD_3OD$) 7.96 (dd, *J* = 8.6, 5.6 Hz, 2H, 2x ArC**H**CCO), 7.19 (dd, *J* = 8.9, 8.6 Hz, 2H, ArC**H**CF), 4.97 (d, *J* = 2.3 Hz, 1H, C**H**OHC=N), 4.29 – 4.18 (m, 1H, $CH_3$C**H**N), 4.14 (dd, *J* = 5.4, 2.3 Hz, 1H, $CH_3$CHC**H**OH), 1.36 (d, *J* = 7.1 Hz, 3H, C**H**$_3$). δH (800 MHz, $CD_3OD$) 7.99 – 7.94 (m, 2H, 2x ArC**H**CCO), 7.21 – 7.17 (m, 2H, ArC**H**CF), 4.97 (d, *J* = 2.3 Hz, 1H, C**H**OHC=N), 4.24 (qd, *J* = 7.2, 5.4 Hz, 1H, $CH_3$C**H**N), 4.14 (dd, *J* = 5.4, 2.3 Hz, 1H, $CH_3$CHC**H**OH), 1.36 (d, *J* = 7.2 Hz, 3H, C**H**$_3$). δF (376 MHz, $CD_3OD$) −111.34. δC (101 MHz, $CD_3OD$) 173.88 (Ar**C**N), 165.85 (d, $^{1}J_{CF}$ = 250.0 Hz, Ar**C**F), 131.64 (d, $^{3}J_{CF}$ = 8.8 Hz, Ar**C**HCCN), 130.62 (d, *J* = 3.1 Hz, Ar**C**CN), 116.50 (d, $^{2}J_{CF}$ = 22.0 Hz, Ar**C**HCF), 83.66 (**C**HOHC=N), 79.54 (**C**HOHCHCH$_3$), 69.08 (**C**HCH$_3$), 14.56 (**C**H$_3$). HRMS(ESI) *m/z* calculated for $C_{11}H_{12}FNO_2$ [M+H]$^+$ 209.0852, observed 210.1012 [M+H]$^+$.

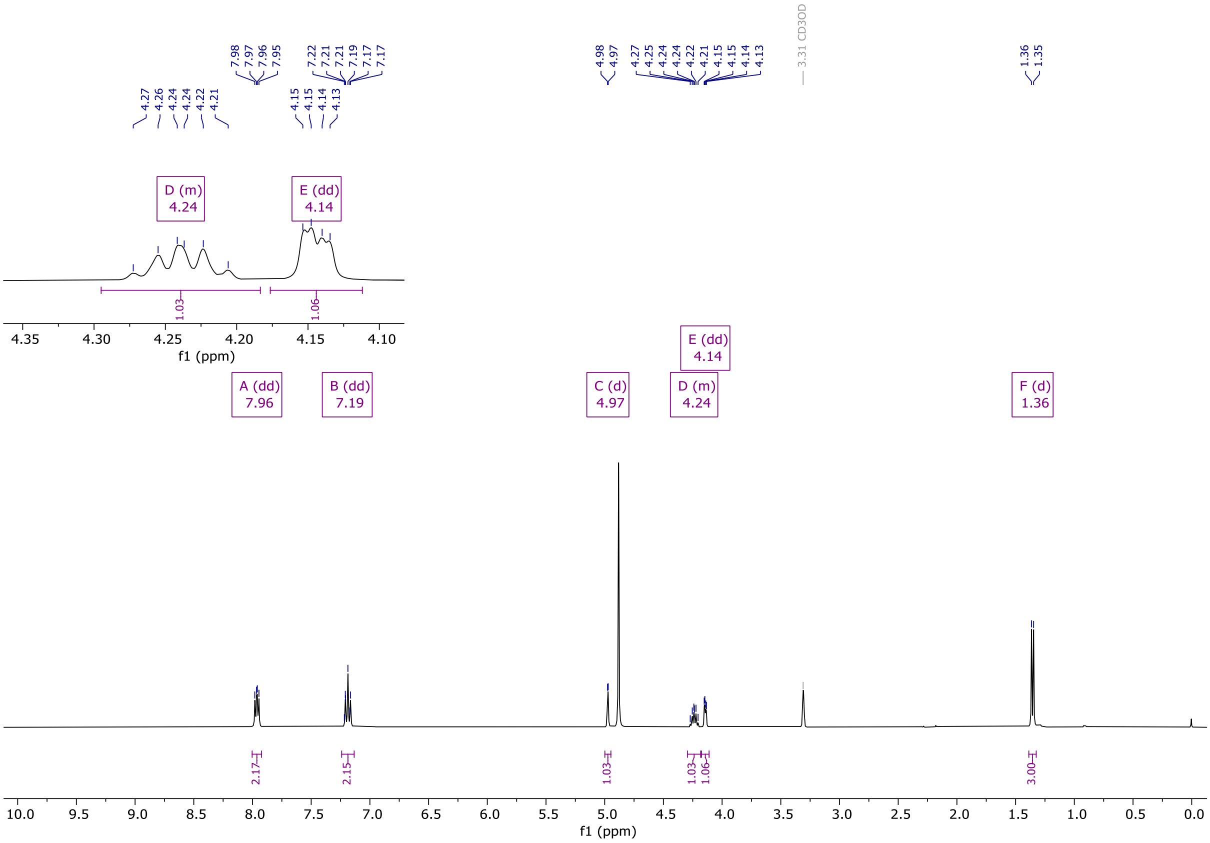


Figure S50: $^{1}H$ NMR (400 MHz) of biocatalytically synthesized pyrroline **9ai**. Expansion pane of 4.10–4.35 ppm showing the C**H**$CH_3$ and $CH_3$CHC**H**OH signals.

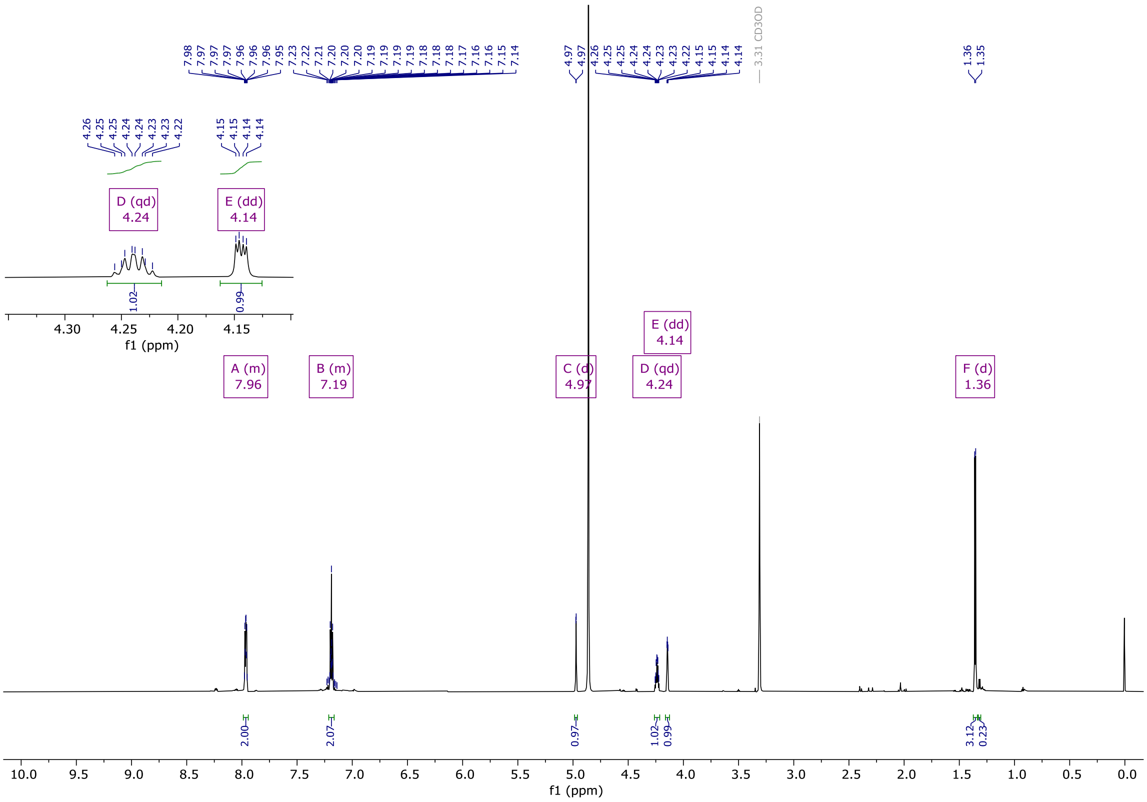


Figure S51: $^{1}H$ NMR (800 MHz) of biocatalytically synthesized pyrroline **9ai**. Expansion pane of 4.10–4.35 ppm showing the C**H**$CH_3$ and $CH_3$CHC**H**OH signals.

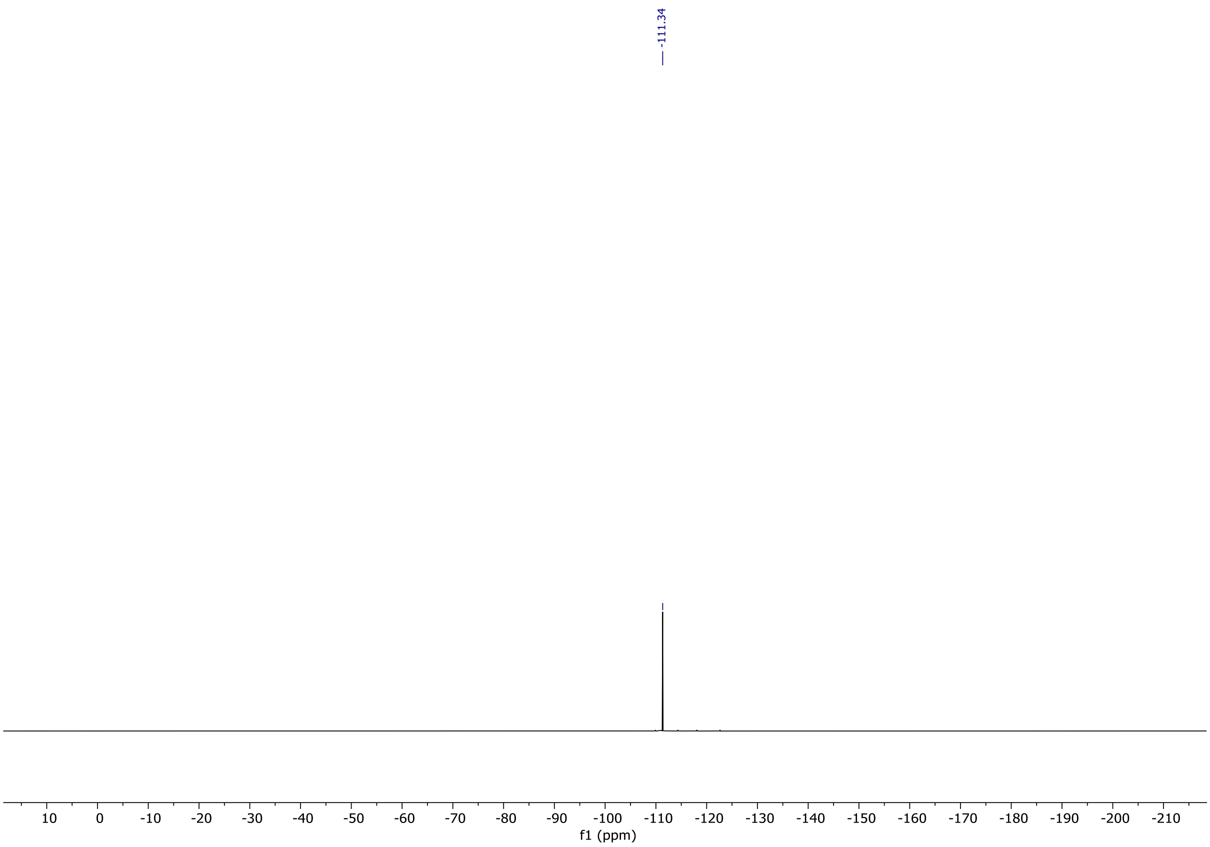


Figure S52: $^{19}F$ NMR of biocatalytically synthesized pyrroline **9ai**.

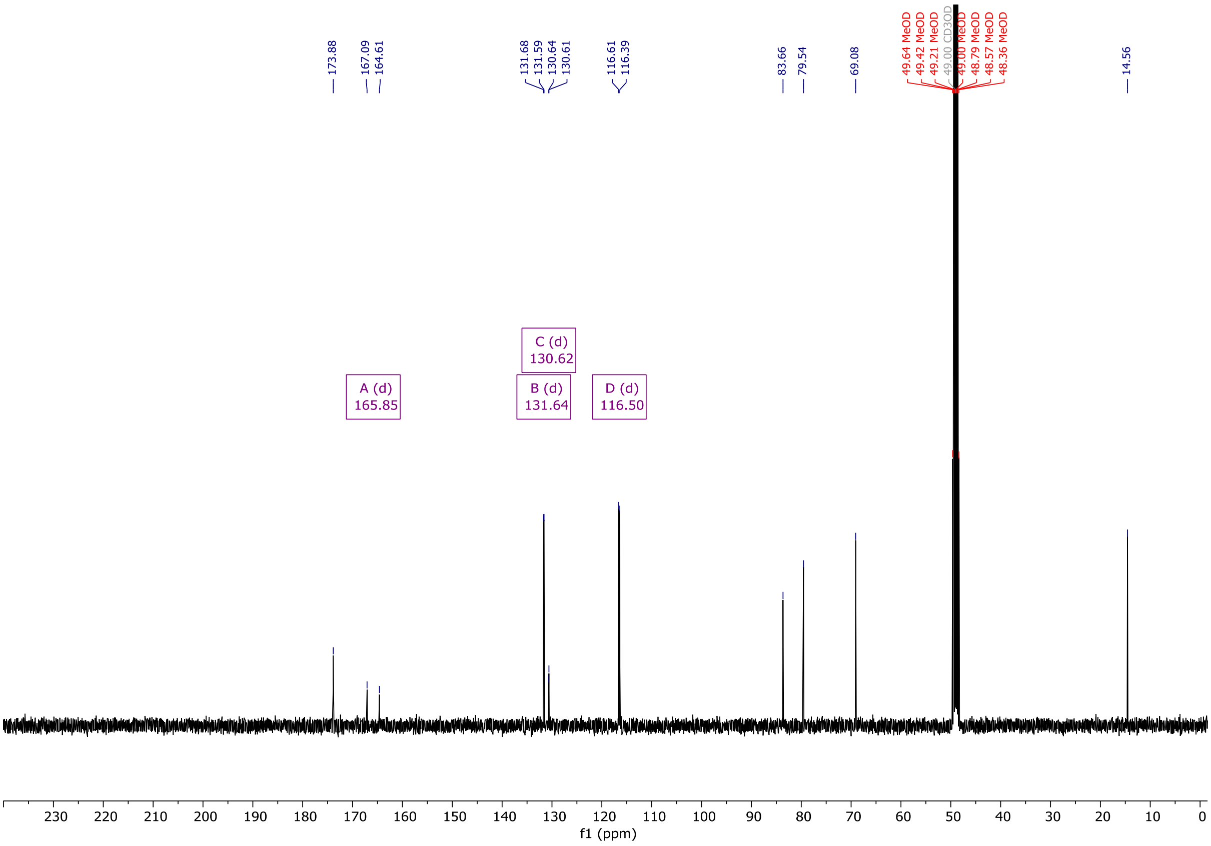


Figure S53: $^{13}C$ NMR of biocatalytically synthesized pyrroline **9ai**.

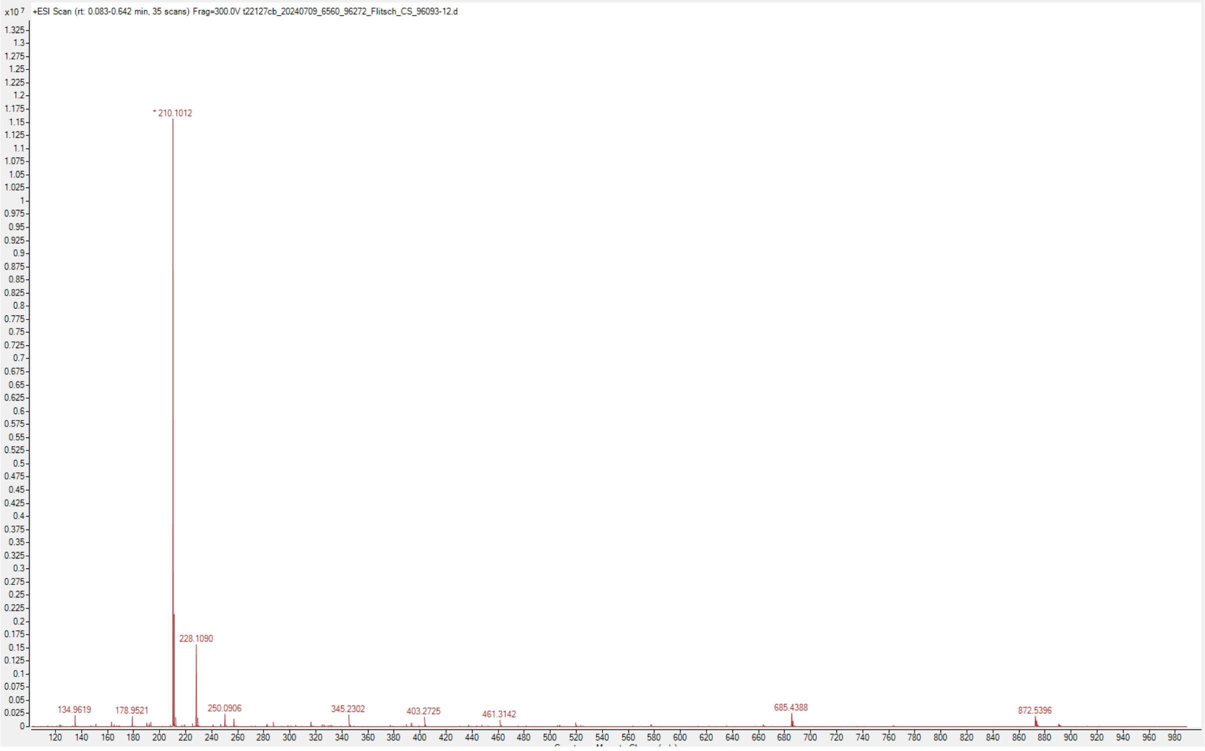


Figure S54: HRMS (ESI) of biocatalytically synthesized pyrroline **9ai**.

## (3R,4S,5S)-5-methyl-2-(4-bromophenyl)-1-pyrroline-3,4-diol **8ai**

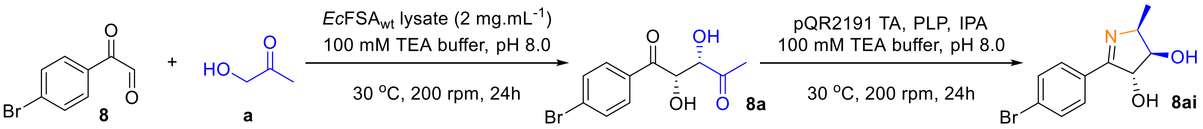


Synthesized using the sequential cascade with a modified purification procedure. The biotransformations were poured on a column of DOWEX®50WX8 ($H^+$ form, 20 mL). The column was washed with $H_2O$ (60 mL) and then eluted with 0.4 M $NH_3$ (100 mL) and 1 M $NH_3$ (60 mL). The ninhydrin positive fractions were pooled and concentrated under $N_2$ overnight. The residue was dissolved in a minimum of distilled water and then poured on a column of DOWEX®50WX8 ($NH_4^+$ form, 20 mL). The column was washed with $H_2O$ (60 mL) and then eluted with a gradient of $NH_3$ (0.25 M, 0.5 M and 0.75 M, 50 mL each concentration). The ninhydrin positive fractions were pooled and concentrated under $N_2$ overnight. The solid residue was then dissolved in a minimum of n-propanol/$H_2O$:8/2 and the product was further purified by column chromatography (n-propanol/$H_2O$:8/2). The ninhydrin positive fractions were pooled and concentrated under $N_2$ overnight. The title compound was isolated as a white solid (40 mg, 30%). δH (400 MHz, $CD_3OD$) 7.85 (d, *J* = 7.2 Hz, 2H, 2x ArC**H**CCO), 7.65 (d, *J* = 8.5 Hz, 2H, ArC**H**CF), 5.01 – 4.96 (m, 1H, C**H**OHC=N), 4.31 – 4.20 (m, 1H, $CH_3$C**H**N), 4.19 – 4.13 (m, 1H, $CH_3$CHC**H**OH), 1.38 (d, *J* = 7.2 Hz, 3H, C**H**$_3$).δC (101 MHz, $CD_3OD$) 174.09 (Ar**C**N), 133.27 (Ar**C**Br), 132.85 (Ar**C**HCCN), 130.96 (Ar**C**HCBr), 126.40 (Ar**C**CN), 83.57(**C**HOHCN), 79.53 (**C**HOHCHCH$_3$), 69.29 (**C**HCH$_3$), 14.50 (**C**H$_3$). HRMS(ESI) *m/z* calculated for $C_{11}H_{12}BrNO_2$ $[M+H]^+$ 270.0124 and 272.0104, observed 270.0087 and 272.0097 $[M+H]^+$.

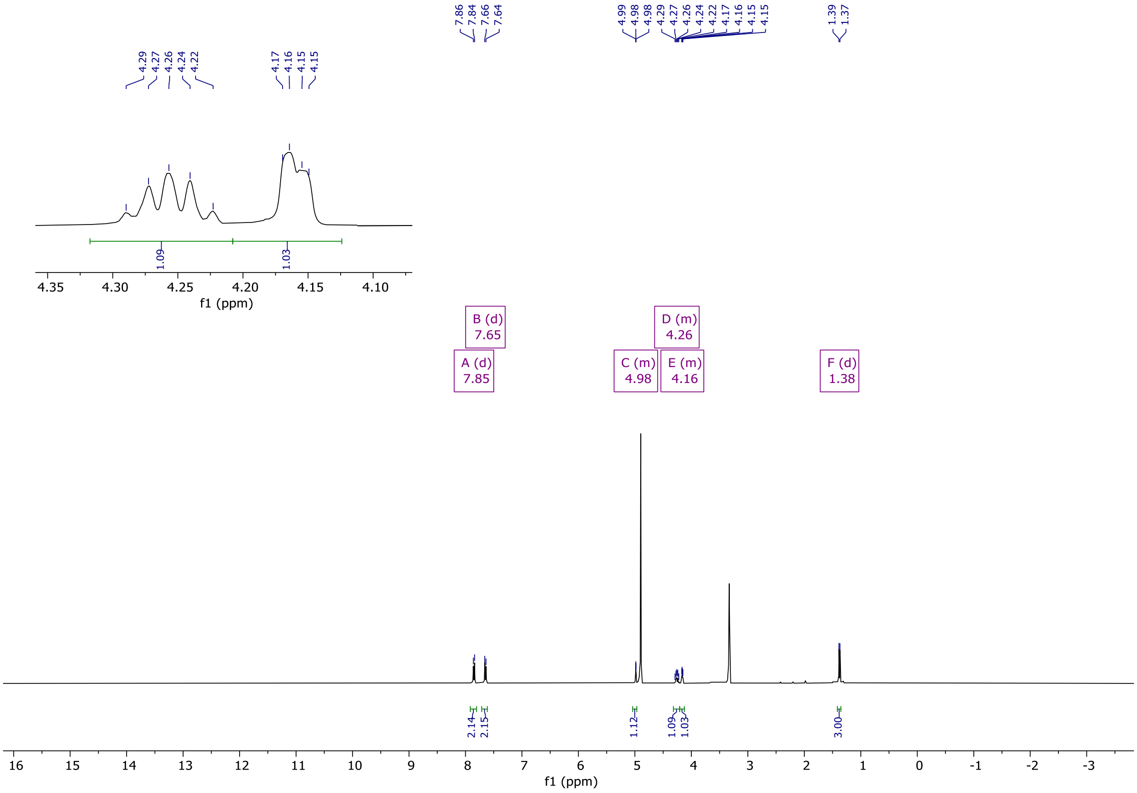


Figure S55: $^{1}H$ NMR (400 MHz) of biocatalytically synthesized pyrroline **8ai**. Expansion pane of 4.10–4.35 ppm showing the C**H**$CH_3$ and $CH_3$CHC**H**OH signals.

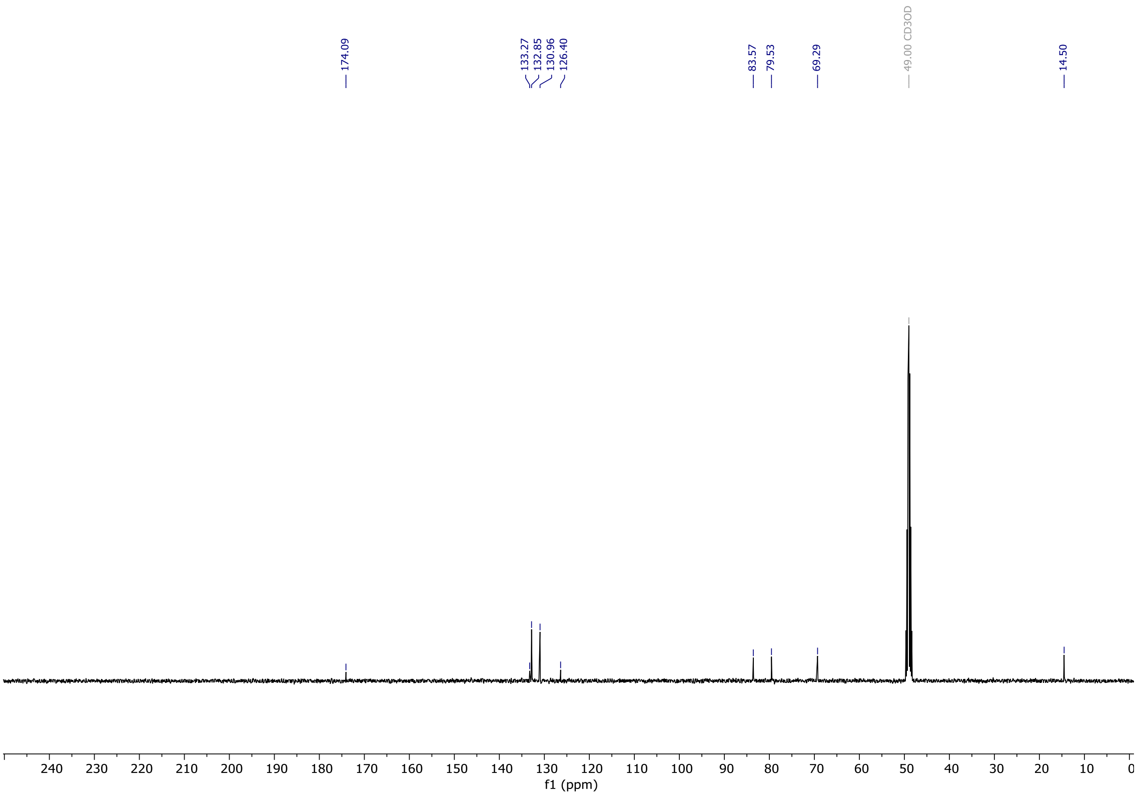


Figure S56: $^{13}C$ NMR of biocatalytically synthesized pyrroline **8ai**.

*Determination of transaminase product stereochemistry*

The stereochemistry of transaminase products was determined using $^{1}$H-NMR coupling constants based on comparison to known compounds and evaluation of H-C-C-H dihedral angles across the pyrroline ring system.

Table S7: Potential diastereomeric transaminase products (3*R*,4*R*,5*R*)- or (3*R*,4*R*,5*S*)-**7ai** and reference literature compound (3*S*,4*R*,5*S*)-**7ai. Top:** Coupling constants for key protons on the pyrroline ring. Coupling constants for the transaminase product are as observed in the 400 and 800 MHz spectra reported in this work, and taken from the literature for the reference compound.[8] **Bottom**: Dihedral angles between the coupled protons along the C4-C5 and C3-C4 bonds. Values are the average of three replicate calculations (± standard deviation).

| Coupling constants | Transaminase product (one of two diastereomers) | | Reference compound |
|---|---|---|---|
| | 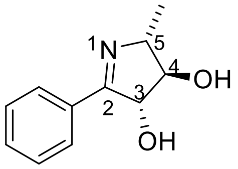 (3*R*,4*R*,5*R*)-**7ai** | 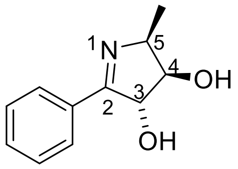 (3*R*,4*R*,5*S*)-**7ai** | 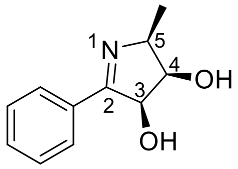 (3*S*,4*R*,5*S*)-**7ai** |
| $^{3}J_{H_3-H_4}$ / Hz | 2.3 | | 5.5 |
| $^{3}J_{H_4-H_5}$ / Hz | 5.2 | | 5.5 |
| $^{3}J_{H_5-H_{Me}}$ / Hz | 7.2 | | 7.1 |
| Dihedral angle / ° | | | |
| $\theta_{H-C4-C5-H}$ | −98.5 (± 0.036) | 36.2 (± 0.022) | 40.7 (± 0.011) |
| $\theta_{H-C3-C4-H}$ | 93.5 (± 0.034) | 89.9 (± 0.027) | −31.6 (± 0.012) |

The difference in magnitude of the $^{3}J_{H_3-H_4}$ coupling constants (Table S7) of the reference compound (3*S*,4*R*,5*S*)-**7ai** (5.5 Hz, C3 and C4 substituents *cis*) and the transaminase product (2.3 Hz) shows a *trans* orientation of substituents on C3 and C4 in the transaminase product (derived from the stereochemistry formed in the aldolase catalyzed step). Conversely, the similarity of the $^{3}J_{H_4-H_5}$ constants for the reference compound and transaminase product (5.5 and 5.2 Hz, respectively) suggests a *cis* orientation of substituents on C4 and C5 (as found in the reference compound).

The dihedral angles between the coupled protons across the C4-C5 and C3-C4 bonds of the pyrroline ring ($\theta_{H-C4-C5-H}$ and $\theta_{H-C3-C4-H}$, respectively, Table S7) further support this assignment. Chem3D models of the respective compounds were generated, energy minimized using MM2 and the key dihedral angles were measured. A large difference in the magnitude of dihedral angles for *cis* and *trans* oriented substituents on C3, C4 and C5 was observed. In the reference compound (3*S*,4*R*,5*S*)-**7ai**, the dihedral angles are 40.7° and −31.6° for the H-C4-C5-H and H-C3-C4-H planes, respectively. In comparison, the dihedral angles $\theta_{H-C3-C4-H}$ for the two possible transaminase products (3*R*,4*R*,5*R*)-**7ai** and (3*R*,4*R*,5*S*)-**7ai** (93.5° and 89.9°) are much larger, and the accompanying observed $^{3}J_{H_3-H_4}$ is much smaller (2.3 Hz vs 5.5 Hz). The dihedral angles for H-C4-C5-H also corroborate the stereochemical assignment by coupling constants as $\theta_{H-C4-C5-H}$ for the reference compound (3*S*,4*R*,5*S*)-**7ai** and (3*R*,4*R*,5*S*)-**7ai** are similar (40.7° and 36.2°, respectively), whereas $\theta_{H-C4-C5-H}$ for (3*R*,4*R*,5*R*)-**7ai** is much larger (−98.5 °) and so a larger difference in $^{3}J_{H_4-H_5}$ would be expected (observed coupling constant = 5.2 Hz, reference compound = 5.5 Hz).

Together, these data support the formation of the (*S*) stereochemistry at the new amine centre in accordance with previous work with this transaminase and the overall stereochemistry of the transaminase product is (3*R*,4*R*,5*S*).[5,9]

## *Reduction of transaminase derived pyrrolines*

### *Chemical reduction of Ph,Me substituted pyrroline **7ai***

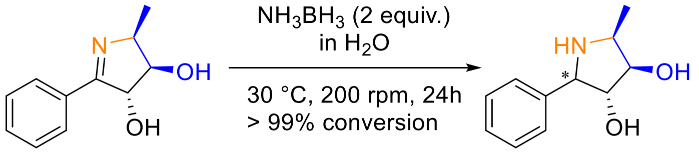


20 mM **7ai** 6:4 d.r. **7aii**

(3*R*,4*S*,5*S*)-5-methyl-2-phenyl-1-pyrrolidine-3,4-diol (4 mg, 0.02 mmol) isolated from a transaminase biotransformation was dissolved in water or methanol (0.952 mL) and $NH_3BH_3$ (2 equiv., 38 µL of a 1M stock) was added. The reactions were incubated at 30 °C for 24 hours and monitored by UPLC-QDa and NMR. Aliquots for UPLC-QDa were taken and diluted 10-fold (i.e. 30 µL reaction mix diluted to 300 µL total volume) with 9:1 water/methanol, centrifuged (13,000 rpm, 5 mins) and decanted into 0.45 µm filter vials. At 24 hours the reaction mixture was concentrated *en vacuo* and the residue dissolved in $D_2O$ for NMR analysis. Full substrate depletion was observed by NMR in the reaction performed in water and two diastereomeric products were observed in a 6:4 ratio. A new peak was observed on UPLC-QDa analysis with *m/z* 194 corresponding to the pyrrolidine product $[M+H]^+$ molecular ion. δH (400 MHz, $D_2O$) 7.54–7.30 (m, 10H, Ar, both diastereomers), 4.85 (d, *J* = 3.8 Hz, 1H, C**H**Ph, minor diastereomer), 4.40 (dd, *J* = 3.8, 1.6 Hz, 1H, PhCHC**H**OH, minor diastereomer), 4.33 – 4.27 (m, 1H, $CH_3$CHC**H**OH, minor diastereomer), 4.25 (dd, *J* = 6.0, 2.5 Hz, 1H, PhCHC**H**OH, major diastereomer), 4.14 (dd, *J* = 4.9, 2.5 Hz, 1H, $CH_3$CHC**H**OH, major diastereomer), 4.10 – 4.06 (m, 1H, C**H**$CH_3$, minor diastereomer), 4.05 (d, *J* = 6.0 Hz, 1H, C**H**Ph, major diastereomer), 3.63–3.51 (m, 1H, C**H**$CH_3$, major diastereomer) 1.38 (d, *J* = 6.9 Hz, 3H, C**H**$_3$, minor diastereomer), 1.27 (d, *J* = 6.8 Hz, 3H, C**H**$_3$, major diastereomer). Impurity carried through from substrate: 3.75 (q, *J* = 7.2 Hz, 1H), 1.45 (d, *J* = 7.2 Hz, 3H). δC (101 MHz, $D_2O$) 129.01 (Ar), 128.75 (Ar), 128.62 (Ar), 127.88 (Ar), 127.63 (Ar), 83.08 (PhCH**C**HOH, major diastereomer), 78.30 ($CH_3$CH**C**HOH, major diastereomer), 77.37 (PhCH**C**HOH, minor diastereomer), 76.57 ($CH_3$CH**C**HOH, minor diastereomer), 68.13 (**C**HPh, major diastereomer), 63.85 (**C**HPh, minor diastereomer), 56.92 (**C**H$CH_3$, both diastereomers), 12.02 (**C**$H_3$, major diastereomer), 11.58 (**C**$H_3$, minor diastereomer).

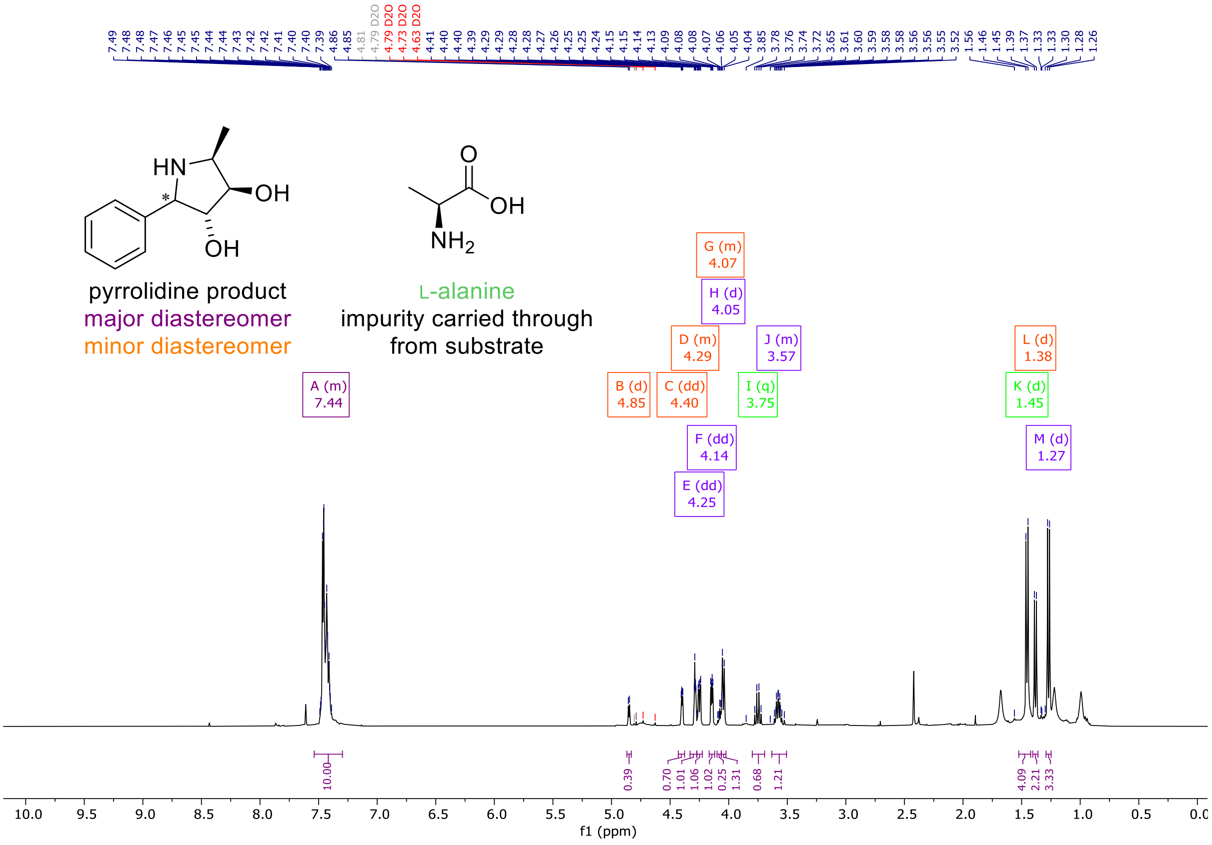


Figure S57: [1]H-NMR spectrum of the chemical reduction reaction using **7ai** as a substrate.

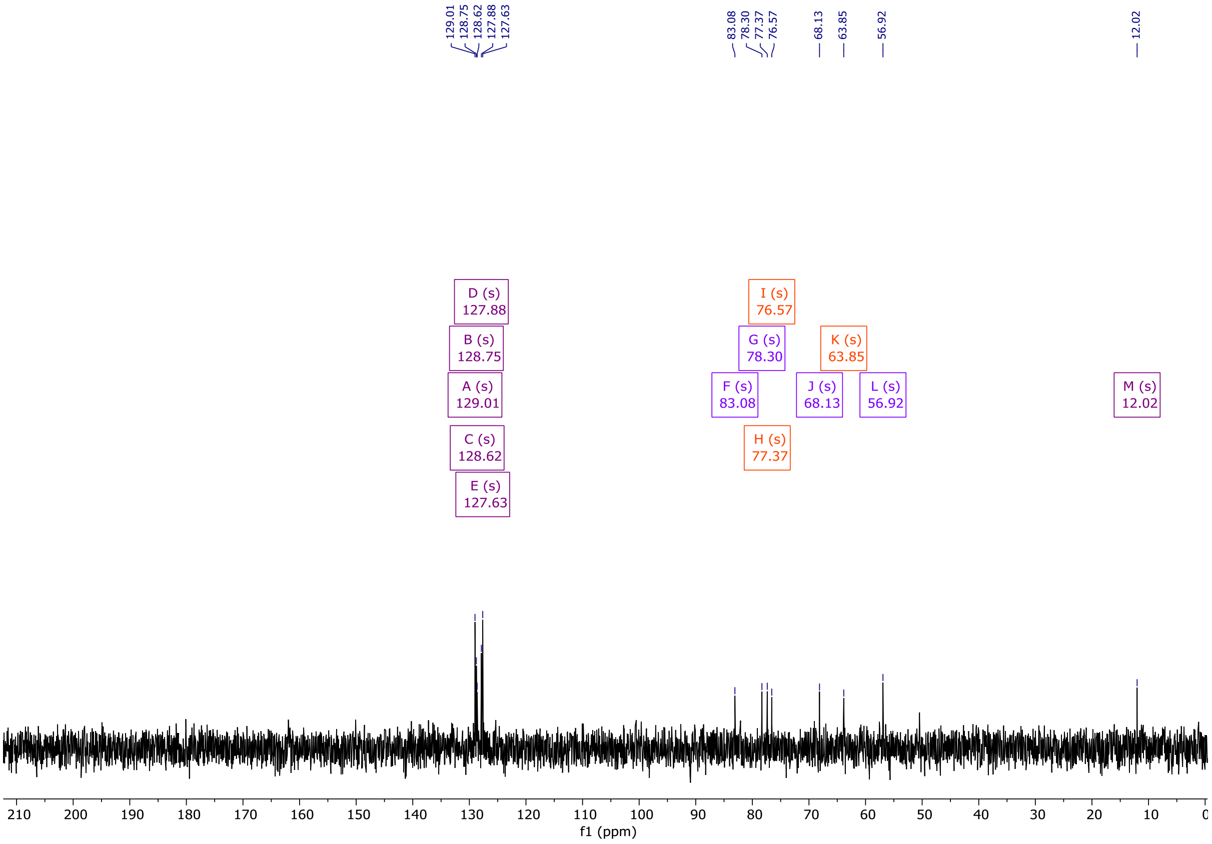


Figure S58: [13]C-NMR spectrum of the chemical reduction reaction using **7ai** as a substrate.

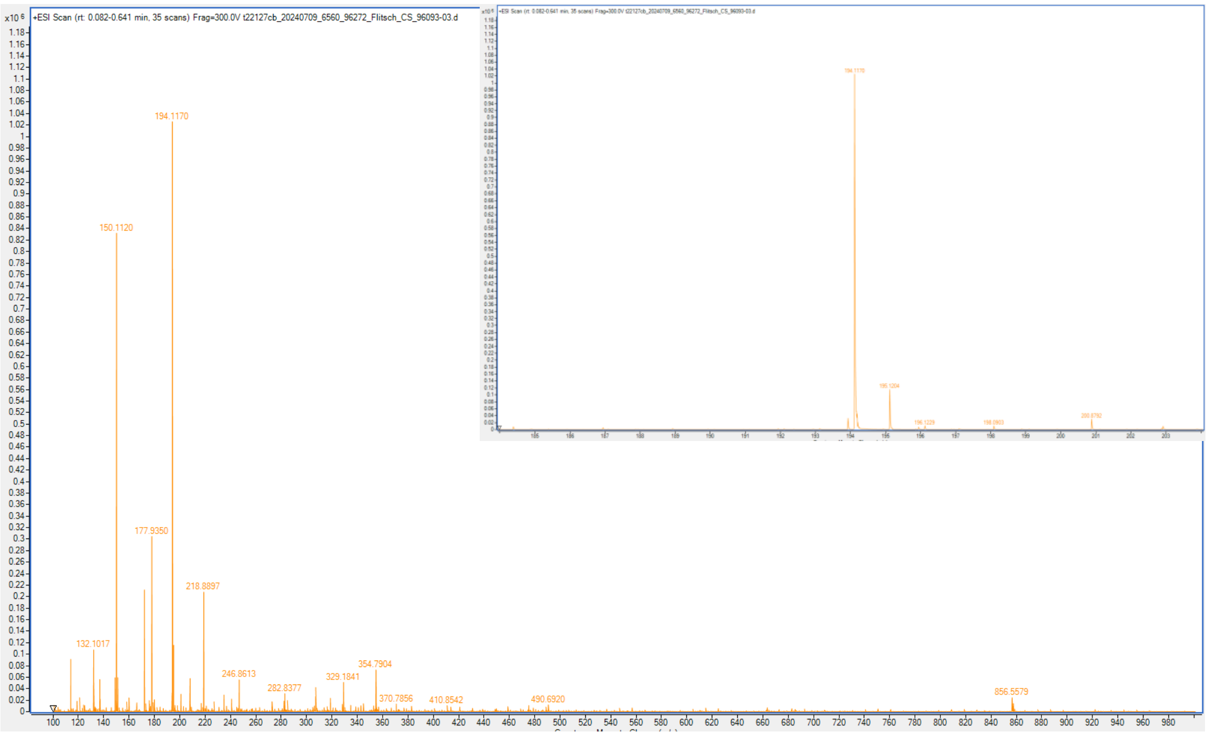


Figure S59**:** HRMS of the chemical reduction reaction using **7ai** as a substrate showing *m/z* 194.1170 corresponding to the pyrrolidine product **7aii**.

### *Screening of reductases for IRED activity against transaminase derived pyrroline **7ai***

A small panel of diverse NAD(P)H dependent reductase enzymes were selected based on previously reported similarity in substrate scope for reduction of pyrrolines or hydroxylated *N*-heterocycles. Amongst this panel were metagenomic IREDs, the well-studied fungal reductive aminase *Ad*RedAm, two reductases with identified *in vitro* activity towards piperidine iminosugars and three biosynthetic enzymes reported to catalyze imine reduction in the biosynthesis of anisomycin (Figure S60).

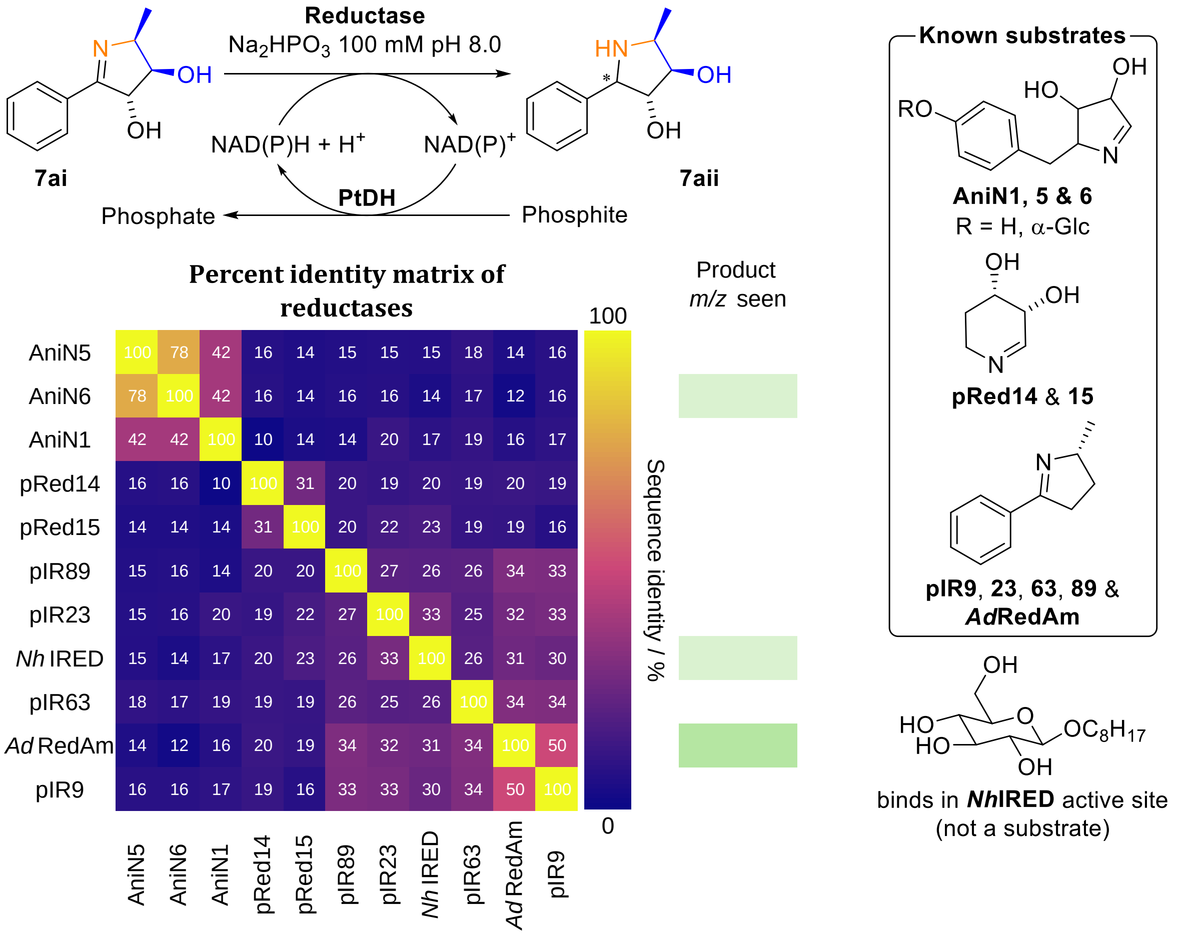


Figure S60**:** The enzymatic reduction reaction of pyrroline **7ai** used to screen for IRED activity. A percentage identity matrix of the screened reductases to show the typically low identity amongst these enzymes and their relevant known substrates. Initial qualitative MS based screening suggests AniN6, *Nh*IRED and *Ad*RedAm can reduce the pyrroline due to appearance of the pyrrolidine *m/z* after 24h biotransformations.

The reductases were initially screened for activity towards the reduction of **7ai** by QDa mass spectrometry. Reductases which gave a positive hit for presence of the pyrrolidine product $[M+H]^+$ species (AniN6, *Nh*IRED and *Ad*RedAm) were taken forward for 500 µL scale biotransformations which were monitored by $^1H$ NMR.

The reaction conditions for screening of reductases by $^1H$ NMR were as follows: 1 mg.mL$^{-1}$ purified reductase, 0.5 mM $NADP^+$ (or $NAD^+$ for AniN6), 10 mM pyrroline substrate **7ai**, 0.25 mg.mL$^{-1}$ PtDH, in 100 mM NaPt buffer 100 mM pH 8.0. The total reaction volume was 500 µL and reactions were incubated for 24 h at 30 °C, 200 rpm. The reactions were subsequently centrifuged (13,000 rpm, 10 mins), freeze dried and resuspended in $D_2O$ (500 µL) for NMR analysis. Low conversion was seen in these reactions and so a replicate set of reactions was performed with

identical conditions except an additional portion of enzyme was added at 24 hours followed by a second incubation period (24 hours, 30 °C, 200 rpm). At the end of this second incubation, the reactions were centrifuged (13,000 rpm, 10 mins), freeze dried and resuspended in $D_2O$ for NMR analysis. A control reaction with omission of the reductase and otherwise identical conditions as above was also performed.

These biotransformations show that both *Nh*IRED and *Ad*RedAm have the capability to reduce pyrroline **7ai**, albeit in low to moderate yield. *Ad*RedAm was the more efficient of the two catalysts, forming 31% of pyrrolidine product **7aii** (relative to remaining substrate, 25% relative to all species) after 24 hours and 59% after 48 hours and two additions of 1 $mg.mL^{-1}$ enzyme (43% relative to all species). Conversely, no pyrrolidine product was seen in biotransformations with *Nh*IRED until 48 hours with two portions of enzyme (30% relative to remaining substrate, 19% relative to all species). Both *Ad*RedAm and *Nh*IRED appeared to form a major product stereoisomer which corresponds to the minor diastereomer formed in the chemically reduced product. No pyrrolidine was detected in biotransformations with AniN6. In all biotransformations (enzymatic and control) a non-enzymatic byproduct was also observed. The amount of byproduct is diminished in reactions where the pyrrolidine has formed, thus it is plausibly in equilibrium with the pyrroline substrate (e.g. the ring opened amino ketone).

Table S8**:** Integrals and relative ratios of substrate and product C**H**$_3$ signals in $^1$H NMR analysis of reductase biotransformations. Integrals are relative to the substrate C**H**$_3$ doublet which was set to 1. Relative distributions were calculated as proportion of all species present to nearest whole number (values in brackets represent amount relative to remaining substrate signal). 48h data points correspond to reactions with an additional portion of enzyme and extra 24 h incubation time as described above. The signal at 1.45 ppm corresponds to a non-enzymatic byproduct, 1.39 ppm one diastereomer of the pyrrolidine product, 1.37 ppm a transient unidentified byproduct and 1.32 ppm the pyrroline substrate **7ai**. The other diastereomer of the pyrrolidine product appears at 1.27 ppm and was formed as a near baseline minor product (c. 9:1 d.r. for *Ad*RedAm).

| | **Integral of $CH_3$ doublets** | | | | **Relative distribution / %** | | | |
|---|---|---|---|---|---|---|---|---|
| Reductase | 1.45 | 1.39 | 1.37 | 1.32 (**7ai**) | 1.45 | 1.39 | 1.37 | 1.32 (**7ai**) |
| AniN6 24h | 0.15 | - | 0.12 | 1 | 12 | - | 9 (11) | 79 |
| AniN6 48h | 0.62 | - | - | 1 | 38 | - | - | 62 |
| *Nh*IRED 24h | 0.25 | - | 0.22 | 1 | 17 | - | 15 (18) | 68 |
| *Nh*IRED 48h | 0.89 | 0.43 | - | 1 | 38 | 19 (30) | - | 43 |
| *Ad*RedAm 24h | 0.32 | 0.44 | - | 1 | 18 | 25 (31) | - | 57 |
| *Ad*RedAm 48h | 0.95 | 1.44 | - | 1 | 28 | 43 (59) | - | 29 |
| Control 24h | 0.32 | - | 0.20 | 1 | 21 | | 13 (17) | 66 |
| Control 48h | 0.84 | - | - | 1 | 46 | - | - | 54 |

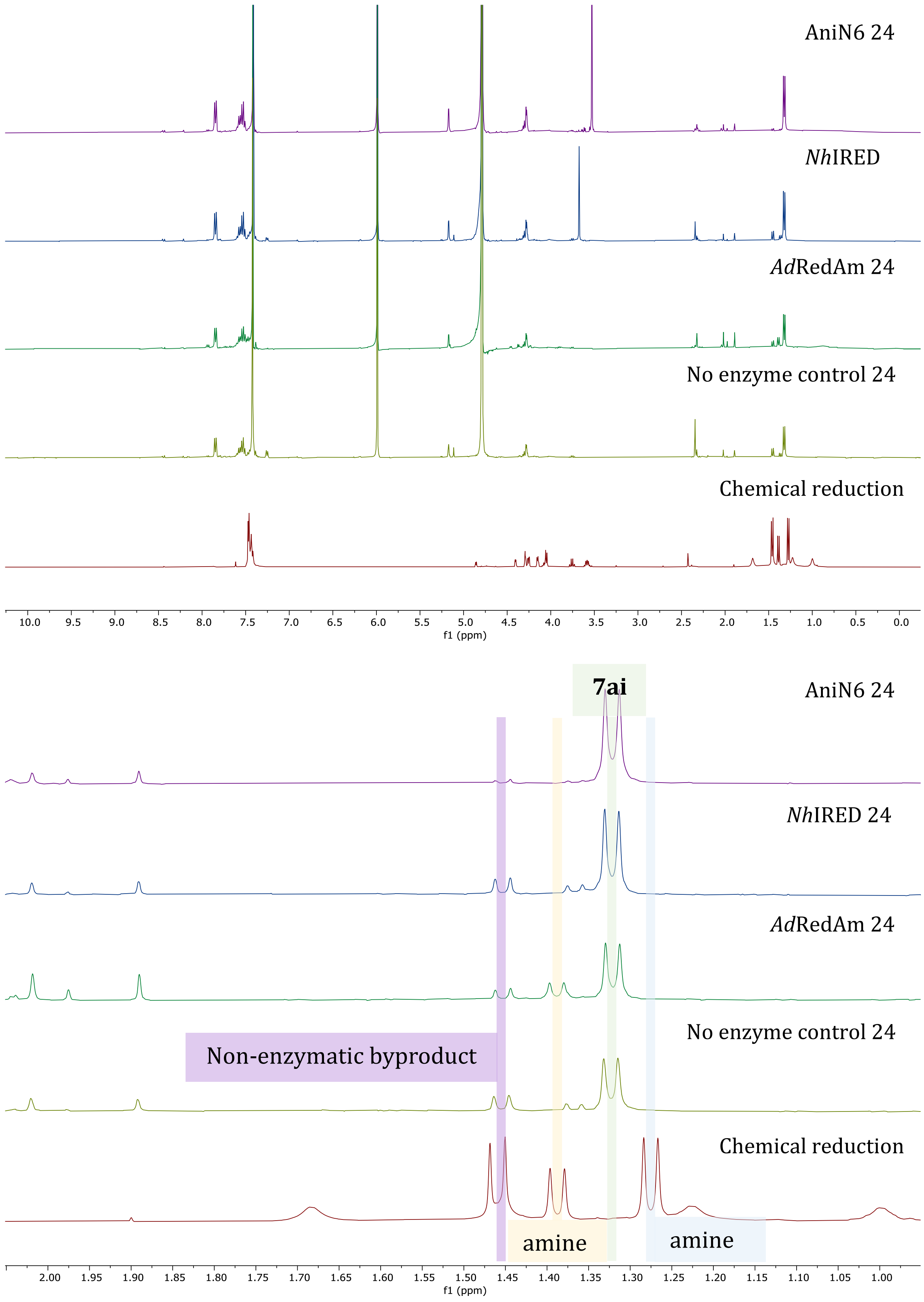


Figure S61**:** Stacked $^{1}H$ NMR spectra of reductase biotransformations with pyrroline substrate **7ai** at 24 hours. **Top:** spectral region 0–10 ppm, **bottom:** expansion pane of 1.0–2.0 ppm region showing key C**H**$_3$ doublets.

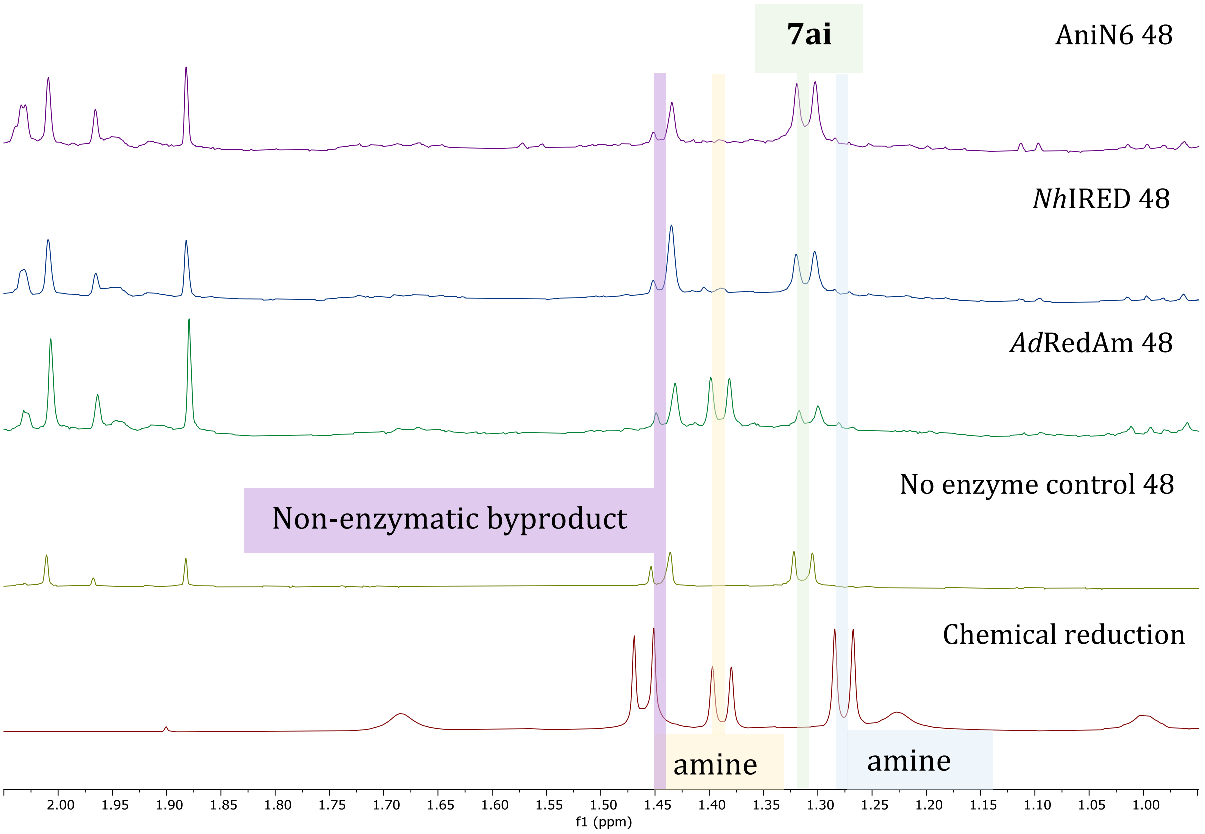


Figure S62**:** Stacked [1]H NMR spectra of reductase biotransformations with pyrroline substrate **7ai** at 48 hours after addition of additional enzyme and further incubation – expansion pane of 1.0–2.0 ppm region showing key C**H**$_3$ doublets.

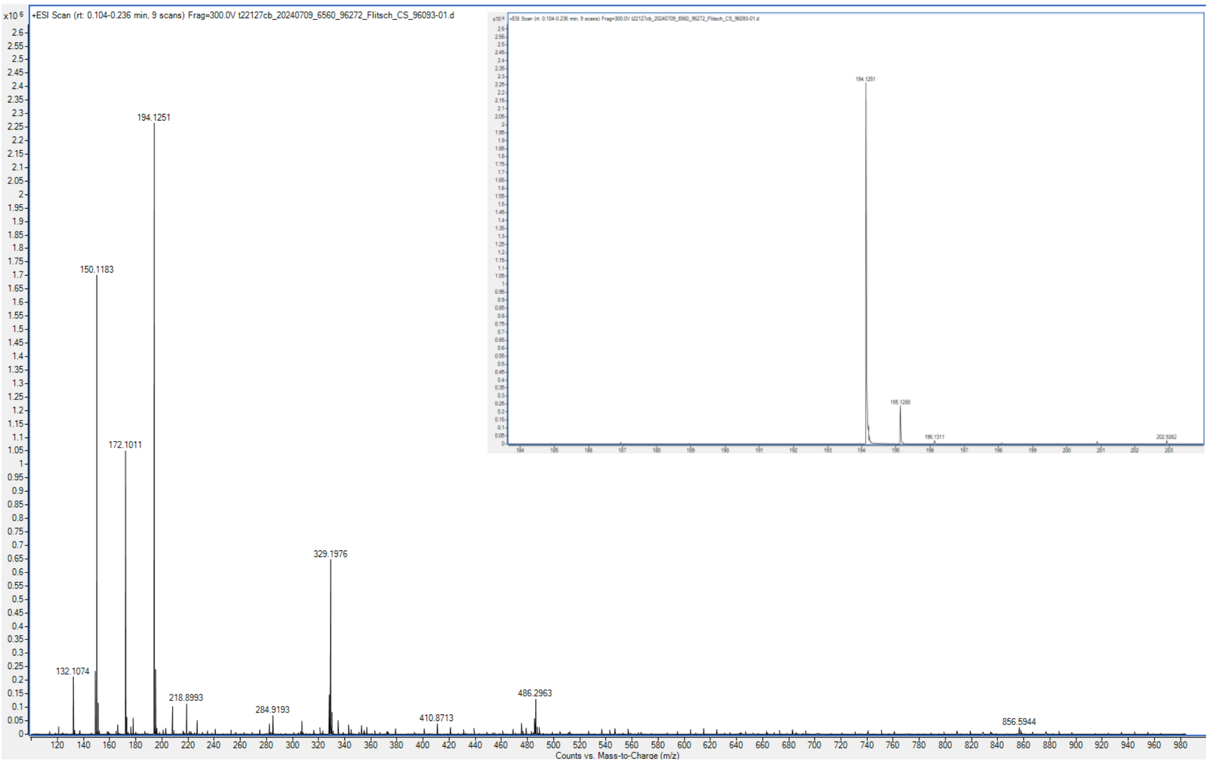


Figure S63: HRMS of the crude *Ad*RedAm catalyzed reaction showing *m/z* 194.1251, which corresponds to the pyrrolidine product **7aii**.

### *Comparison of selectivity in enzyme catalyzed and chemical imine reduction*

The reaction of the best performing enzyme, *Ad*RedAm was compared to the chemical reduction of the same pyrroline substrate **7ai**. Based on observations in the initial screening that an additional portion of enzyme gave improved conversions, the enzymatic biotransformation was performed with increased enzyme loading. In addition, the reactions were conducted in triethanolamine buffer to ensure comparability of results and compatibility to the conditions of the FSA-TA cascade. The conditions were as follows:

**Enzymatic biotransformation:** 2.5 mg/mL purified *Ad*RedAm, 0.5 mM $NADP^+$, 10 mM substrate, 0.25 mg/mL PtDH and 50 mM NaPt were dissolved in triethanolamine buffer (20 mM final concentration, pH 8.0). **Chemical reduction reaction:** 10 mM substrate and 20 mM $NH_3BH_3$ were dissolved in 50 mM NaPt and triethanolamine buffer (20 mM final concentration, pH 8.0). A **control reaction** with no reductant (chemical or enzymatic) was performed using 10 mM substrate dissolved in 50 mM NaPt and triethanolamine buffer (20 mM, pH 8.0). All reactions were performed with a total volume of 500 µL and incubated for 24 h at 30 °C, 200 rpm then centrifuged (13k rpm, 10 mins) and supplemented with 100 uL of $D_2O$ for analysis by $^1H$ water suppression NMR.

Analysis of the $^1H$ NMR after 24 hours shows only 26% conversion in the enzymatic biotransformation and a *d.r.* of 9:1 which corresponds to the value observed in the initial NMR

screening at 48h. Again, the major diastereomer of the enzymatic reaction corresponds to the minor diastereomer formed by chemical reduction, although absolute stereochemistry of these compounds has not been determined.

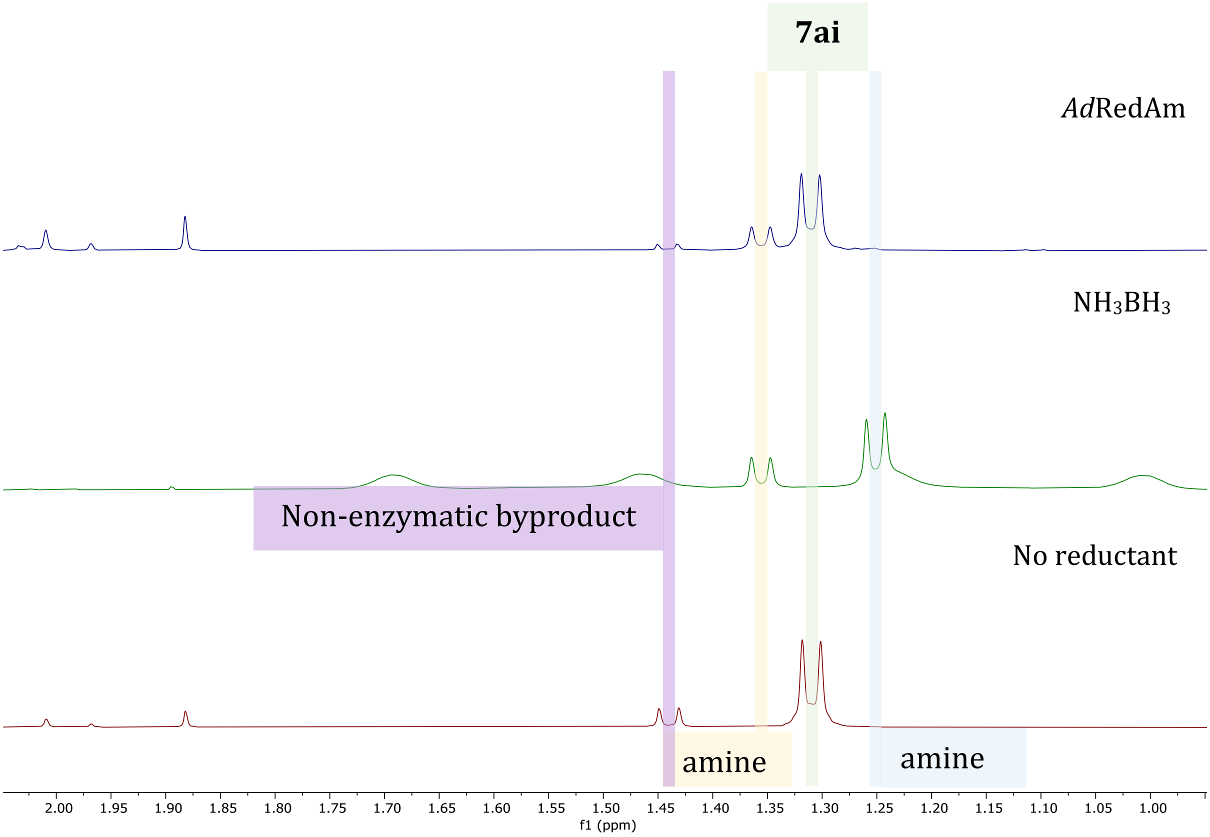


Figure S64: Stacked $^1H$ NMR spectra of the enzymatic, chemical and control reactions of pyrroline **7ai** – expansion pane of 1.0–2.0 ppm region showing key C**H**$_3$ doublets.

### *Attempts to scale-up the enzymatic reduction*

A 5 mL scale enzymatic reduction reaction with *Ad*RedAm was performed with the intention to isolate and characterize the pyrrolidine product; the conditions were as follows: 1 mg/mL purified *Ad*RedAm, 0.5 mM $NADP^+$, 10 mM substrate **7ai**, 0.25 mg/mL PtDH and 50 mM NaPt were dissolved in triethanolamine buffer (100 mM final concentration, pH 8.0). The total reaction volume was 500 μL and reactions were incubated at 30 °C, 200 rpm. At 24h, an aliquot was taken for $^1$H NMR analysis (500 μL), supplemented with $D_2O$ (50 μL) and centrifuged (13,000 rpm, 10 mins). An additional portion of 1 mg/mL enzyme was added to the reaction mixture and the reaction incubated for a further 24 hours before another sample was taken. This process was then repeated at 48 hours (total reaction time) until a third sample was taken at 72 hours. At this scale the enzymatic reduction proceeded much more slowly than at analytical scale and only reached 10% conversion by 48 hours after two additions of enzyme. By 72 hours of reaction time the substrate had degraded significantly.

### *Attempts to assemble the FSA-TA-IRED cascade*

Hoping to overcome the low activity of the reductase by forming the pyrroline **7ai** *in situ*, biotransformations comprising FSA, TA and reductase were performed. As with the FSA-TA cascade, biotransformations were performed in sequential and telescoped fashions.

#### *Telescoped FSA-TA-IRED cascade conditions*

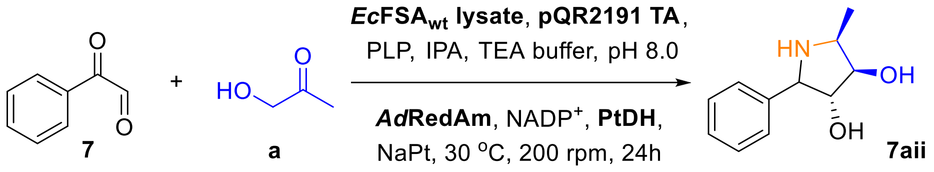


Reactions were carried out in 2 mL Eppendorf tubes with a total reaction volume of 600 µL. Each reaction contained components diluted from stock solutions, with final concentrations of 20 mM ketoaldehyde substrate, 20 mM donor substrate (hydroxyacetone, 1 equiv.), 200 mM IPA (10 equiv.), 1 mM PLP, 2 mg ml$^{-1}$ lyophilized FSA cell free extract, 1 mg ml$^{-1}$ pQR2191 TA, *Ad*RedAm (1 mg/mL), NADP+ (0.5 mM), PtDH (0.25 mg/ml) and sodium phosphite (100 mM) in 100 mM triethanolamine (TEA) buffer pH 8.0 containing 10 v% DMSO. Reactions were incubated at 30 °C, 200 rpm, 24 h and followed by $^1$H NMR analysis in $D_2O$ (250 uL biotransformation, 350 uL $D_2O$). A control reaction with $NH_3BH_3$ (100 mM) instead of the reductase components (*Ad*RedAm, NADP, PtDH and NaPt) was also performed. In this reaction the mixture quickly faded from a bright yellow to colourless, indicating reduction or reductive amination of the PLP required for transaminase reactivity.

#### *Sequential FSA-TA-IRED cascade conditions*

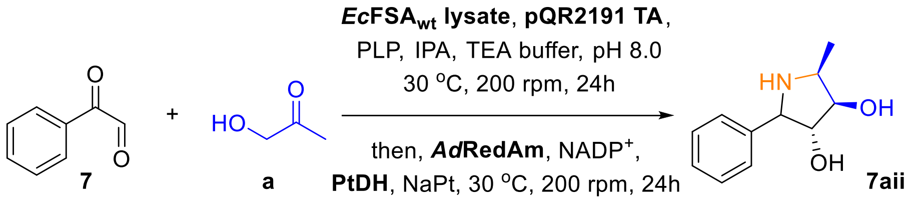


Reactions were carried out in 2 mL Eppendorf tubes with a total reaction volume of 600 µL. Each reaction contained components diluted from stock solutions, with final concentrations of 20 mM ketoaldehyde substrate, 20 mM donor substrate (hydroxyacetone, 1 equiv.), 200 mM IPA (10 equiv.), 1 mM PLP, 2 mg ml$^{-1}$ lyophilized FSA cell free extract, 1 mg ml$^{-1}$ pQR2191 TA in 100 mM triethanolamine (TEA) buffer pH 8.0 containing 10 v% DMSO. Reactions were incubated at 30 °C, 200 rpm for up to 24 h and followed by $^1$H NMR analysis in $D_2O$ (250 uL biotransformation, 350 uL $D_2O$). At 24h the reaction was supplemented with *Ad*RedAm (1 mg/mL), NADP+ (0.5 mM), PtDH (0.25 mg/ml) and sodium phosphite (100 mM) and incubated again at 30 °C, 200 rpm, 24 h with analysis by $^1$H NMR. A control reaction with $NH_3BH_3$ (100 mM) instead of the reductase components (*Ad*RedAm, NADP, PtDH and NaPt) was also performed.

Table S9: Relative distributions of products observed in FSA-TA-IRED cascade reactions. Data represent an average of two identical runs for each experiment.

| Reductant | Reaction mode | Total reaction time | Ratio of species present | | | |
|---|---|---|---|---|---|---|
| | | | HA (**a**) | **7a** | **7ai** | **7aii** |
| *Ad*RedAm | Telescoped | 24 | 3.3 | 16.6 | 70.3 | 9.9 |
| | | 48 | 5.9 | 17.1 | 67.8 | 9.2 |
| | Sequential | 48 | 5.3 | 19.9 | 63.1 | 11.7 |
| $NH_3BH_3$ | Telescoped | 24 | 0[a] | 0[a] | 0[a] | 0[a] |
| | Sequential | 48 | 0 | 0 | 26.4 | 73.6[b] |
| None | | 24 | 1.2 | 21.6 | 77.2 | 0 |

1H NMR signals used for determination of product ratios: HA **a** (C**H**$_3$, s, 2.12 ppm), **7a** (ArC**H**CC=O, 2H d, 8.00 ppm), **7ai** (ArC**H**CC=N, 2H d, 7.84 ppm), **7aii** (C**H**$_3$, 3H d, 1.35 ppm). [a] the telescoped reaction with $NH_3BH_3$ formed only reduction or reductive amination products of the substrates **7**, **a** and PLP with the isopropyl amine present in the reaction mixture. [b] calculated using only the minor diastereomer as the other diastereomer is partially obscured by the IPA signal.

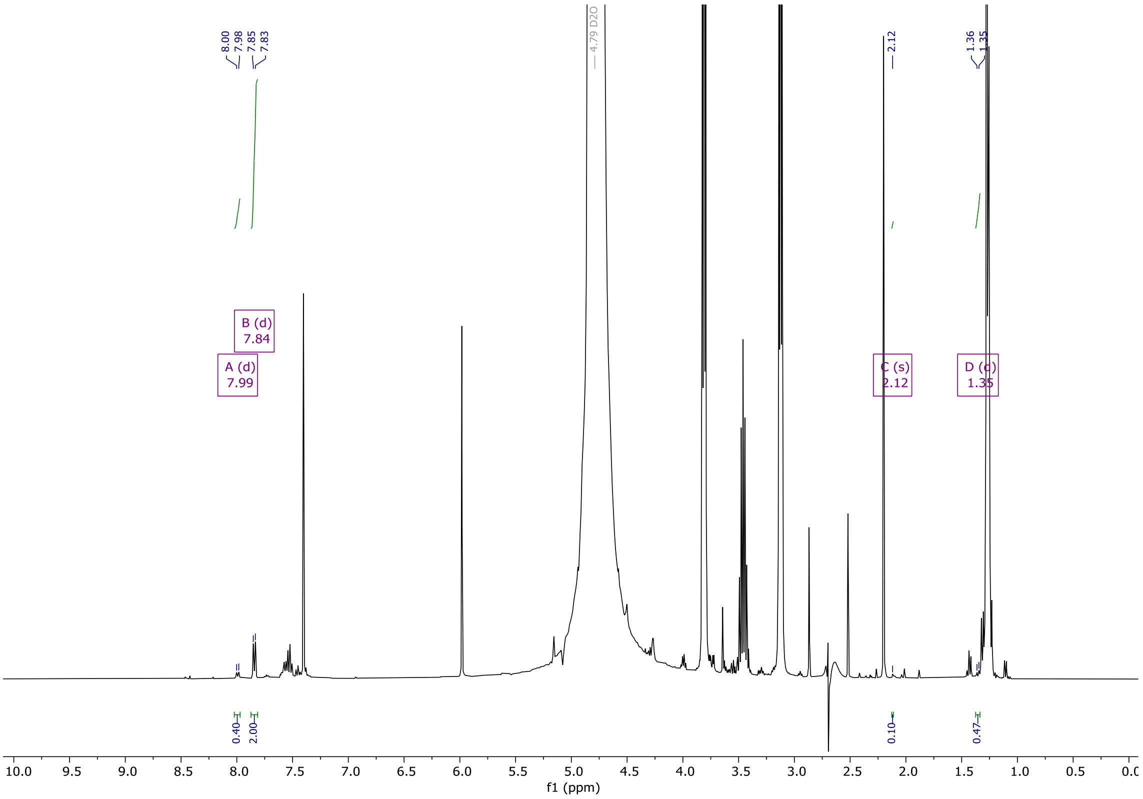


Figure S65: $^1$H NMR of the telescoped FSA-TA-*Ad*RedAm cascade at 24h. Integrated signals correspond to compounds **7a** (A), **7ai** (B), HA **a** (C) and **7aii** (D).

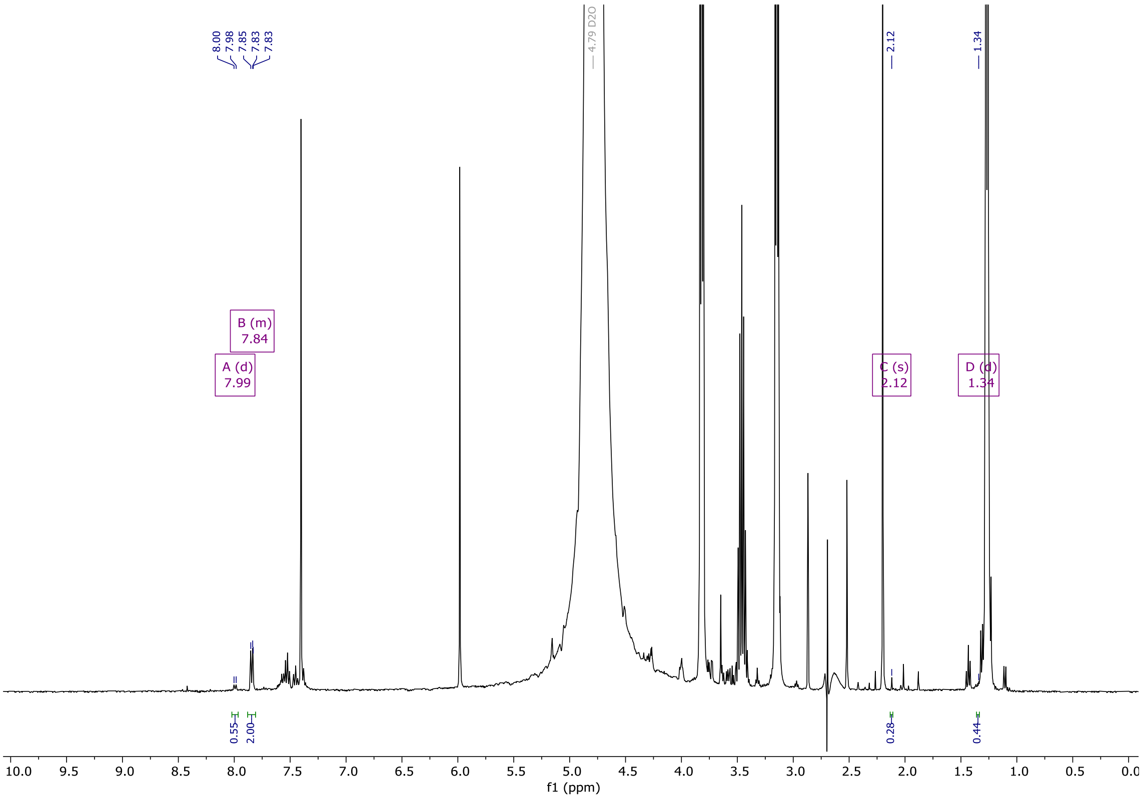


Figure S66: [1]H NMR of the telescoped FSA-TA-*Ad*RedAm cascade at 48h. Integrated signals correspond to compounds **7a** (A), **7ai** (B), HA **a** (C) and **7aii** (D).

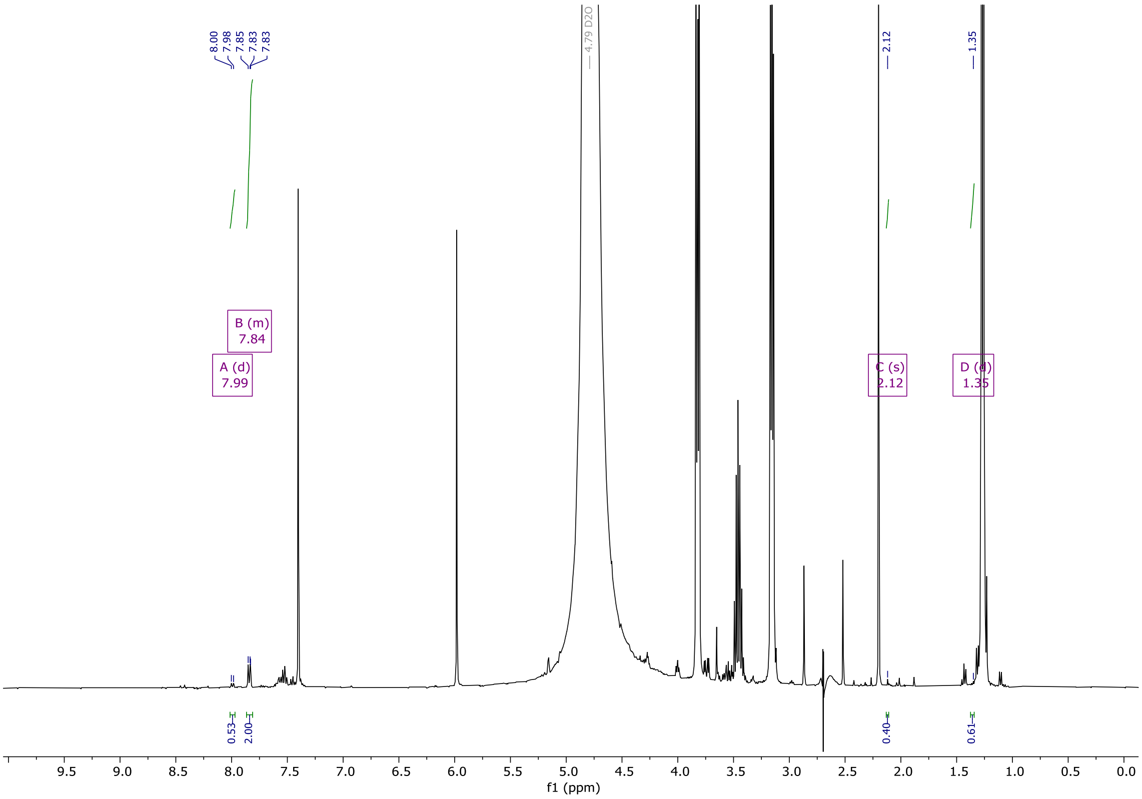


Figure S67: [1]H NMR of the sequential FSA-TA-*Ad*RedAm cascade at 48h. Integrated signals correspond to compounds **7a** (A), **7ai** (B), HA **a** (C) and **7aii** (D).

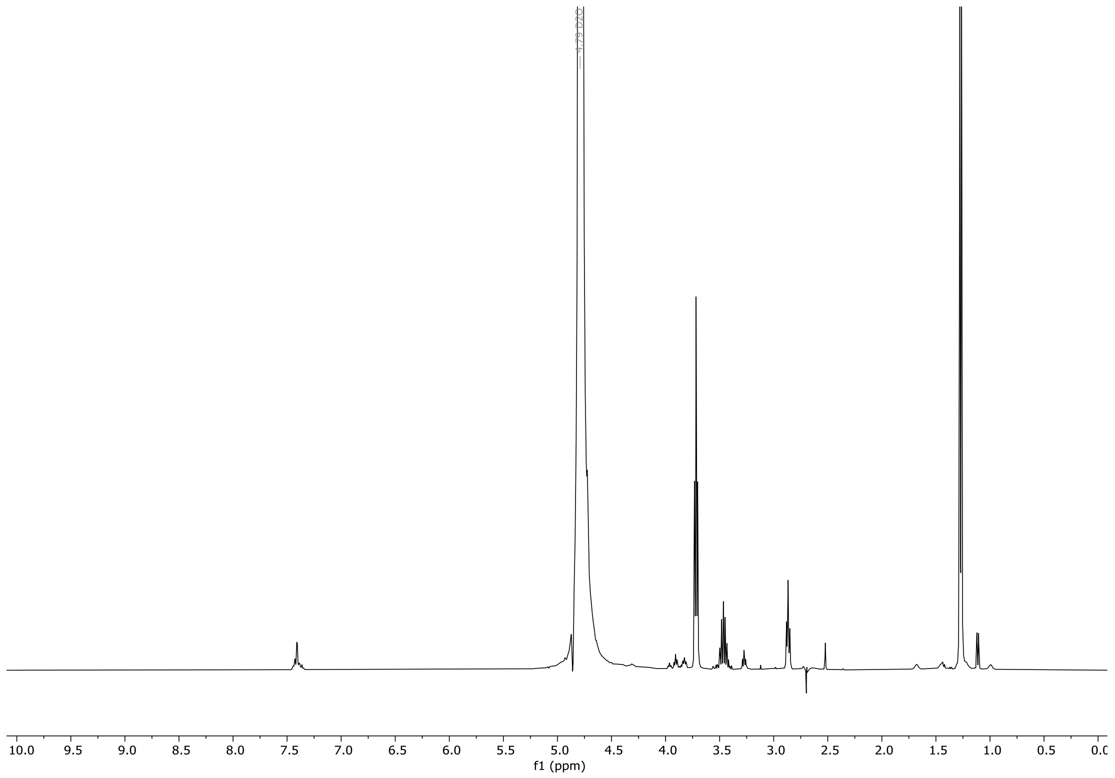


Figure S68: ¹H NMR of the telescoped FSA-TA-$NH_3BH_3$ cascade reaction at 24h. Full depletion of the HA **a** signal at 2.12 is observed but no peaks from the desired products **7a**, **7ai** or **7aii** are observed. Undesired reductions or reductive aminations of the starting materials **7**, **a** and PLP with the IPA present in the reaction are likely to have occurred and this is supported by the newly formed doublet at 1.11 ppm which could be the methyl group of a reduced HA species.

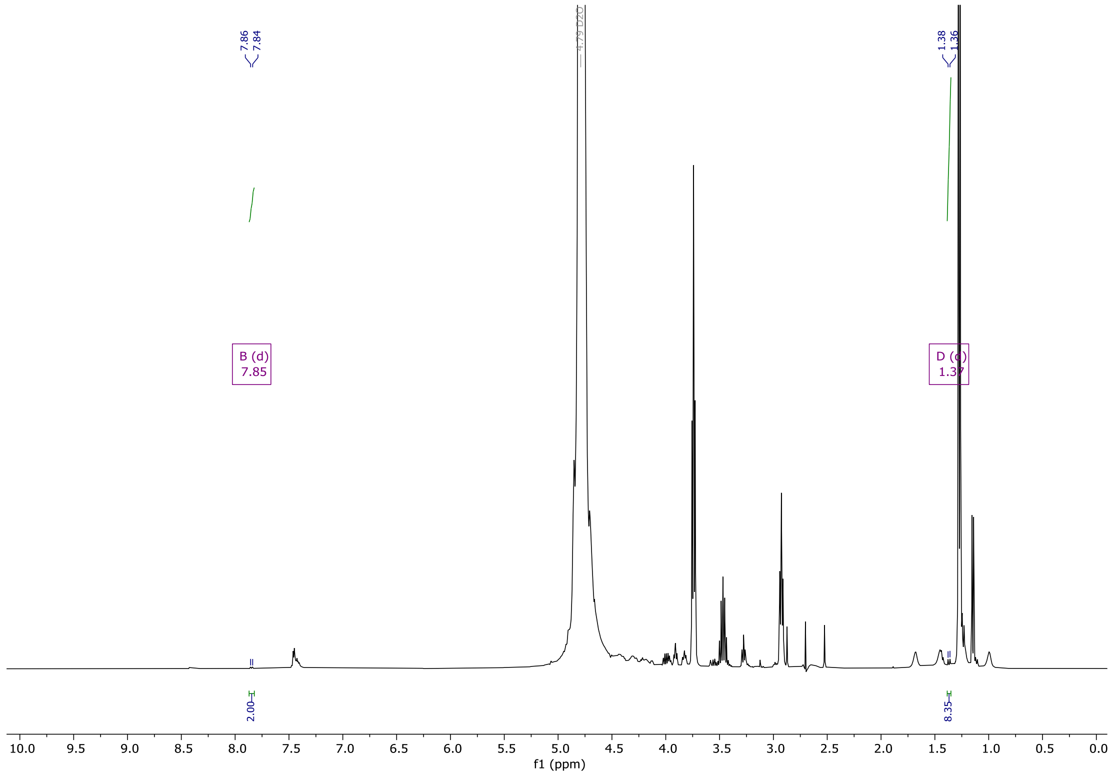


Figure S69: ¹H NMR of the sequential FSA-TA-$NH_3BH_3$ cascade at 48h. Integrated signals correspond to compounds **7ai** (B), and **7aii** (D). No remaining **7a** or HA **a** were observed.

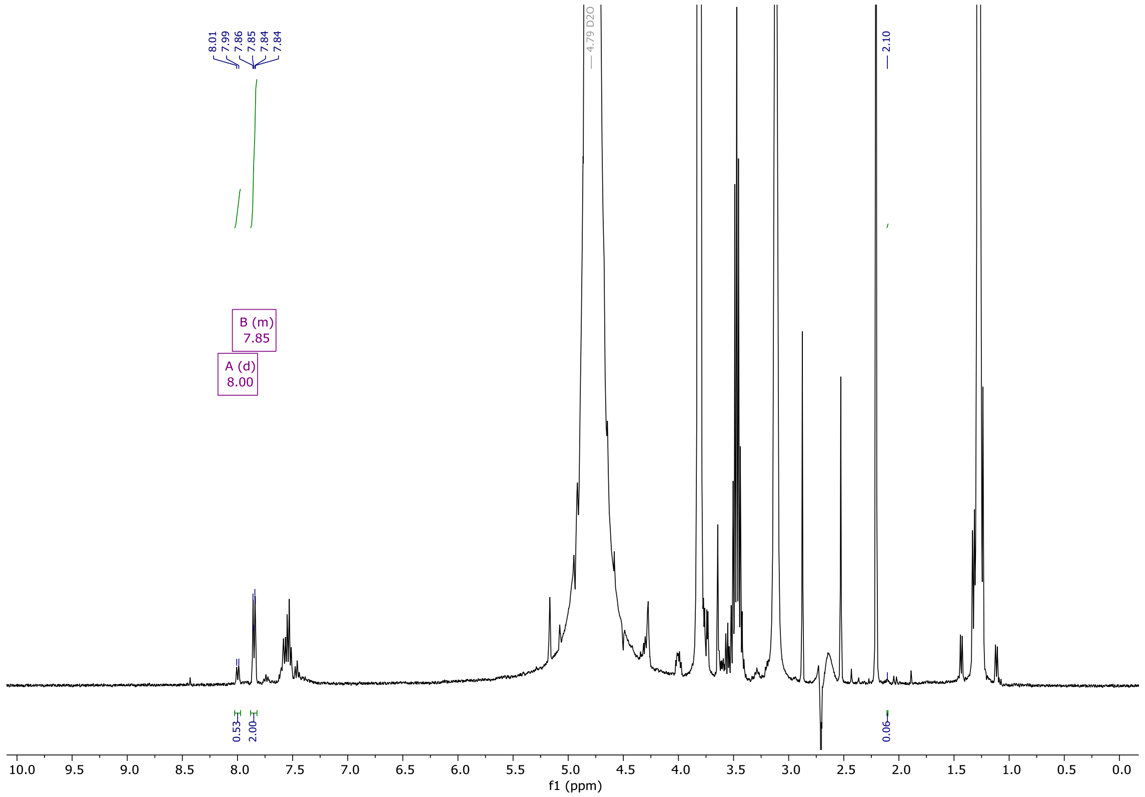


Figure S70: $^{1}H$ NMR of an FSA-TA-IRED cascade biotransformation with no reductant at 24h. Integrated signals correspond to compounds **7a** (A), **7ai** (B) and HA **a** (2.10 ppm). No **7aii** is observed.

## *Glycosidase activity assays*

Glycosidase activity assays were performed to determine if any of the produced compounds exhibit inhibition against commonly tested glycosidases. The enzymes α-glucosidase from rice, β-glucosidase from almond, α-galactosidase from green coffee bean, β-galactosidase from *Aspergillus oryzae* and α-mannosidase from jack bean were purchased from Sigma Aldrich along with their respective *para*-nitrophenyl glycoside substrates.

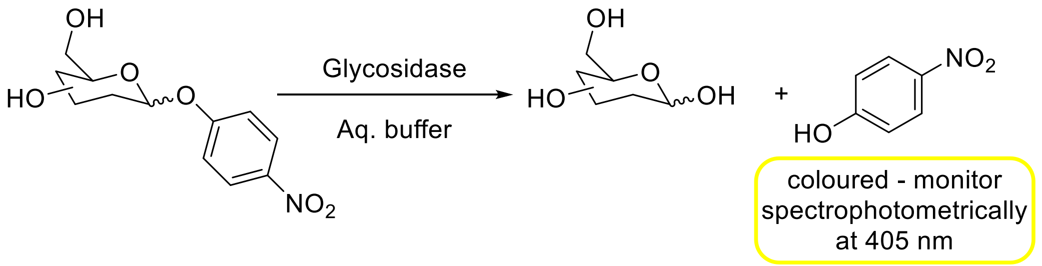


Figure S71: Reaction scheme of the colourimetric assay used to monitor glycosidase hydrolysis activity and inhibition

Assays were performed in a flat bottomed 96-well plate by premixing glycosidase enzyme (0.0015–0.015 mg.mL$^{-1}$) with potential inhibitors (1 mM) in NaPi buffer (50 mM, pH 6.8) for one hour at 30 °C before addition of the *p*-nitrophenyl glycoside substrate where by the reaction was maintained at 30 °C and the UV absorbance at 405 nm was measured using a Tecan Infinite Pro plate reader. Mean slopes of kinetic data reduction across the first 5–15 minutes of reaction time were calculated in Magellan to determine the effectiveness of each compound as a potential inhibitor. A further control reaction with 1-DNJ as a known inhibitor was performed. Reactions were performed in triplicate and the calculated rates and errors are shown in the table below.

Table S10: Rates of hydrolysis measured for various glycosidases in the presence or absence of compounds **7a**, **7ai**, **7aii**, **9ai**, 1-DNJ and DAB-1.

| Glycosidase | Rate of hydrolysis of *p*-nitrophenyl glycoside (min$^{-1}$) | | | | | | |
|---|---|---|---|---|---|---|---|
| | **7a** | **7ai** | **7aii** | **9ai** | **None** | **1-DNJ** | **DAB-1** |
| α-glucosidase (rice) | 0.0125 (±0.00054) | 0.0134 (±0.00012) | 0.0115 (±0.000052) | 0.0129 (±0.000066) | 0.0131 (±0.000125) | 0.000308 (±0.00030) | 0.000428 (±0.00013) |
| β-glucosidase (almond) | 0.577 (±0.0025) | 0.491 (±0.0056) | 0.416 (±0.0088) | *n.d.* | 0.601 (±0.0088) | 0.0958 (±0.0075) | 0.0778 (±0.0023) |
| α-galactosidase (green coffee bean) | 0.188 (±0.059) | 0.166 (±0.015) | 0.232 (±0.0067) | 0.200 (±0.0037) | 0.192 (±0.0074) | 0.114 (±0.0041) | 0.214 (±0.0050) |
| β-galactosidase (*Aspergillus oryzae*) | 0.0192 [a] (±0.00048) | 0.0193 [a] (±0.00010) | 0.0173 [a] (±0.0023) | 0.0191 [a] (±0.00015) | 0.0187 [a] (±0.00016) | 0.0183 [a] (±0.000065) | *n.d.* |
| α-mannosidase (jack bean) | 0.484 (±0.017) | 0.471 (±0.0039) | 0.345 (±0.013) | 0.518 (±0.010) | 0.485 (±0.013) | 0.0721 (±0.0018) | 0.0120 (±0.0011) |

[a] Data for β-galactosidase (*Aspergillus oryzae*) given in units of s-1.

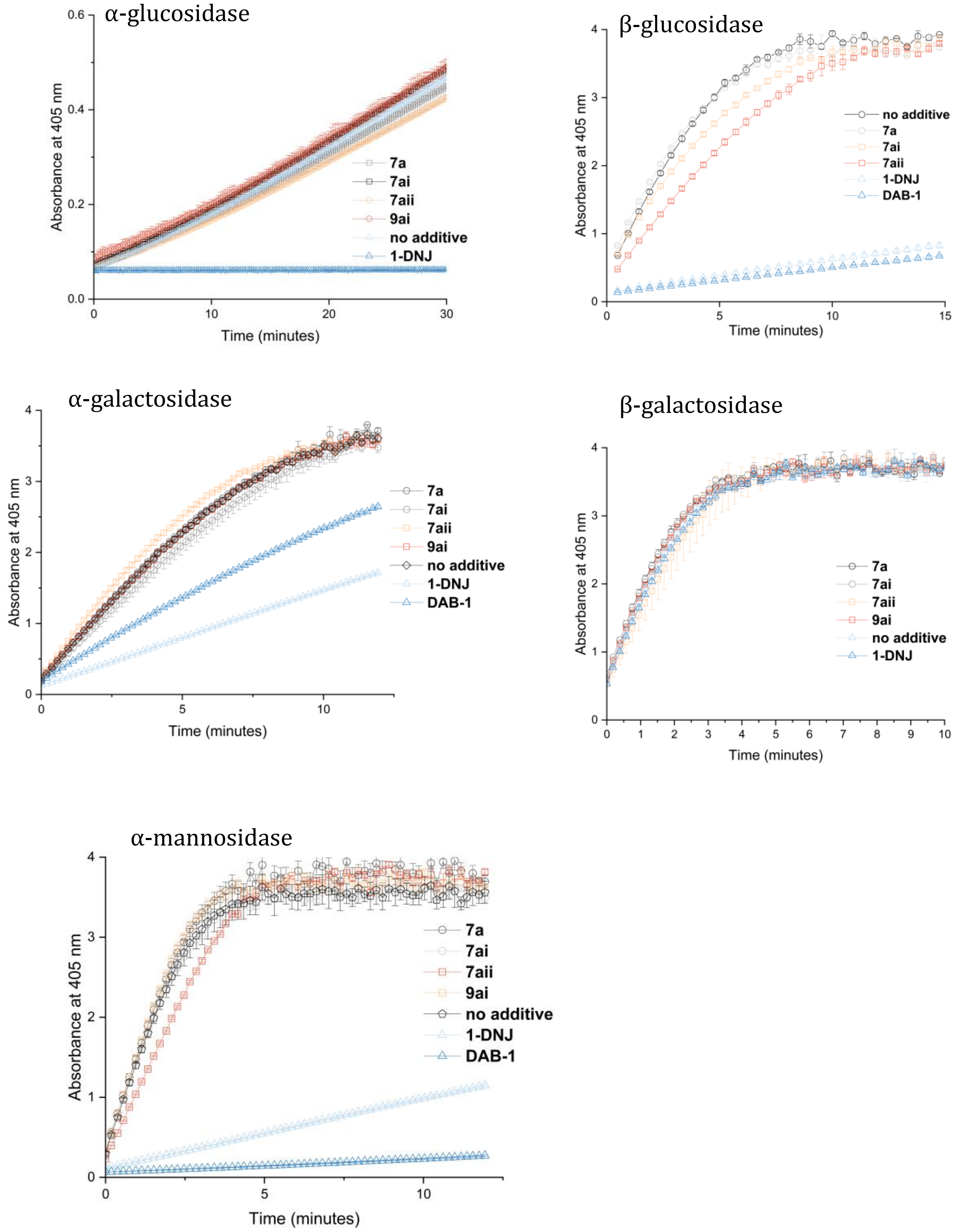


Figure S72: Progress curves for glycosidase inhibition reactions

## *UPLC-QDa chromatograms of FSA biotransformations*

### *Calibration curves*

| Concentration / mM | Peak Area |
|---|---|
| 2 | 19211714 |
| 1 | 9402134 |
| 0.5 | 4731915 |
| 0.25 | 2334962 |
| 0.125 | 1193119 |
| 0.0675 | 588155 |

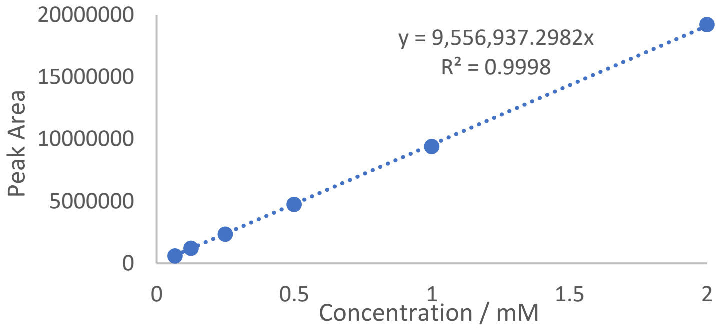


Figure S73: Calibration curve of phenylglyoxal **7**. Data collected using UPLC method 2 at 254 nm.

| Concentration / mM | Peak Area |
|---|---|
| 2 | 30016523 |
| 1 | 16579358 |
| 0.5 | 8638948 |
| 0.25 | 5464264 |
| 0.125 | 2678941 |

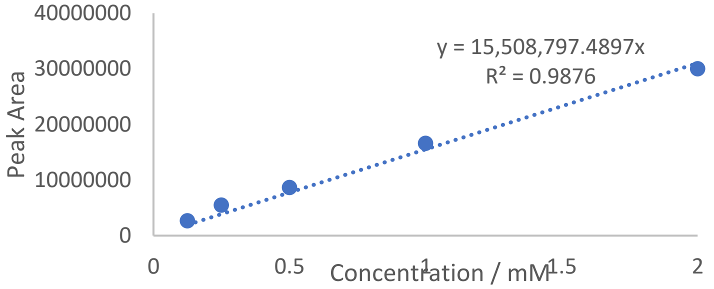


Figure S74: Calibration curve of 4-bromophenylglyoxal **8**. Data collected using UPLC method 2 at 254 nm.

| Concentration / mM | Peak Area |
|---|---|
| 2 | 26112206 |
| 1 | 13608198 |
| 0.5 | 7624035 |
| 0.25 | 4245196 |
| 0.125 | 2233820 |

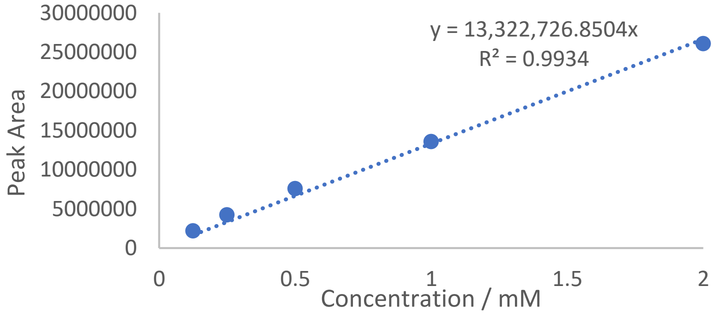


Figure S75: Calibration curve of 4-fluorophenylglyoxal **9**. Data collected using UPLC method 2 at 254 nm.

| Concentration / mM | Peak Area |
|---|---|
| 2 | 8434695 |
| 1 | 4619112 |
| 0.5 | 2554181 |
| 0.25 | 1331986 |
| 0.125 | 648082 |

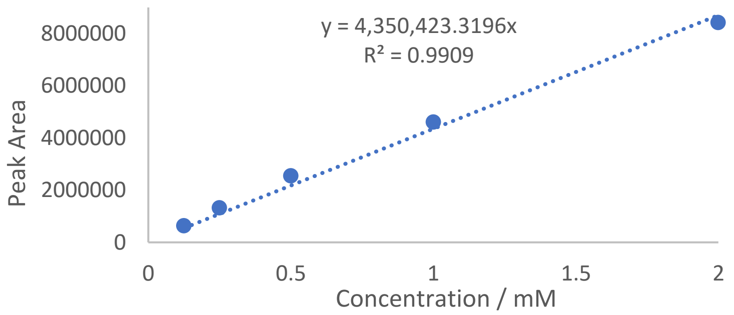


Figure S76: Calibration curve of 4-trifluoromethylphenylglyoxal **10**. Data collected using UPLC method 2 at 254 nm.

| Concentration / mM | Peak Area |
|---|---|
| 2 | 13275133 |
| 1 | 6993741 |
| 0.5 | 3706772 |
| 0.25 | 2014760 |
| 0.125 | 1111283 |

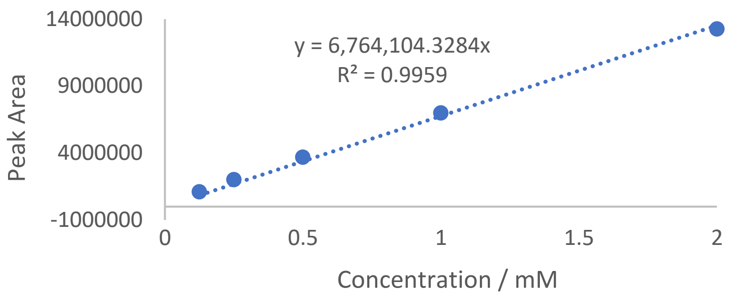


Figure S77: Calibration curve of 3-methoxyphenylglyoxal **11**. Data collected using UPLC method 2 at 254 nm.

| Concentration / mM | Peak Area |
|---|---|
| 2 | 5313409 |
| 1 | 2644440 |
| 0.5 | 1479368 |
| 0.25 | 802548 |
| 0.125 | 421584 |

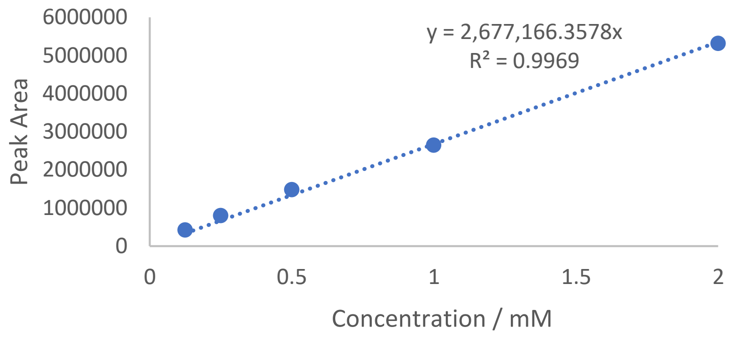


Figure S78: Calibration curve of 3-bromophenylglyoxal **12**. Data collected using UPLC method 2 at 254 nm.

| Concentration / mM | Peak Area |
|---|---|
| 2 | 9210038 |
| 1 | 4810557 |
| 0.5 | 2280240 |
| 0.25 | 1433655 |
| 0.125 | 688467 |

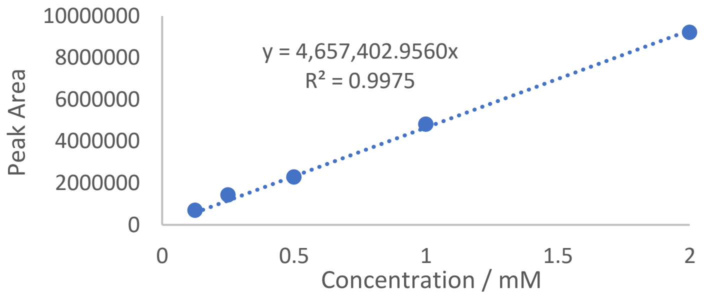


Figure S79: Calibration curve of 3-chlorophenylglyoxal **13**. Data collected using UPLC method 2 at 254 nm.

| Concentration / mM | Peak Area |
|---|---|
| 2 | 6831425 |
| 1 | 3283898 |
| 0.5 | 1749083 |
| 0.25 | 940793 |
| 0.125 | 536961 |

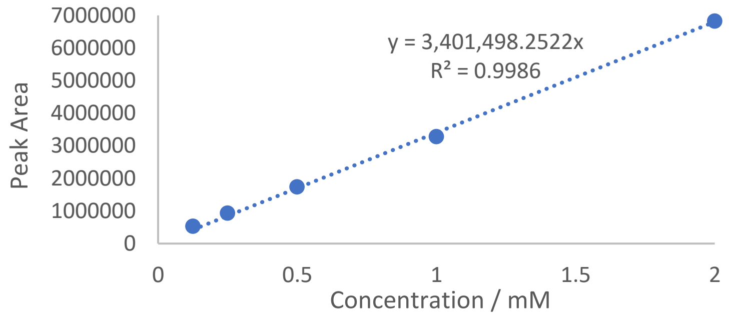


Figure S80: Calibration curve of 5-bromothienylglyoxal **14**. Data collected using UPLC method 2 at 254 nm.

| Concentration / mM | Peak Area |
|---|---|
| 2 | 2105741 |
| 1 | 1060735 |
| 0.5 | 588070 |
| 0.25 | 308195 |
| 0.125 | 203772 |

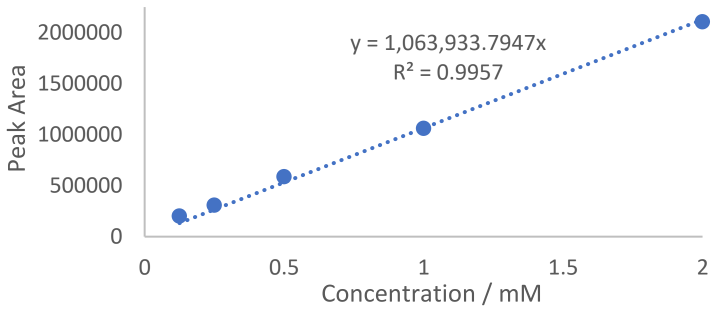


Figure S81: Calibration curve of 3,4-methylenedioxyphenylglyoxal **15**. Data collected using UPLC method 2 at 254 nm.

| Concentration / mM | Peak Area |
|---|---|
| 2 | 9284841 |
| 1 | 5158245 |
| 0.5 | 2928103 |
| 0.25 | 1619946 |
| 0.125 | 870927 |

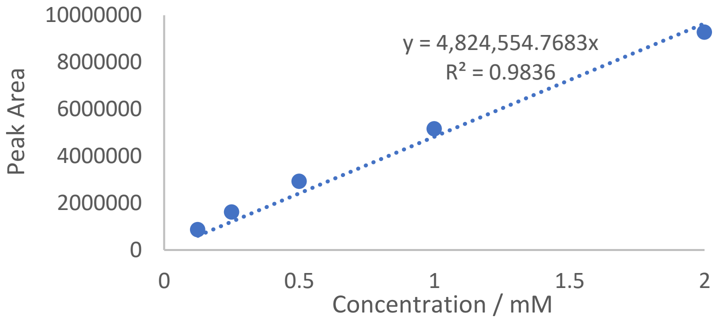


Figure S82: Calibration curve of 4-hydroxyphenylglyoxal **16**. Data collected using UPLC method 2 at 254 nm.

| Concentration / mM | Peak Area |
|---|---|
| 2 | 4151543 |
| 1 | 2195870 |
| 0.5 | 1256780 |
| 0.25 | 694067 |
| 0.125 | 378797 |

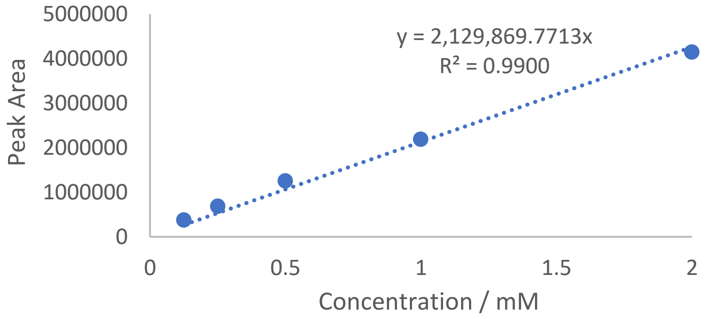


Figure S83: Calibration curve of 4-morpholinophenylglyoxal **17**. Data collected using UPLC method 2 at 254 nm.

| Concentration / mM | Peak Area |
|---|---|
| 2 | 4566383 |
| 1 | 2293920 |
| 0.5 | 1207869 |
| 0.25 | 571989 |
| 0.125 | 361282 |

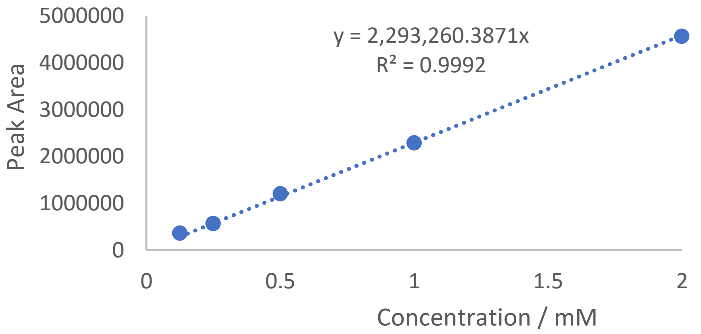


Figure S84: Calibration curve of 2-trifluoromethylphenylglyoxal **18**. Data collected using UPLC method 2 at 254 nm.

| Concentration / mM | Peak Area |
|---|---|
| 2 | 3300998 |
| 1 | 1774084 |
| 0.5 | 989492 |
| 0.25 | 577204 |
| 0.125 | 283154 |

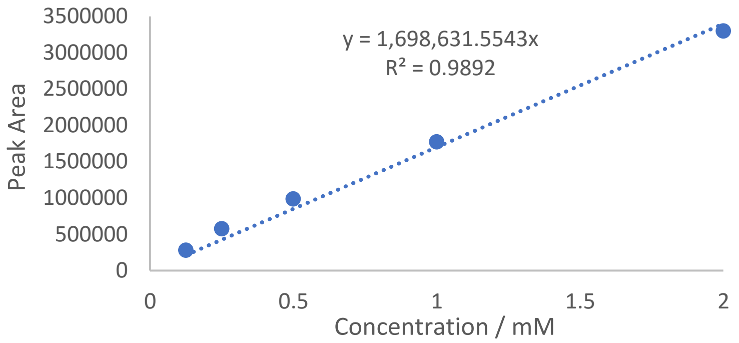


Figure S85: Calibration curve of 4-azidophenylglyoxal **19**. Data collected using UPLC method 2 at 254 nm.

| Concentration / mM | Peak Area |
|---|---|
| 2 | 18252493 |
| 1 | 10239801 |
| 0.5 | 5878215 |
| 0.25 | 3276737 |
| 0.125 | 1818240 |

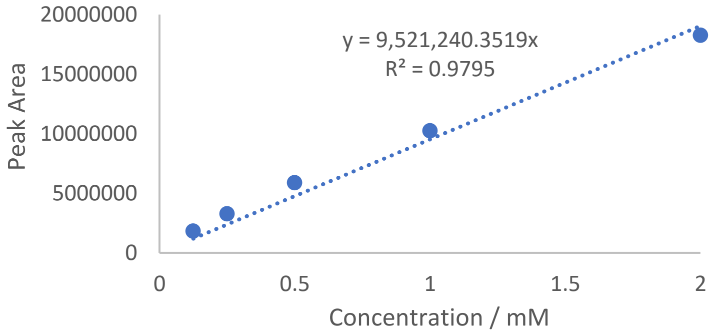


Figure S86: Calibration curve of 2,4-difluorophenylglyoxal **20**. Data collected using UPLC method 2 at 254 nm.

| Concentration / mM | Peak Area |
|---|---|
| 2 | 27918906 |
| 1 | 15749126 |
| 0.5 | 7158694 |
| 0.25 | 3644140 |
| 0.125 | 1989685 |

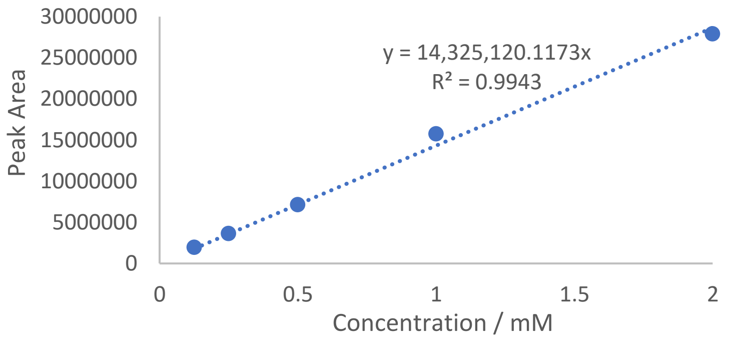


Figure S87: Calibration curve of 6-methoxynapthylglyoxal **21**. Data collected using UPLC method 2 at 254 nm.

*Zero-hour biotransformations*

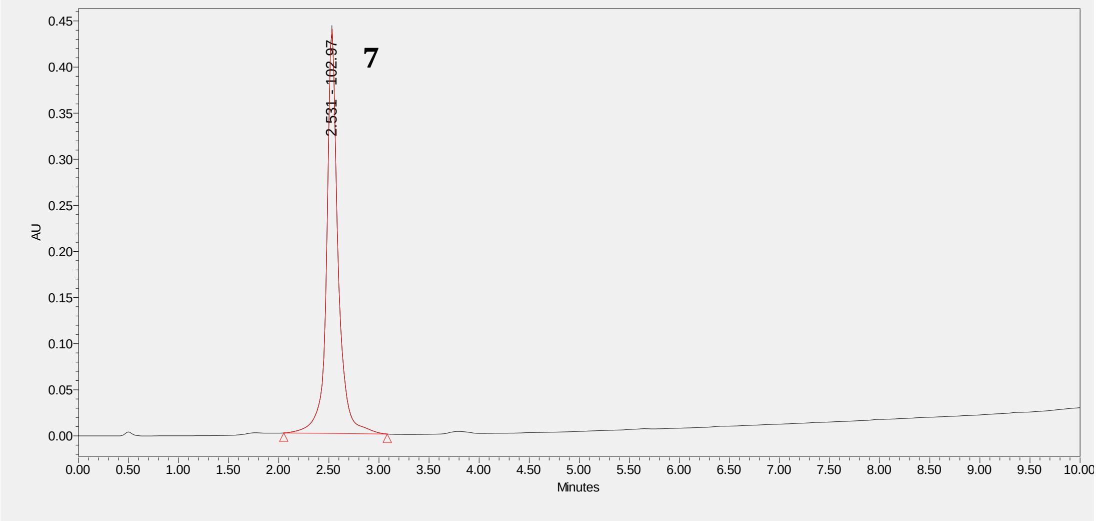


Figure S88**:** Reverse phase (C18) UPLC-QDa trace (method 1) of phenylglyoxal **7** hydrate substrate standard.

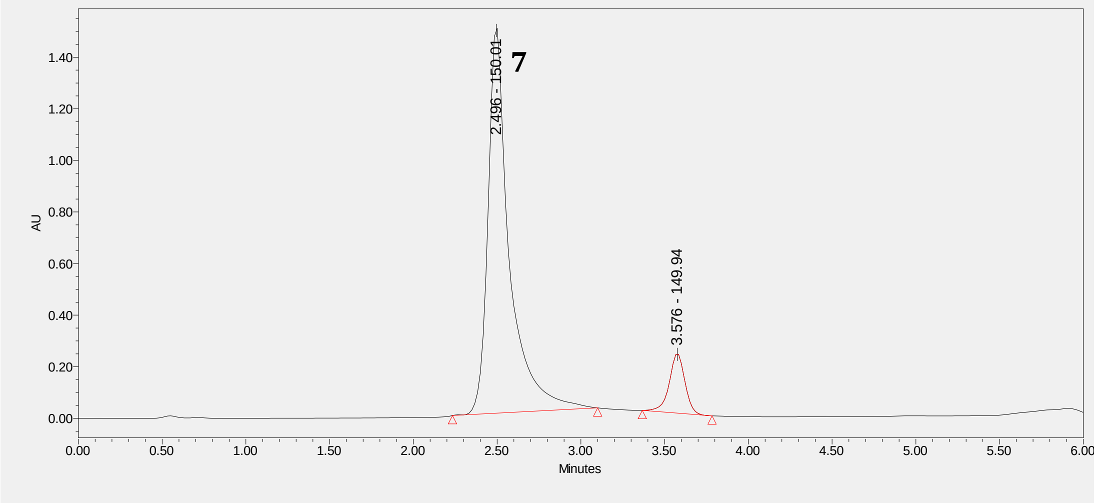


Figure S89**:** UPLC-QDa trace (method 2) of phenylglyoxal **7** biotransformation at 0h.

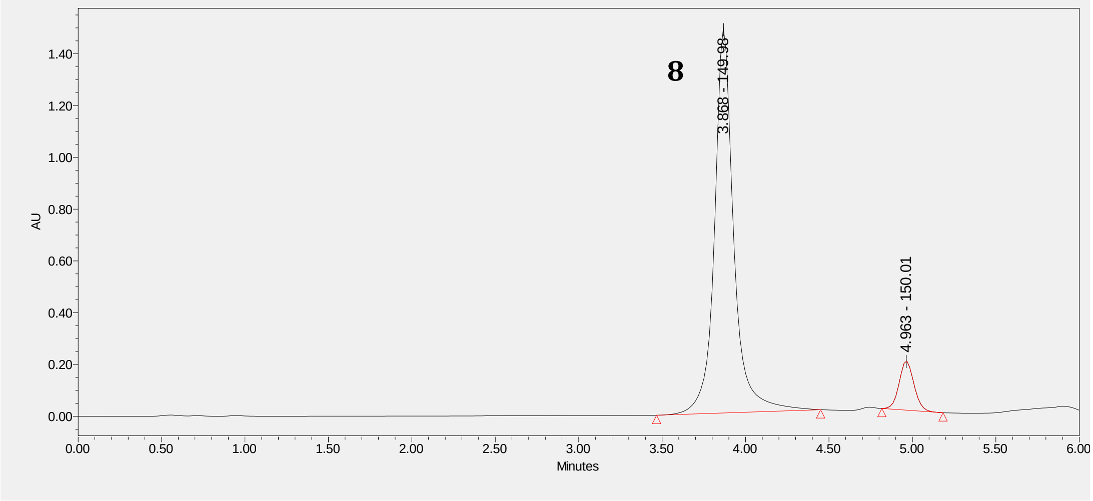


Figure S90**:** UPLC-QDa trace (method 2) of 4-BrPhglyoxal **8** biotransformation at 0h.

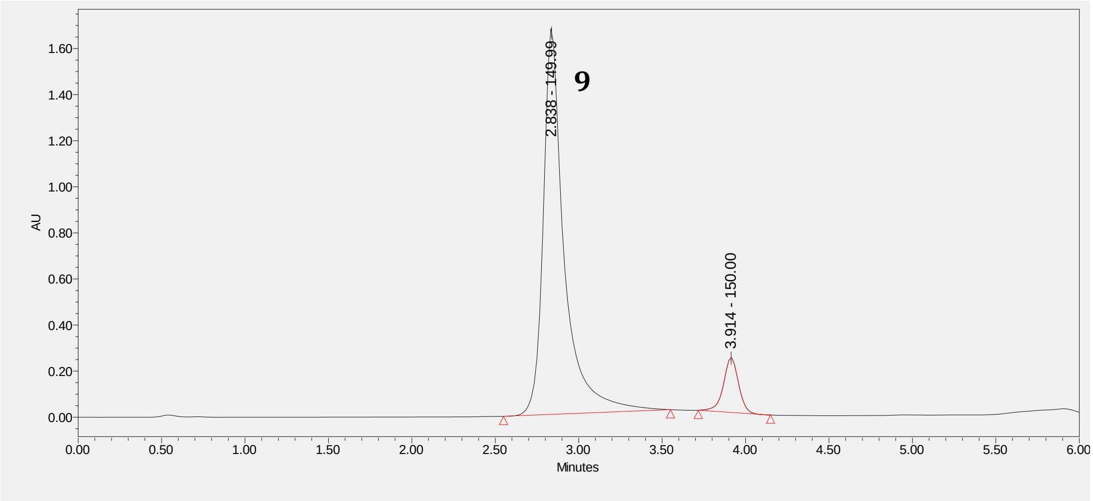


Figure S91: UPLC-QDa trace (method 2) of 4-FPhglyoxal **9** biotransformation at 0h.

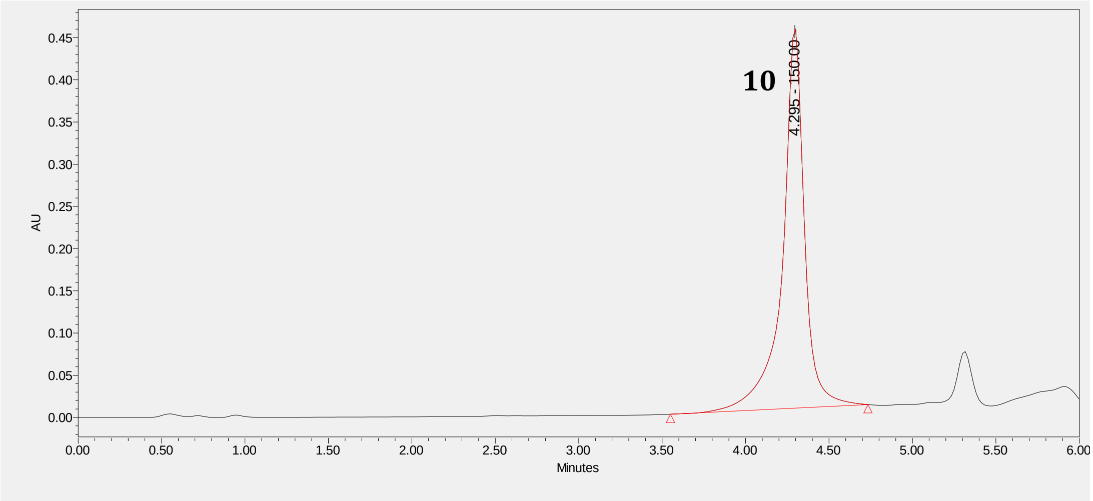


Figure S92: UPLC-QDa trace (method 2) of 4-$CF_3$Phglyoxal **10** biotransformation at 0h.

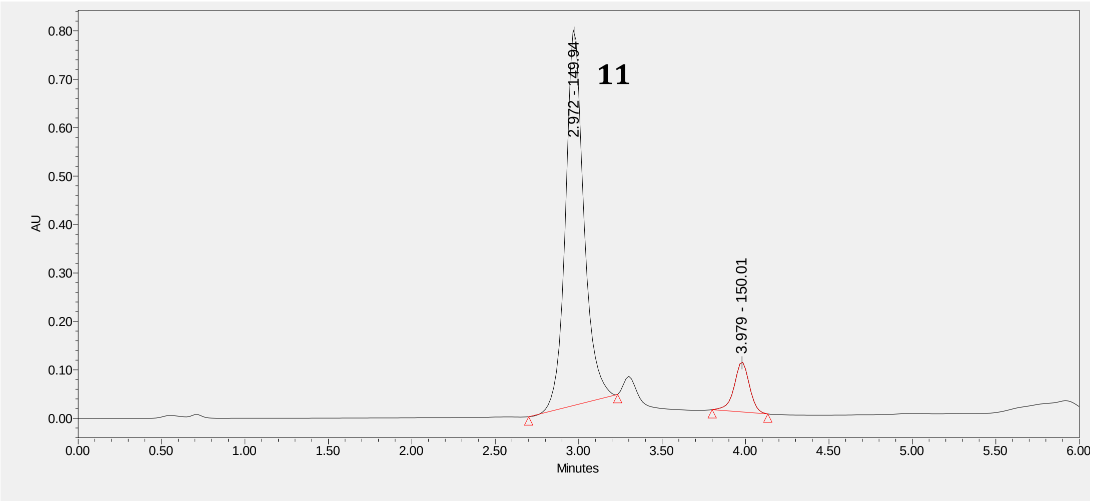


Figure S93: UPLC-QDa trace (method 2) of 3-OMePhglyoxal **11** biotransformation at 0h.

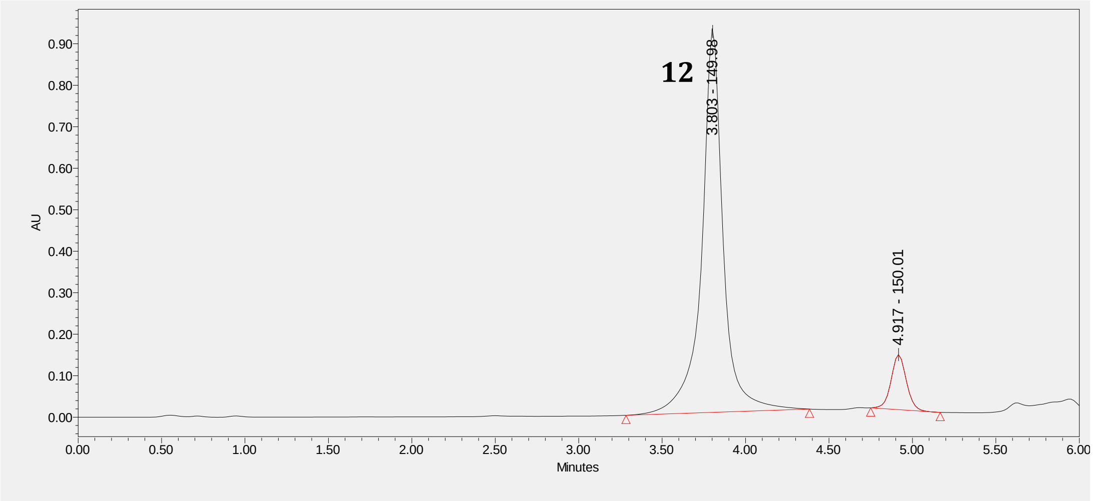


Figure S94: UPLC-QDa trace (method 2) of 3-BrPhglyoxal **12** biotransformation at 0h.

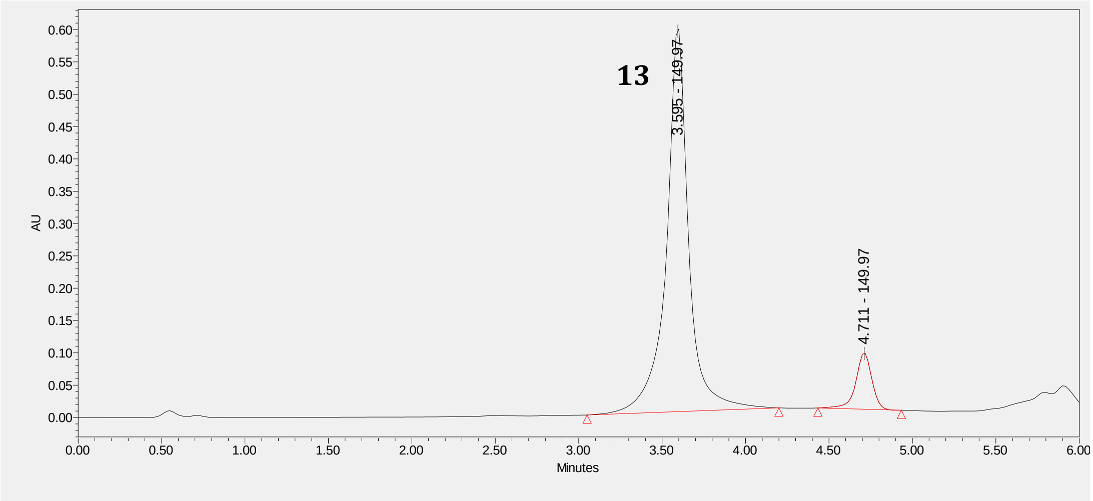


Figure S95: UPLC-QDa trace (method 2) of 3-ClPhglyoxal **13** biotransformation at 0h.

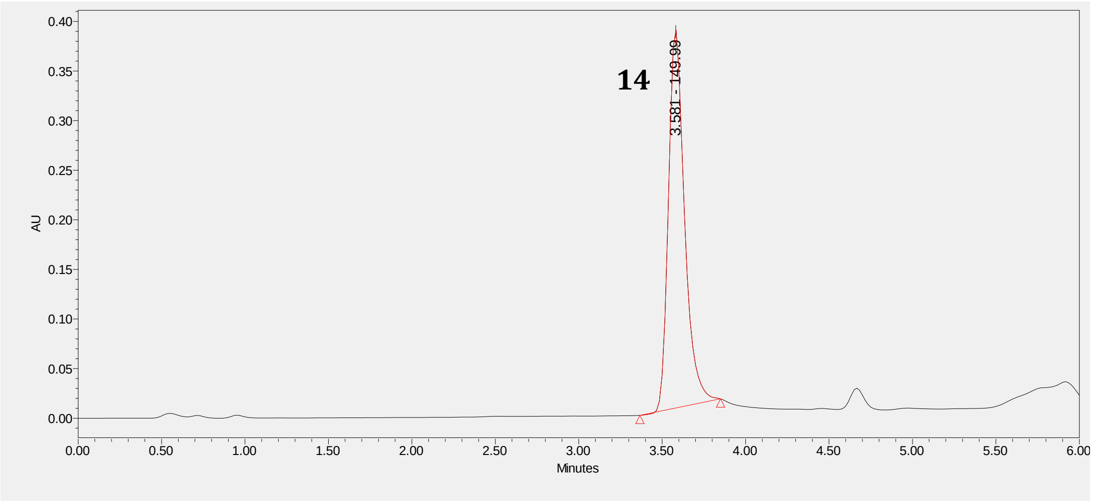


Figure S96: UPLC-QDa trace (method 2) of 5-BrThienylglyoxal **14** biotransformation at 0h.

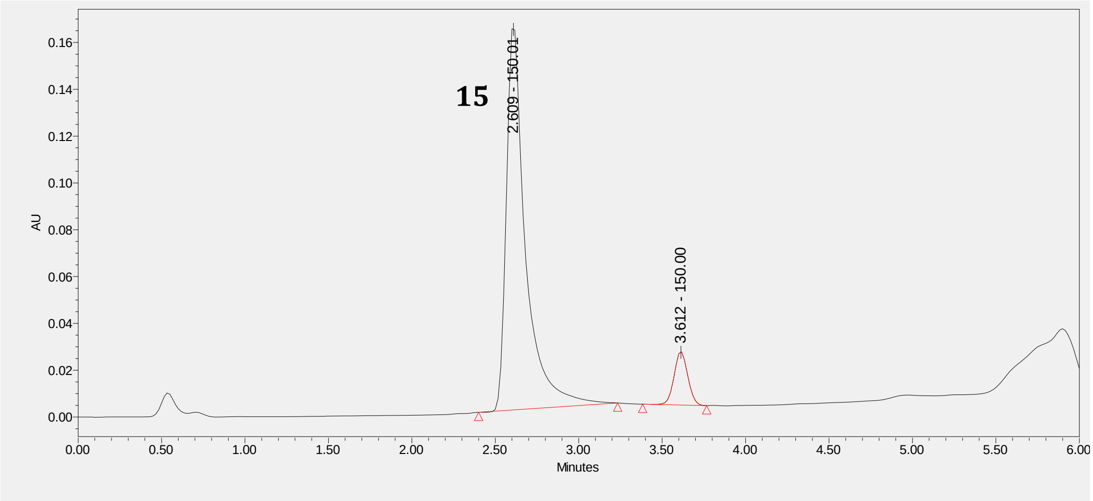


Figure S97**:** UPLC-QDa trace (method 2) of 3,4-methylenedioxyPhglyoxal **15** biotransformation at 0h.

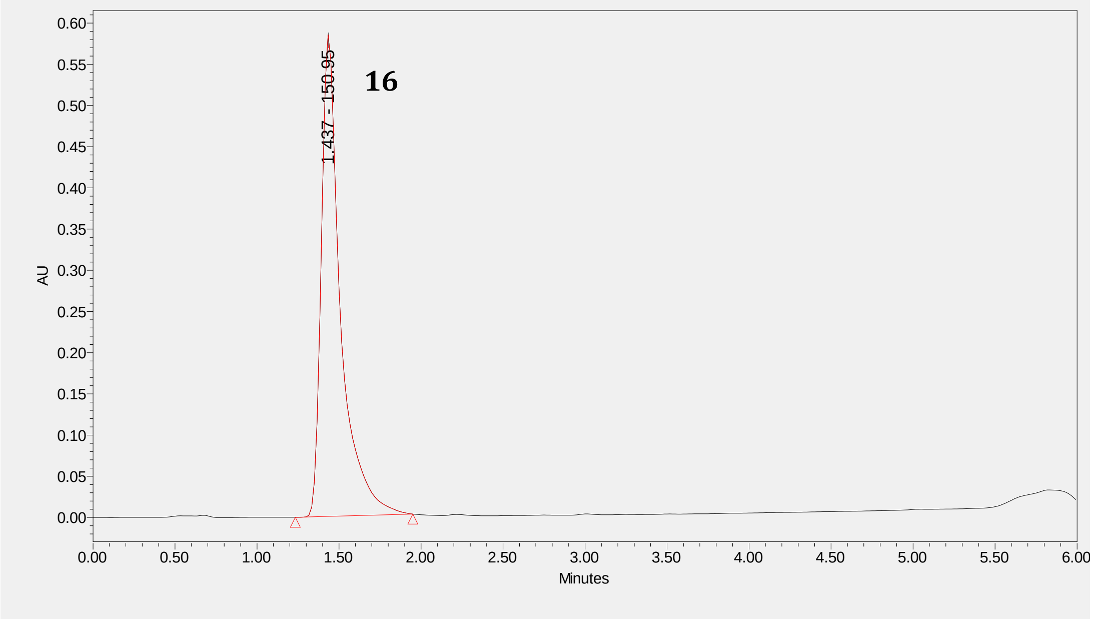


Figure S98**:** UPLC-QDa trace (method 2) of 4-hydroxyPhglyoxal **16**.

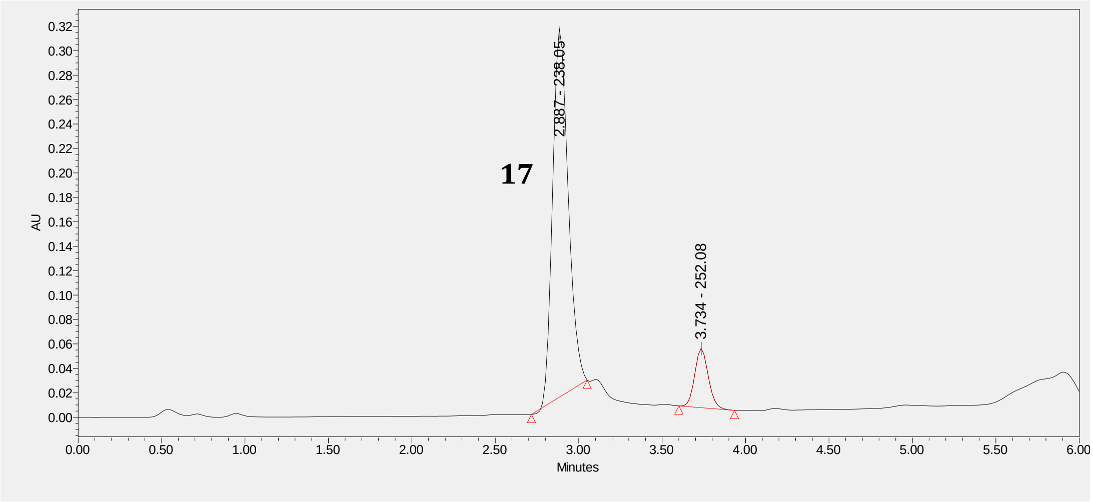


Figure S99**:** UPLC-QDa trace (method 2) of 4-MorpholinoPhglyoxal **17** biotransformation at 0h.

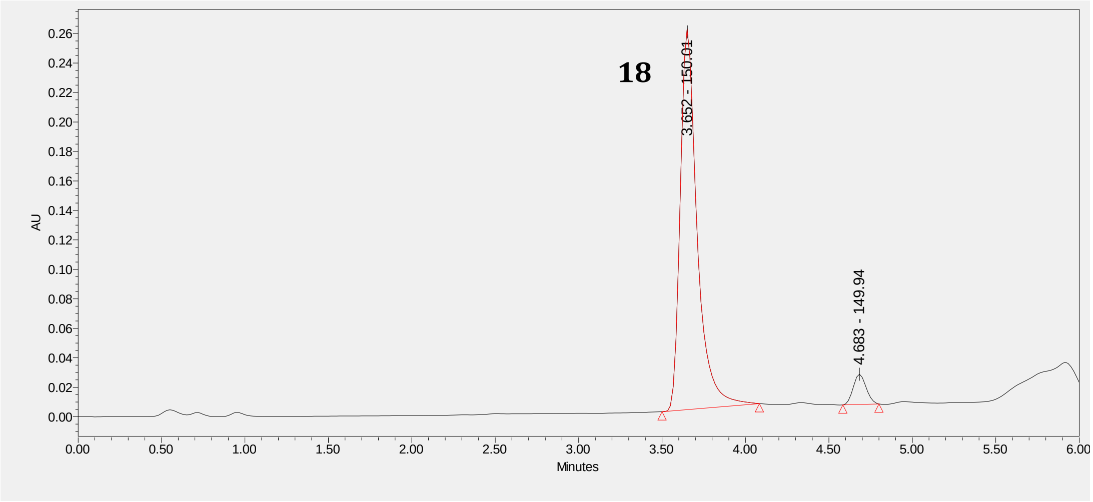


Figure S100**:** UPLC-QDa trace (method 2) of 2-$CF_3$Phglyoxal **18** biotransformation at 0h.

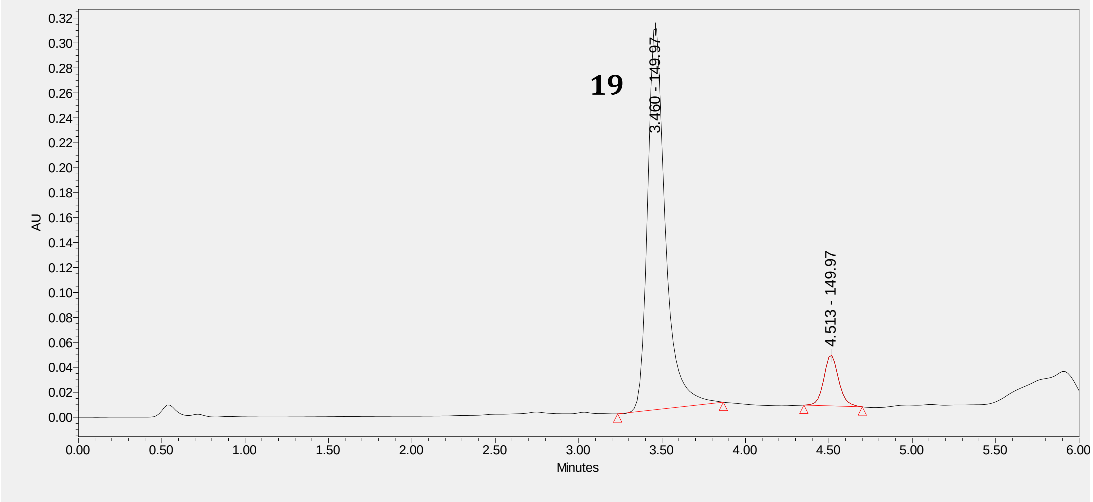


Figure S101**:** UPLC-QDa trace (method 2) of 4-AzidoPhglyoxal **19** biotransformation at 0h.

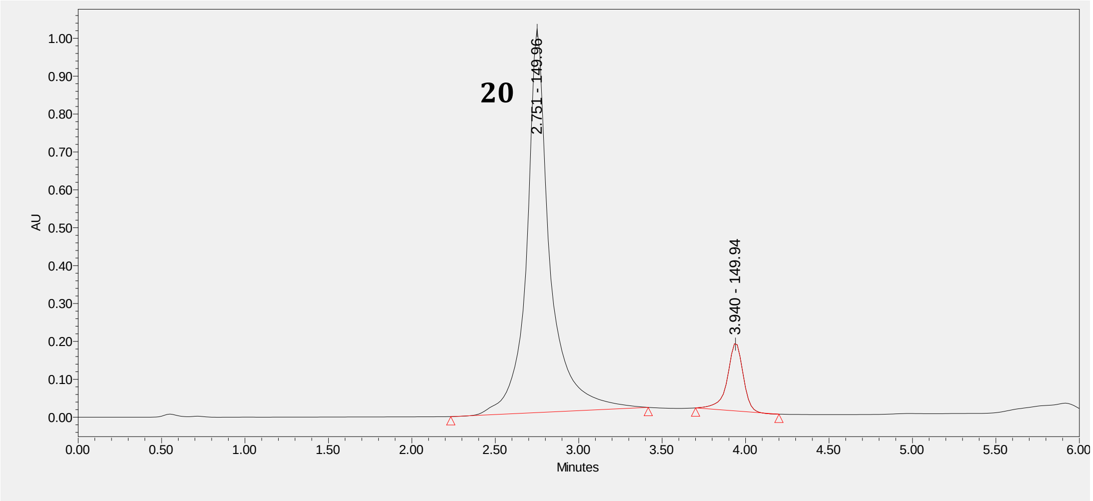


Figure S102: UPLC-QDa trace (method 2) of 2,4-DiFPhglyoxal **20** biotransformation at 0h.

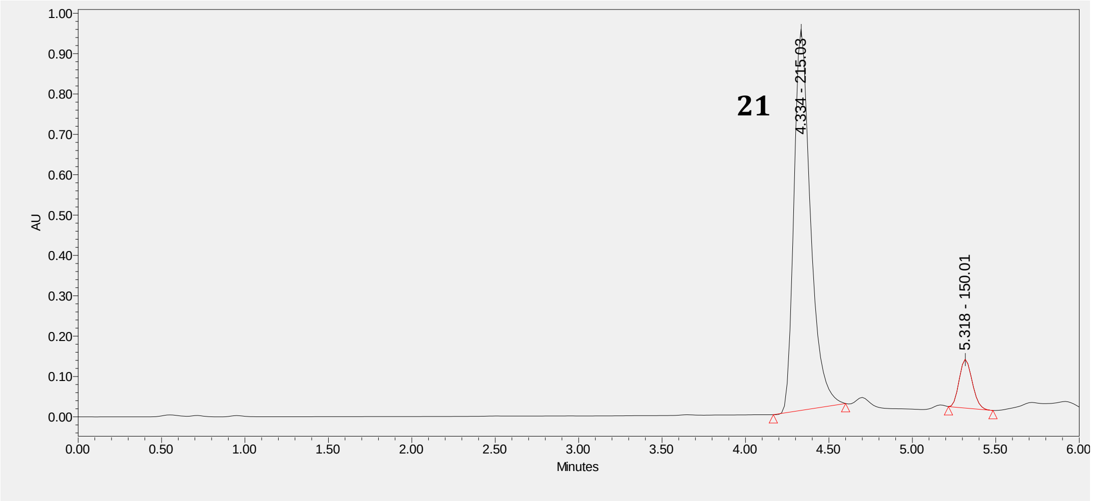


Figure S103: UPLC-QDa trace (method 2) of 6-MeONapthylglyoxal **21** biotransformation at 0h.

## *Example traces of biotransformations at 24h*

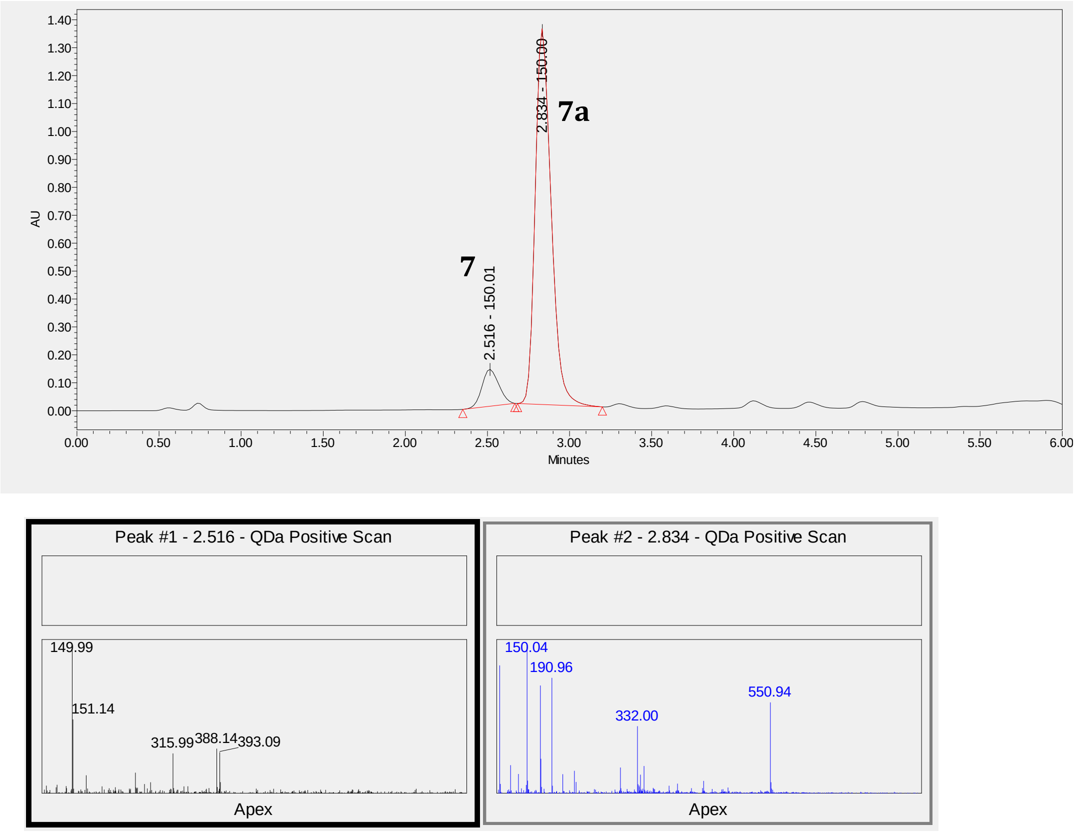


Figure S104: UPLC-QDa trace (method 2) of phenylglyoxal **7** biotransformation with HA **a** at 24h.

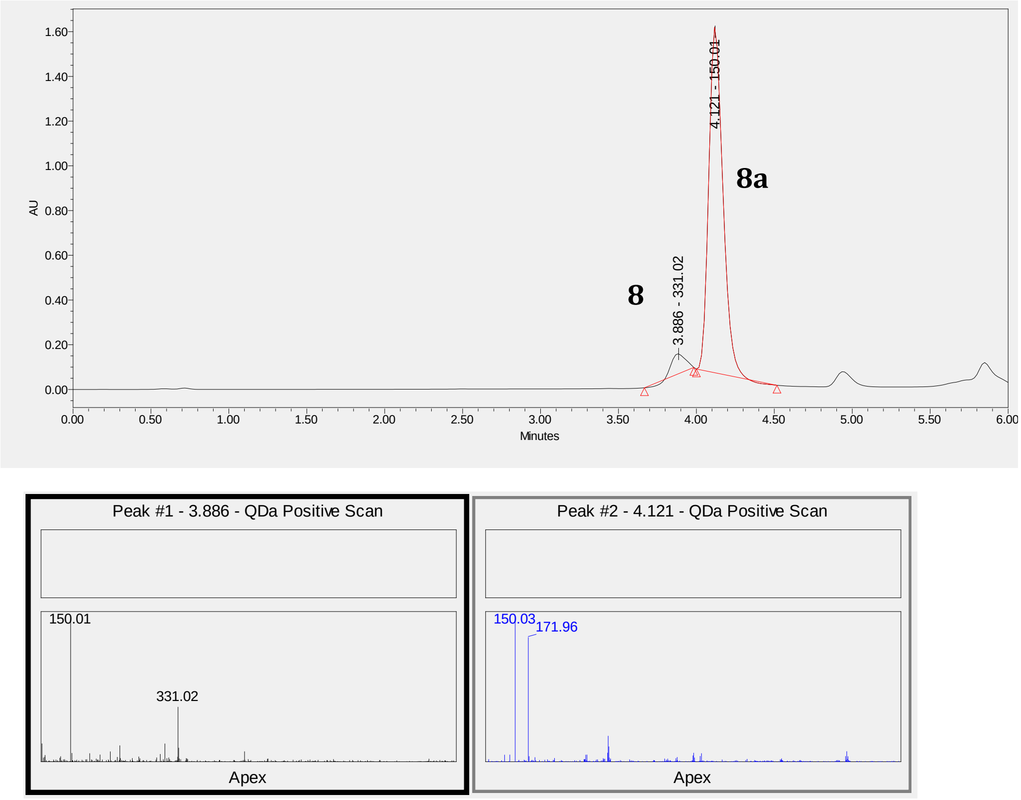


Figure S105: UPLC-QDa trace (method 2) of 4-BrPhglyoxal **8** biotransformation with HA **a** at 24h.

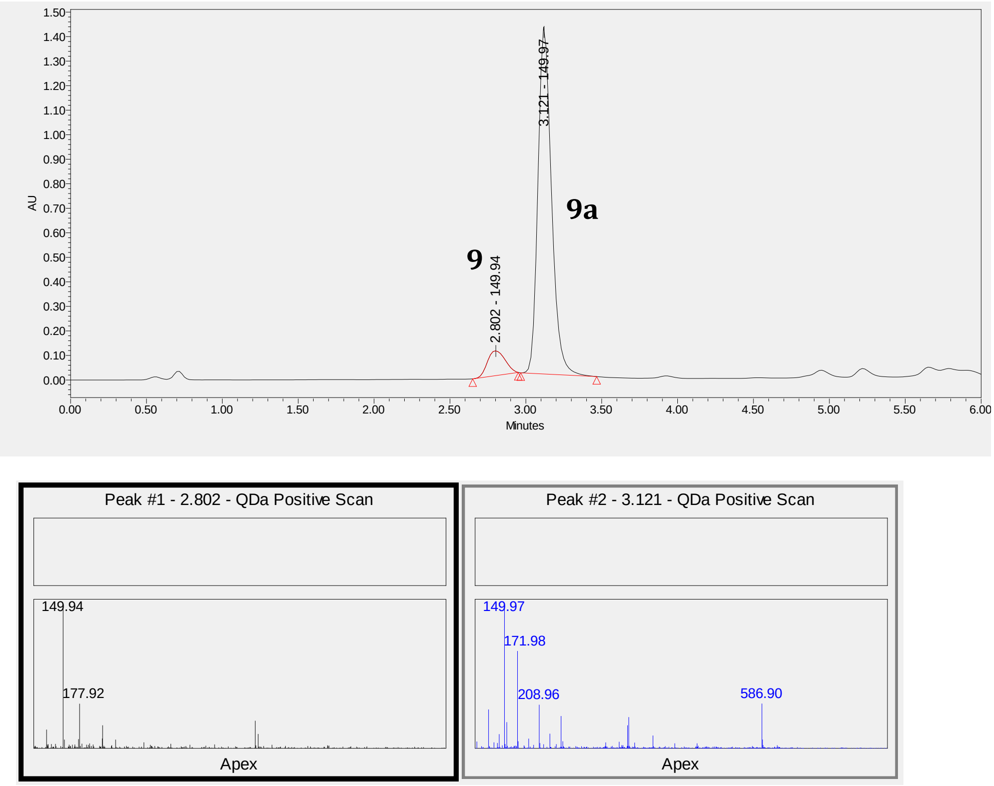


Figure S106: UPLC-QDa trace (method 2) of 4-FPhglyoxal **9** biotransformation with HA **a** at 24h.

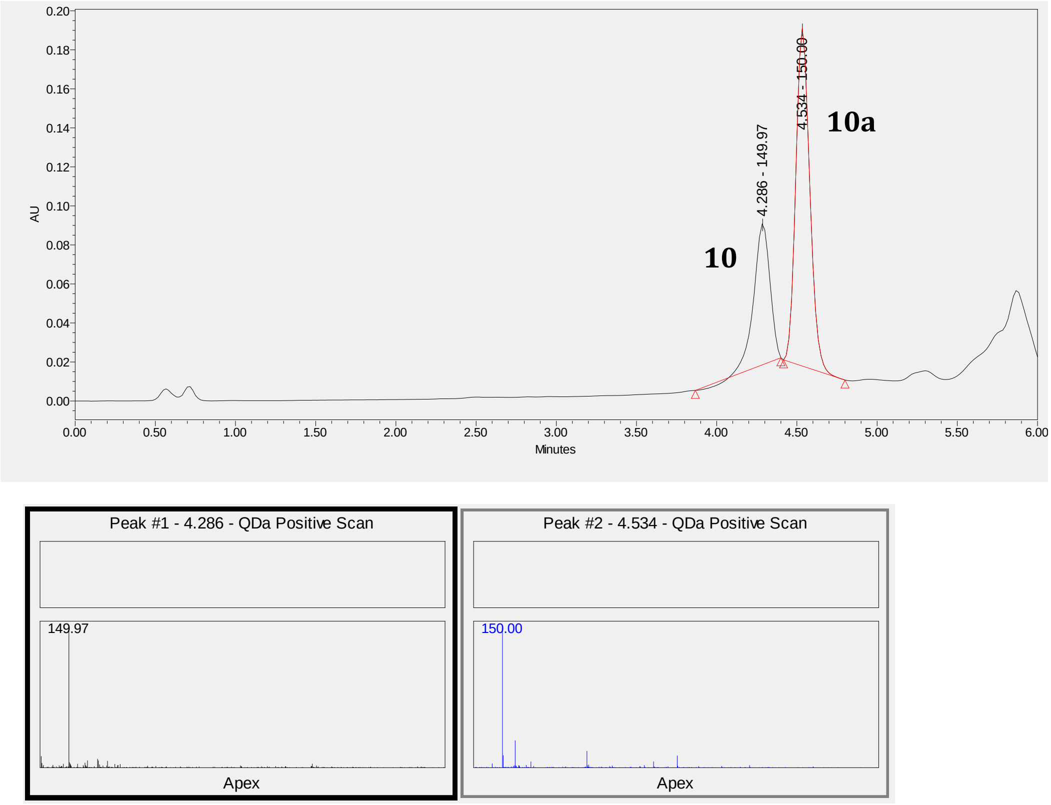


Figure S107: UPLC-QDa trace (method 2) of 4-$CF_3$Phglyoxal **10** biotransformation with HA **a** at 24h.

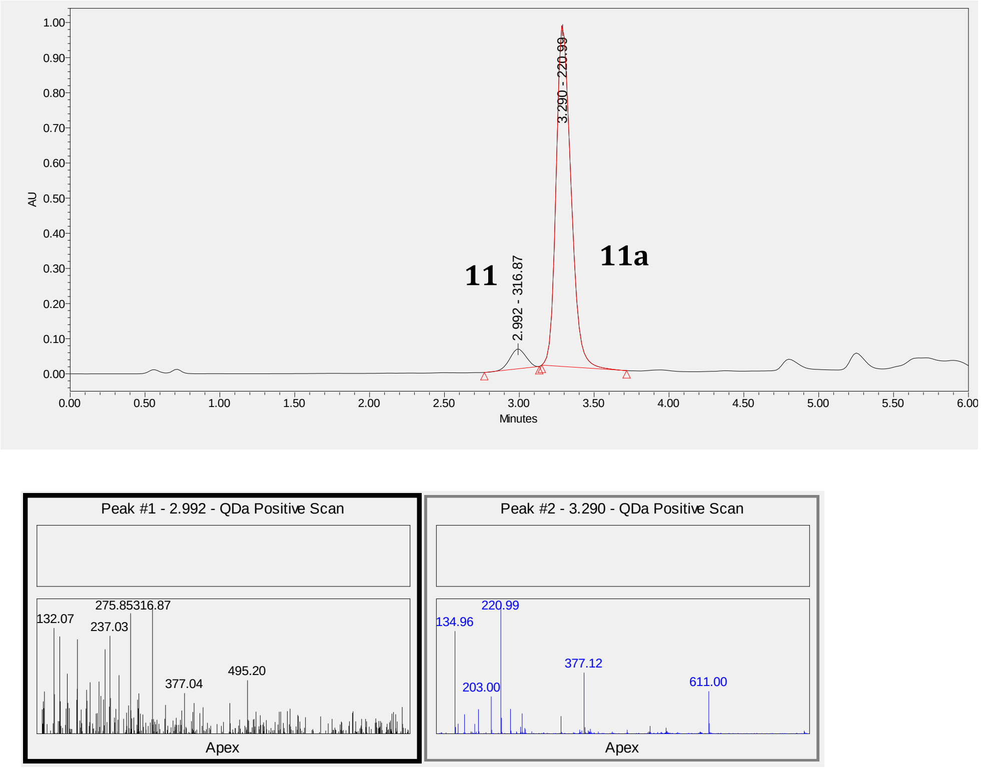


Figure S108: UPLC-QDa trace (method 2) of 3-OMePhglyoxal **11** biotransformation with HA **a** at 24h.

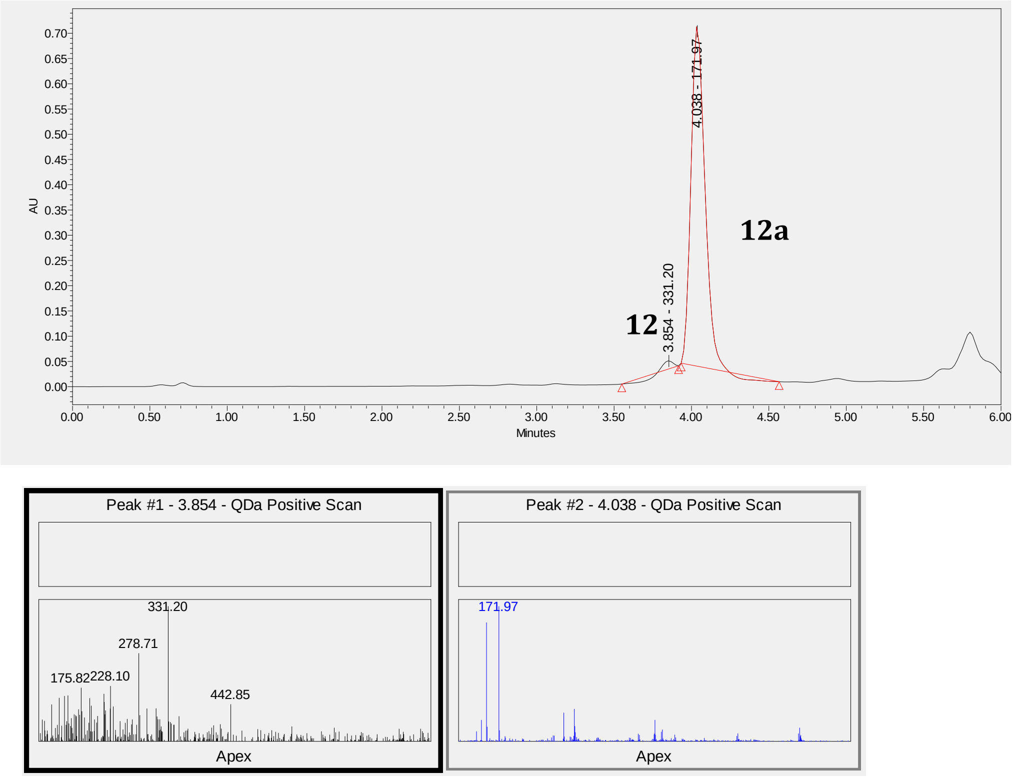


Figure S109: UPLC-QDa trace (method 2) of 3-BrPhglyoxal **12** biotransformation with HA **a** at 24h.

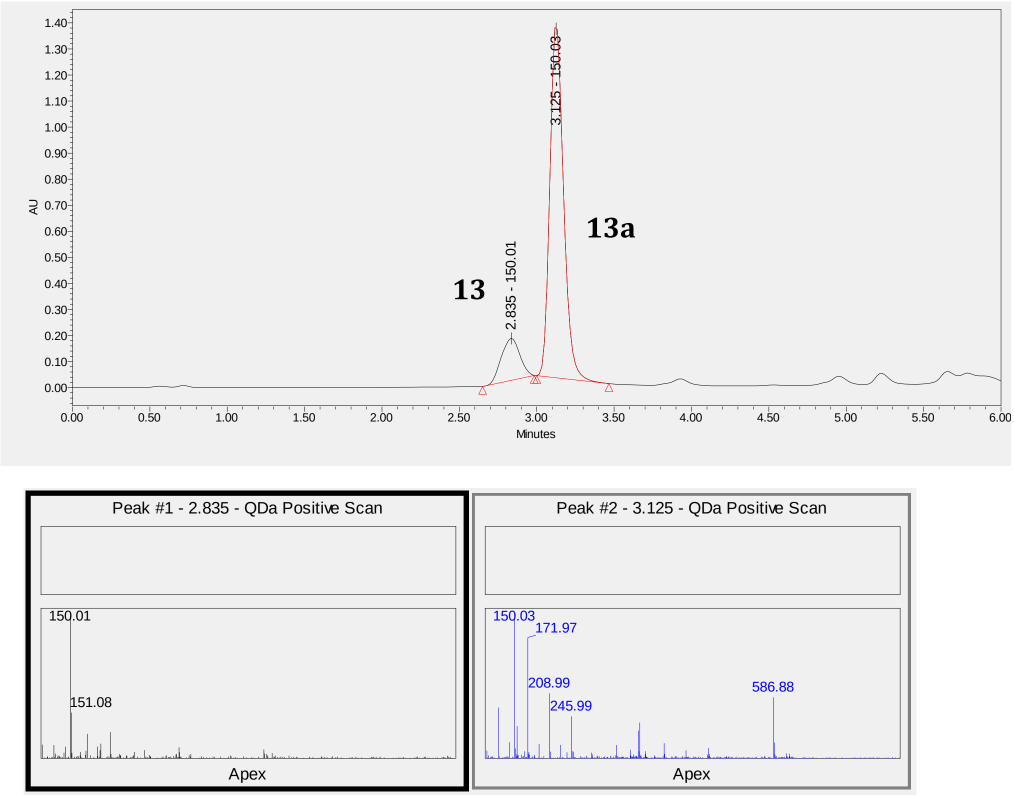


Figure S110: UPLC-QDa trace (method 2) of 3-ClPhglyoxal **13** biotransformation with HA **a** at 24h.

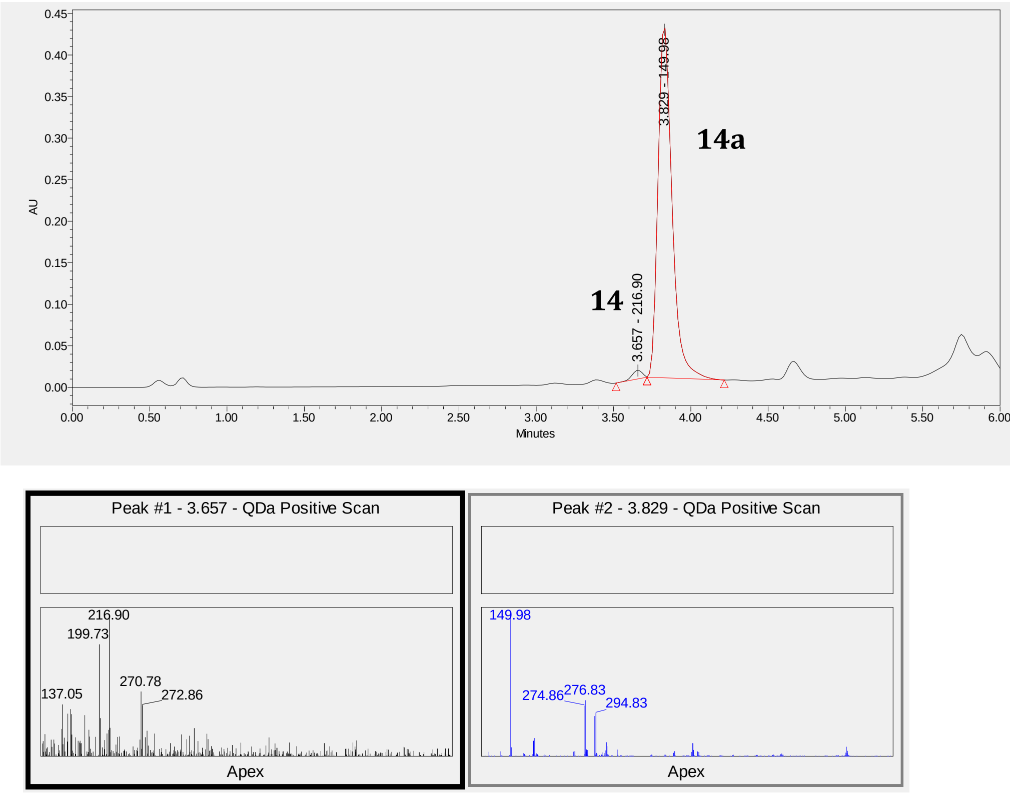


Figure S111: UPLC-QDa trace (method 2) of 5-BrThienylglyoxal **14** biotransformation with HA **a** at 24h.

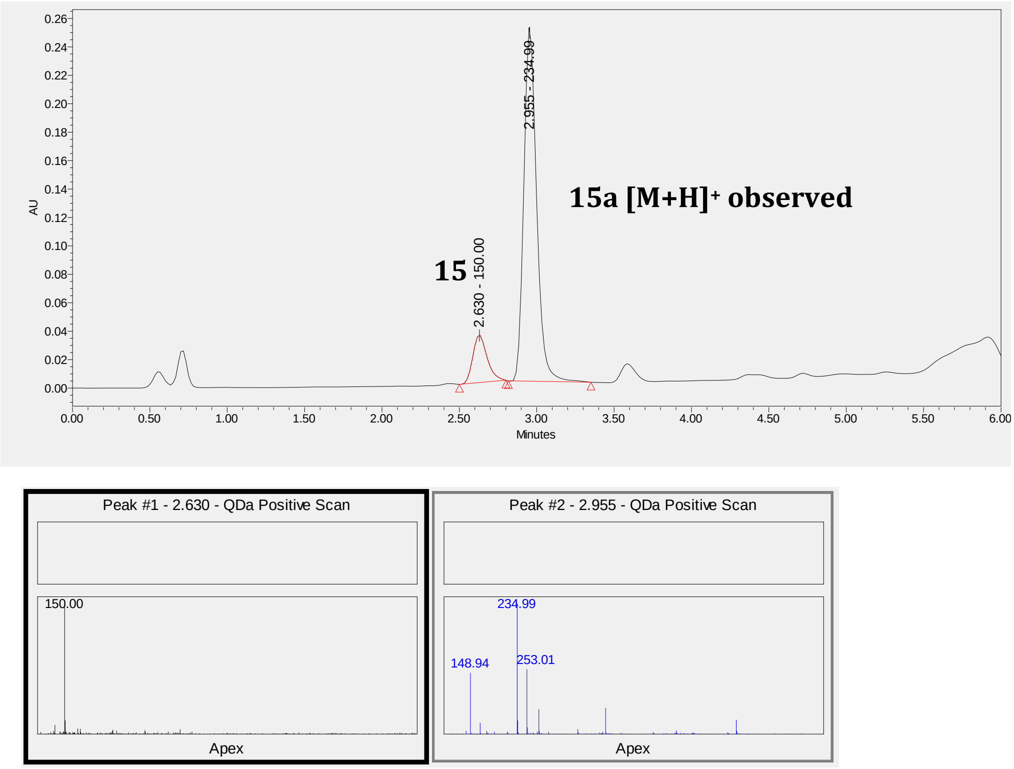


Figure S112: UPLC-QDa trace (method 2) of 3,4-methylenedioxyPhglyoxal **15** biotransformation with HA **a** at 24h.

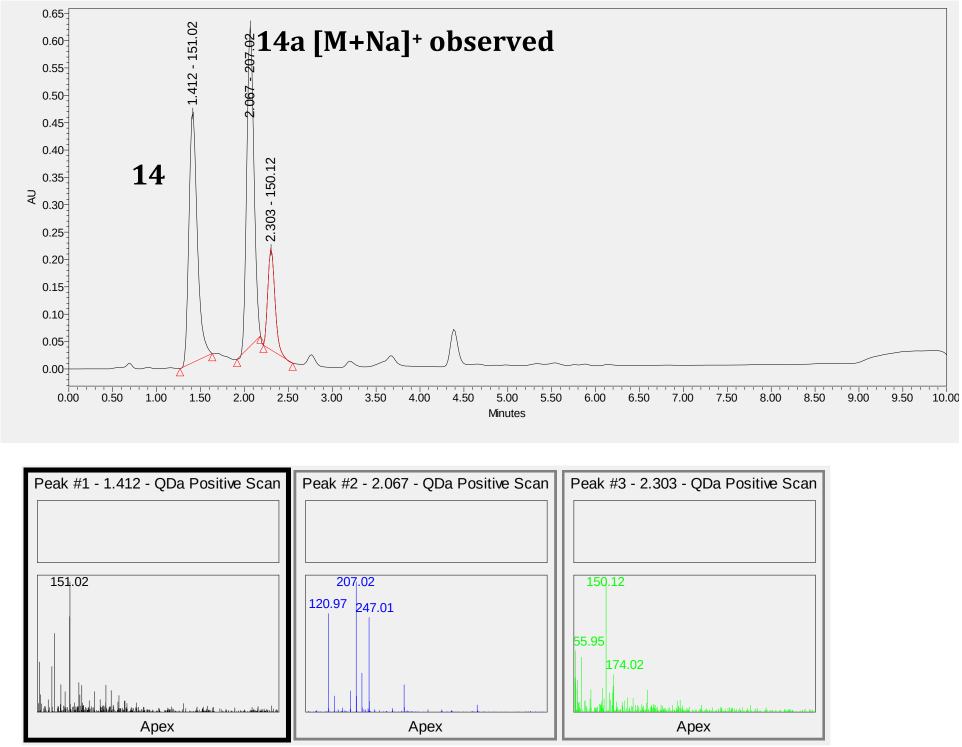


Figure S113: UPLC-QDa trace (method 1) of 4-OHPhglyoxal **14** biotransformation with HA **a** at 24h.

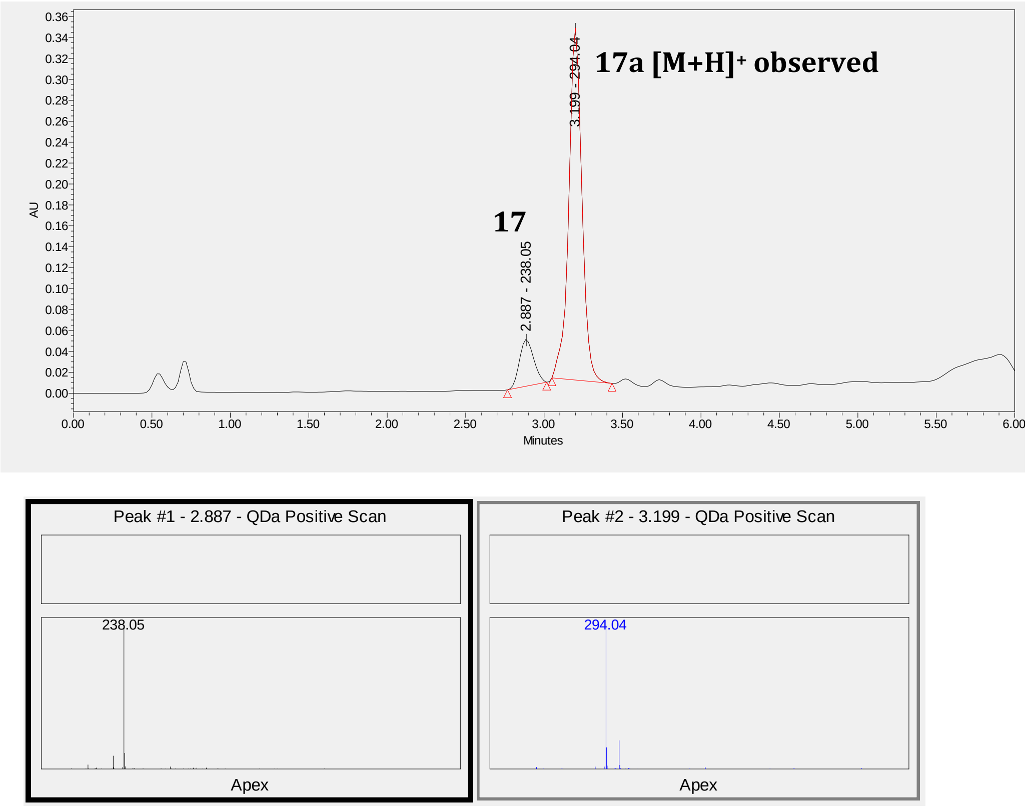


Figure S114: UPLC-QDa trace (method 2) of 4-MorpholinoPhglyoxal **17** biotransformation with HA **a** at 24h.

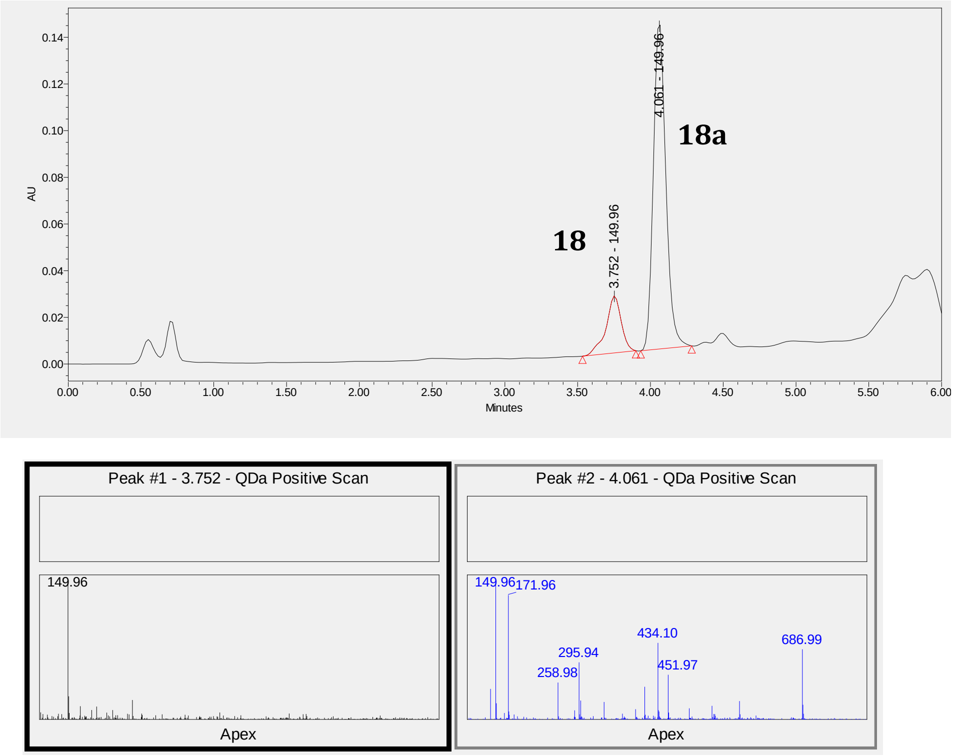


Figure S115: UPLC-QDa trace (method 2) of 2-$CF_3$Phglyoxal **18** biotransformation with HA **a** at 24h.

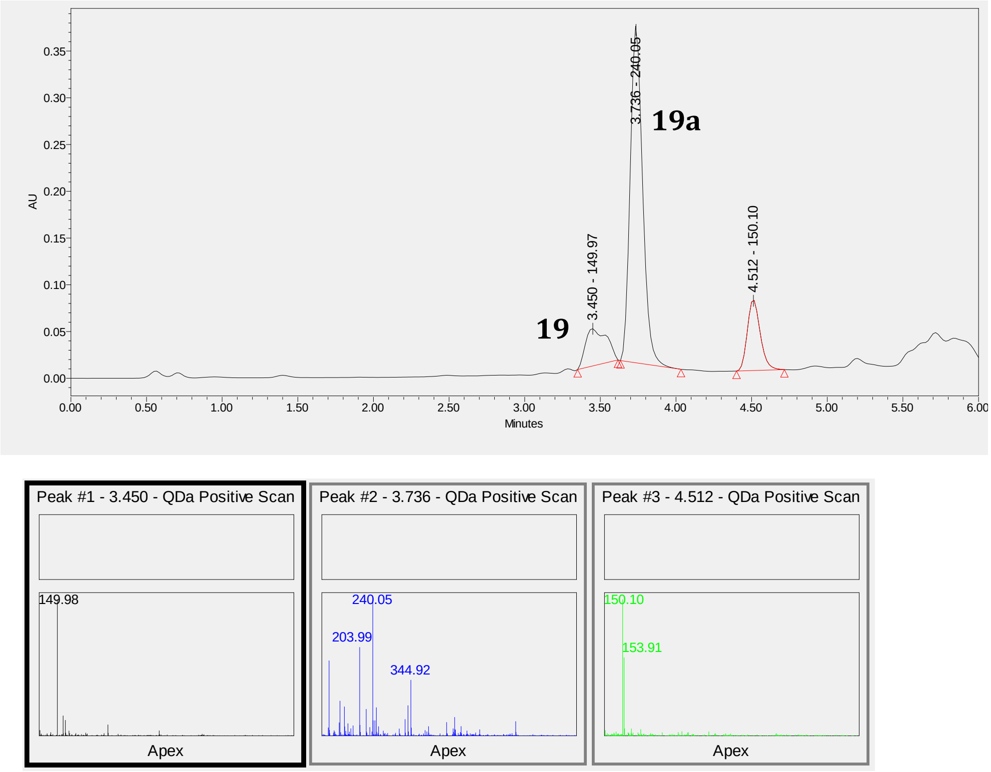


Figure S116**:** UPLC-QDa trace (method 2) of 4-AzidoPhglyoxal **19** biotransformation with HA **a** at 24h.

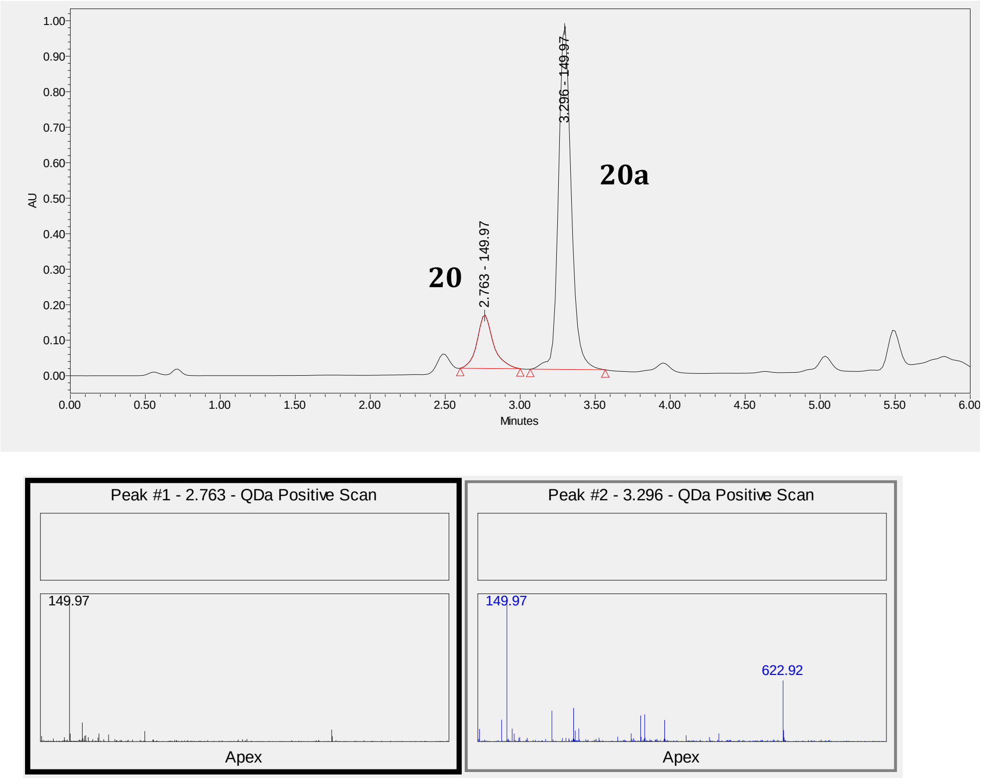


Figure S117**:** UPLC-QDa trace (method 2) of 2,4-DiFPhglyoxal **20** biotransformation with HA **a** at 24h.

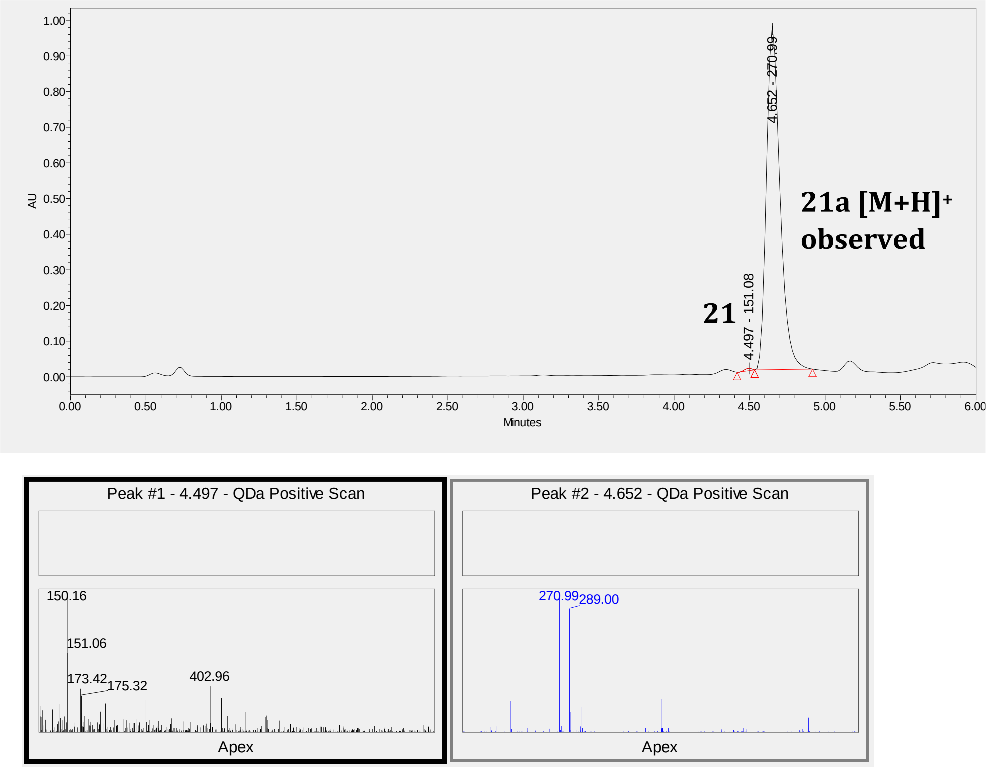


Figure S118: UPLC-QDa trace (method 2) of 6-MeONapthylglyoxal **21** biotransformation with HA **a** at 24h.

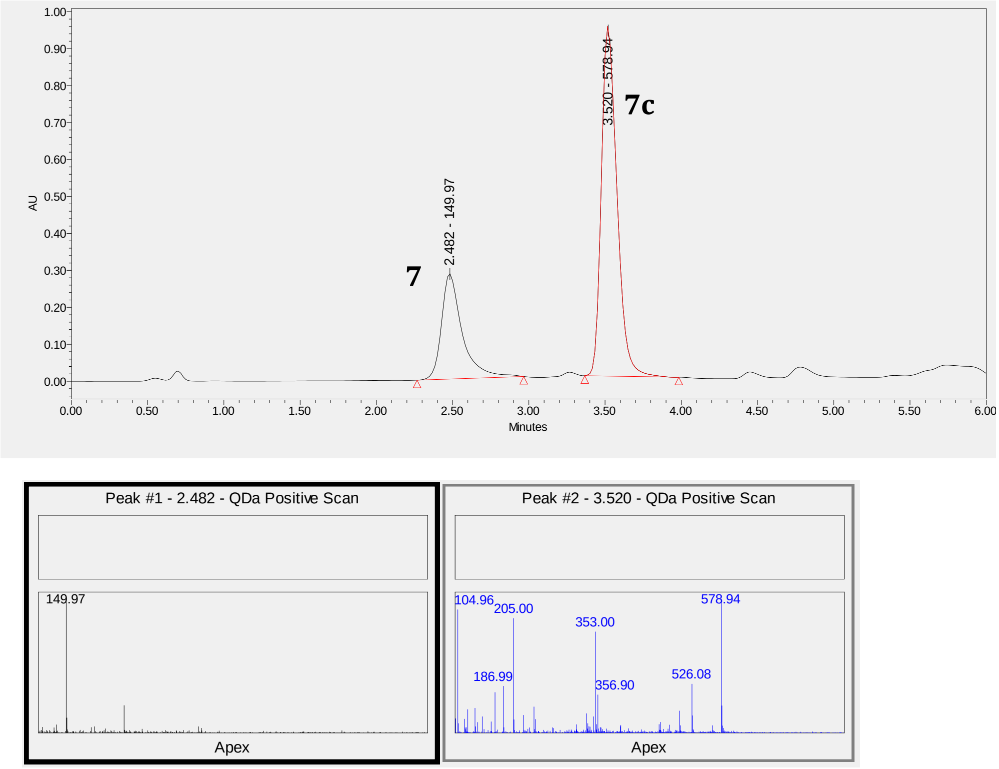


Figure S119: UPLC-QDa trace (method 2) of phenylglyoxal **7** biotransformation with HB **c** at 24h.

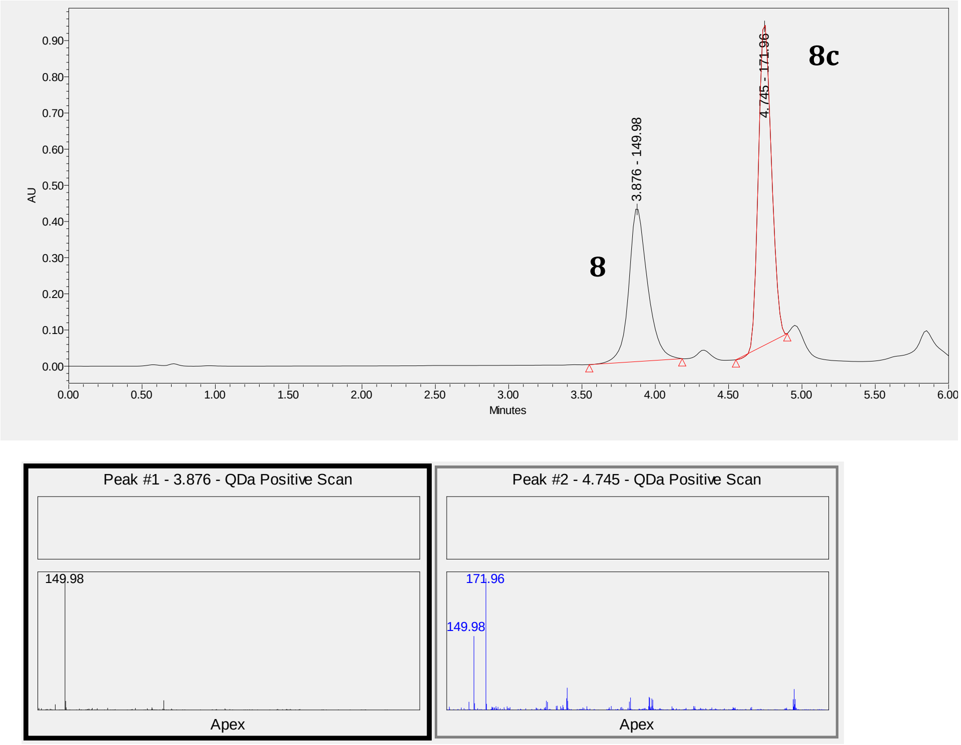


Figure S120: UPLC-QDa trace (method 2) of 4-BrPhglyoxal **8** biotransformation at 24h.

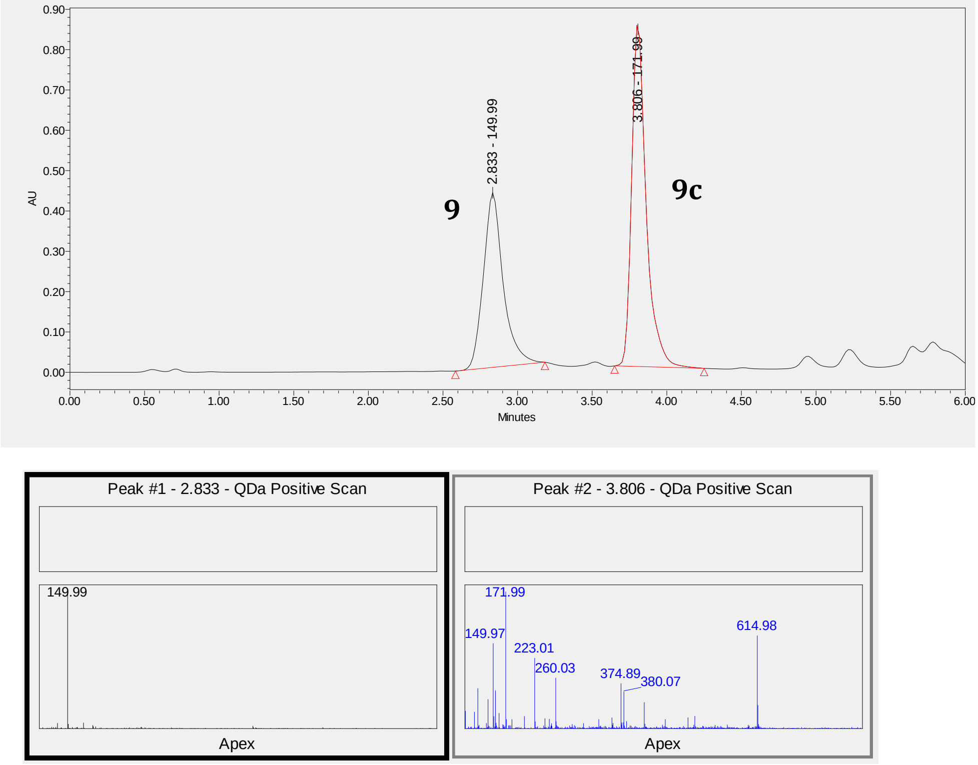


Figure S121: UPLC-QDa trace (method 2) of 4-FPhglyoxal **9** biotransformation with HB **c** at 24h.

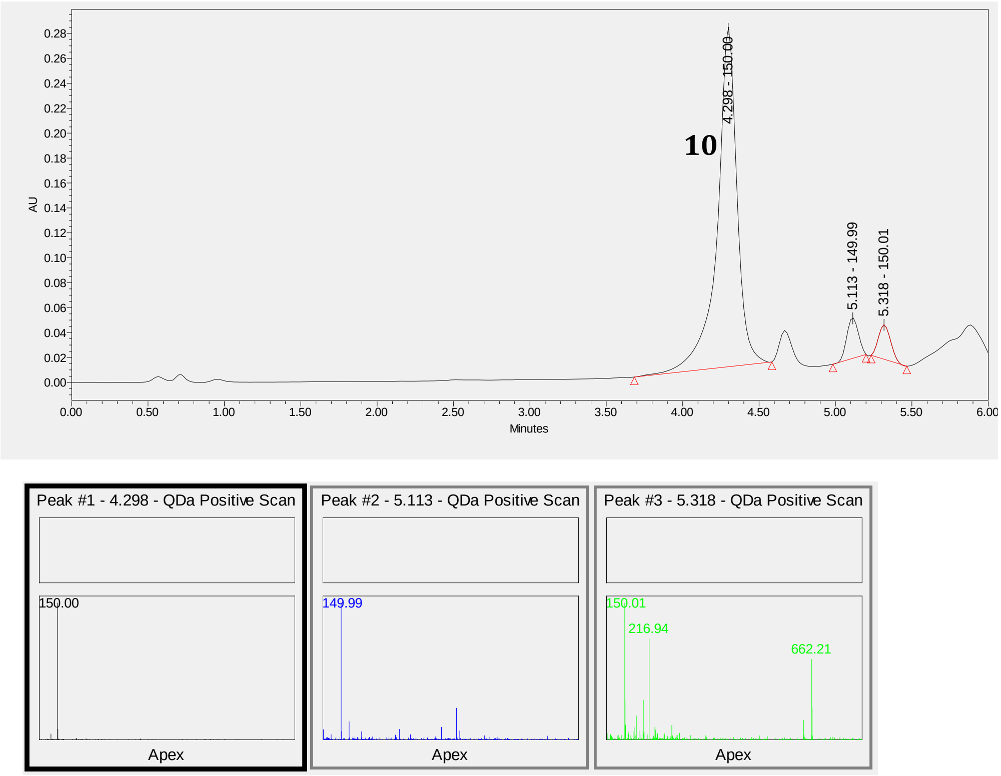


Figure S122: UPLC-QDa trace (method 2) of 4-$CF_3$Phglyoxal **10** biotransformation with HB **c** at 24h.

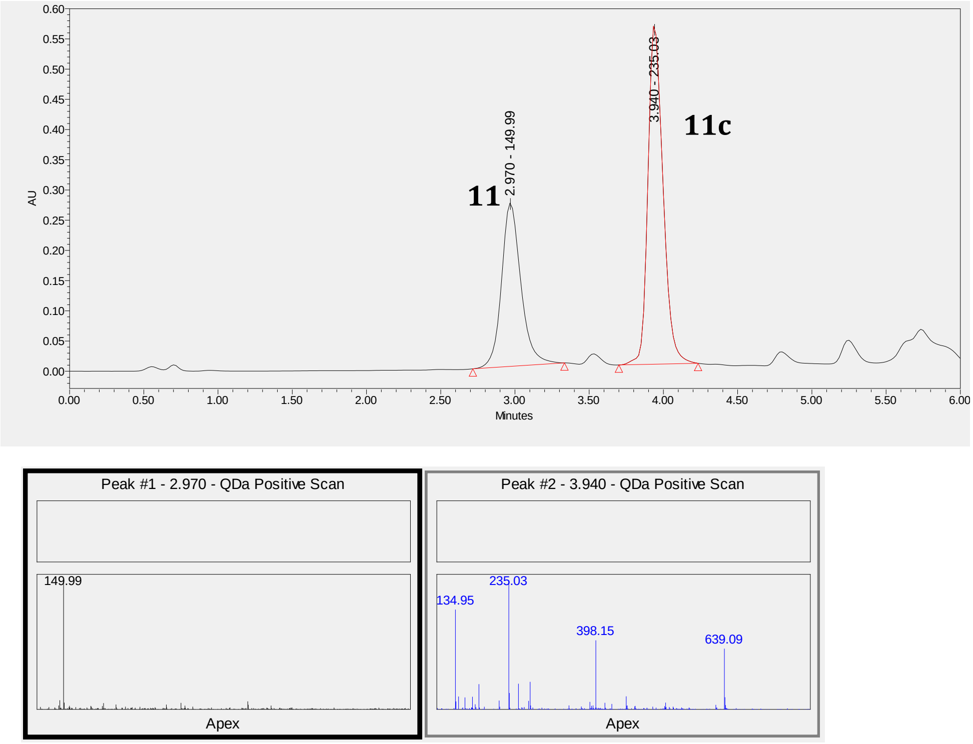


Figure S123: UPLC-QDa trace (method 2) of 3-OMePhglyoxal **11** biotransformation with HB **c** at 24h.

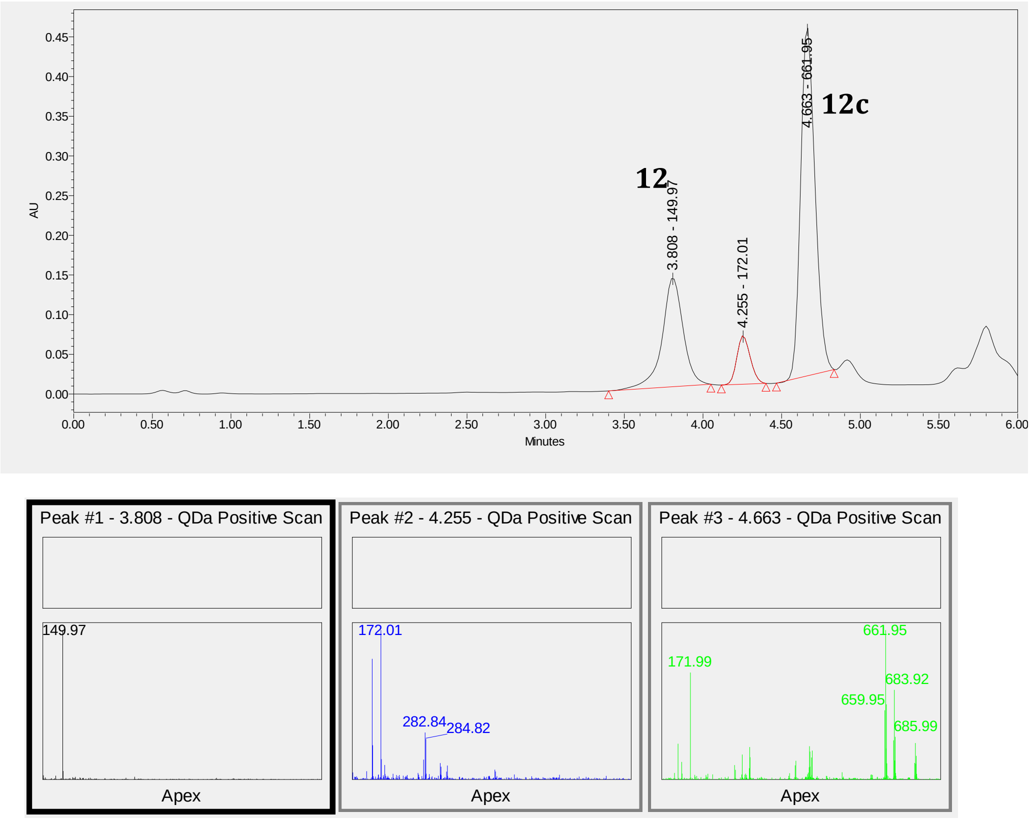


Figure S124: UPLC-QDa trace (method 2) of 3-BrPhglyoxal **12** biotransformation with HB **c** at 24h.

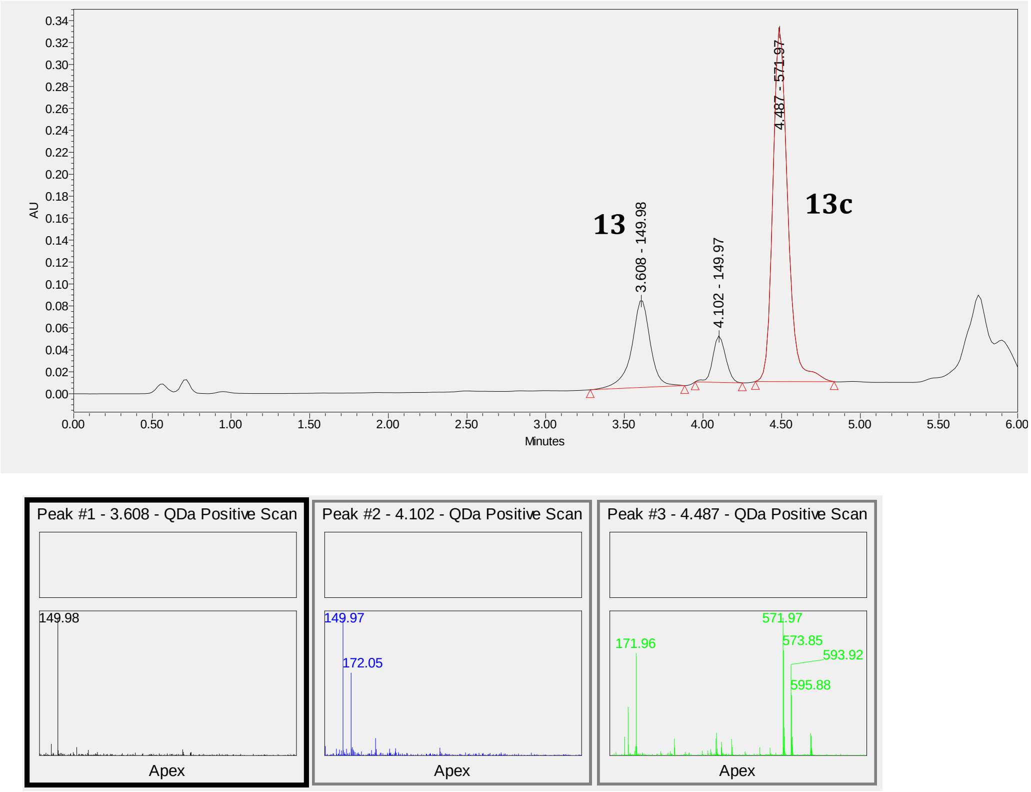


Figure S125: UPLC-QDa trace (method 2) of 3-ClPhglyoxal **13** biotransformation with HB **c** at 24h.

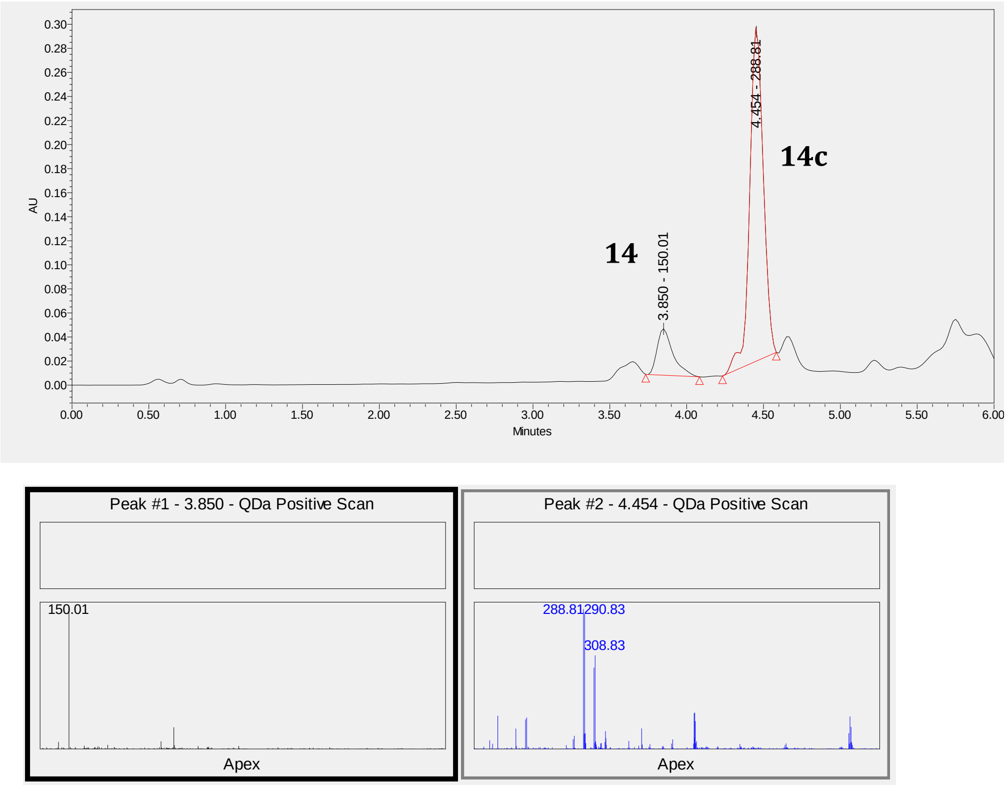


Figure S126: UPLC-QDa trace (method 2) of 5-BrThienylglyoxal **14** biotransformation with HB **c** at 24h.

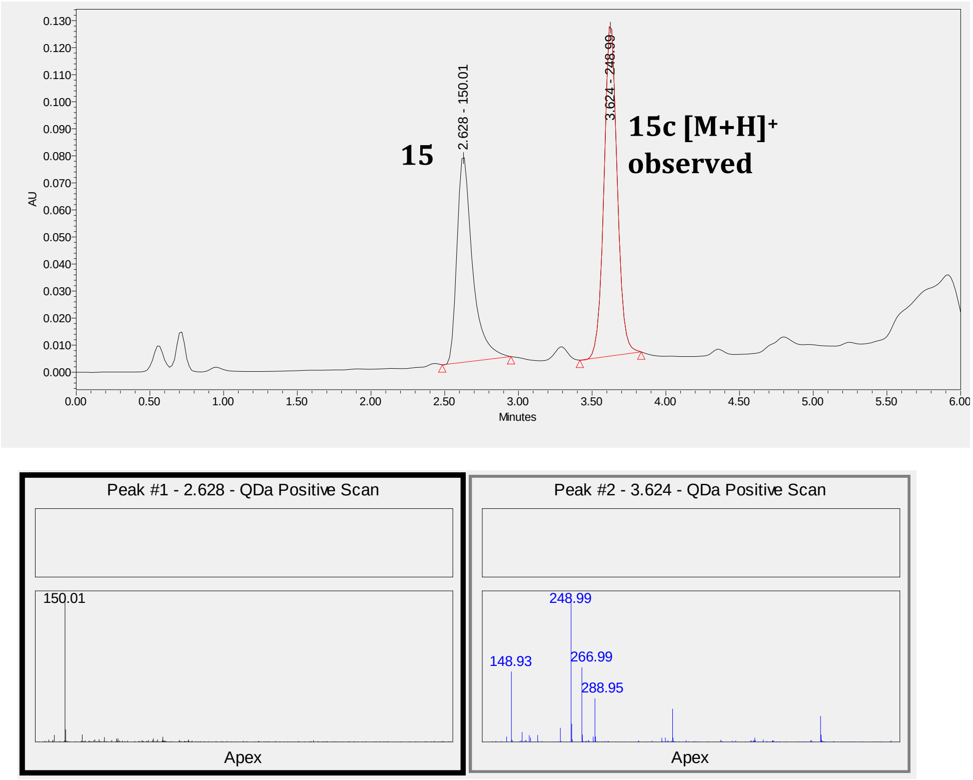


Figure S127: UPLC-QDa trace (method 2) of 3,4-methylenedioxyPhglyoxal **15** biotransformation with HB **c** at 24h.

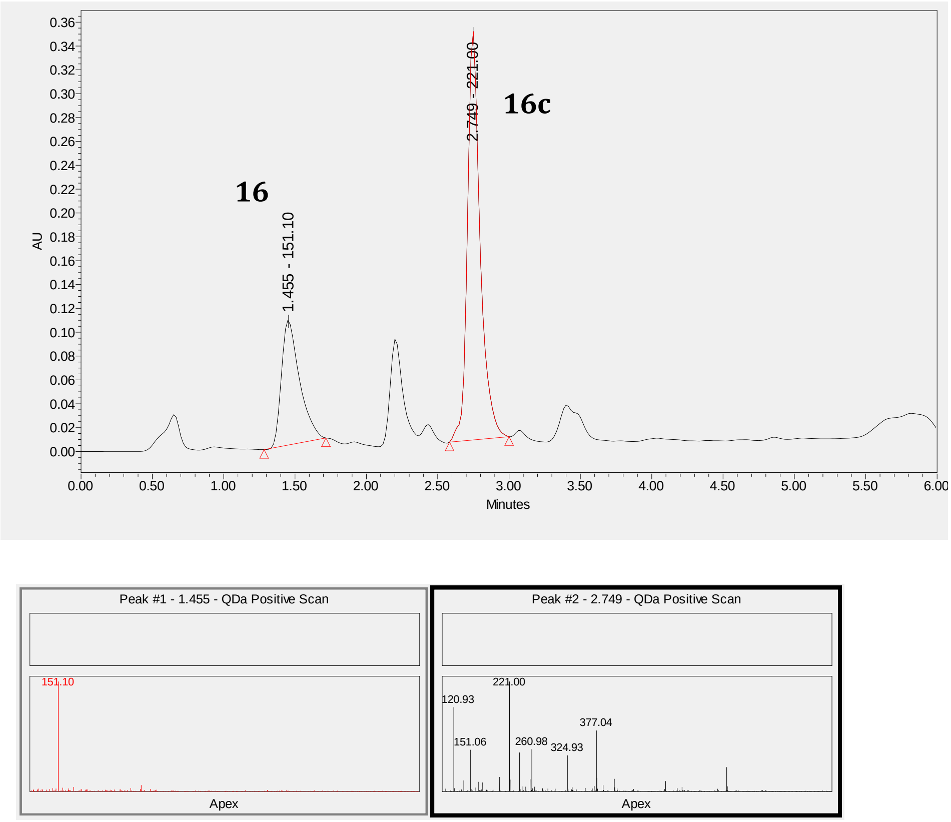


Figure S128: UPLC-QDa trace (method 2) of 4-hydroxyPhglyoxal **16** biotransformation with HB **c** at 24h.

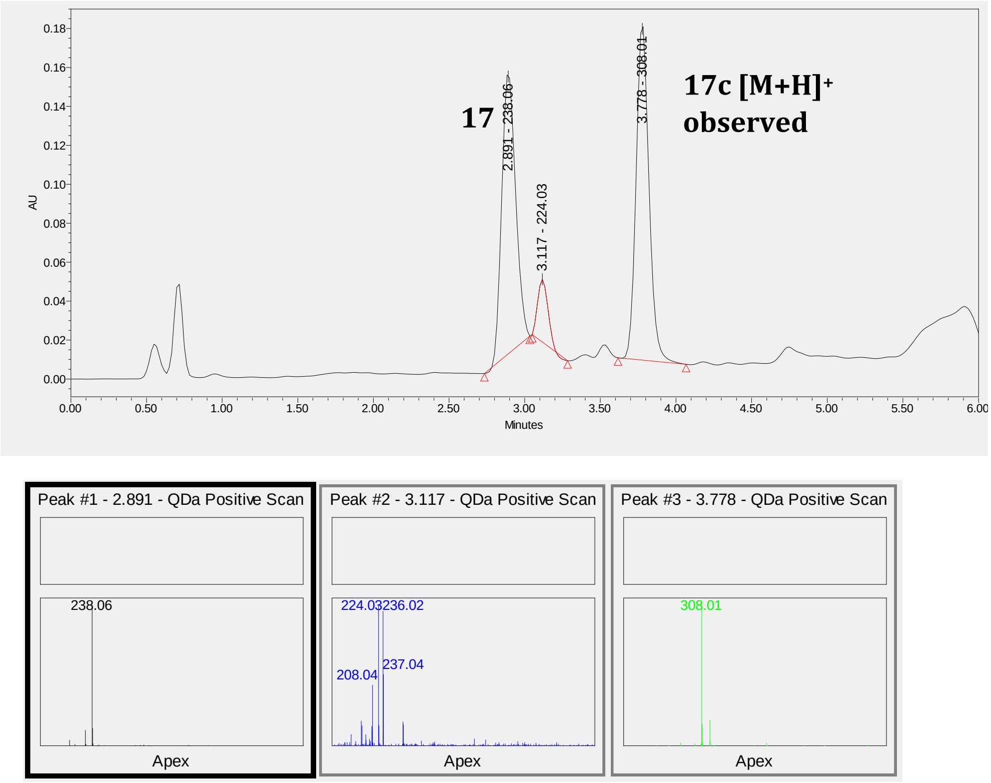


Figure S129**:** UPLC-QDa trace (method 2) of 4-MorpholinoPhglyoxal **17** biotransformation with HB **c** at 24h.

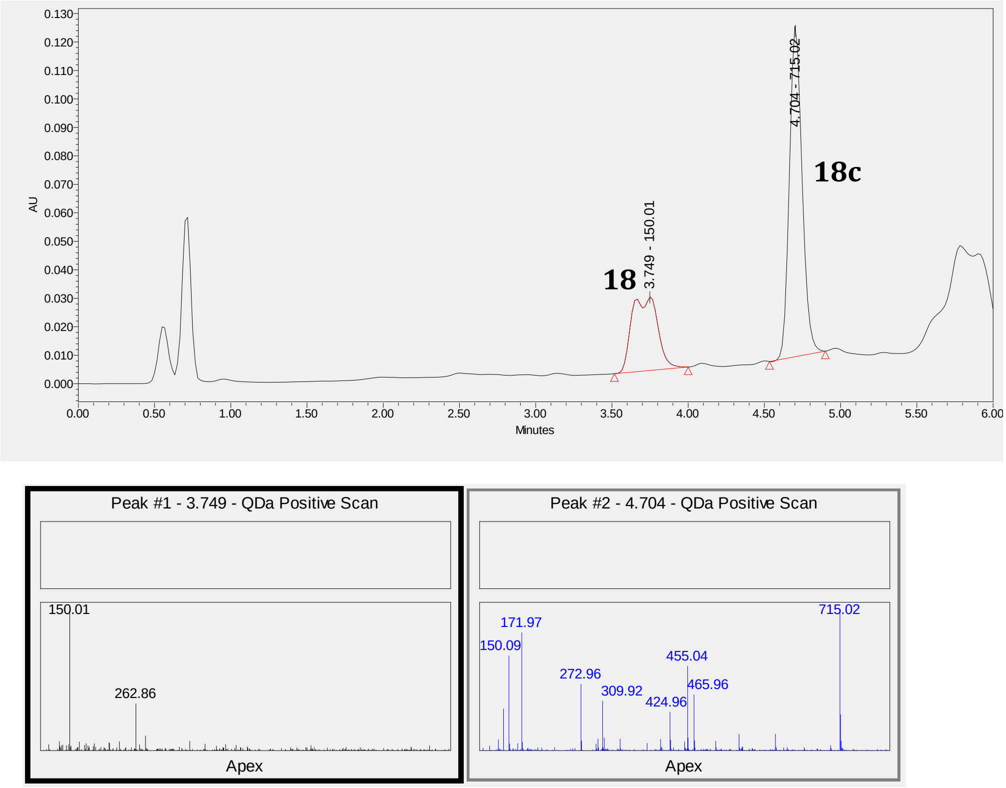


Figure S130: UPLC-QDa trace (method 2) of 2-$CF_3$Phglyoxal **18** biotransformation with HB **c** at 24h.

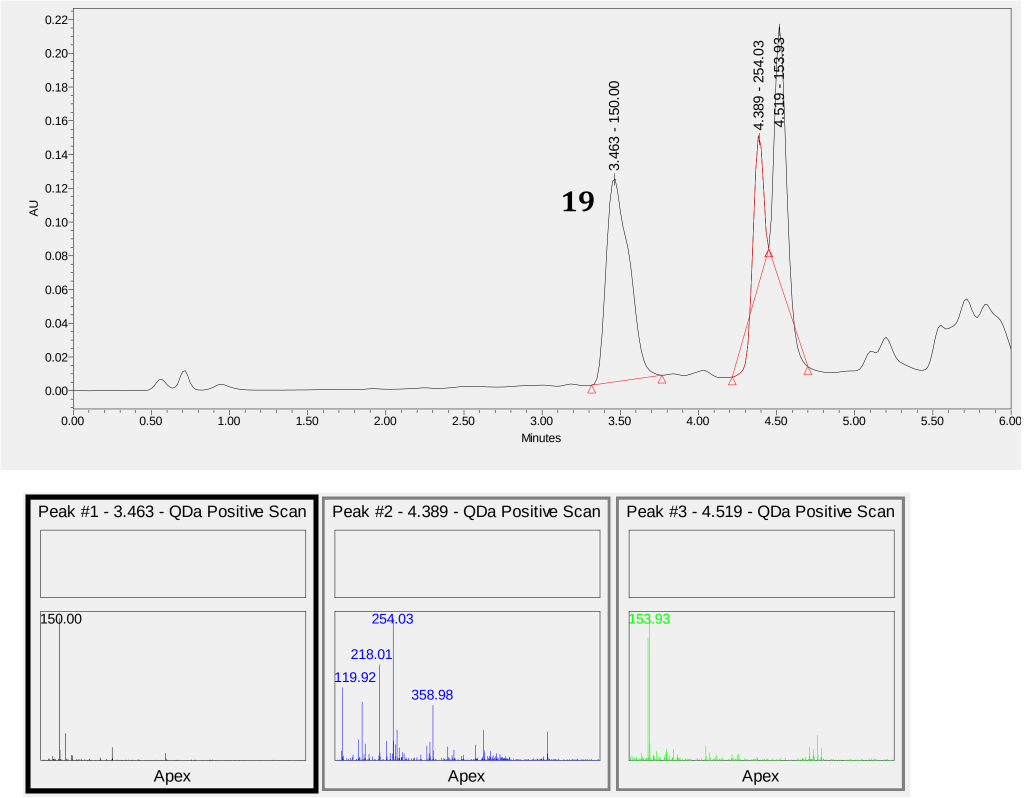


Figure S131: UPLC-QDa trace (method 2) of 4-AzidoPhglyoxal **19** biotransformation with HB **c** at 24h.

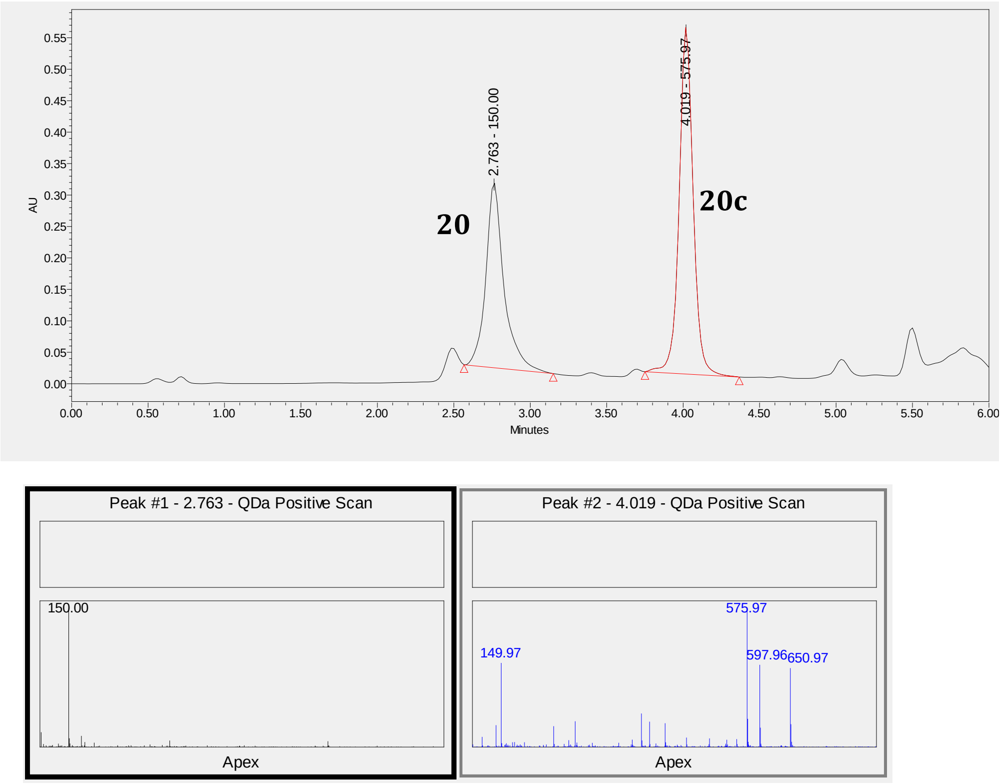


Figure S132: UPLC-QDa trace (method 2) of 2,4-DiFPhglyoxal **20** biotransformation with HB **c** at 24h.

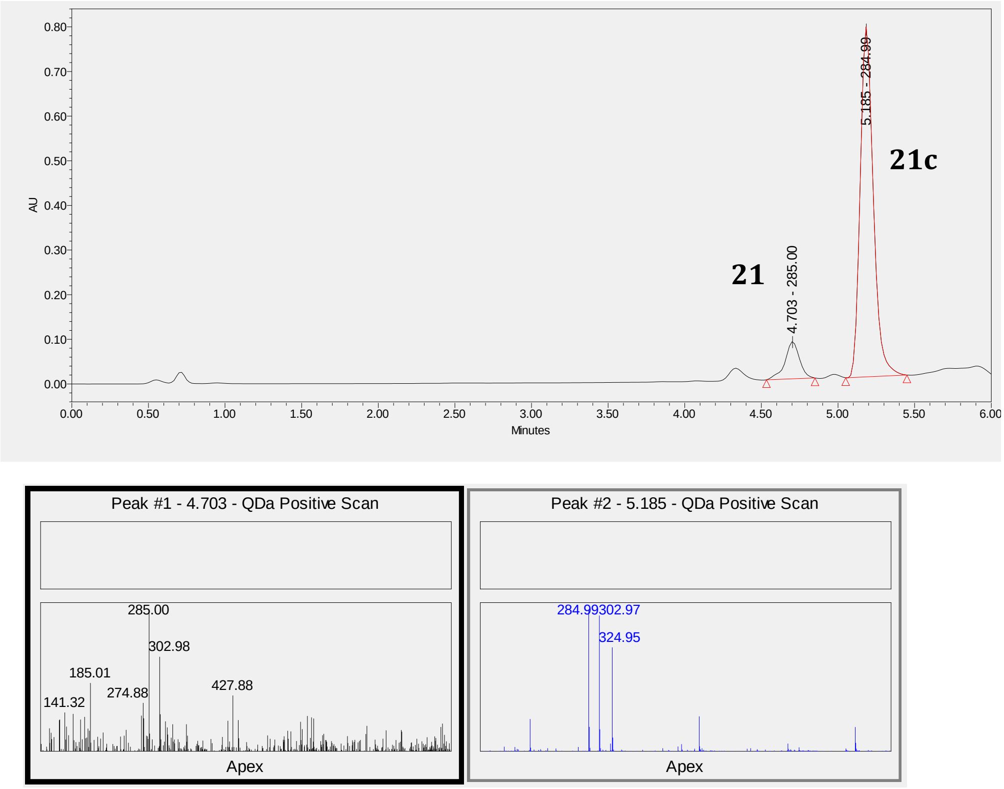


Figure S133: UPLC-QDa trace (method 2) of 6-MeONapthylglyoxal **21** biotransformation with HB **c** at 24h.

*NMR spectra used to determine conversion in FSA biotransformations of **22** and **23***

*Glyoxylic acid **22***

*Screening reactions*

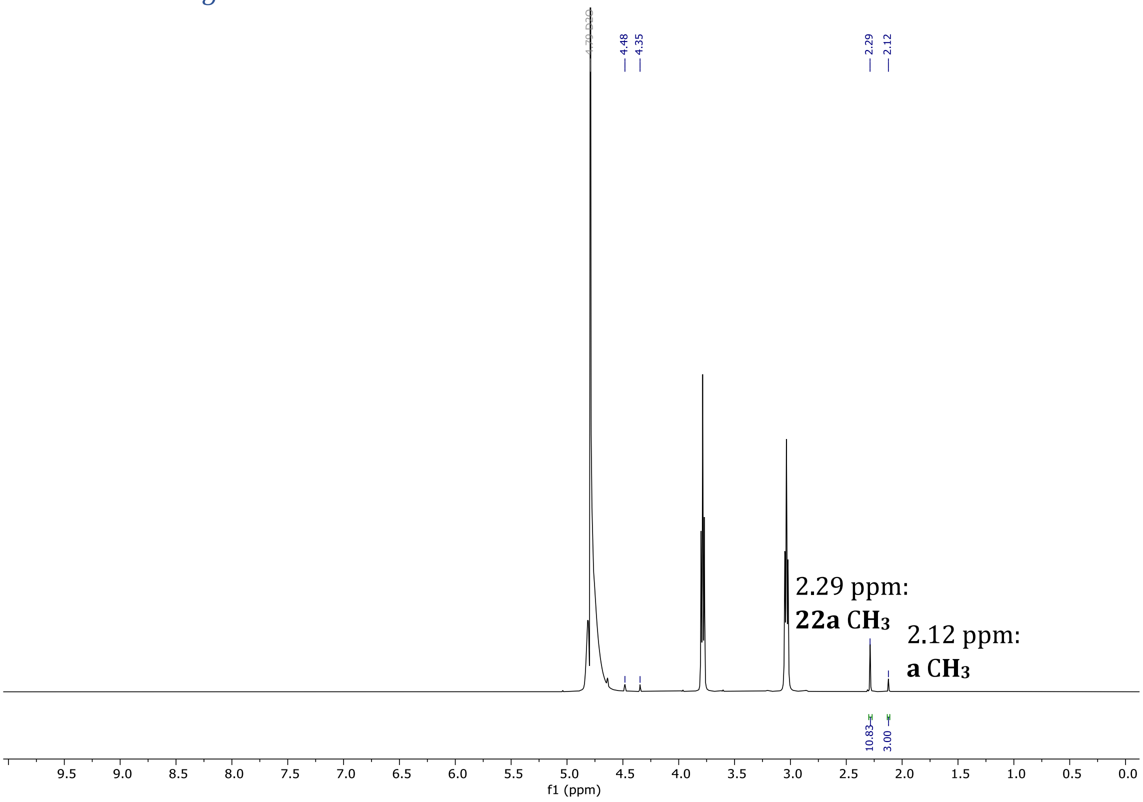


Figure S134: $^{1}H$ NMR spectra of an FSA biotransformation of glyoxylic acid **22** and **a** at 24h.

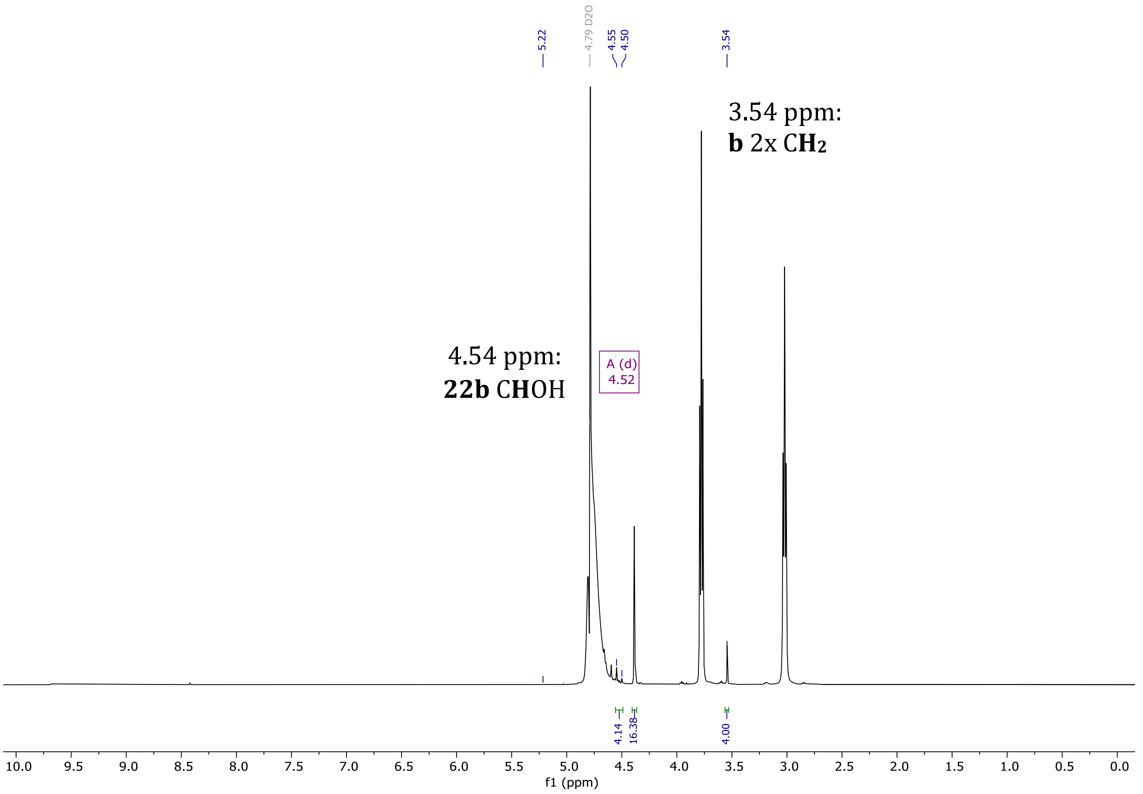


Figure S135: $^{1}H$ NMR spectra of an FSA biotransformation of glyoxylic acid **22** and **b** at 24h.

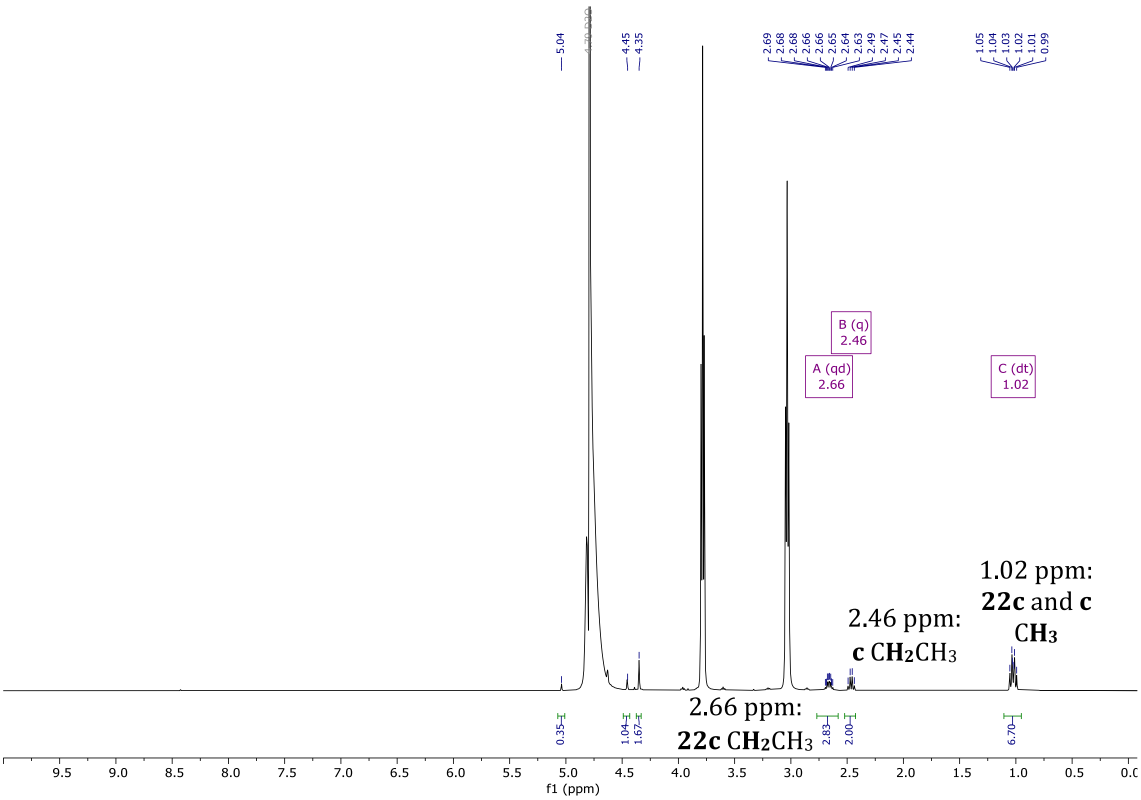


Figure S136: $^1$H NMR spectra of an FSA biotransformation of glyoxylic acid **22** and **c** at 24h.

*Time course at increased substrate loading*

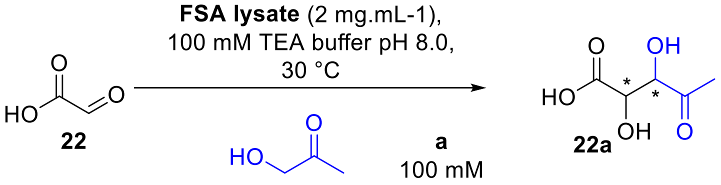


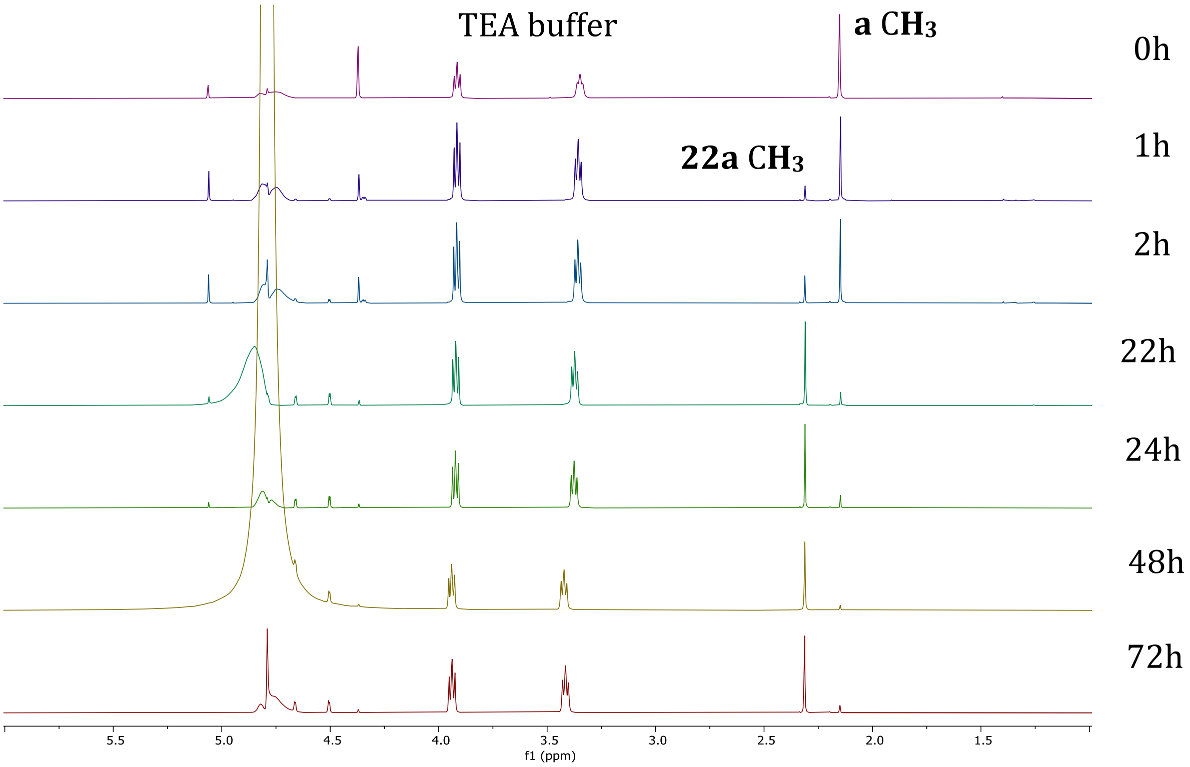


Figure S137: $^{1}$H NMR data for time course of the *Ec*FSA$_{wt}$ catalyzed reaction of hydroxyacetone **a** and glyoxylic acid **22**. Reaction performed at 100 mM substrate concentration with no DMSO.

## *Trifluoropyruvaldehyde **23***

### *Screening reactions*

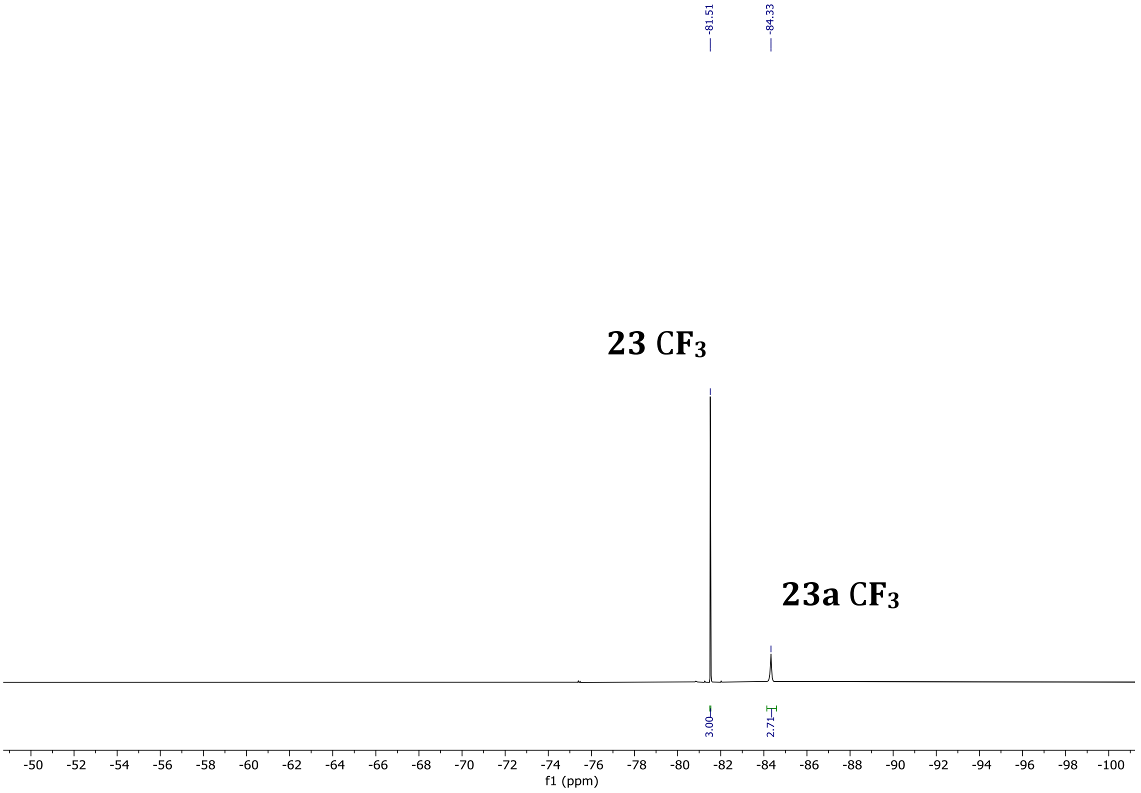


Figure S138: $^{19}$F NMR of an FSA biotransformation of trifluoropyruvaldehyde **23** and **a** at 24h.

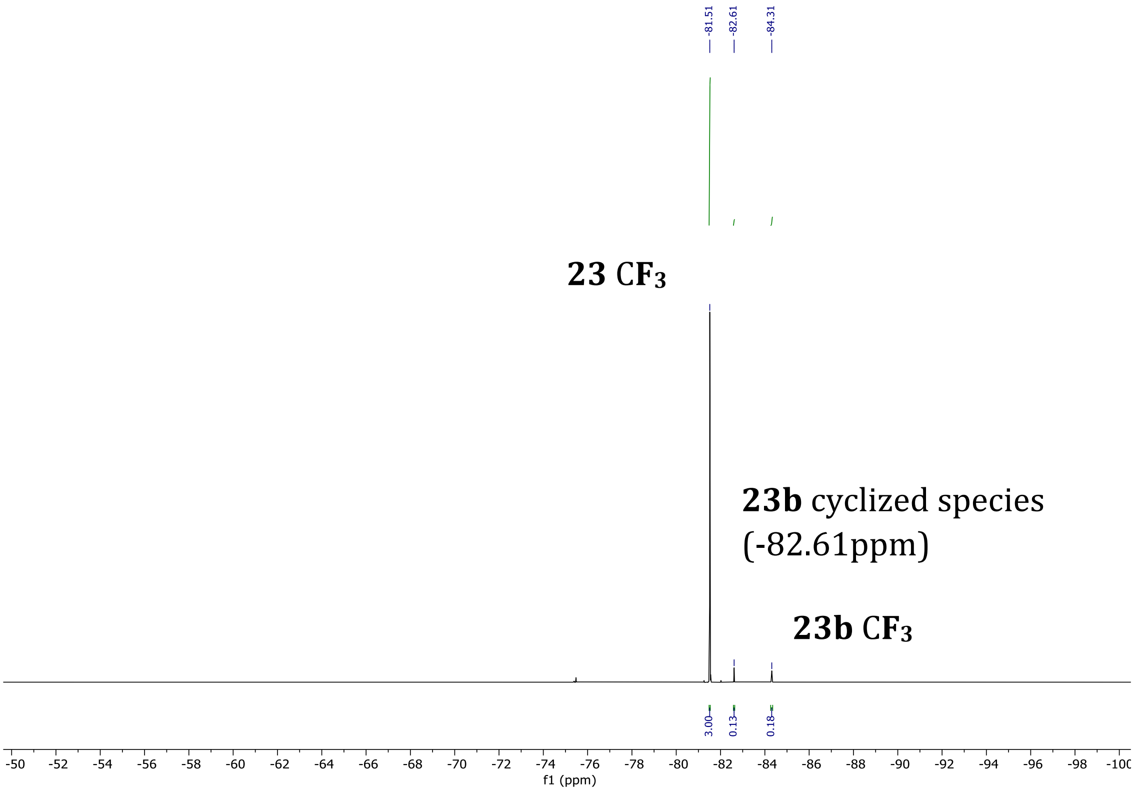


Figure S139: $^{19}$F NMR of an FSA biotransformation of trifluoropyruvaldehyde **23** and **b** at 24h.

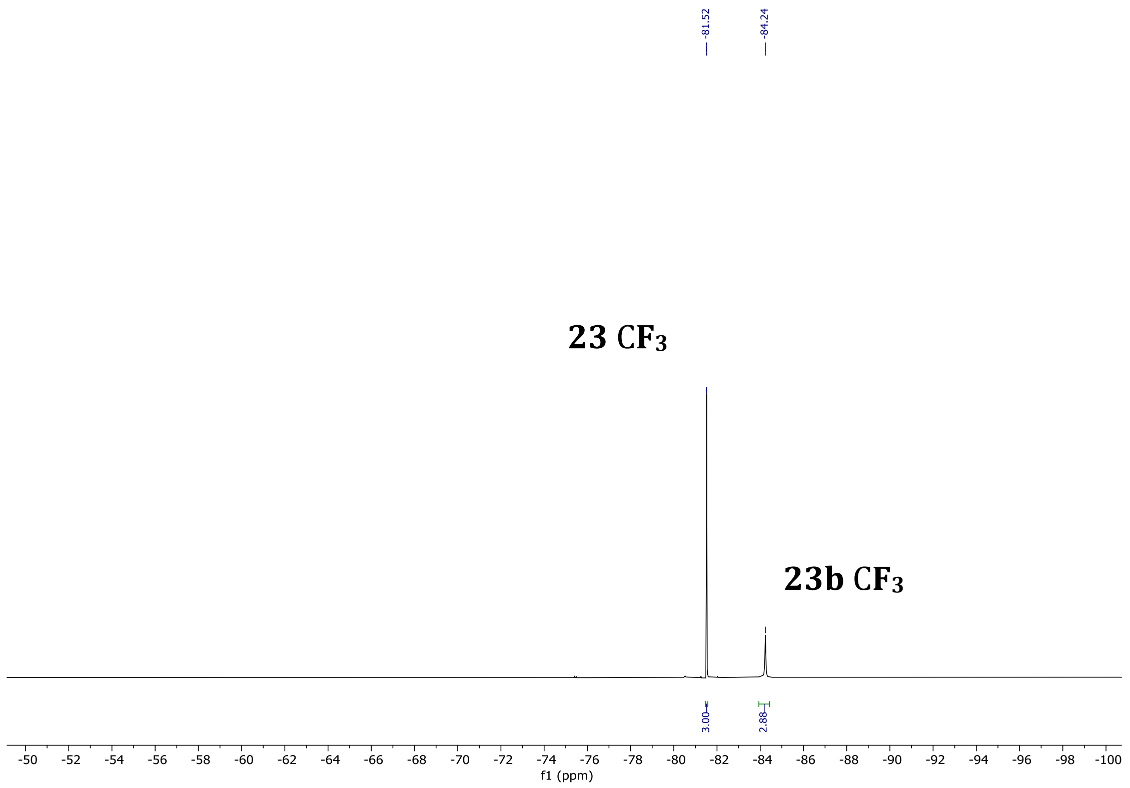


Figure S140: [19]F NMR of an FSA biotransformation of trifluoropyruvaldehyde **23** and **c** at 24h.

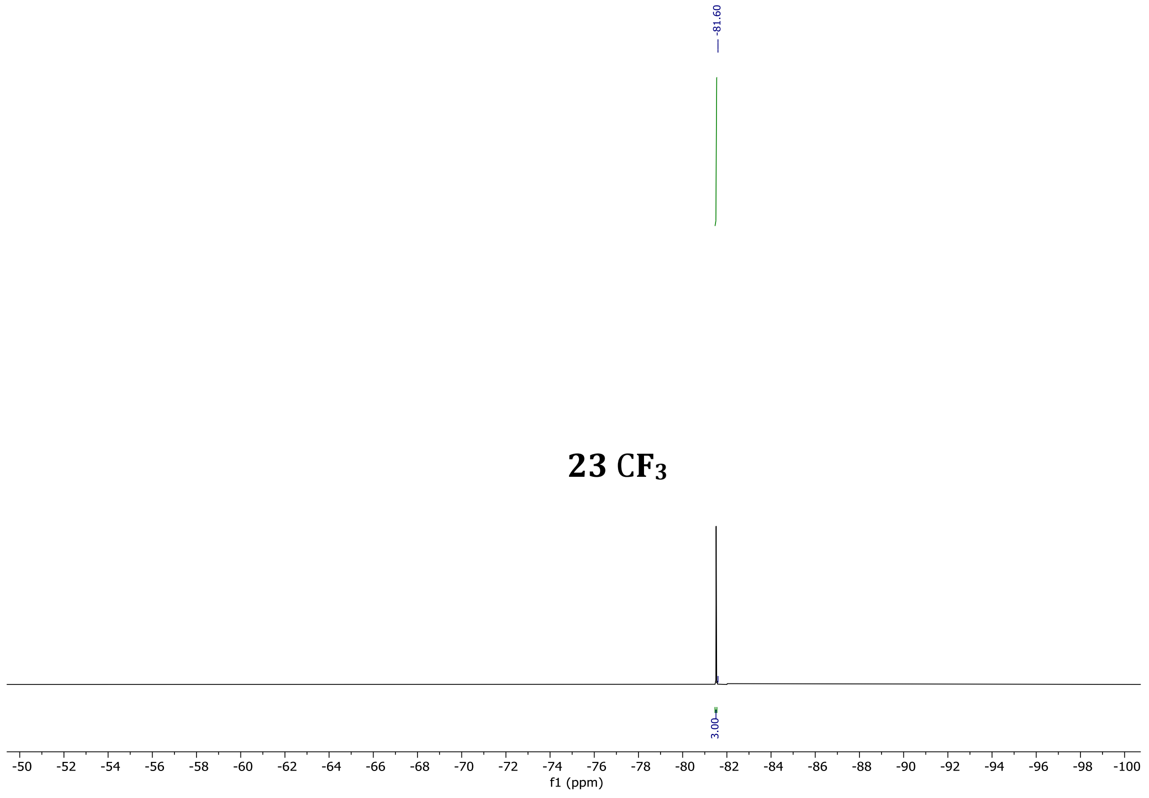


Figure S141: [19]F NMR of a no enzyme control of trifluoropyruvaldehyde **23** and **a** at 24h.

*Time course at increased substrate loading*

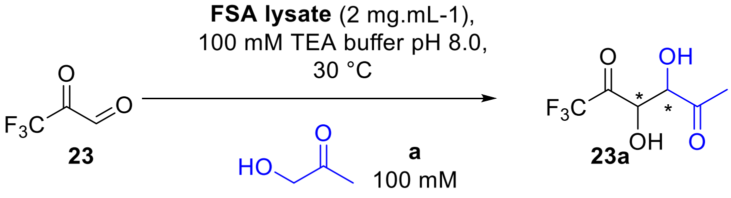


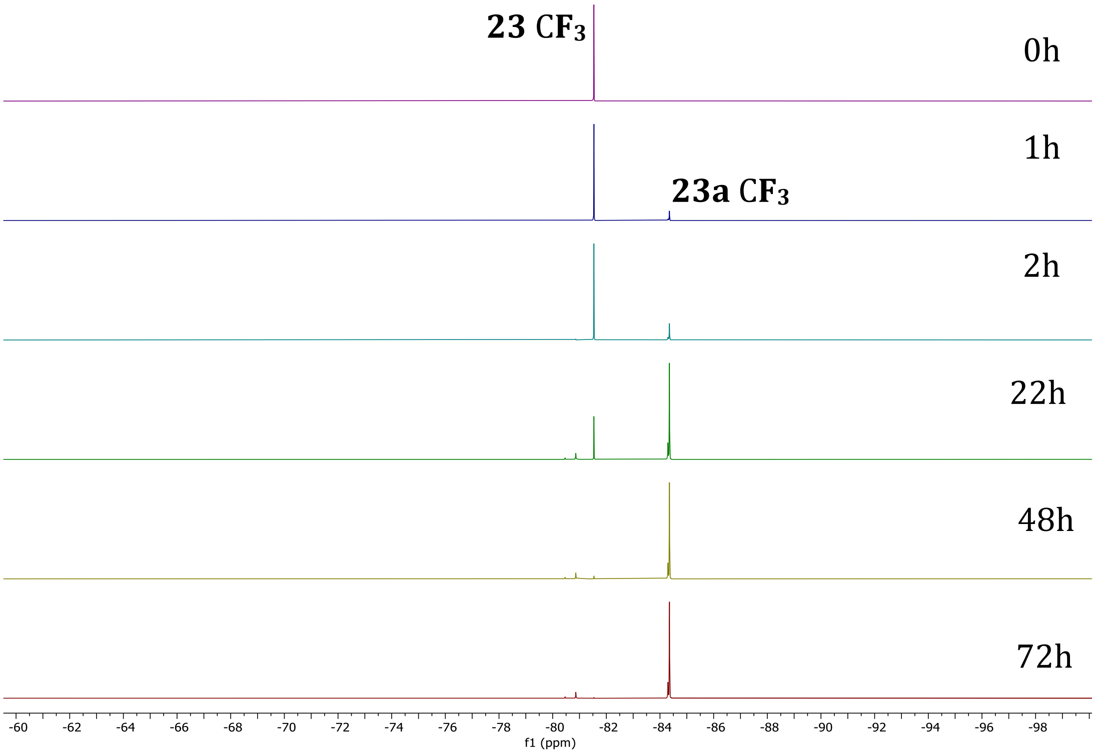


Figure S142: $^{19}F$ NMR data for time course of the *Ec*FSA$_{wt}$ catalyzed reaction of hydroxyacetone **a** and trifluoropyruvaldehyde **23**. Reaction performed at 100 mM substrate concentration with no DMSO.

## *Investigations into alternate enzymatic transformations of aldol adducts.*

### *Screening of metagenomic imine reductases*

A panel of metagenomic imine reductases from Prozomix, which has previously been used effectively for the reductive amination of aldolase derived dihydroxyketones was screened for activity in the reductive amination of **9a** with cyclopropylamine.[10,11] Based on recent work from Turner *et al.* it was anticipated that a single enzyme reductive amination-imine reduction cascade might afford the fully reduced pyrrolidine products.[12]

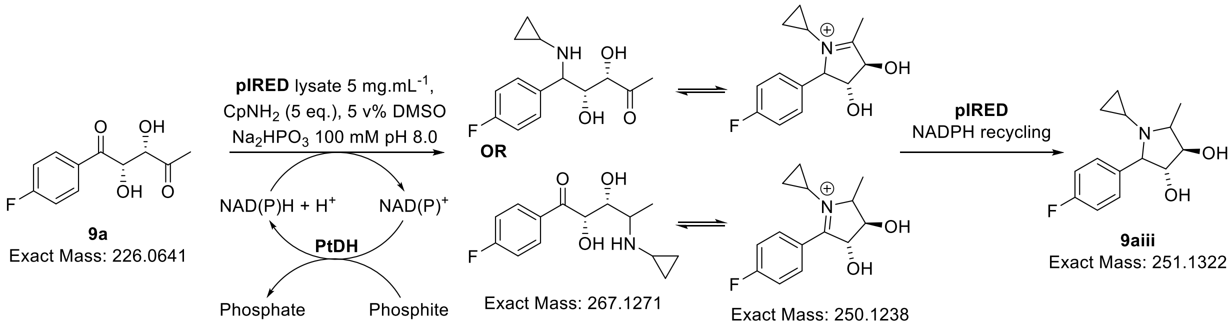


Figure S143: Reaction scheme for the postulated IRED mediated reductive amination-imine reduction cascade of diketone substrate **9a**.

Biotransformations were performed in flat-bottomed 96 well plates with a total reaction volume of 100 μL and contained IRED lysate (5 mg.mL$^{-1}$), diketone substrate **9a** (5 mM), cyclopropylamine (25 mM, 5 eq.), NADP$^+$ (0.5 mM) and PtDH (0.2 mg.mL$^{-1}$) in NaPt buffer (100 mM, pH 8.0) containing 5 v% DMSO. A no enzyme control biotransformation also was performed and a chemical reaction containing diketone substrate (5 mM), cyclopropylamine (25 mM) and $NH_3BH_3$ (50 mM) in NaPt buffer (100 mM, pH 8.0) containing 5 v% DMSO. After 24 hours the biotransformations were filtered and an aliquot taken and diluted 5-fold in water/methanol (95:5) for UPLC-QDa analysis. The chemical reduction reaction showed formation of four new peaks with an *m/z* corresponding to the pyrrolidine product $[M+H]^+$ 252.12. The no enzyme control showed no substrate depletion. Across the IREDs screened, only a handful returned new signals with the corresponding pyrrolidine *m/z*, mostly at trace levels and all below 5% conversion. Additionally, a second, unidentified byproduct peak was observed in these biotransformations and the no enzyme control reaction.

| | 1 | 2 | 3 | 4 | 5 | 6 | 7 | 8 | 9 | 10 | 11 | 12 | 13 | 14 | 15 | 16 | 17 | 18 | 19 | 20 | 21 | 22 | 23 | 24 |
|---|---|---|---|---|---|---|---|---|---|---|---|---|---|---|---|---|---|---|---|---|---|---|---|---|
| A | 1 | 2 | 3 | 4 | 5 | 6 | 7 | 8 | 9 | 10 | 11 | 12 | 13 | 14 | 15 | 16 | 17 | 18 | 19 | 20 | 21 | 22 | 23 | 24 |
| B | 25 | 26 | 27 | 28 | 29 | 30 | 31 | 32 | 33 | 34 | 35 | 36 | 37 | 38 | 39 | 40 | 41 | 42 | 43 | 44 | 45 | 46 | 47 | 48 |
| C | 49 | 50 | 51 | 52 | 53 | 54 | 55 | 56 | 57 | 58 | 59 | 60 | 61 | 62 | 63 | 64 | 65 | 66 | 67 | 68 | 69 | 70 | 71 | 72 |
| D | 73 | 74 | 75 | 76 | 77 | 78 | 79 | 80 | 81 | 82 | 83 | 84 | 85 | 86 | 87 | 88 | 89 | 90 | 91 | 92 | 93 | 94 | 95 | 96 |
| E | 97 | 98 | 99 | 100 | 101 | 102 | 103 | 104 | 105 | 106 | 107 | 108 | 109 | 110 | 111 | 112 | 113 | 114 | 115 | 116 | 117 | 118 | 119 | 120 |
| F | 121 | 122 | 123 | 124 | 125 | 126 | 127 | 128 | 129 | 130 | 131 | 132 | 133 | 134 | 135 | 136 | 137 | 138 | 139 | 140 | 141 | 142 | 143 | 144 |
| G | 145 | 146 | 147 | 148 | 149 | 150 | 151 | 152 | 153 | 154 | 155 | 156 | 157 | 158 | 159 | 160 | 161 | 162 | 163 | 164 | 165 | 166 | 167 | 168 |
| H | 169 | 170 | 171 | 172 | 173 | 174 | 175 | 176 | 177 | 178 | 179 | 180 | 181 | 182 | 183 | 184 | 185 | 186 | 187 | 188 | 189 | 190 | 191 | 192 |
| I | 193 | 194 | 195 | 196 | 197 | 198 | 199 | 200 | 201 | 202 | 203 | 204 | 205 | 206 | 207 | 208 | 209 | 210 | 211 | 212 | 213 | 214 | 215 | 216 |
| J | 217 | 218 | 219 | 220 | 221 | 222 | 223 | 224 | 225 | 226 | 227 | 228 | 229 | 230 | 231 | 232 | 233 | 234 | 235 | 236 | 237 | 238 | 239 | 240 |
| K | 241 | 242 | 243 | 244 | 245 | 246 | 247 | 248 | 249 | 250 | 251 | 252 | 253 | 254 | 255 | 256 | 257 | 258 | 259 | 260 | 261 | 262 | 263 | 264 |
| L | 265 | 266 | 267 | 268 | 269 | 270 | 271 | 272 | 273 | 274 | 275 | 276 | 277 | 278 | 279 | 280 | 281 | 282 | 283 | 284 | 285 | 286 | 287 | 288 |
| M | 289 | 290 | 291 | 292 | 293 | 294 | 295 | 296 | 297 | 298 | 299 | 300 | 301 | 302 | 303 | 304 | 305 | 306 | 307 | 308 | 309 | 310 | 311 | 312 |
| N | 313 | 314 | 315 | 316 | 317 | 318 | 319 | 320 | 321 | 322 | 323 | 324 | 325 | 326 | 327 | 328 | 329 | 330 | 331 | 332 | 333 | 334 | 335 | 336 |
| O | 337 | 338 | 339 | 340 | 341 | 342 | 343 | 344 | 345 | 346 | 347 | 348 | 349 | 350 | 351 | 352 | 353 | 354 | 355 | 356 | 357 | 358 | 359 | 360 |
| P | 361 | 362 | 363 | 364 | 365 | 366 | 367 | 368 | 369 | 370 | 371 | 372 | 373 | 374 | 375 | 376 | 377 | 378 | 379 | 380 | 381 | 382 | 383 | 384 |

Figure S144: 384 well plate heatmap representation of IREDs screened. Green shaded cells indicate a new peak with *m/z* 252.12 was formed with lower than 5% conversion.

Due to the low formation of product peak and the relative success of the transaminase screening these results were not investigated further. However, the enzymes screened in this panel may provide a suitable enzyme scaffold for development of a single enzyme capable of catalyzing challenging reductive amination-imine reduction cascades to form pyrrolidine iminosugars.

| Concentration / mM | Peak Area |
|---|---|
| 1 | 12469436 |
| 0.5 | 6335647 |
| 0.25 | 3162386 |
| 0.125 | 1617523 |
| 0.0675 | 828857 |

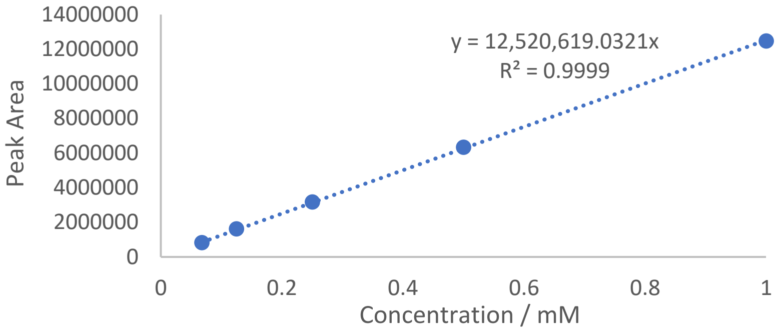


Figure S145: Calibration curve
of compound **9a**. Data collected using UPLC method 3 at 254 nm.

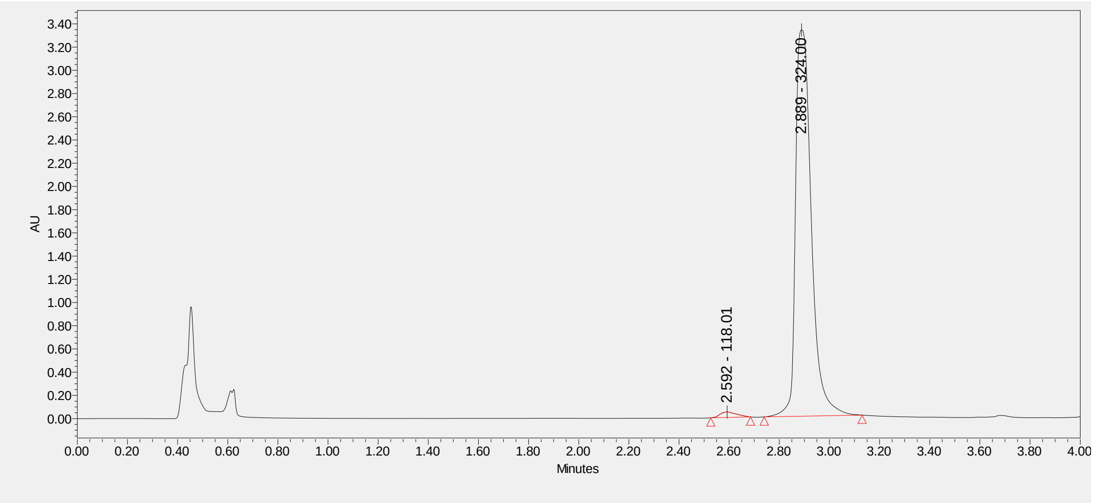


Figure S146: UPLC-QDa trace (method 3) of a biotransformation from the IRED screen of **9a** at 0h.

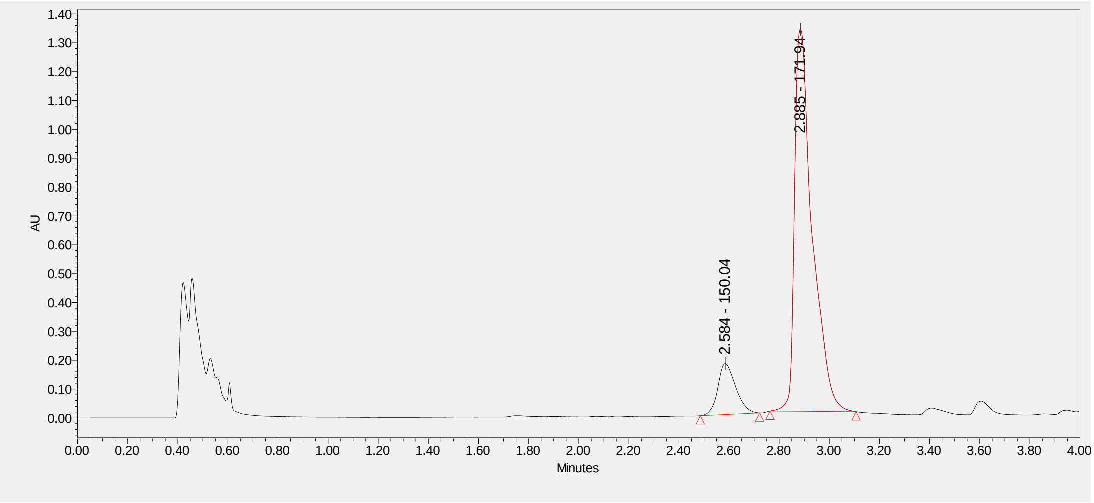


Figure S147: UPLC-QDa trace (method 3) of a no enzyme control biotransformation from the IRED screen of **9a** at 24h.

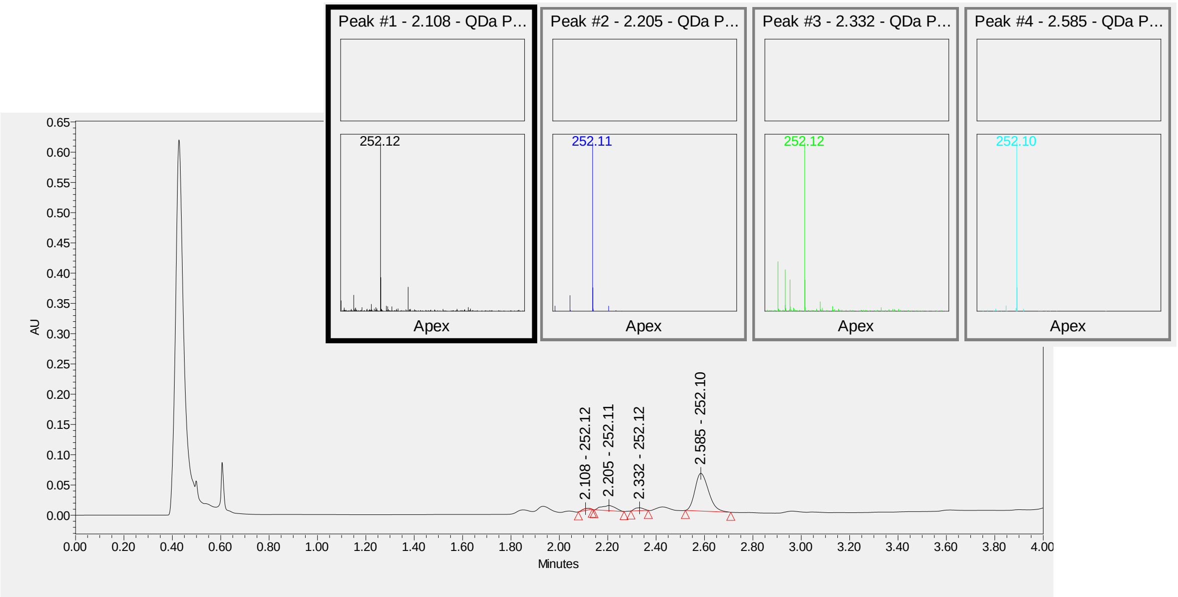


Figure S148: UPLC-QDa trace (method 3) of the chemical reductive amination of **9a** with cyclopropylamine at 24h. Full depletion of the substrate and eight new peaks are observed. Of these, four provide the expected *m/z* of the pyrrolidine product **9aiii** (252.12).

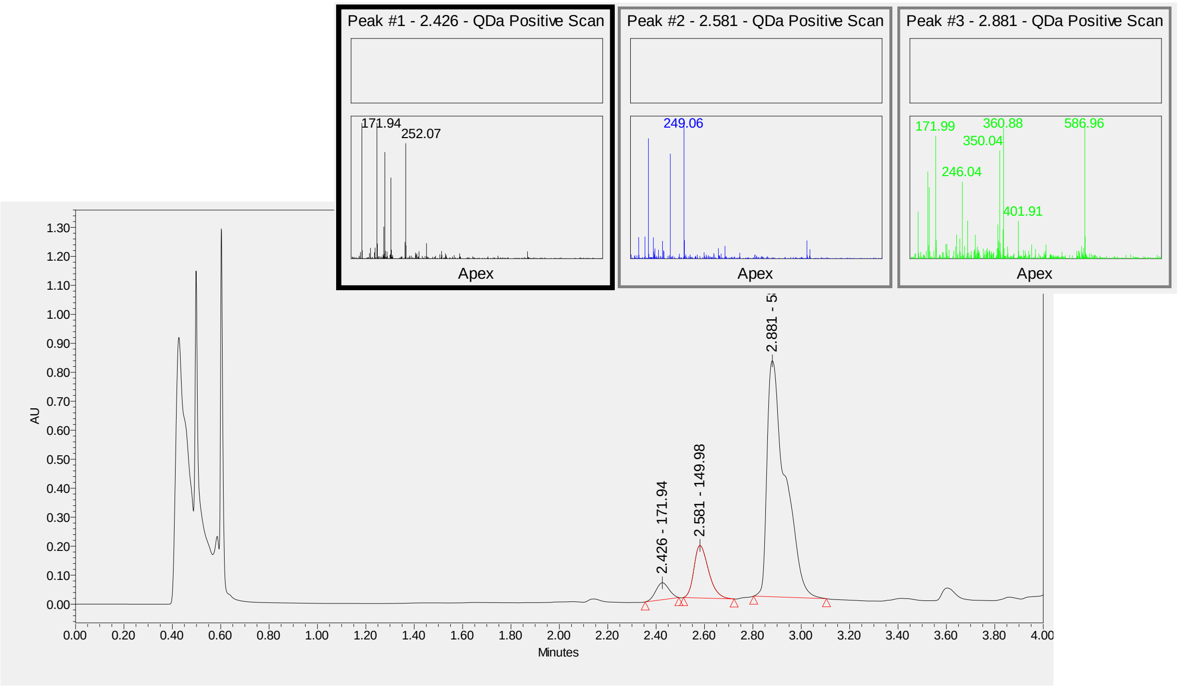


Figure S149: UPLC-QDa trace (method 3) of an example biotransformation (IRED90) showing a new peak with the pyrrolidine **9a** *m/z* (252.12) from the IRED screen of **9a** at 24h. Only 4% conversion of substrate was achieved.

## *References*


(1) Johannes, T. W.; Woodyer, R. D.; Zhao, H. Efficient Regeneration of NADPH Using an Engineered Phosphite Dehydrogenase. *Biotechnology and Bioengineering* **2007**, *96* (1), 18–26. https://doi.org/10.1002/bit.21168.

(2) Zhou, Y.-H.; Zhang, Y.-Z.; Wu, Z.-L.; Cai, T.; Wen, W.; Guo, Q.-X. Organocatalytic Asymmetric Aldol Reaction of Arylglyoxals and Hydroxyacetone: Enantioselective Synthesis of 2,3-Dihydroxy-1,4-Diones. *Molecules* **2020**, *25* (3), 648. https://doi.org/10.3390/molecules25030648.

(3) Kaulmann, U.; Smithies, K.; Smith, M. E. B.; Hailes, H. C.; Ward, J. M. Substrate Spectrum of ω-Transaminase from Chromobacterium Violaceum DSM30191 and Its Potential for Biocatalysis. *Enzyme and Microbial Technology* **2007**, *41* (5), 628–637. https://doi.org/10.1016/j.enzmictec.2007.05.011.

(4) Villegas-Torres, M. F.; Martinez-Torres, R. J.; Cázares-Körner, A.; Hailes, H.; Baganz, F.; Ward, J. Multi-Step Biocatalytic Strategies for Chiral Amino Alcohol Synthesis. *Enzyme and Microbial Technology* **2015**, *81*, 23–30. https://doi.org/10.1016/j.enzmictec.2015.07.003.

(5) Leipold, L.; Dobrijevic, D.; Jeffries, J. W. E.; Bawn, M.; Moody, T. S.; Ward, J. M.; Hailes, H. C. The Identification and Use of Robust Transaminases from a Domestic Drain Metagenome. *Green Chem.* **2019**, *21* (1), 75–86. https://doi.org/10.1039/C8GC02986E.

(6) Cerioli, L.; Planchestainer, M.; Cassidy, J.; Tessaro, D.; Paradisi, F. Characterization of a Novel Amine Transaminase from Halomonas Elongata. *Journal of Molecular Catalysis B: Enzymatic* **2015**, *120*, 141–150. https://doi.org/10.1016/j.molcatb.2015.07.009.

(7) Galman, J.; Gahloth, D.; Parmeggiani, F.; Slabu, I.; Leys, D. Characterisation of a Putrescine Transaminase from Pseudomonas Putida and Its Application to the Synthesis of Benzylamine Derivatives. *Frontiers in Bioengineering and Biotechnology* **2018**.

(8) Behr, J.-B. Synthesis and L-Fucosidase Inhibitory Activity of a New Series of Cyclic Sugar Imines—in Situ Formation and Assay of Their Saturated Counterparts. *Tetrahedron Letters* **2009**, *50* (31), 4498–4501. https://doi.org/10.1016/j.tetlet.2009.05.075.

(9) Subrizi, F.; Benhamou, L.; Ward, J. M.; Sheppard, T. D.; Hailes, H. C. Aminopolyols from Carbohydrates: Amination of Sugars and Sugar-Derived Tetrahydrofurans with Transaminases. *Angewandte Chemie International Edition* **2019**, *58* (12), 3854–3858. https://doi.org/10.1002/anie.201813712.

(10) Marshall, J. R.; Yao, P.; Montgomery, S. L.; Finnigan, J. D.; Thorpe, T. W.; Palmer, R. B.; Mangas-Sanchez, J.; Duncan, R. A. M.; Heath, R. S.; Graham, K. M.; Cook, D. J.; Charnock, S. J.; Turner, N. J. Screening and Characterization of a Diverse Panel of Metagenomic Imine Reductases for Biocatalytic Reductive Amination. *Nat. Chem.* **2021**, *13* (2), 140–148. https://doi.org/10.1038/s41557-020-00606-w.

(11) Ford, G. J.; Swanson, C. R.; Bradshaw Allen, R. T.; Marshall, J. R.; Mattey, A. P.; Turner, N. J.; Clapés, P.; Flitsch, S. L. Three-Component Stereoselective Enzymatic Synthesis of Amino-Diols and Amino-Polyols. *JACS Au* **2022**, *2* (10), 2251–2258. https://doi.org/10.1021/jacsau.2c00374.

(12) Ramsden, J. I.; Zucoloto da Costa, B.; Heath, R. S.; Marshall, J. R.; Derrington, S. R.; Mangas-Sanchez, J.; Montgomery, S. L.; Mulholland, K. R.; Cosgrove, S. C.; Turner, N. J. Bifunctional Imine Reductase Cascades for the Synthesis of Saturated N-Heterocycles. *ACS Catal.* **2024**, *14* (19), 14703–14710. https://doi.org/10.1021/acscatal.4c03832.
